# Activation of mTOR signaling in adult lung microvascular progenitor cells accelerates lung aging

**DOI:** 10.1172/JCI171430

**Published:** 2023-12-15

**Authors:** Emma C. Mason, Swapna Menon, Benjamin R. Schneider, Christa F. Gaskill, Maggie M. Dawson, Camille M. Moore, Laura Craig Armstrong, Okyong Cho, Bradley W. Richmond, Jonathan A. Kropski, James D. West, Patrick Geraghty, Brigitte N. Gomperts, Kevin C. Ess, Fabienne Gally, Susan M. Majka

**Affiliations:** 1 Department of Medicine, Division of Pulmonary, Critical Care and Sleep Medicine, National Jewish Health, Denver, Colorado, USA.; 2 Pulmonary Vascular Research Institute Kochi and AnalyzeDat Consulting Services, Kerala, India.; 3 Department of Dermatology, Vanderbilt University Medical Center, Nashville, Tennessee, USA.; 4 Department of Immunology and Genomic Medicine, Center for Genes, Environment and Health, National Jewish Health, Denver, Colorado, USA.; 5 Department of Biostatistics and Informatics, University of Colorado Anschutz Medical Campus, Aurora, Colorado, USA.; 6 Division of Pediatric Neurology, Department of Pediatrics, Vanderbilt University Medical Center, Nashville, Tennessee, USA.; 7 Genomics and Microarray Core, University of Colorado Cancer Center, Anschutz Medical Center, Aurora, Colorado, USA.; 8 Department of Medicine, Division of Allergy, Pulmonary and Critical Care Medicine, Vanderbilt University Medical Center and Department of Veterans Affairs, Nashville, Tennessee, USA.; 9 Division of Pulmonary and Critical Care Medicine, SUNY Downstate Medical Center, Brooklyn, New York, USA.; 10 Translational Research, UCLA Broad Stem Cell Research Center; Pediatrics Division of Pulmonary Medicine, University of California, Los Angeles, California, USA.; 11 Gates Center for Regenerative Medicine and Stem Cell Biology, University of Colorado, Aurora, Colorado, USA.

**Keywords:** Stem cells, Vascular Biology, Adult stem cells, Endothelial cells, Microcirculation

## Abstract

Reactivation and dysregulation of the mTOR signaling pathway are a hallmark of aging and chronic lung disease; however, the impact on microvascular progenitor cells (MVPCs), capillary angiostasis, and tissue homeostasis is unknown. While the existence of an adult lung vascular progenitor has long been hypothesized, these studies show that Abcg2 enriches for a population of angiogenic tissue-resident MVPCs present in both adult mouse and human lungs using functional, lineage, and transcriptomic analyses. These studies link human and mouse MVPC-specific mTORC1 activation to decreased stemness, angiogenic potential, and disruption of p53 and Wnt pathways, with consequent loss of alveolar-capillary structure and function. Following mTOR activation, these MVPCs adapt a unique transcriptome signature and emerge as a venous subpopulation in the angiodiverse microvascular endothelial subclusters. Thus, our findings support a significant role for mTOR in the maintenance of MVPC function and microvascular niche homeostasis as well as a cell-based mechanism driving loss of tissue structure underlying lung aging and the development of emphysema.

## Graphical abstract

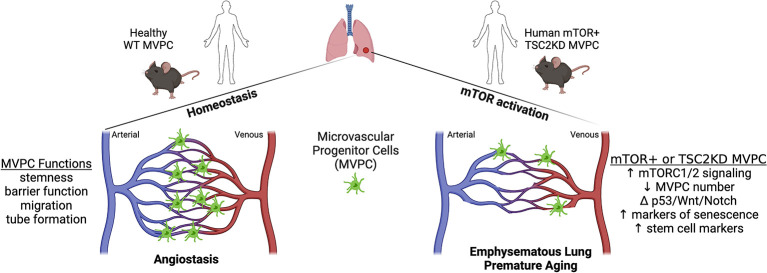

## Introduction

The development of lung diseases, including chronic obstructive pulmonary disease (COPD)/emphysema associated with cigarette smoke exposure, is likened to accelerated aging of the lung, because the environmental exposures enhance oxidative stress and chronic inflammation that drive loss of tissue structure and function (1–3). While most studies of lung aging have focused on the contribution of dysfunctional epithelium, epithelial progenitors, and fibroblasts (3–7) to chronic lung diseases (CLDs), little is known about the underlying vascular changes or remodeling that contribute to these processes. We have previously demonstrated that Abcg2^+^ microvascular progenitor cells (MVPCs) are enriched for rigorous angiogenic ability and play a role in homeostasis or adaptive angiogenesis associated with injury or CLDs (8–12). Such CLDs are also linked to dysregulated mammalian target of rapamycin (mTOR) signaling (13–21). While adult MVPC function is necessary for microvascular homeostasis, it is not known how increased mTOR signaling in MVPCs impacts progenitor function or increased susceptibility to CLDs.

Activation of the mTOR pathway is implicated in the accumulation of senescent cells during COPD, contributing to the development of emphysema by limiting tissue repair (13). Conversely, mTOR is implicated in the pathogenesis of pulmonary fibrosis impacting fibroblast proliferation, survival, and differentiation (14). In the multiprotein mTORC1 complex, mTOR activity is regulated by tuberous sclerosis complex 2 (TSC2) and the binding partner, TSC1 (22). Loss of TSC2 function leads to activation of mTOR signaling that may result in abnormal somatic or progenitor cell differentiation and proliferation as well as senescence, in some instances driving tissue remodeling, compromising organ function, and limiting repair (13, 18–21). Tissue remodeling as a consequence of deregulated *TSC2* expression and/or function in the lung may include abnormal vasculature, loss of epithelial structure, smooth muscle cell accumulation, and formation of nonmalignant tumors as described in tuberous sclerosis and lymphangioleiomyomatosis (LAM) (15–17).

To date, most preclinical studies to define cell and mechanistic origins of tissue remodeling due to TSC2 dysfunction have been performed with developmental models (15, 23–28). Paracrine and heterotypic cell-cell interactions in the pathogenesis of Tsc2-driven tissue remodeling are strongly supported in rodent models and primary human cells by heterogeneous increased expression of the mTOR target p-S6, indicating non-uniform mTOR activation (29–31). Additionally, adoptive transplantation of human TSC/LAM cells (32, 33) or rat uterine tumor-derived smooth muscle cells (34) into immunocompromised recipient mouse models demonstrated loss of alveolar structure. These studies provide evidence that cell-extrinsic TSC2 function or mTOR signaling plays an important role in lung tissue homeostasis and remodeling; however, they do not provide a clear cell-based paracrine mechanism for adult pulmonary remodeling. In contrast, cell-specific knockdown of *Tsc1* in developing mouse endothelium and adult mouse alveolar epithelium or endothelium-activated mTOR signaling, resulting in abnormal angiogenesis and smooth muscle differentiation, increased cellular senescence and the development of emphysematous phenotype (13, 35), indicative of both cell-intrinsic and -extrinsic effects (35).

Our current studies build on these findings and test the hypothesis that mTORC1 activation via Tsc2 depletion in adult lung MVPCs negatively impacts progenitor function in the microvascular niche. In complementary mouse and human functional and transcriptomic studies, we demonstrate that adult lung Abcg2 MVPCs are of veinous origin microvascular progenitor pool. mTORC1 activation in MVPCs disrupted microvascular homeostasis, resulting in simplified alveolar structure and function with deregulation of multiple developmental signaling pathways, including mTORC2. Together these studies highlight the need for a more detailed understanding of how mTOR activation in MVPCs impacts both the microvascular and alveolar epithelial niche via regulation of downstream signaling pathways.

## Results

### Human mTORC1-activated MVPCs disrupt lung microvascular endothelial barrier function and tissue structure.

**Figure 1:**
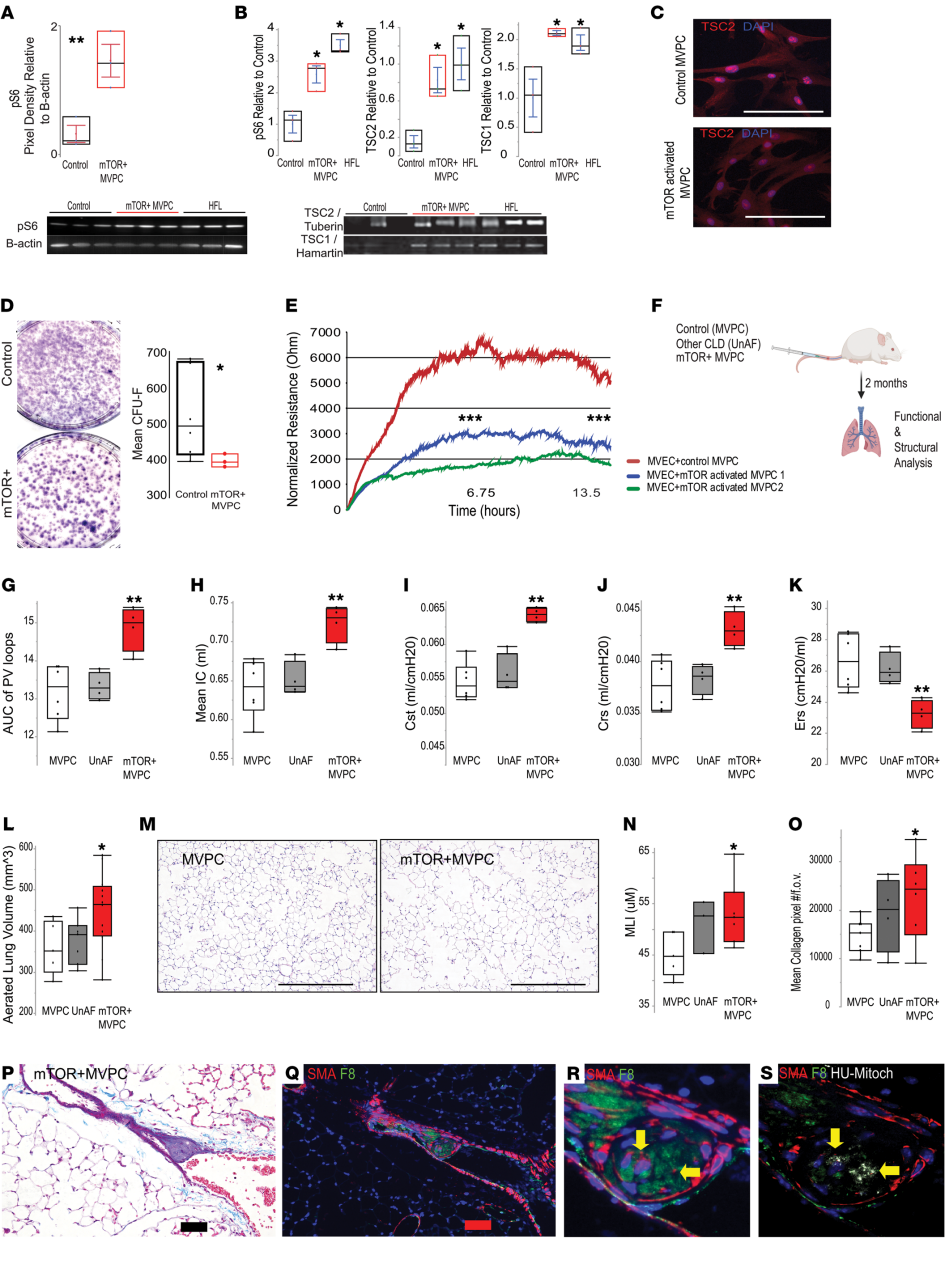
mTOR-activated lung MVPCs negatively impact progenitor function and alveolar structure. (**A**) Expression of p-S6 levels as an indication of mTOR signaling by Western blot analysis in isolated normal (3 independent patient primary cell lines) or LAM MVPCs (mTOR+; 1 patient, 3 independent cell lines, all female) normalized to total protein and β-actin. (**B**) Expression of p-S6, TSC2, and TSC1 normalized to total protein and β-actin relative to control MVPCs respectively by Western blot analysis in normal (3–4 independent patient primary cell lines, 2 female, 2 male, age 60–67), mTOR+ (1 patient, 3 independent cell lines, age 63), and fetal (human fetal lung [HFL]; 3 independent patient primary cell lines, 17–20 weeks of gestation, 2 male, 1 female) MVPCs. Data were analyzed by 1-way ANOVA followed by Tukey’s honestly significant difference (HSD) post hoc analysis and are presented as mean ± SEM. *P < 0.05, **P < 0.01. (**C**) Immunostaining to localize TSC2 (red) in control and mTOR+ MVPCs. Scale bars: 100 μm. (**D**) Representative images of colony-forming unit fibroblasts (CFU-F) on day 10. (**E**) Electric Cell-Substrate Impedance Sensing analysis of endothelial barrier function recovery following injury in the presence of control MVPC or two mTOR+ MVPC lines. ***P < 0.001. (**F**) To create humanized mice, we adoptively transferred 500,000 age-matched (age 60–66 years) female human MVPCs from a healthy control (*n* = 5 recipient mice), unaffected (non-cystic lung; 2 independent COPD patient cell lines, *n* = 3 recipient mice per line), mTOR+ (cystic lung; 3 independent cell lines, *n* = 5, 3, and 3 recipient mice), or HBSS vehicle control (*n* = 9 recipient mice) via tail vein of recipient female NSG mice (age 14 weeks). This study was repeated twice independently. Lung function and structure were analyzed at 2 months. (**G**–**K**) Airway physiology was measured using flexiVent analyses. (**G**) Area under the curve of pressure/volume (PV) loops. (**H**) Mean inspiratory capacity (IC). (**I**) Quasi-static compliance (Cst). (**J**) Respiratory system compliance (Crs). (**K**) Respiratory system elastance (Ers). (**L**) Aerated lung volume determined by micro-CT. (**M**) Representative H&E staining. Scale bars: 100 μm. (**N**) Mean linear intercept (MLI). (**O**) Collagen deposition quantified by trichrome stain and Fiji (NIH) analysis. Data were analyzed by 1-way ANOVA followed by Tukey’s HSD post hoc analysis and are presented as mean ± SEM. *P < 0.05, **P < 0.01. (**P**–**S**) Lung vascular lesions were identified at 2 months and trichrome stained to detect collagen (**P**) and immunostained to localize α-smooth muscle actin (SMA, red) and factor 8 (F8, green) (**Q**). (**R**) Enlarged image. (**S**) Costaining to detect human mitochondrial antigen (white). Scale bars: 100 μm.

mTOR signaling regulates vascular development, angiogenesis, and progenitor fate (13, 18–21, 36). Therefore, MVPCs represent a target to elucidate the mechanism by which enhanced mTOR signaling disrupts the microvascular niche and loss of epithelial progenitor or tissue function (37). MVPCs were isolated from explanted normal (*n* = 3–4) or LAM (*n* = 1; 3 independent cell lines) lung tissue and characterized as previously described (Supplemental Figure 1, A and B; supplemental material available online with this article; https://doi.org/10.1172/JCI171430DS1) (11, 12, 38–41). LungMAP (https://lungmap.net) was used to localize Abcg2 MVPCs in human capillary subsets and demonstrated that they are largely associated with the capillary 1 (Cap1) population (Supplemental Figure 1C). We compared human adult MVPCs relative to those isolated from human fetal lung (*n* = 3), since developmental signaling pathways are co-opted during adult diseases (42) and Tsc2/mTOR signaling is characterized as integral during lung vascular and epithelial embryogenesis (43). Primary human lung MVPCs were analyzed by Western blot to detect the expression of the binding partners TSC1 and TSC2 and phosphorylation of 70S6 (p-S6) as an indirect measure of mTORC1. Western blot analysis and immunofluorescence staining confirmed mTOR signaling activation in LAM MVPCs relative to WT via increased levels of the mTOR target p-S6 (Figure 1, A and B, and Supplemental Figure 1D). We thus refer to the MVPCs derived from a LAM patient as mTOR activated (mTOR+). The mTOR+ MVPCs did express TSC1 and TSC2 protein at levels significantly different from the control and fetal lung MVPCs (Figure 1B). TSC2 was localized to the cytoplasm and cell nuclei of control and mTOR+ MVPCs (Figure 1C), while p-S6 was localized in the cytoplasm (Supplemental Figure 1D). Genomic sequencing of the TSC2 loci in the mTOR+ MVPCs identified several small nucleotide polymorphisms previously identified in sporadic pulmonary LAM tissues using deep sequencing (44) in the absence of loss of heterozygosity (Supplemental Table 1) (29, 45). Karyotyping confirmed chromosomal integrity (Supplemental Figure 2, A and B), and proliferation did not significantly differ between cell lines (Supplemental Figure 1E). As TSC2/tuberin protein is expressed by cells that comprise tuberous sclerosis or LAM lesions as well as cells adjacent to the lesions (29), it is likely that TSC2 function is compromised in these mTOR+ MVPCs and may be more important than the presence or absence of protein in the regulation of mTOR signaling (29–31).

To define a functional consequence of mTOR activation (mTOR+) in MVPCs, colony-forming unit fibroblast (CFU-F) analysis was performed to evaluate the clonal progenitor potential. CFU-F identified a decrease in clonal expansion potential or stemness (Figure 1D). Next, the impact of mTOR+ MVPCs on migration, repair, and integrity of microvascular endothelial cell (MVEC) barrier function was quantified (Figure 1E). Control or mTOR+ MVPCs were added to an established monolayer of WT lung MVECs, and electrical wounding was performed with Electric Cell-Substrate Impedance Sensing. The recovery of barrier function was measured over time by the detection of resistance. The cultures with control MVPCs resumed barrier function at a faster rate relative to the mTOR+ groups, which did not reach control levels after 13.5 hours. These results suggest that the mTOR+ MVPCs have a detrimental effect on endothelial barrier function and response to injury.

A role for TSC2-dependent paracrine and heterotypic cell-cell interactions in lung remodeling is supported by our in vitro analyses as well as rodent models (15, 29–31, 35, 46). To examine how mTORC1 activation in MVPCs impacts lung tissue structure, adoptive transfer of primary human MVPCs to immunocompromised recipient mice was performed (Figure 1F) (38, 39). Age-matched (60–66 years of age) female human MVPCs from healthy, unaffected (non-cystic lung) or mTOR+ activated (cystic lung) tissue were injected into the tail vein of recipient female NOD *scid* gamma (NSG) mice at 14 weeks of age. Pulmonary function analyses demonstrated significant loss of tissue elasticity in the recipients of mTOR+ MVPCs relative to healthy control or unaffected donor lines (Figure 1, G–K), which correlated with micro-CT data indicating significantly increased aerated lung volume (Figure 1L) at 2 months after cell transfer. Histological analyses confirmed loss of alveolar complexity with increasing mean linear intercept (MLI) and accumulation of interstitial collagen (Figure 1, M–O). Pulmonary hypertension and vascular leak were not detected (Supplemental Figure 3, A and B). Adoptive transfer of mTOR+ MVPCs did not result in significant donor-derived proliferative lesions as previously reported for other mTOR+ cell types (16, 32–34) and proliferative fibroblasts from fibrotic lungs (47). However, intraluminal vascular lesions were identified (Figure 1, P–R, and Supplemental Figure 3C). The abnormal collections of cells expressed factor 8 (F8), colocalized with collagen, and were encapsulated by smooth muscle actin–expressing cells. Cells in the lesions were donor derived, as they expressed human mitochondrial antigen (Figure 1S). These studies demonstrated that the loss of alveolar structure is a consequence of exogenous non-lung-derived cell effects of mTOR+ MVPCs and provide evidence that cell-extrinsic mTOR signaling plays an important role in tissue remodeling.

### mTOR activation in MVPCs disrupts multiple developmental signaling pathways.

**Figure 2:**
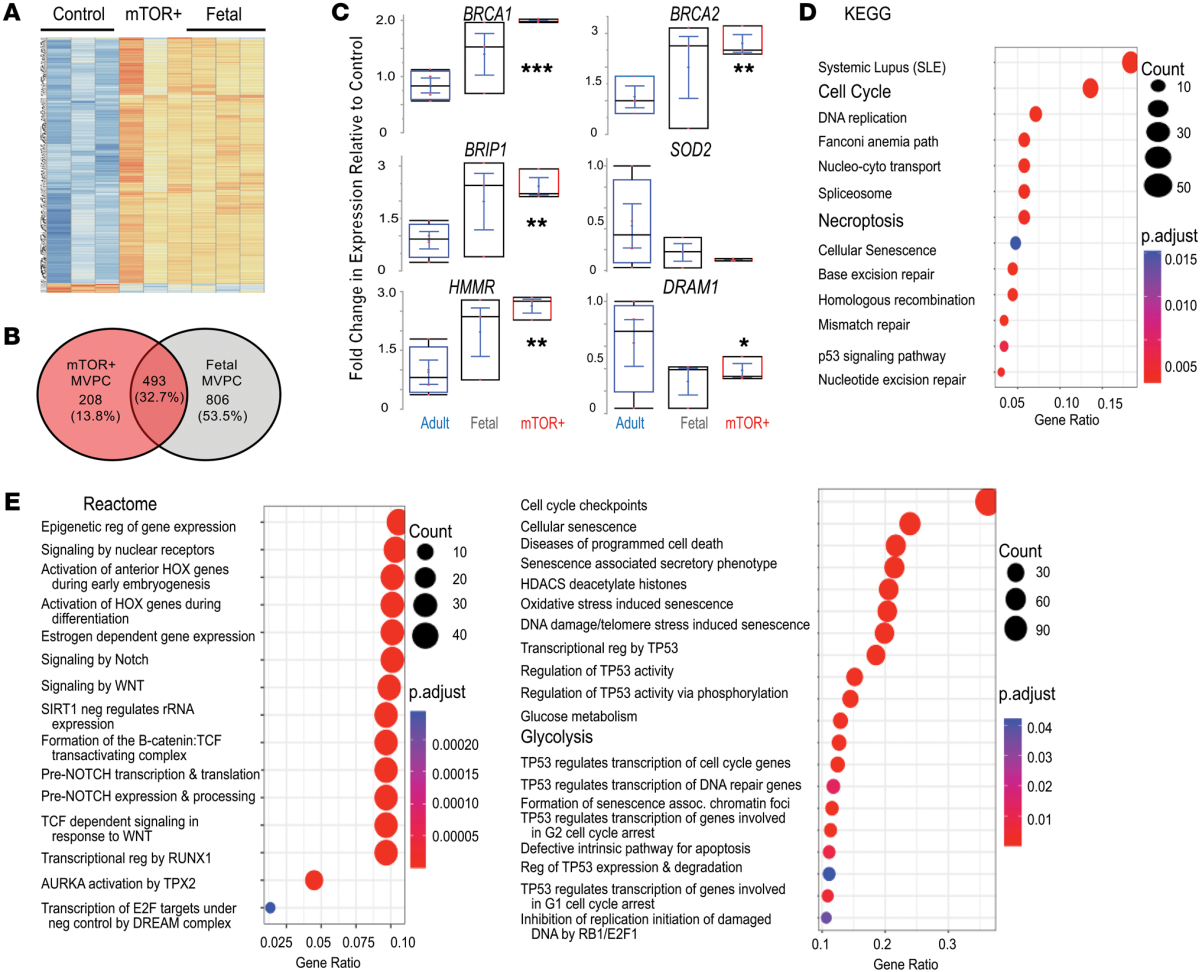
mTOR-activated human lung MVPCs demonstrate similarities to fetal MVPC gene signature and activation of developmental signaling pathways. (**A**) Array analysis was performed using Affymetrix Human Gene 1.0 ST chips to compare human lung MVPCs isolated from explanted patient lungs or fetal lung fibroblast cultures: normal (3–4 independent patient primary cell lines, 2 female, 2 male, age 60–67), mTOR+ (1 patient, 3 independent cell lines, age 63 years), and fetal (HFL; 3 independent patient primary cell lines, 17–20 weeks of gestation, 2 male, 1 female) MVPCs. Differential expression analysis was carried out using Bioconductor (v3.2, R 3.2.2). A minimal fold change of 1.7, up or down, and *P* < 0.05 were used as criteria for defining differentially expressed genes. Expression values for these genes are represented in a heatmap. (**B**) Venn diagram of differentially expressed genes (DEGs) from fetal or mTOR+ MVPCs relative to control. (**C**) Reverse transcriptase PCR analysis was performed using equal amounts of cDNA from independent MVPC lines to validate the array findings. Each patient sample was analyzed in triplicate, standardized to GAPDH, and normalized to control presented in lane 1 set to 1. Control, blue; fetal, gray; mTOR+ samples, red. *n*= 3–4, 3, 3. Asterisks represent *P* values comparing adult with mTOR+ MVPCs. Data were analyzed by nonparametric Wilcoxon/Kruskal-Wallis test and a χ^2^ approximation and are presented as mean ± SEM. *P < 0.05, **P < 0.01, ***P < 0.001. (**D** and **E**) KEGG (**D**) and Reactome (**E**) analyses. Dot plot showing significantly enriched pathways and Reactome functional categories in DEG lists from mTOR+ versus control. The color scale represents the adjusted *P* values obtained for the enrichment of the category in each gene list.

To elucidate pathways in human MVPCs that were deregulated by mTOR activation, transcriptome expression analysis via microarray comparing primary human lung MVPCs from normal control, mTOR+, and fetal lung was performed. After normalization, hierarchical clustering analyses were presented as a heatmap (Figure 2A and Supplemental Figure 4A), illustrating significant differences between the mTOR+ MVPCs and normal controls. Interestingly, mTOR+ MVPC patterns of gene expression overlapped approximately 33% with the fetal lung MVPCs (Figure 2B). Differentially expressed transcripts were selected for validation based on their role during vascular endothelial and smooth muscle cell homeostasis, angiogenesis, and tumorigenesis in multiple organ systems and represented pathways including p53/DNA repair/autophagy/tumor suppression (*BRCA1*, *BRCA2*, *BRIP1*, *DRAM1*, *FoxM1*) (48–50), oxidant stress and cell senescence (*SOD2*), and cell motility/inflammation (*HMMR*) (Figure 2C, Supplemental Figure 1F, and Supplemental Figure 4A). The increased expression of *BRCA1*/*BRCA2*/*BRIP1* and decrease in *DRAM1* in the mTOR+ MVPCs relative to control suggested that p53 signaling was altered. Further analysis of differentially expressed gene lists using the Kyoto Encyclopedia of Genes and Genomes (KEGG) and Reactome showed significantly enriched functional categories and pathways in mTOR+ versus control MVPCs (Figure 2, D and E, and Supplemental Figure 1G). The gene ratio presented indicates the number of differentially expressed genes in a pathway annotation relative to the total number of differentially expressed genes, illustrating significantly affected annotations. KEGG analysis broadly defined differences in DNA replication/repair and p53 signaling pathway between the groups (Figure 2D). Reactome analyses highlighted more granular targets and pathways including HOX, Notch, Wnt, RUNX, TPX2, metabolism, cell cycle, senescence, and p53 (Figure 2E and Supplemental Tables 2 and 3). Western blot analysis was performed to analyze protein expression levels in the mTOR+ versus control MVPCs specific to senescence, cell cycle arrest, and mTORC2 activity (Supplemental Figure 4B). No significant differences in expression of p53, MDM2, p16, and p21 (51) were detected, further supporting the proliferation assay result (Supplemental Figure 1E). Interestingly, increased mTORC2 activity was identified using p-AKT^Ser473^ in the mTOR+ MVPCs isolated from the most significantly affected areas of cystic lung tissue (*n* = 2). Taken together these data suggest that activation of mTORC1 signaling in MVPCs negatively impacts their function and may negatively impact lung microvascular homeostasis and tissue structure as well as dysregulate additional signaling pathways such as mTORC2, Wnt, p53, and Notch.

### Increased mTORC1 signaling in mouse MVPCs negatively impacts microvascular homeostasis, tissue structure, and progenitor function.

**Figure 3:**
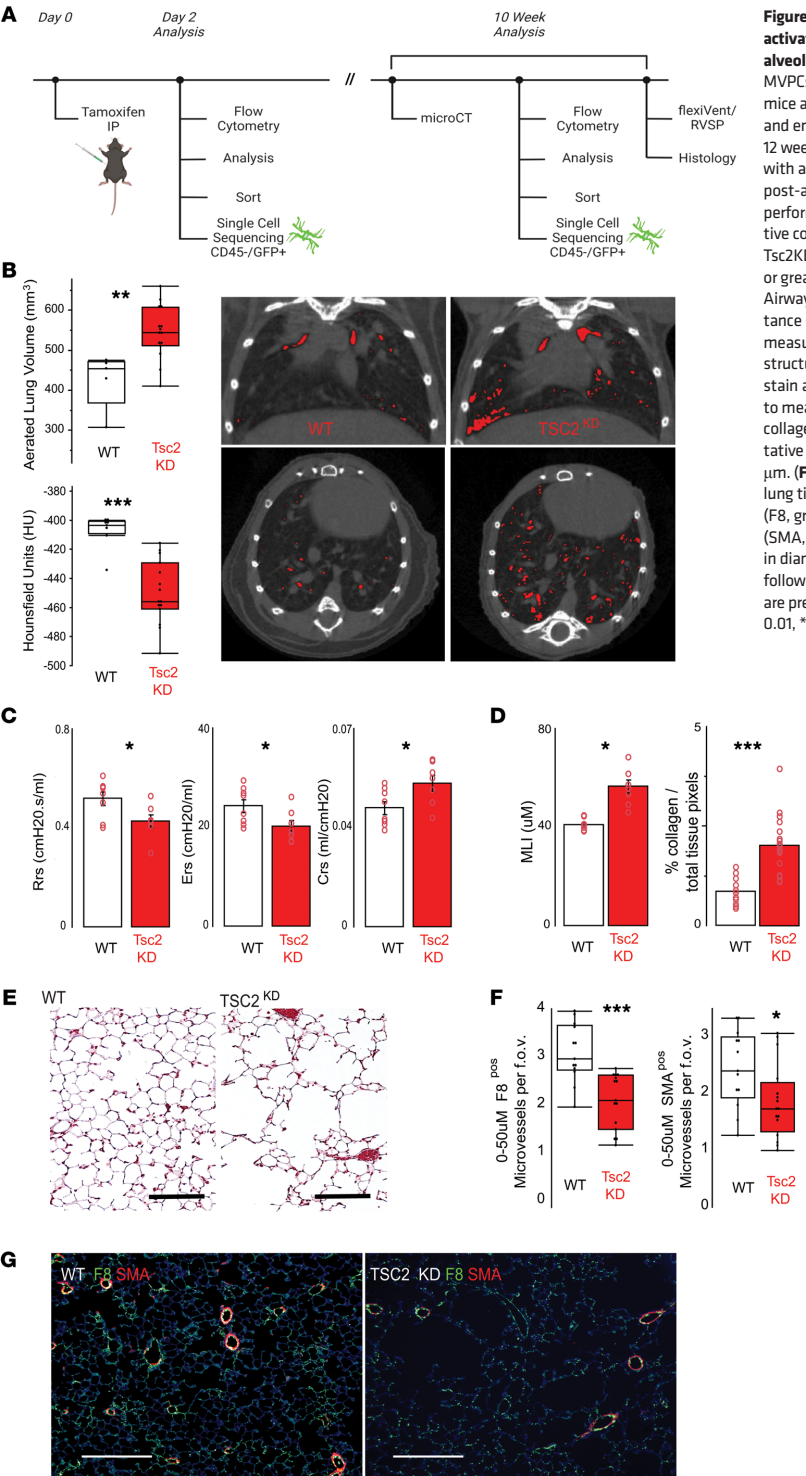
Depletion of *Tsc2* in adult lung MVPCs activates mTOR signaling and drives loss of alveolar structure. (**A**) Depletion of Tsc2 in MVPCs was induced in adult female and male mice at 12 weeks of age with tamoxifen (0.5 mg) and endpoint analysis conducted between 10 and 12 weeks. (**B**) Micro-CT imaging was conducted with a Bruker SkyScan 1276, and quantitative post-analyses of whole lung structure were performed using CTAn software. Representative coronal and axial scans from a WT versus Tsc2KD mouse. Red indicates Hounsfield HU 637 or greater, indicating loss of tissue structure. (**C**) Airway physiology — total resistance (Rrs), elastance (Ers), and system compliance (Crs) — was measured using a SCIREQ flexiVent. (**D**) Lung structure was analyzed using H&E or trichrome stain and quantified using Metamorph and Fiji to measure mean linear intercept (MLI) and total collagen (blue pixels), respectively. (**E**) Representative H&E-stained lung images. Scale bar: 100 μm. (**F** and **G**) Immunostaining was performed on lung tissue sections to detect factor 8–positive (F8, green) and α-smooth muscle actin–positive (SMA, red) microvessels ranging from 0 to 50 μm in diameter. Data were analyzed by 1-way ANOVA followed by Tukey’s HSD post hoc analysis and are presented as mean ± SEM. *P < 0.05, **P < 0.01, ***P < 0.001; *n*= 10–15 mice per group.

To address whether activation of mTORC1 signaling in MVPCs impacts pulmonary structure and function, we engineered a mouse model to deplete Tsc2 in MVPCs by crossing ABCG2-Cre^ERT2^/(Cg)-Gt(ROSA)26Sortm4(ACTB-tdTomato-EGFP) mice (52) with mice harboring a conditional *Tsc2* allele (9, 10, 27, 40, 53), designated as Tsc2 knockdown (Tsc2KD). Endpoint analyses were conducted between 9 and 12 weeks after induction (Figure 3A) (27). Micro-CT analysis illustrated a significant loss of lung parenchymal structure identified as increased aerated lung volume and decreased Hounsfield units (Figure 3B). Analyses of airway function quantified emphysematous-like changes in the Tsc2KD group relative to WT including decreased resistance and elastance and increased compliance (Figure 3C). Histological evaluation showed increased MLI and collagen accumulation in the Tsc2KD group relative to WT and visible loss of surfaces for gas exchange, which supported the functional data (Figure 3, D and E). Alveolar simplification was correlated to decreased microvessel density in the absence of pulmonary hypertension (Figure 3, F and G, and Supplemental Figure 5, A–C).

**Figure 4:**
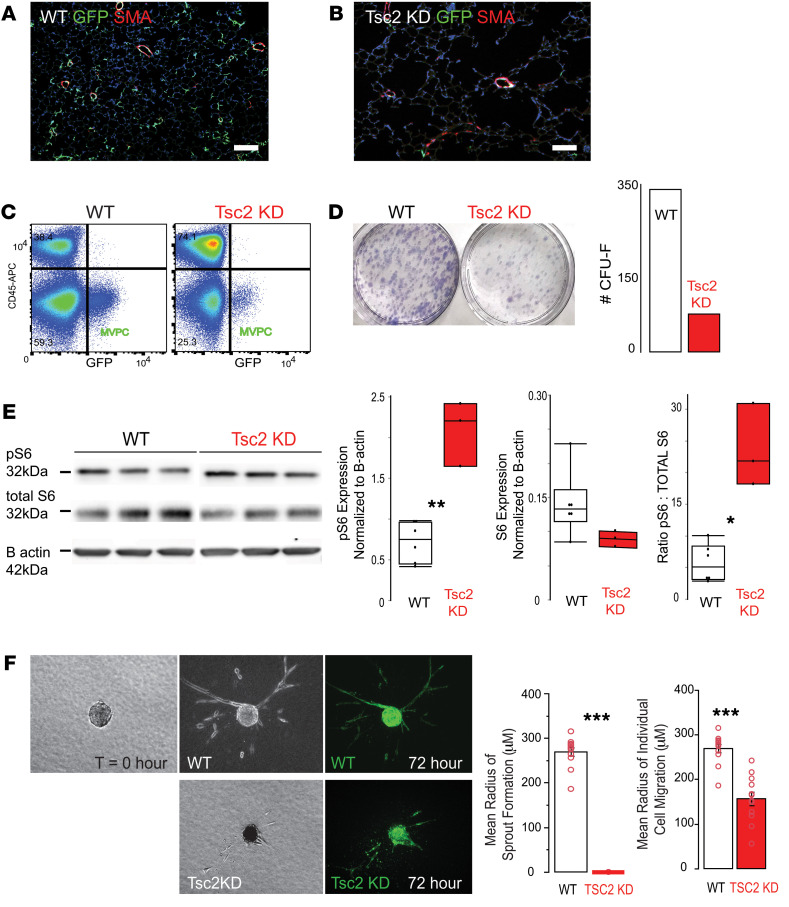
Depletion of *Tsc2* in lung MVPCs decreases number, progenitor expansion, and sprouting while increasing mTOR activity. Depletion of Tsc2 in MVPCs was induced in adult female and male mice at 12 weeks of age with tamoxifen (0.5 mg; *n* = 10–17). (**A** and **B**) Lineage tracing was performed to localize MVPCs at 10 weeks in tissue sections by immunostaining to detect eGFP (green) and α-smooth muscle actin (SMA, red). Scale bars: 100 μm. (**C**) Primary MVPCs were isolated 2 days after induction (Figure 3A) from a pooled sample (*n* = 5 or 8). Representative flow cytometry dot plots of mouse lung single-cell suspension. CD45^–^GFP^+^ MVPCs were sorted from WT or Tsc2KD and cultured to establish primary cell lines from both male and female mice (*n* = 5–8 for each group). (**D**) Colony-forming unit formation. Representative images Giemsa-stained on day 10. (**E**) Quantification of WT and Tsc2KD MVPC levels of p-S6 and total S6 by Western blot was used to determine mTOR activation state (3 independent replicates per group). Data were analyzed by 1-way ANOVA followed by Tukey’s HSD post hoc analysis and are presented as mean ± SEM. (**F**) MVPC spheroids in collagen (*T* = 0) for up to 72 hours (9). The radius of sprouts and migrating cells was quantitated. The experiment was repeated twice independently, and a total of 20 spheroids were quantitated per group. Data were analyzed by 1-way ANOVA followed by Tukey’s HSD post hoc analysis and are presented as mean ± SEM. *P < 0.05, **P < 0.01, ***P < 0.001.

To elucidate potential cell-based mechanisms by which deregulation of mTOR signaling in MVPCs drives microvessel rarefaction and loss of tissue structure, we performed lineage tracing and isolation of eGFP-labeled MVPCs (Figure 3A). Lineage tracing was analyzed at 10 weeks after tamoxifen induction, and decreased numbers of MVPCs in the Tsc2KD mice correlated to loss of alveolar complexity (Figure 4, A and B, and Supplemental Figure 5D). eGFP-positive MVPC lines were isolated by flow sorting (Figure 4C) from age- and sex-matched mice 2 days after tamoxifen induction. After expansion, primary MVPC functional progenitor and angiogenic properties as well as transcriptomic signatures were analyzed. Isolated MVPC lines were validated by evaluation of clonal expansion using CFU-F analysis, which identified a functional decrease in clonal potential/stemness following Tsc2KD as indicated by the decreased number of CFU (Figure 4D). Western blot analysis defined differences in mTOR activity (Figure 4E), while decreased Tsc2 expression was validated via bulk RNA-Seq (Table 1, boldface text). Levels of p-S6 were increased while total S6 levels were unchanged, the ratio indicative of mTORC1 activation in the Tsc2KD MVPCs (Figure 4E). Interestingly, we found that one Tsc2KD primary line appeared to have activated mTOR while another Tsc2KD line appeared to have regulated mTOR activity based on the Western blot analysis of p-S6 (Supplemental Figure 6B). No significant difference in proliferation was noted between the lines up to 96 hours (Supplemental Figure 6A). The angiogenic sprouting ability of WT and Tsc2KD mTOR+ was tested in a spheroid assay, which showed that mTOR+ Tsc2KD had decreased angiogenic sprouting and individual cell migration up to 72 hours (Figure 4F). The addition of WT lung MVECs did not affect the sprouting deficit of mTOR+ Tsc2KD MVPCs (Supplemental Figure 6C). These data further support findings in our human models and directly demonstrate that activation of mTOR signaling in MVPCs negatively impacts microvascular homeostasis, tissue structure, and progenitor function.

### Mechanistic consequences of Tsc2KD and mTOR dysregulation in MVPCs are linked to p53 and Wnt pathways.

**Figure 5:**
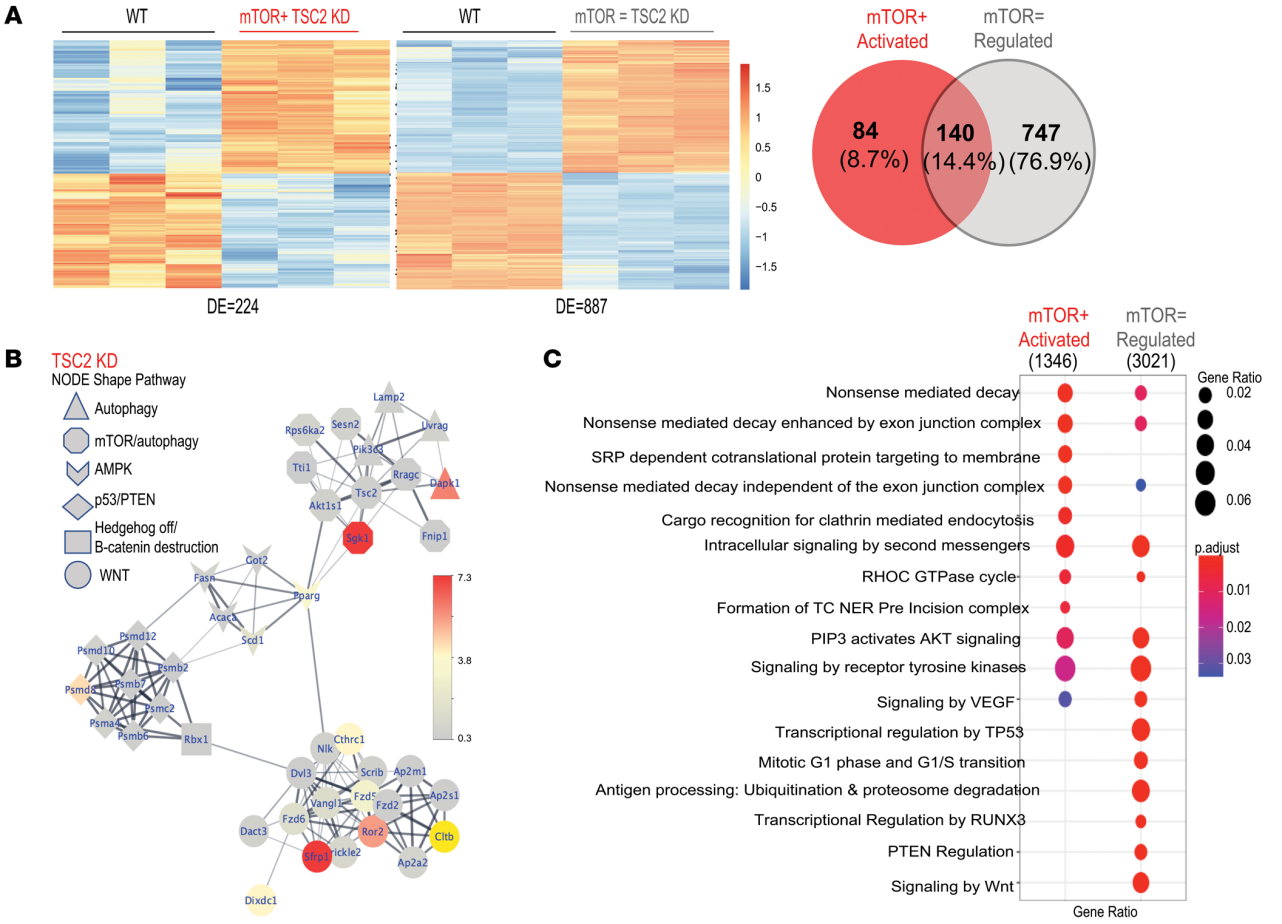
Transcriptomic comparison of MVPCs isolated from WT, mTOR-activated, and mTOR-regulated cell lines identifies links between mTOR and p53 signaling pathways. CD45^–^GFP^+^ MVPCs were sorted from WT or Tsc2KD mouse lungs and cultured to establish primary cell lines from matched WT (2 cell lines), mTOR regulated (1 cell line), and mTOR+ (1 cell line). Each independent cell line was analyzed in triplicate using bulk RNA-Seq. (**A**) Heatmap comparisons of DEGs with an average normalized expression above 2 and a log_2_ fold change greater than 1 in either direction are represented. There are 224 significantly differentially expressed genes (KD vs. WT) in the mTOR+ comparison and 887 significantly differentially expressed genes (KD vs. WT) in the Tsc2-regulated comparison, log fold-change (lfc) ≥ 1. A Venn diagram of DEGs from mTOR-activated (mTOR+) MVPCs relative to mTOR-regulated (mTOR=) MVPCs is shown. (**B**) Functional interaction network of mTOR-regulated DEGs. The STRING database of protein-protein interactions was used to derive the interactions. The shapes of nodes in the network correspond to functions of pathways, while the colors are scaled to biological significance score (61) of the respective gene (log_2_ fold change × –log_10_ adjusted *P* value). Genes identified as unique regulators are gray. mTOR, gray; p53, red; Tsc2, yellow. All interactions shown here have STRING scores of 0.4 or above, representing medium confidence or higher in the evidence of interaction. (**C**) Dot plot of Reactome functional categories that were significantly enriched in the mTOR-activated DEGs list versus regulated MVPCs generated using the CompareCluster function from the Bioconductor package ClusterProfiler (102). The size of the dots corresponds to the gene ratio, the number of genes in the list annotated to the given Reactome category divided by the total number of DEGs with unique Entrez identifiers in the list. The color scale represents the adjusted *P* values obtained for enrichment of the category in each gene list.

An unbiased transcriptomic comparison was used to define mechanisms underlying the decreased stemness and angiogenic ability in mTOR+ Tsc2KD versus mTOR-regulated versus WT MVPCs. Additionally, we identified a Tsc2KD line that exhibited p-S6 expression similar to WT levels (Supplemental Figure 6B), suggesting cell-intrinsic adaptive regulation of mTOR. Bulk RNA-Seq was performed in triplicate using primary WT, Tsc2KD mTOR-regulated, and mTOR+ Tsc2KD lines (Figure 5). After normalization, hierarchical clustering analyses were presented as a heatmap (Figure 5A) depicting significant differences in gene expression between WT and Tsc2KD lines. Tsc2KD was confirmed in bulk RNA-Seq analysis of the MVPC lines (Table 1). Using Reactome, KEGG, and Gene Ontology pathway analyses, we identified p53-dependent mechanisms of TSC2/mTOR regulation differentially controlled between mTOR-activated and mTOR-regulated cell lines (Figure 5, B and C, Table 1, and Supplemental Figure 7). Genes identified as unique regulators were color-coded to match gene lists annotated in Table 1 and Reactome pathways of interest in Table 2. STRING analysis identified major pathways mTOR and autophagy; p53/PTEN proteosome; Wnt signaling; and AMPK/PPAR signaling (Supplemental Table 4). Gene set testing using the mROAST function of the limma package indicated that mTORC1-mediated signaling is upregulated in the mTOR-activated MVPCs and remains unchanged in the mTOR-regulated MVPCs. p53 stability, transcriptional regulation by p53, and Wnt signaling are downregulated in mTOR-regulated mice, and this downregulation may be altered in mTOR+ Tsc2KD MVPCs. Thus, the statistical tests we conducted on our bulk RNA-Seq data suggest that with the increase in mTORC1-mediated signaling upon mTOR activation, the downregulatory control on other developmental pathways like p53 and Wnt may be lessened. The effect of perturbed developmental pathways on cellular processes is evidenced by a clear enrichment in autophagic and apoptotic/senescent pathways in single-cell data of our mTOR+ Tsc2KD MVPCs as compared with controls (Supplemental Figure 8). These data further support our human models and directly demonstrate that mTORC1 functions as a signaling hub, suggesting that the MVPC phenotype resulting from aberrant activation of mTOR is dependent on additional signaling pathways such as Wnt, AMP kinase, and p53.

**Figure 6:**
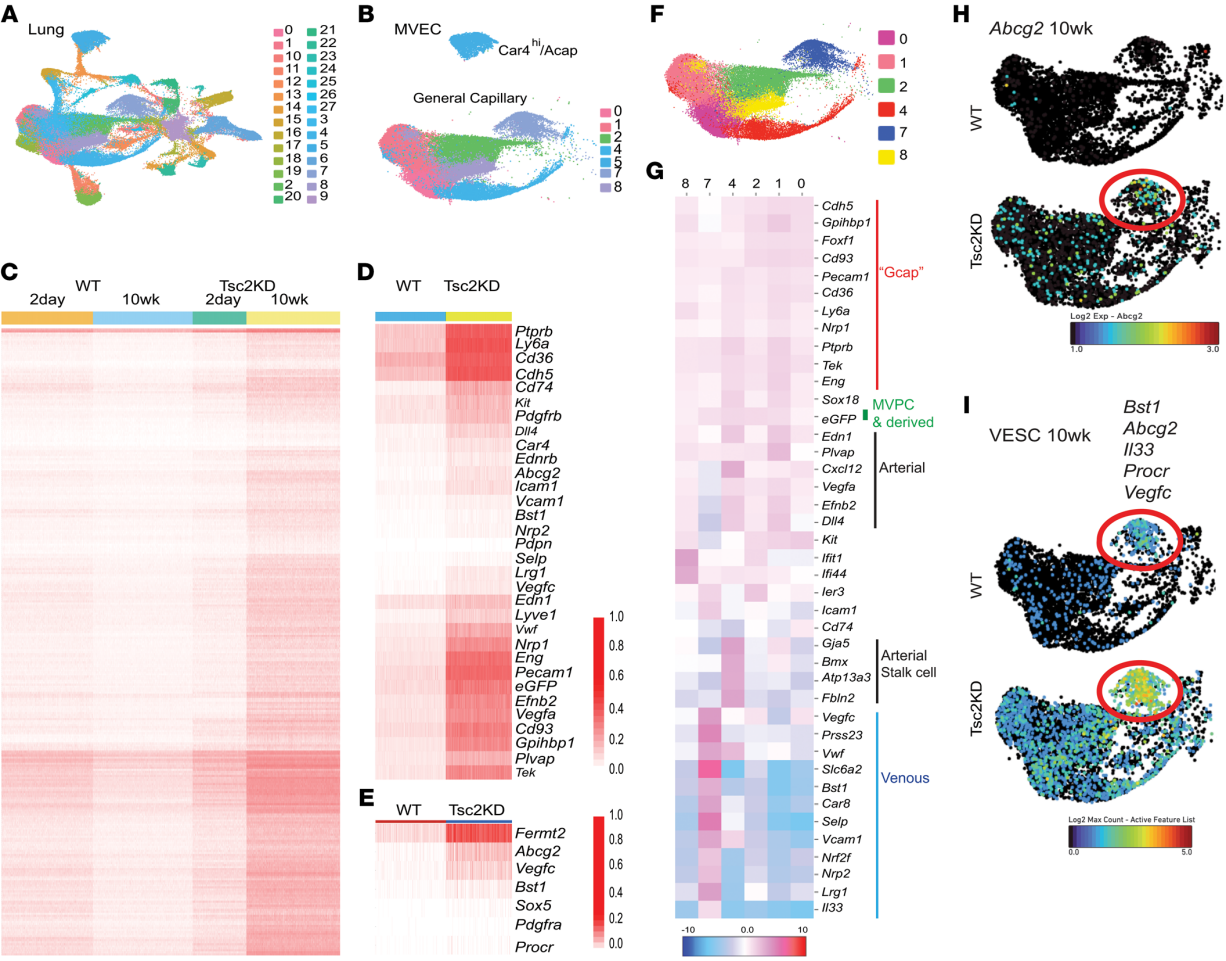
Angiodiversity in the lung microvascular capillary bed, localization of MVPCs, and impact of Tsc2KD in MVPCs at the single-cell level. Adult WT or Tsc2KD mice were induced with tamoxifen; 2 days or 10 weeks later, lungs were collected and digested to form single-cell suspensions, and samples were pooled and sequenced (WT: day 2, *n* = 5; 10 weeks, *n* = 4; Tsc2KD: day 2, *n* = 4; 10 weeks, *n* = 4). (**A** and **B**) Uniform manifold approximation and projection (UMAP) with Seurat clustering of single-cell lung suspensions in Loupe Browser 5.0 (10x Genomics) was annotated to define lung MVEC clusters as general capillary (Gcap) or Car4^hi^/aerocytes (Acap). Common cluster designations (0 to 27) are presented. (**C**) Differential expression of Tsc2KD versus WT MVEC clusters at 2 days and 10 weeks. (**D** and **E**) DEGs detected in a comparison of Tsc2KD versus WT at 10 weeks using Seurat FindMarkers were used with a cutoff of adjusted *P* value less than 0.05 and log fold change of at least 1. A custom list of genes important for vascular function was selected from the DEG list. Heatmap was generated using the dittoHeatmap function from the dittoSeq package (Bioconductor). Expression of vascular (**D**) and mesenchymal stem cell–related (**E**) markers in WT and TSC2KD at 10 weeks. (**F** and **G**) Loupe Browser v5 was used to define the heterogeneity in the general capillary bed via dot plot and heatmap of known microvascular endothelial genes. (**H** and **I**) *Abcg2* expression (**H**) or coexpression (**I**) of vascular endothelial stem cell (VESC) markers at 10 weeks in WT or Tsc2KD dot plot. Five to eight mice per group were pooled for these experiments.

To evaluate the impact of mTOR activation by Tsc2KD in MVPCs on the microvascular endothelial cell (MVEC) population, we used single-cell transcriptomic analysis of fractionated lung tissue. Mouse lung samples were pooled, and cell sorting was performed to collect WT and mTOR+ Tsc2KD CD45^–^GFP^+^ and CD45^–^GFP^–^ samples from 2 days and 10 weeks after tamoxifen induction. Following annotation of the original 28 lung subclusters (Figure 6A), capillary MVECs were localized to 7 subclusters (Figure 6B). Cluster 5 was identified as Car4^hi^/alveolar endothelial cells (Acap, Cap2), which localized as distinct from the bulk population of microvascular endothelium (MVECs), previously annotated as general capillary (Gcap or Cap1) (54, 55). For differentially expressed or select genes, the SCTransform-normalized values for the samples from the Seurat object were plotted via heatmap using dittoSeq. Temporal comparison of the MVEC populations demonstrated that in WT the population was transcriptionally stable from 2 days to 10 weeks after induction, as would be expected during angiostasis (Figure 6C). In contrast, lung MVECs from the mice with mTOR+ Tsc2KD MVPCs demonstrated significant increases in gene expression at 10 weeks relative to day 2 after induction (adjusted *P* value < 0.05 and absolute log fold change > 1; 443 genes; Figure 6C and Supplemental Table 5). Increased gene expression was related to mesenchymal stem cell differentiation, microvascular endothelial differentiation, mTOR activation, autophagy, and apoptosis/senescence in the absence of significant proliferation or change in percentage cell distribution per cluster (Figure 6, D and E, Supplemental Table 6, and Supplemental Figure 8, A and B). These detailed complementary analyses identify mechanistic consequences of Tsc2KD and mTOR dysregulation in MVPCs and highlight roles for p53 signaling in the regulation of mTOR and deregulated Wnt signaling as potential drivers of MVPC loss of function and microvascular niche homeostasis underlying loss of distal lung tissue structure and function.

**Figure 7:**
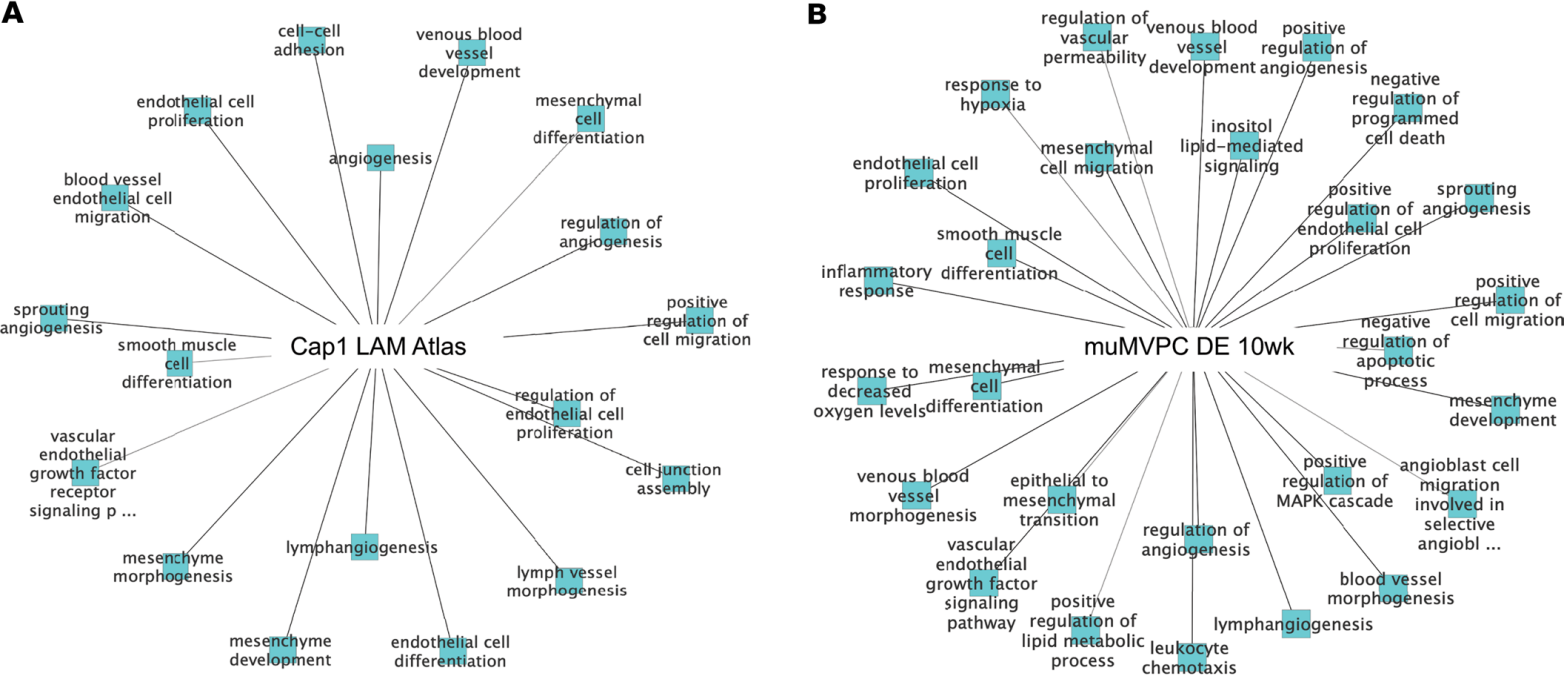
Significant overlap in functional motifs of signature genes characteristic of human mTOR+ capillary microvascular endothelium and equivalent cells in Tsc2KD mice. (**A**) One hundred signature genes of human Cap1 cells were downloaded from the LAM Cell Atlas (https://research.cchmc.org/pbge/lunggens/LCA/LCA.html) and used as input for ToppCluster to explore Gene Ontology biological process (GOBP) annotations. (**B**) Mesenchymal stem cell marker genes differentially expressed between WT and Tsc2KD at 10 weeks after tamoxifen induction in scRNA-Seq analysis were used as input for ToppCluster to explore GOBP annotations.

Angiodiversity in the capillary microvascular endothelium was evident in scSEQ Seurat clustering (Figure 6, A, B, and F). The MVECs were clustered into groups like those identified in lung tumorigenesis (56, 57). While the capillary MVECs express what has been labeled a general capillary transcriptome, specialized arterial and venous signatures also coexist as have been described in numerous other tissue-specific capillary beds (Figure 6G). At angiostasis, *Abcg2* expression was low in the WT capillary microvasculature; however, with Tsc2KD it was increased significantly, most notably in cluster 7 or the venous cluster (Figure 6, E and H; cluster 7, red circle). Ten weeks after tamoxifen induction, the frequency of cells expressing *Abcg2* transcript in venous cluster 7 was 0 in WT and 70 in Tsc2KD. High levels of *Abcg2* expression are an enrichment tool for progenitors. However, analysis of *Abcg2* coexpression with additional vascular endothelial progenitor markers, *Bst1*/*CD157*, *IL33*, *Procr*, and *Vegfc*, further defined the MVPC progenitors to the venous cluster 7 (Figure 6I). These findings were further confirmed by functional motifs generated using single-cell RNA-Seq (scRNA-Seq) differential gene expression analysis of the human LAM MAP Cap1 population of normal versus mTORC+ genes (100 total genes) compared with the mouse WT and Tsc2KD at 10 weeks (all genes in analysis; Figure 7, A and B). Functional motif signatures of genes demonstrated significant commonalities in Gene Ontology biological processes. The significant common pathways included venous blood vessel development/morphogenesis, regulation of angiogenesis, positive regulation of cell migration, endothelial cell proliferation, endothelial cell differentiation, lymphangiogenesis, mesenchymal cell morphogenesis/development/differentiation, angiogenesis/vessel morphogenesis, VEGF signaling, and smooth muscle cell differentiation. Taken together, these data demonstrate angiodiversity in the lung capillary microvascular network and that the phenotype and function of a venous adult lung angiogenic CD45^–^Abcg2^+^ MVPC subpopulation are regulated by mTORC1 signaling.

## Discussion

Reactivation and dysregulation of the developmental signaling pathway mTOR are a hallmark of aging and chronic lung diseases (13–21). In the present study we investigated the consequence of mTORC1 signaling activation, with loss of Tsc2 function, in the regulation of Abcg2^+^ microvascular progenitor cell (MVPC) function and the integrity of alveolar-capillary structure. Using complementary translational models, our data support a significant role for mTOR in the maintenance of human and mouse MVPC progenitor function via crosstalk with multiple signaling cascades as well as a cell-based mechanism underlying lung tissue remodeling and the development of emphysema. Furthermore, using single-cell transcriptomic analysis, we localized the Abcg2^+^ MVPCs within the angiodiverse human and mouse capillary microvascular clusters and demonstrated how mTOR activation enhances the appearance of MVPCs within the capillary-venous subcluster. Thus, our studies are, to our knowledge, the first to demonstrate the venous localization of an adult lung microvascular progenitor and link transcriptomic-functional-structural relationships in an adult lung microvascular progenitor population.

Here we show that a collection of SNPs in the *TSC2* gene may contribute to alteration of function that results in an inability of the protein to suppress mTOR, leading to signaling activation (44). These results are supported by previous studies describing haploinsufficient tumors in both humans and mice rather than loss of heterozygosity (29, 44, 45). TSC2 expression was present in the human mTOR-activated MVPCs, and these cells had a non-cell-autonomous effect on the recipient lung tissue structure. The recipients of mTOR-activated lung-derived MVPCs demonstrated airspace enlargement and vascular lesions in the absence of significant ectopic proliferation by the donor cells. The airspace enlargement was replicated in our complementary mouse model using an inducible knockdown (KD) of *Tsc2* (53) in adult lung MVPCs. Tsc2KD in adult mice resulted in the development of an emphysematous phenotype and function, decreased microvessel density, and decreased vascular progenitor self-renewal and angiogenic sprouting. Together these data also suggest that loss of MVPC-regulated microvascular stability and structure has a negative impact on lung alveolar epithelial progenitor numbers and function, either directly or indirectly (37, 46).

Depletion of Tsc1 or Tsc2 in adult lung epithelium, smooth muscle, or vascular endothelium demonstrated that the activation of mTORC1 drove cell senescence, loss of lung alveolar structure, and the appearance of emphysema with pulmonary hypertension and significant vascular remodeling in male mice (13). Developmental Tsc2 knockout using Tbx4 targeted all developing lung mesenchyme including endothelial, fibroblast, and smooth muscle lineages (58), resulting in a simplified alveolar structure (15, 59). We previously observed a similar loss of structure and tissue remodeling driven by Wnt activation in MVPCs in an adult model of microvascular injury or genetic depletion of MVPCs with loss of adaptive angiogenesis (9, 10). In contrast, mTOR activation in differentiated endothelium induces proliferation and stimulates neointimal hypertrophy and inflammatory signaling (60). Taken together these data allude to the substantial impact of deregulated mTOR in the microvascular niche underlying both vascular and epithelial tissue remodeling in adult CLDs. This concept is strongly supported by transcriptomics of mTOR-driven tumors influencing endothelial cell function and tumor angiogenesis (61). Given the pivotal inductive role of the lung mesenchyme in lung tissue development influencing both the epithelium and vasculature as well as COPD/emphysema, these findings illustrate the importance of mTOR signaling in these processes (62–64).

The strength of our model is that we use Abcg2 to identify MVPCs (9, 10, 12, 38–40, 65). This methodology has facilitated the isolation and confirmation of enriched angiogenic progenitor activity by both mouse and human MVPCs in a variety of model systems combining histological and physiological endpoints. Loss of progenitor clonal expansion and repair of endothelial barrier suggested that mTOR activation impaired the homeostatic function of these cells and the adjacent endothelium in the absence of significant changes in proliferative rate. These data were further supported by lack of difference in protein expression between WT and mTOR+ human MVPC levels of p53, MDM2, p16, and p21. In contrast, the increase in mTORC2 activity (p-AKT^Ser473^) was detected in the MVPCs with the highest level of mTORC1 activity (66, 67). The activation of mTORC2 may also explain in part the limitations of rapamycin and rapalogs to inhibit all mTORC1 processes and eliminate the causative cells underlying disease processes. Balanced mTORC1/2 signaling is responsible in part for maintenance and proliferation of progenitor cells (21, 68), whereas aberrant activation may prevent progenitor self-renewal and promote progenitor differentiation or senescence in a cell- or context-specific manner (19, 21, 68).

mTOR activation in both mouse and human MVPCs facilitated the identification of linked signaling pathways of importance underlying the loss of tissue structure as well as common Gene Ontology biological processes related to angiostasis, angiogenesis, and remodeling regulated by mTOR signaling. The complex relationship between TSC2/mTORC1 and p53 has previously been defined in human stem cell reprogramming (69) and zebrafish (70) as well as homeostasis, stress, cancer, metabolism, aging, and senescence (22, 71–76). The influence of both p53 and AKT/mTOR on the Wnt pathway at the intersection of metabolism and injury was necessary for repair, and optimal repair was dependent on the coactivation of multiple pathways (50, 77–81). Notch signaling is also critical for the suppression of TSC2 in progenitor populations to drive lineage commitment and differentiation (21), and its intersection with Wnt drives arterial specification in the developing vasculature (82). Taken together these studies illustrate the complexity of crosstalk between signaling pathways regulating vascular progenitor function and lung tissue structure with deregulated mTORC1 activity, while the role of mTORC2 is less clear (83, 84).

To further define a mechanistic basis for deviation in MVPC function and differentiation, we used transcriptomics to examine both human and mouse mTOR-activated cells relative to control. We demonstrate that the WT microvascular capillary endothelial transcriptome was stable by comparing 2 days versus 10 weeks after induction, which is expected for a progenitor population during adult tissue homeostasis. In contrast, we found that mTOR activation in mouse MVPCs drove transcriptional reprogramming resulting in increased expression of progenitor markers as well as differentiated vascular capillary, venous, and inflammatory endothelial markers. These data support a mechanism for inhibition of mTOR in the endothelium, which improves autophagy, vascular integrity, attenuation of apoptosis, and inflammation (85–87). The intersection of these pathways represents control of progenitor function as well as energy sensing and metabolism (88).

In the current studies, we identify the capillary-venous location of these Abcg2 MVPCs in mouse single-cell data sets and highlight the heterogeneity within the microvasculature of the lung. Our functional as well as transcriptomic data demonstrate the difficulty of pinpointing the adult lung Abcg2 MVPCs during tissue homeostasis. However, when mTOR is activated, an increase in progenitor markers highlights the microvascular progenitor pool within the venous subpopulation. Developmental studies have also defined a venous origin for capillary sprouting in the brain microvasculature (89, 90). The evolution of transcriptomics to single-cell RNA-Seq has begun to define heterogeneity and function of blood microvascular endothelial cells (ECs) in a variety of adult organ systems, including lung tumors, lymph nodes, kidneys, liver, brain, and heart (40, 54, 55, 91–97). Additionally, the balance of mTOR with additional developmental signaling cascades, including p53, Wnt, and Notch, can maintain adult stem cell niches and delay progenitor exhaustion via regulation of quiescence and the cell cycle (68, 71).

Considerable progress has been made in understanding the mechanism by which decreased TSC2 function increases mTORC1 signaling in specific cell subsets, including MVPCs. While mTORC1 inhibitors can slow the progression of pulmonary remodeling, they do not eliminate or “correct” the underlying cells that contribute to the pathogenesis, nor do they affect mTORC2 (98). The pivotal identification of the venous location of the subset of MVPCs facilitates our ability to harness resident cells for tissue repair as well as to provide an understanding of disease pathogenesis. Together these studies highlight the need for a more detailed understanding of TSC2/mTOR-dependent mesenchymal, vascular, and epithelial cell-cell interactions and their impact on linked regulatory signaling pathways to facilitate the development of new interventional combination therapies targeted toward restoration of lung structure and function.

## Methods

### Isolation and characterization of murine and human primary lung MVPCs.

Mouse MVPCs were isolated from the lineage tracing murine strains based on eGFP expression by flow cytometry (Figure 4C). Flow sorting coupled to antibody labeling of Abcg2^+^ was used to isolate human lung MVPCs from tissue explants of adult controls (*n* = 4, male and female), COPD patients (*n* = 2, female), and 1 patient diagnosed with LAM (3 independent cell lines, female) or fetal lung fibroblast cultures (*n* = 3, male and female). Three independent cell lines were generated from 3 different lobes of the LAM lung explant, including very affected and cystic, less affected, and cystic and unaffected. Techniques were previously described, and ABCG2 was validated as a cell surface marker for both murine eGFP-labeled and human MVPCs (Supplemental Figure 1, A and B) (10, 38–40).

### Transgenic depletion of Tsc2.

ABCG2-Cre^ERT2^ mice, a gift from B. Sorrentino (St. Jude Children’s Research Hospital, Memphis, Tennessee, USA) (52), were crossed to a fluorescent eGFP reporter (Cg)-Gt(ROSA)26Sortm4(ACTB-tdTomato-EGFP) strain (The Jackson Laboratory, stock 007676; designated mT/mG) to facilitate lineage analysis and quantitation via eGFP expression. A third gene, a *Tsc2* conditional allele, was crossed into the mice to deplete *Tsc2* (53), designated Tsc2 knockdown (Tsc2KD) (9, 10, 40). Recombination of the *Tsc2* allele in the lung was confirmed via Transnetyx genomic analysis of lung tissue. The mice were randomized and distributed with 3–5 mice per cage for studies (both male and female). Micro-CT was performed 2 days before tamoxifen induction and confirmed the absence of baseline differences in tissue structure between the strains (not shown; *n* = 6 male and 5 female WT, 6 male and 6 female Tsc2KD). Endpoint analyses were conducted between 9 and 12 weeks (after tamoxifen induction).

### Characterization of physiological and structural alterations in the lung as a result of Tsc2KD in MVPCs.

Measurements of lung leak, right ventricular systolic pressure, and flexiVent (SCIREQ) physiology were performed as previously described (8, 99). Detailed methods for the former as well as micro-CT imaging, histological processes, and quantitation of lung structure and protein expression may be found in Supplemental Methods. All antibodies are referenced in Supplemental Table 7.

### Ex vivo analyses of MVPC function.

Murine and human MVPC isolation and characterization were performed as previously described (see reagent list, Supplemental Table 7) (8–10, 38, 40). Detailed methods are presented in Supplemental Methods. The spheroid sprouting assay and quantitation were performed as we previously described and were analyzed using Wimasis WimSprout image analysis software (9). Cell proliferation was analyzed using CellTiter 96AQ_ueous_One Solution (Promega) following the manufacturer’s protocol. Electric Cell-Substrate Impedance Sensing (ECIS; Applied Biophysics) was performed to examine migration and repair of barrier function following a “wounding” stimulus in cocultures of normal or mTOR+ MVPCs and human lung microvascular endothelium (9). These experiments were performed with 2 sample replicates and repeated twice.

### Genomic analyses of MVPCs.

Detailed methods are presented in Supplemental Methods. scRNA-Seq was performed using the Chromium Single Cell 3′ Library and Gel Bead Kit (v3.1, 10x Genomics), and the Chromium X and libraries were sequenced on an Illumina NovaSeq 6000. Sequencing guidelines for scRNA-Seq included producing at least 50,000 sequencing reads per cell to ensure adequate depth of coverage for comparative analysis as we previously reported (9). Data were collected on 95,037 cells from 12 samples (WT and Tsc2KD 2 days after tamoxifen induction: CD45^–^GFP^+^ [5,885 cells WT, 3,456 cells KD], CD45^–^, CD45^+^; WT and Tsc2KD 10 weeks after tamoxifen induction: CD45^–^GFP^+^ [6,406 cells WT, 6,019 cells KD], CD45^–^, CD45^+^). All post-quantification quality control, normalization, integration, clustering, and marker finding were performed in Seurat (v3.1; Satija laboratory, ref. 100). Differentially expressed genes were defined as having a Bonferroni-adjusted *P* value less than 0.05, a log FC greater than 0.1, and more than 5% of cells with detectable expression. Microarray analysis of human lung MVPCs was performed as previously described (Gene Expression Omnibus GSE225760, NCBI 23718621) (9, 10, 39, 101), in triplicate or with an *n* of 3 or more independent patient samples. Representative genes were selected for target validation (see reagent list, Supplemental Table 7).

### Mouse human MVPC chimeras.

NSG mice were obtained from The Jackson Laboratory (stock 005557). Primary human lung MVPC lines (LAM/mTOR+, LAM/unaffected, control MVPC) were expanded and resuspended in HBSS (Thermo Fisher Scientific) and filtered through a 100 μm cell strainer (Thermo Fisher Scientific). Five hundred thousand total female cells in 100 μL were injected i.v. via the tail vein of recipient female NSG mice at 14 weeks of age. Mice received injections on day 0, and micro-CT scans were performed prior to harvest. Endpoint analyses were conducted at 2 and 6 months.

### Statistics.

Data were analyzed by 1-way ANOVA followed by Tukey-Kramer honestly significant difference (HSD) post hoc analysis. Murine quantitative PCR and patient samples were analyzed using nonparametric Wilcoxon/Kruskal-Wallis test and a χ^2^ approximation. All analyses used JMP version 16 software. Data are presented as mean ± SEM. Significance was defined as *P* value less than 0.05.

### Study approval.

The Institutional Animal Care and Use Committee at National Jewish Health and Vanderbilt University Medical Center approved all animal procedures and protocols. These studies used banked patient cell lines obtained using IRB 9401 approved by the Vanderbilt University Medical Center IRB Committee, Nashville, Tennessee, USA. Patients gave consent under this IRB for the generation and storage of human cell lines as previously described (10, 39).

### Data availability.

The data sets generated and analyzed during the current study are available in Gene Expression Omnibus (GEO)/NCBI (GSE225656, NCBI 23699747; GSE225760, NCBI 23718621; GSE242065, NCBI 24235838) or by request from the corresponding author. All supporting data are provided in the Supporting Data Values file or will be provided by the corresponding author upon request.

## Author contributions

ECM was responsible for investigation, methodology, software, validation, data curation, formal analysis, and writing — original draft preparation and review and editing. SM was responsible for conceptualization, methodology, bioinformatics analysis, software, validation, formal analysis, data curation, and writing — original draft preparation and review and editing. BRS and MMD were responsible for investigation, methodology, formal analysis, and writing — review and editing. CFG was responsible for investigation, methodology, and formal analysis. CMM was responsible for methodology, software, validation, formal analysis, data curation, and writing — original draft preparation and review and editing. LCA was responsible for investigation and methodology. OC was responsible for investigation, methodology, formal analysis, and writing — review and editing. BWR and JAK were responsible for resources, data curation, and writing — review and editing. JDW was responsible for investigation, resources, data curation, and writing — review and editing. PG and KCE were responsible for conceptualization, methodology, investigation, resources,supervision, and writing — review and editing. BNG was responsible for resources, investigation, and conceptualization. FG was responsible for investigation, methodology, and writing — original draft preparation and review and editing. SMM was responsible for conceptualization, methodology, formal analysis, investigation, resources, data curation, and writing — original draft preparation and review and editing, as well as visualization, supervision, and funding acquisition.

## Supplementary Material

Supplemental data

Supporting data values

## Figures and Tables

**Table 2:**
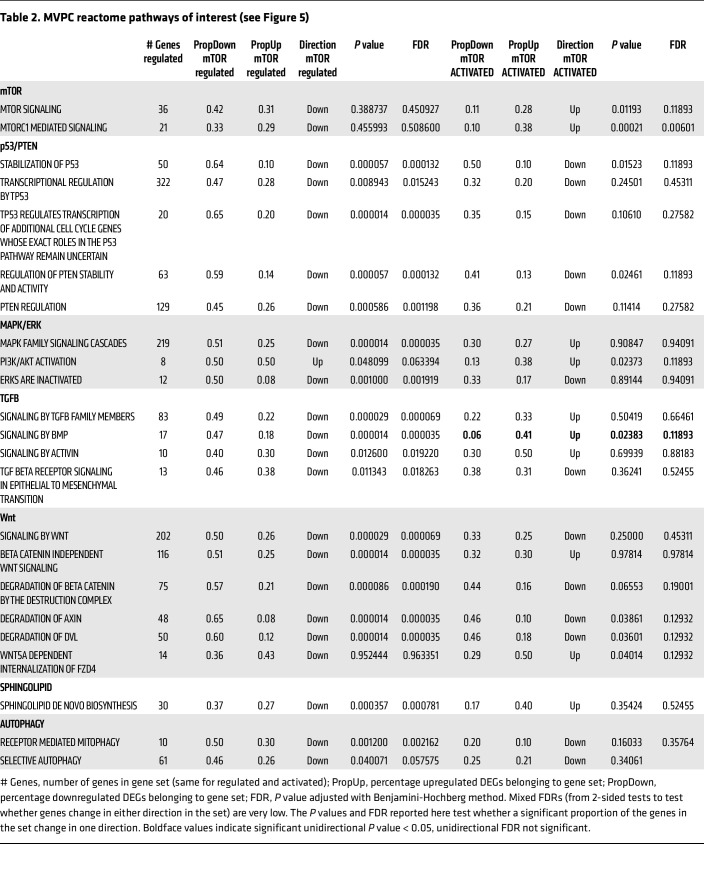
MVPC reactome pathways of interest (see Figure 5)

**Table 1:**
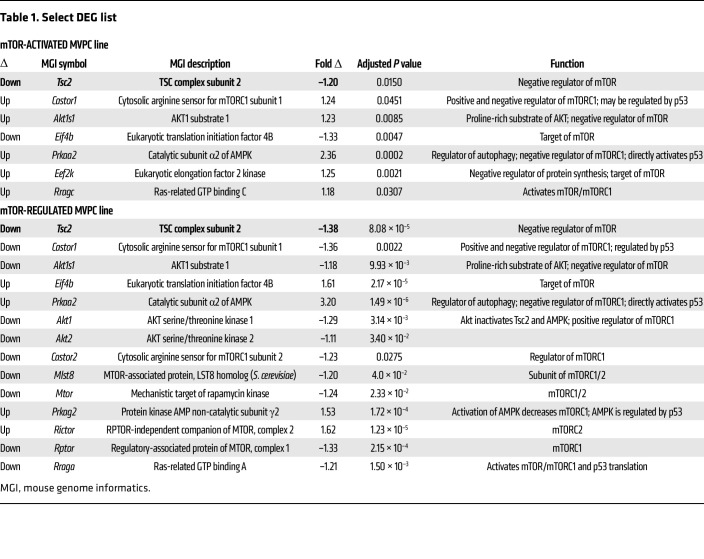
Select DEG list

## Version 1 10/24/2023

In-Press Preview

## Version 2 12/15/2023

Electronic publication
