# Supplementary material for: Activation of mTOR signaling in adult lung microvascular progenitor cells accelerates lung aging
Source: J Clin Invest. 2023 Dec 15;133(24):e171430. doi: 10.1172/JCI171430 (PMC10721153; doi:10.1172/JCI171430)
Supplement: Supplemental data [file jci-133-171430-s092.pdf]

SUPPLEMENTAL DATA

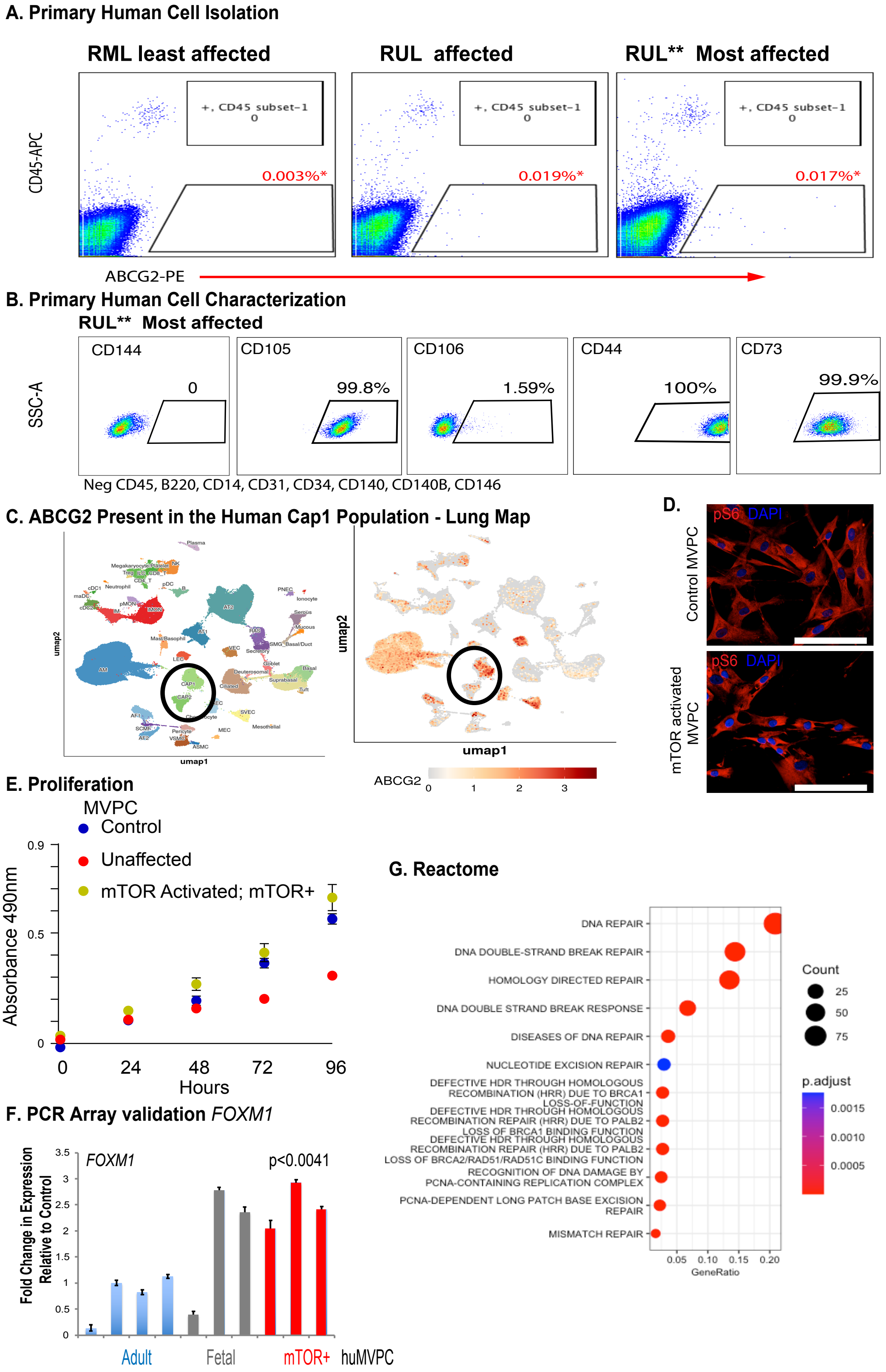

Supplemental Figure 1 (Figures 1&2). Characterization of mTOR activated MVPC. A. Explanted tissue from a LAM patient was digested with TypeII collagenase to form a single cell suspension. The suspension was stained with antibodies to detect CD45 (APC) and ABCG2 (PE) to sort $CD45^{neg}ABCG2^{pos}$ MVPC from three areas of the affected tissue. **B.** The primary cells were expanded and characterized for the expression of MVPC cell surface markers by flow cytometry. **C.** Abcg2 transcript expression was localized to the Cap1 population of human microvasculature using LungMAP (https://lungmap.net). **D.** Primary mTOR activated (LAM) MVPC were characterized for the expression of pS6 levels as an indication of mTOR signaling by immunostaining. **E.** MVPC were plated in a 96 well-plate and proliferation was analyzed using CellTiter 96AQ_{ueous}One Solution (Promega, Madison, WI) at the time points stated. The assay was performed in triplicate with four independent replicates. Data were analyzed by one-way ANOVA followed by Tukey's HSD post-hoc analysis and presented as mean (+/-SEM). **F.** RT-PCR analysis was performed to validate differences in *FOXM1* expression identified by array findings using equal amounts of cDNA from independent MVPC lines to validate the array findings. Normal (3-4 independent patient primary cell lines F,F,M,M age 60-67), mTOR+ (one patient, 3 independent cell lines 63 years of age), and fetal (HFL; 3 independent patient primary cell lines 17-20 weeks of gestation, M,M,F) MVPC. Each patient sample was analyzed in triplicate, standardized to GAPDH and normalized to control presented in lane 1 set to 1. Control = Blue; Fetal = Grey; mTOR+ samples= RED. n= 3-4, 3,3. * represents p-values comparing Adult to mTOR+ MVPC. Each independent primary cell line was analyzed in triplicate and is normalized to control presented in lane 1 set to 1. n=4,3,3. Data were analyzed by nonparametric Wilcoxon/Kruskal Wallis and a chi-square approximation and presented as mean (+/-SEM). **G.** Reactome analyses related to DNA synthesis and repair. Dotplot showing significantly enriched Reactome functional categories and pathways in differentially expressed gene lists mTOR activated versus regulated. The color scale represents the adjusted p-values obtained for the enrichment of the category in each gene list.

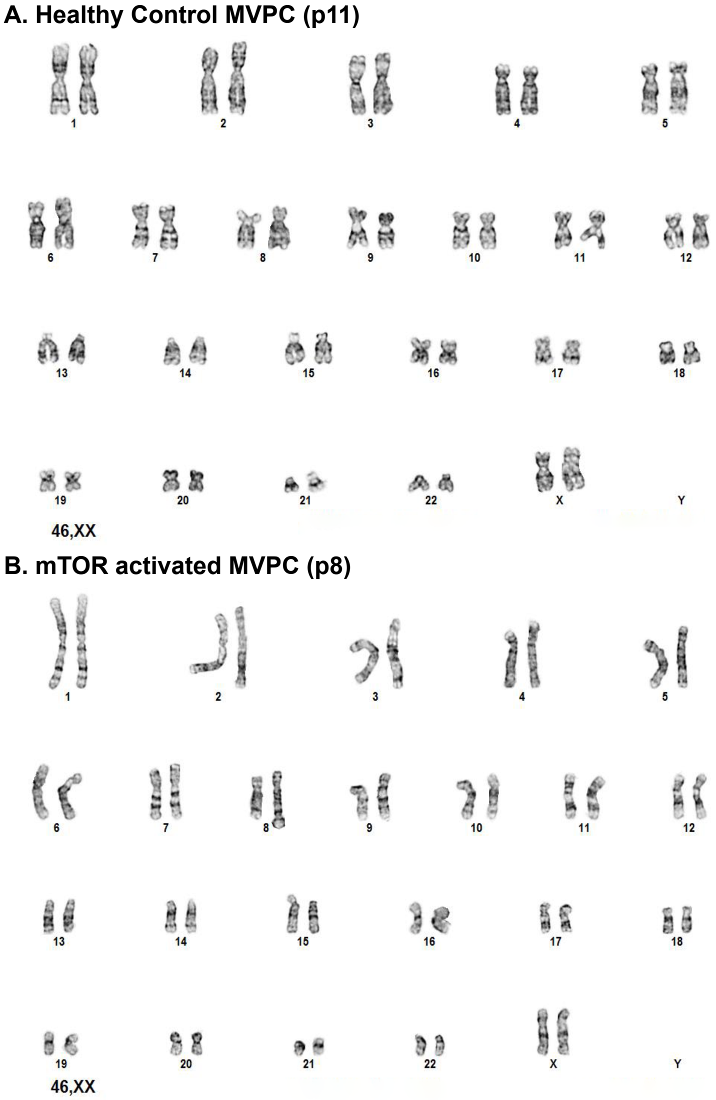

Supplemental Figure 2. Primary human MVPC display a normal karyotype (Figure 1 A-C). Given the effects alterations in mTOR signaling could have on primary cell genomic stability we analyzed representative cell karyotypes to define gross chromosome structure. We performed karyotyping on the most affected mTOR+ primary female 65yr old MVPC line at passage 8 and 19. We also performed karyotyping of an age matched (67yrs of age) human female control line at passage 11 and 19. Representative images are depicted, and karyotypes were characterized as “normal”.

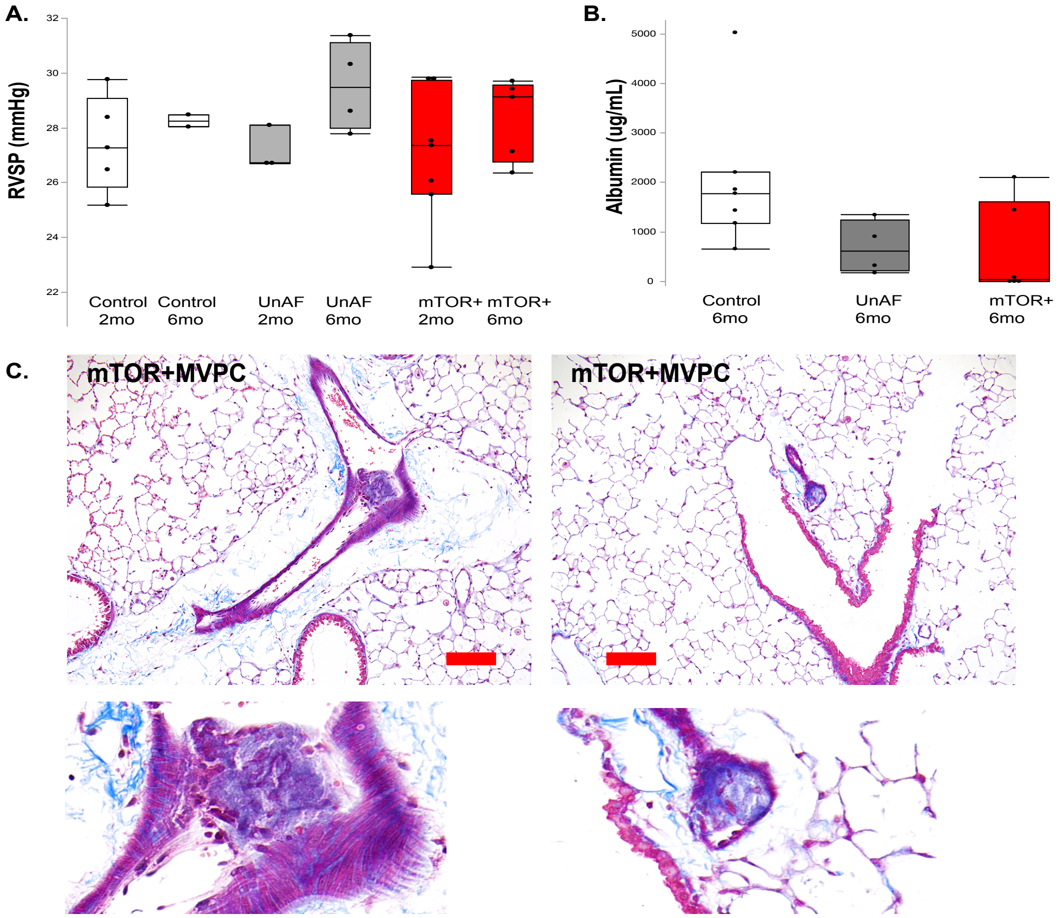

Supplemental Figure 3 (Figure 1G-Q). Adoptive transfer of mTOR activated MVPC does not result in the development of pulmonary hypertension. To create humanized mice, we adoptively transferred 500,000 age matched (60-66 years of age) female human MVPC from healthy control, unaffected (non-cystic lung) or mTOR activated (cystic lung) tissue via the tail vein of recipient female NSG mice (14 weeks of age). n=8 mice per cell line used. Lung function and structure were analyzed at 2 or 6 months. Data were analyzed by one-way ANOVA followed by Tukey's HSD post-hoc analysis and presented as mean (+/-SEM). * $p < 0.05$, ** $p < 0.01$. **A.** Right ventricular systolic pressure (RVSP) was measured post flexiVent analysis by a pressure transducer placed in the right ventricle. **B.** Albumin ELISA was used to measure lung leak at 6 months. **C.** Lung vascular lesions were identified and trichrome stained to detect collagen at 2 months. Enlarged image scale = 100μM.

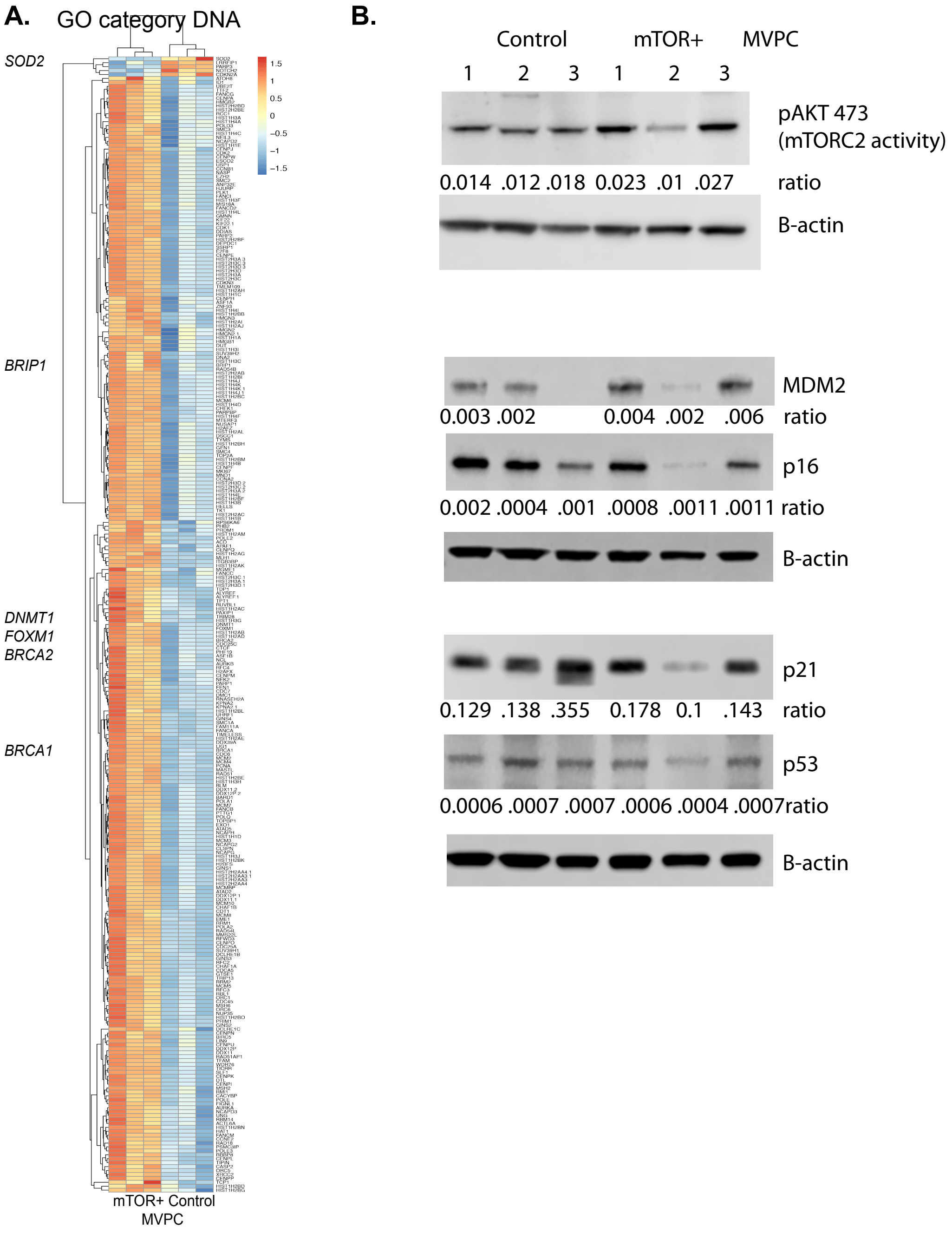

Supplemental Figure 4 (Figure 2 C&D). Differentially expressed genes (DEG) between mTOR activated MVPC and Control. Human lung MVPC were isolated from explanted LAM lung or fetal lung fibroblast cultures. Array analysis was performed, in triplicate or with an n of three or greater independent patient samples. A minimal fold change of 1.7X, up or down, and p<0.05 were employed as criteria for defining differentially expressed genes. Expression values for these genes are represented in a heatmap; generated using the pheatmap function in R.

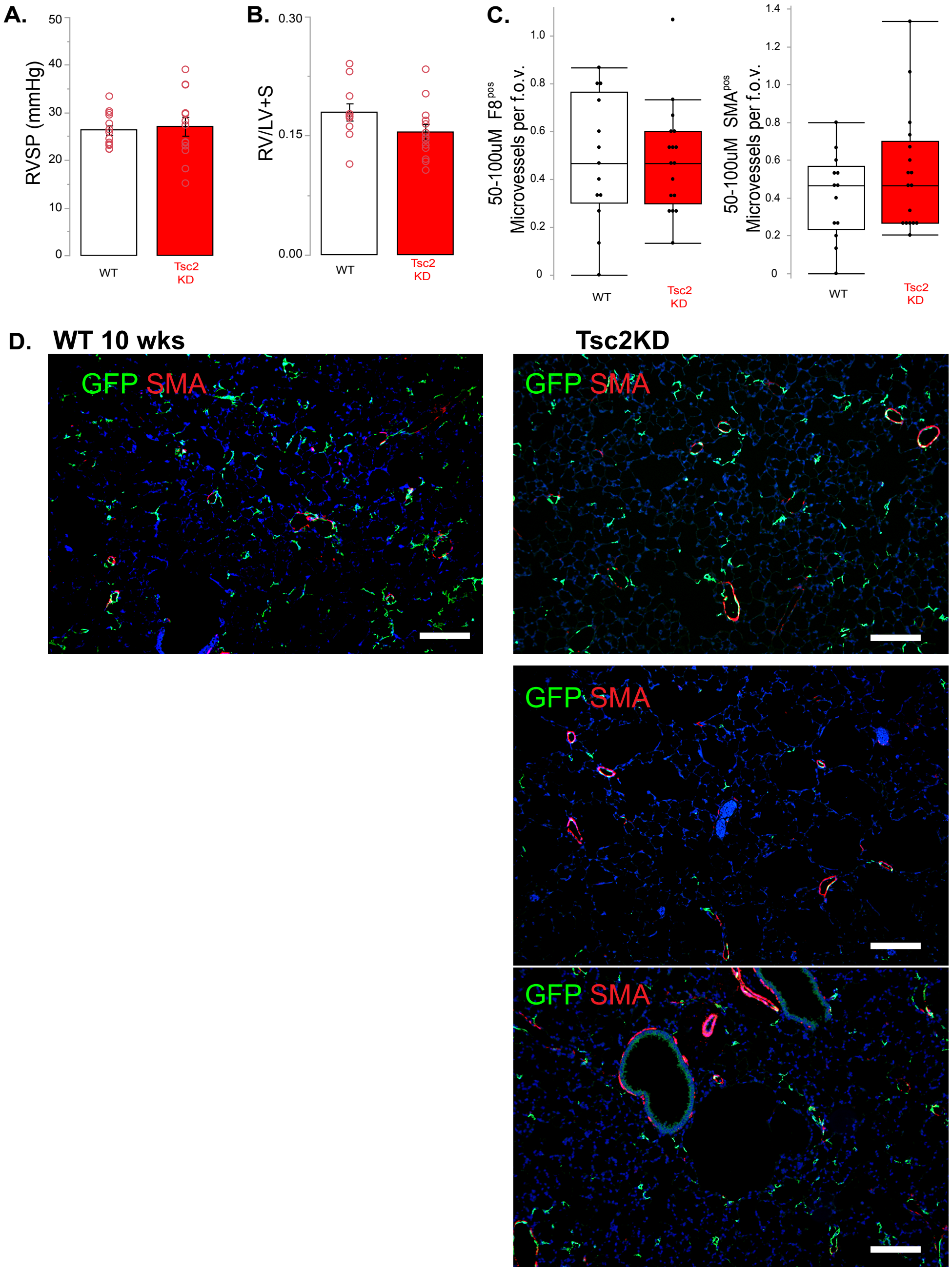

Supplemental Figure 5 (Figures 3&4). Tsc2KD in MVPC does not result in the development of pulmonary hypertension. Adult female and male mice were induced at 12 weeks of age with tamoxifen (0.5mg) and endpoint analysis was conducted between 10-12 weeks of age. Groups: Abcg2CreERT2 ^{fl}*Tsc2* +/+ or -/- (n=10-17). Data were analyzed by one-way ANOVA followed by Tukey's HSD post-hoc analysis and presented as mean (+/-SEM). **A.** Right ventricular systolic pressure (RVSP) was measured post *flexi*Vent analysis by a pressure transducer placed in the right ventricle. **B.** Hearts were collected and weight to quantitate Fulton's index (RV/LV+S). **C&D.** Immunostaining was performed on lung tissue sections to quantitate Factor 8 (F8) and smooth muscle alpha actin (SMA) positive microvessels ranging from 50-100μM in diameter. Data were analyzed by one-way ANOVA followed by Tukey's HSD post-hoc analysis and presented as mean (+/-SEM). **D.** Lineage tracing was performed on tissue sections by immunostaining to detect eGFP (green) and smooth muscle alpha actin (SMA, red). Scale = 100μM.

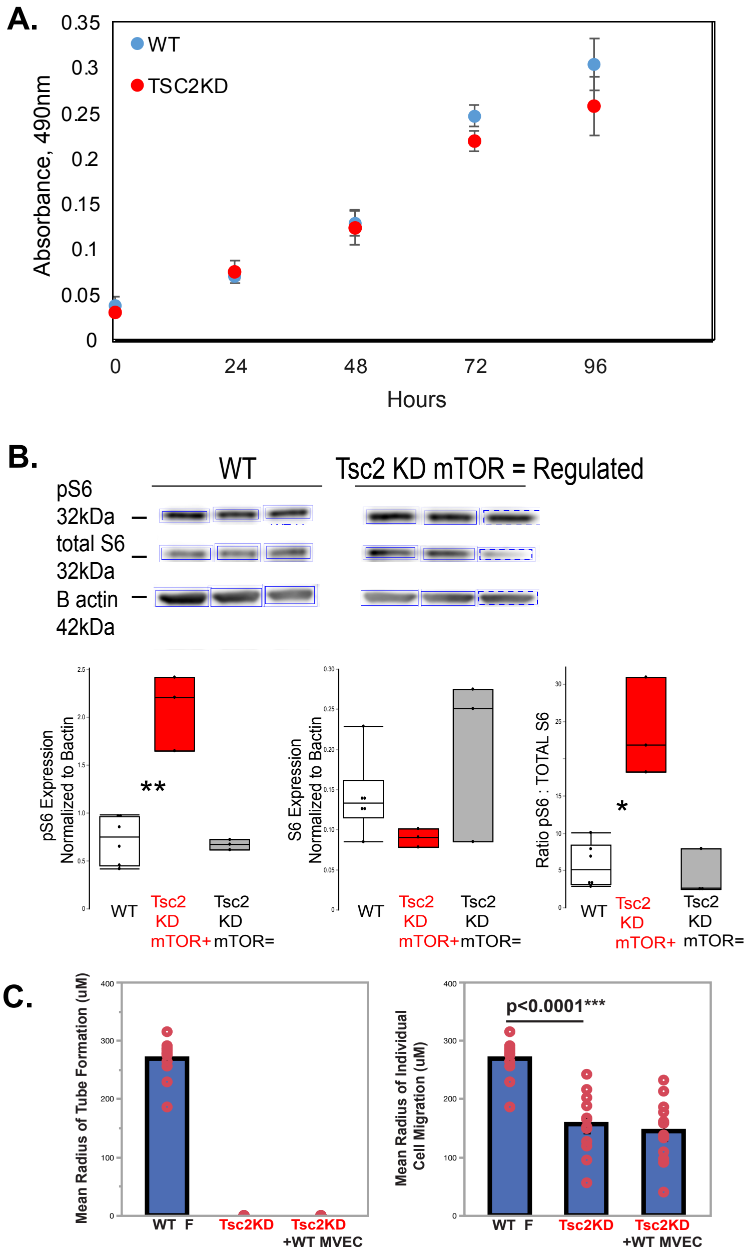

Supplemental Figure 6 (Figure 4). Tsc2KD in MVPC does not alter cell proliferation. A. 2500 murine MVPC per well were plated in a 96 well plate and proliferation was analyzed using CellTiter 96AQ$_{ueous}$One Solution (Promega, Madison, WI) at the time points stated. The assay was performed in triplicate with four independent replicates. Data were analyzed by one-way ANOVA followed by Tukey's HSD post-hoc analysis and presented as mean (+/-SEM). *p<0.05, **p<0.01, ***p<0.001. **B.** Quantification of pS6 and total S6 by Western blot to determine mTOR activation state. We identified one cell line that was knockdown for Tsc2 and mTOR activated (red) and a second line that was knockdown for Tsc2 and mTOR regulated (grey) similar to WT levels. Data were analyzed by one-way ANOVA followed by Tukey's HSD post-hoc analysis and presented as mean (+/-SEM). *p<0.05, **p<0.01. **C.** Spheroids composed of MVPC alone or MVPC + WT MVEC were formed overnight in agarose molds and transferred to collagen for up to 72 hours(9). The radius of sprouts and migrating cells was quantitated. The experiment was repeated twice independently and a total of 20 spheroids were quantitated per group and are represented by the open circles. Data were analyzed by one-way ANOVA followed by Tukey's HSD post-hoc analysis and presented as mean (+/-SEM). *p<0.05, **p<0.01, ***p<0.001.

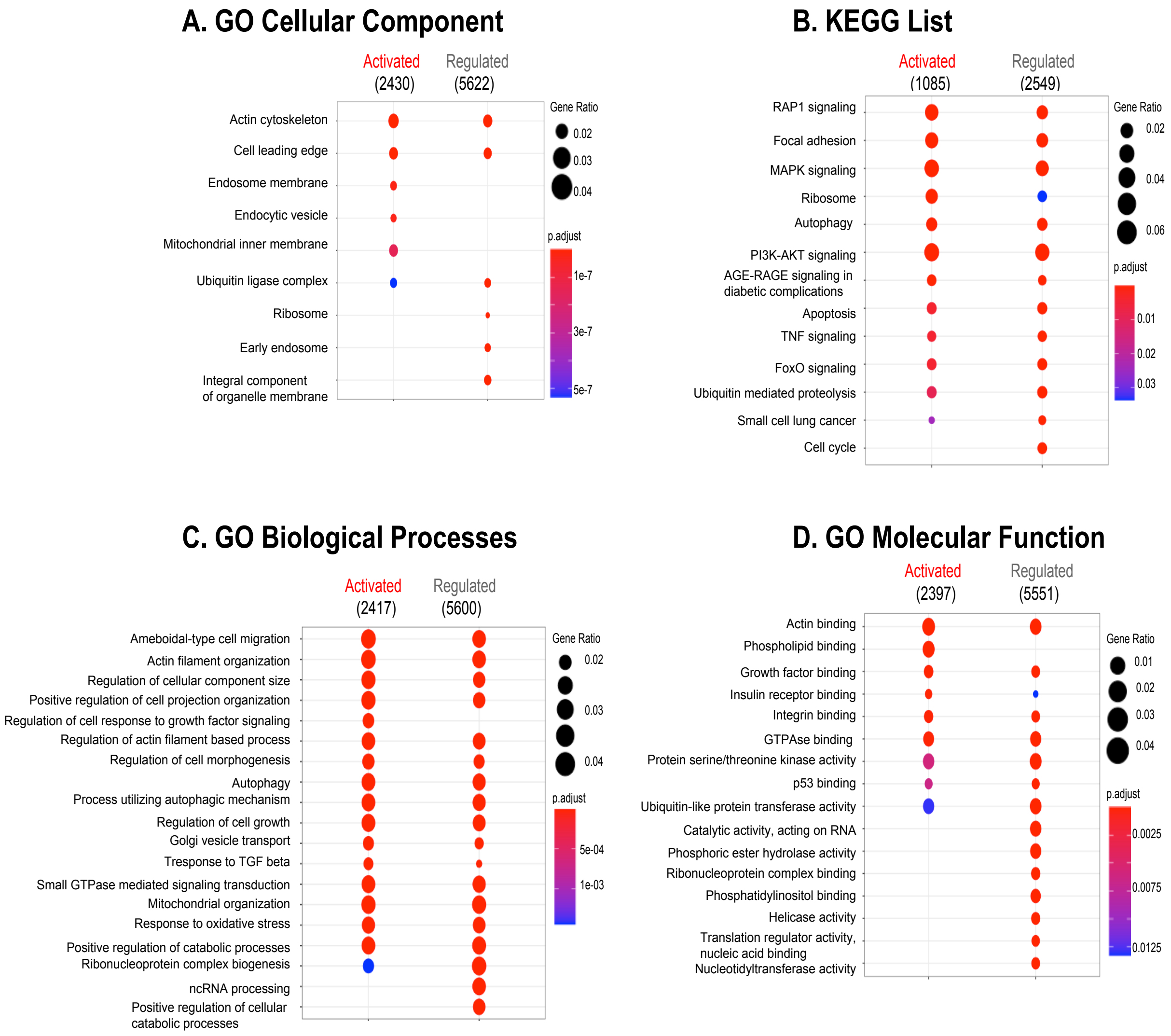

Supplemental Figure 7. Bulk RNA seq of isolated MVPC identified targets of Tsc2/mTOR regulation (Figure 5). Unbiased transcriptomic comparison between primary WT, mTOR activated and mTOR regulated cell lines using KEGG and gene ontology (GO) pathway analyses. Gene lists are included in Tables 1&2 and Supplemental Table 4.

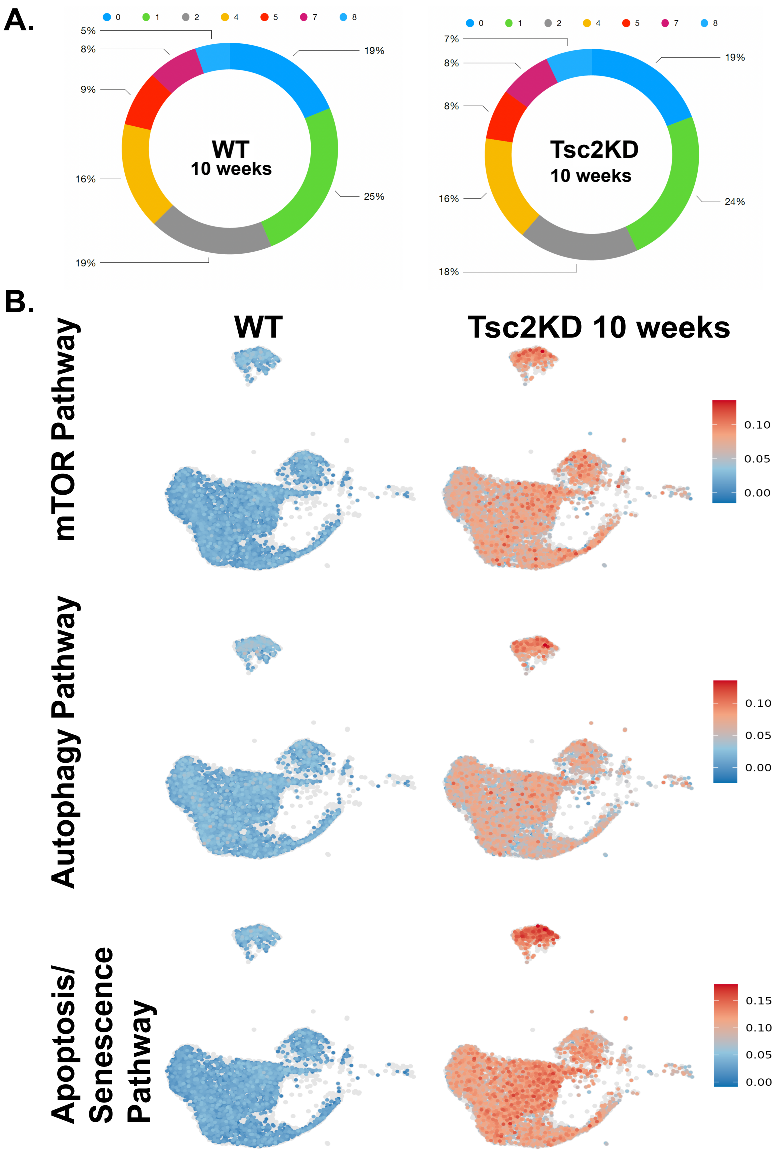

Supplemental Figure 8 (Figures 6&7, Supplemental Table 6). Feature plots of WT versus Tsc2KD MVPC at 10 weeks showing enrichment in select pathways. Unbiased transcriptomic comparison between WT and Tsc2KD MVPC 10 weeks post tamoxifen **A.** demonstrated % cells between clusters and **B.** enrichment in three pathways/functions of interest, namely, autophagy, mTOR pathway and apoptosis/senescence. Relevant terms are available via the mouse MSIGDB database (https://www.gsea-msigdb.org/gsea/msigdb/, **Supplementary Table 6**). Genes associated with the terms were extracted using msigdb package(Bioconductor). Enrichment scores were then calculated for the three sets of genes associated with terms related to the three functions/pathway of interest using AddModuleScore function from the Seurat package. These scores were then mapped onto feature plots using the dittoDimPlot function available in the dittoSeq package(Bioconductor) with the parameter *adjustmen*t set to "relative.to.max".

SUPPLEMENTAL METHODS

Characterization of physiological and structural alterations in the lung as a result of Tsc2KD in MVPC. Lung leak was measured by collecting bronchoalveolar lavage fluid (BALF) and samples were run in triplicate using an Albumin ELISA kit (Fortis Life Sciences, Waltham, MA) following the manufacturer's protocol. Pulmonary artery pressure was documented indirectly by the measurement of right ventricular systolic pressure (RVSP) in an open-chested mouse with a 1.2 French Pressure-Volume Catheter (FTH-1212B-3518 or 4018, Transonic Systems Inc., Ithaca, NY) coupled with quasi-static mechanical properties of the lung measured using the *flexi*Vent invasive plethysmography system (SciREQ, Inc., Montreal, Canada) as previously described(8, 101). To prevent spontaneous respirations, anesthetized mice received 0.8-1.2mg/kg of Pancuronium bromide (Millipore Sigma, St. Louise, MO) via intraperitoneal (i.p.) injection. The following measures of respiratory mechanics were calculated from the *flexi*Vent: Inspiratory Capacity (IC), static compliance (CST), compliance of respiratory system (CRS), elastance of respiratory system (ERS, and Pressure-volume (PV) determined Area Under Curve (AUC).*In vivo* microCT analysis was also performed to quantitate lung structure and function. Mice were anesthetized with 1.5-2.5% isoflurane in 100% oxygen (MWI Veterinary Supply, Boise, ID), placed on a nose cone in a scanning bed in dorsal recumbency and imaged using the NJH Bruker SkyScan 1276 microCT system (Bruker, Allentown, PA). A single 3-dimensional (3D) micro-CT image set was acquired for each mouse using the following parameters: exposure of 121-132ms exposure time, filter Al at 0.5mm, 70 kVp X-ray source voltage, current at 200μA, pixel size of 40.5μm, rotation step of 0.7°, 180° scan over approximately 7 minutes. After scanning, the scans were gated for breath cycle, using the diaphragm location as a guide, using DataViewer software from Bruker. Postprocessing included reconstruction using Bruker NRecon software (v 1.7.4.2) and analyses in Bruker CTAn (v 1.17.7.2+). The analysis included the average density of the lung in aerated lung volume (mm^3) and Houndsfield Units (HU). Briefly, using CTAn software, the CT scan was transformed into a binary image and a density threshold for what was considered aerated lung tissue vs other tissues was chosen by the person performing the data analysis.

This threshold was chosen based on closely matching the binary image of the scan to the actual image of the scan. This same threshold was then applied to each scan. With CTAn software, an automatic series of computer operations separated the aerated lung tissue from the rest of the mouse body, and this was quantitated in mm^3. The Hounsfield Units (HU) were obtained with this same program from a calculation that was based on a CT scan of distilled water serving as a standard for the CT scanner. To illustrate the lung density difference between groups, within the aerated lung volume, all tissue with HU from-635 to -100 was selected and color was applied to this tissue. This range of HU represents the areas of lung with the lowest density.

To analyze changes in lung structure, lungs were inflated using 0.75% low-melt agarose (Isc Bio Express, Kaysville, UT) in phosphate buffered saline (PBS) (Fisher, Hampton, NH) and fixed in 10% Buffered Formalin (Fisher, Hampton, NH) overnight. 5μm thick paraffin sections were stained with standard hematoxylin-eosin (H&E) or Masson's trichrome(8). Histologic endpoints included mean linear intercept (MLI), trichrome quantitation of collagen, muscularization and microvessel density. Images of H&E stained lung sections were taken on a Nikon Eclipse 80i microscope using NIS Elements BR software (v 5.30.03). A minimum of 10 nonoverlapping images of the distal lung were captured at x20. Images were then loaded into a macro within Metamorph Software (v 7.5.0.0) for analysis. MLI was calculated from the average of all the images(102). Masson's trichrome stained sections were captured on a Keyence BZ-X810 microscope using BZ-X800 software (v 2.0 01.02.02.04). A minimum of 10 nonoverlapping images of the distal lung were taken at x40. Images were scanned using Fiji (Image J; v 2.0.0-rc-43/1.51a) using a custom plug-in written by M. Majka (Denver, CO). Immunostaining to detect smooth muscle actin (SMA; Dako clone 1A4) or Factor 8 (A0082 DAKO) was used to quantitate muscularization and microvessel density (6-8 mice per group)(9, 10, 103). Immunofluorescent staining was performed to lineage trace eGFP-labeled lung MVPC as described(9, 10) as well as human MVPC expression of pS6 (#2211S, Cell Signaling Technology, Danvers, MA) and TSC2 (#3612S, Cell Signaling Technology, Danvers, MA).

Ex vivo analyses of MVPC Function. Murine and human MVPC isolation and characterization was performed as previously described, cells sorted and characterized for the expression of mesenchymal progenitor markers (CD105, CD106, CD73) and lack of pericyte, endothelial and inflammatory markers (CD45, B220, F480, CD14, CD34, CD31, VE-Cad)(8-10, 38, 40) (Reagent list **Supplemental Table 7)**. The spheroid sprouting assay and quantitation were performed as we previously described and analyzed using Wimasis WimSprout image analysis software (9). Briefly, sprouting cells were visible between 24-72 hours and visualized by bright field or fluorescence imaging of membrane eGFP. Cell Proliferation was analyzed using CellTiter 96AQ$_{ueous}$One Solution (Promega, Madison, WI) following the manufacturer's protocol. Mouse or human MVPC were plated at 2,500 cells per well in a 96-well plate. The assay was performed three times independently with all samples in triplicate. Electric Cell-substrate Impedance Sensing (ECIS; Applied Biophysics, Troy, NY) was performed to examine migration and repair of barrier function following a "wounding" stimulus in co-cultures of Normal or mTOR+MVPC and human lung microvascular endothelium. Human lung MVECs (Lonza, Walkersville, MD) were plated at a concentration of 112,500 cells per well on gelatin coated 8W1E PET ECIS culture ware arrays overnight to achieve confluence. The following day, MVPCs were added at a concentration of 37,500 cells per well(9). Controls for these experiments included independent untreated MVEC and MVEC with wounding. On the third day, the arrays were inserted into the ECIS ZTheta, and resistance recordings were performed at 4 kHz every 10 minutes over 24 hours. At 2-3 hours, an electrical wound was created by administering a 20 second pulse at 60,000 Hz. The pulse was immediately repeated to ensure cell death. These experiments were performed with two sample replicates and repeated twice.

Genomic analyses of MVPC. Sequencing of the TSC2 gene in MVPC was performed using 500ng of MVPC genomic DNA for whole genome sequencing and the Nova seq platform, with the parameters of PE150 with an average coverage of 30x, using the Vanderbilt University Medical Center Vantage Core facility. The variant effect predictor website HG37/HG19

(http://grch37.ensembl.org/Homo_sapiens/Tools/VEP) was used to identify small nucleotide polymorphisms (SNPs) in TSC2 (ENSG00000103197.18) the gene of interest.

Bulk RNASeq differential gene expression analysis of murine MVPC (GSE 225656; NCBI23699747): Sample counts were generated by the sequencing center at National Jewish Health. BBDuk was used for read trimming. Adapter sequences and the first 12 bases at the 5' end were removed, retaining reads that were greater than 50 bp. Trimmed reads were aligned and raw counts were generated with STAR using mouse mg38.p6 (ENSEMBL release 96) as the reference. The raw counts were further processed and analyzed using R (v 4.0.2) and Bioconductor (v 3.11). The count data were first filtered for low counts by removing all rows, whose averages were less than or equal to 10. Next, the MGI symbols were annotated for gene biotype using the Bioconductor package, biomaRt. Only genes/transcripts with the biotype "protein-coding" were retained for further analyses. To control for GC content and gene length variation that can influence downstream results, normalization using the Bioconductor package, CQN (conditional quantile normalization) was used. Differential expression (DE) statistics were generated using the Bioconductor package, limma. Genes with an average expression value above 2 across mutant and wild type samples and adjusted p-values below 0.05 were used for further analyses. To explore the large DE lists and compare the changes in female and male knockouts, we employed multiple approaches. First, two STRING networks of functional interactions of all DE genes in the two lists were generated using Cytoscape App (v 3.8.2). These networks were clustered using the MCODE algorithm to identify densely functionally connected subnetworks. To get a comparative visual representation of affected Reactome categories in the two gene lists, we employed the CompareCluster function from the Bioconductor package, clusterProfiler. We, then, employed the self-contained gene set test available via the mROAST function of the limma package to test all mSigDb curated mouse Reactome gene sets. This enabled the identification of Reactome categories that were statistically significantly changed. Thus, we employed functional interaction information from STRING database, visual comparison with clusterProfiler and statistical testing with mROAST in limma to

bioinformatically investigate the functional themes of TSC2 knockdown and the differences between mTOR activated versus mTOR regulated MVPC lines.

ScRNA sequencing was performed using the Chromium Single Cell 3' Library and Gel Bead Kit (v 3.1 10x Genomics, Pleasanton, CA) and the Chromium X. Libraries were sequenced on an Illumina Novaseq 6000. Sequencing guidelines for scRNA-seq include producing at least 50,000 sequencing reads per cell to ensure adequate depth of coverage for comparative analysis as we previously reported(9). Demultiplexing, alignment to the mm10 transcriptome, and UMI-collapsing was performed using the Cellranger toolkit (v5.0, 10X Genomics, Pleasanton, CA). Data were collected on 95,037 cells from 12 samples (WT and Tsc2KD 2 days post tamoxifen induction: $CD45^{neg}$ GFP^{pos}(5885cells WT; 3456cells KD), $CD45^{neg}$, $CD45^{pos}$; WT and Tsc2KD 10wk post tamoxifen induction: $CD45^{neg}$ GFP^{pos}(6406cells WT; 6019cells KD), $CD45^{neg}$, $CD45^{pos}$. All post-quantification quality control, normalization, integration, clustering, and marker finding were performed in Seurat (v3.1, Satija Lab(104)). We filtered out low quality cells with less than 200 genes detected or with greater than 7% of mapped reads originating from the mitochondrial genome. We also removed cells with a UMI count greater than the 98th percentile of UMI counts for each sample to limit doublets. Prior to downstream analysis, select mitochondrial and ribosomal genes (genes beginning with MT-, MRPL, MRPS, RPL, or RPS) were removed. The quality-controlled dataset consisted of 91,308 cells. UMI counts were normalized using the SCTransform method based on GLM (v1) in the Seurat R package(104, 105). In addition to adjusting for sequencing depth, we also adjusted for the proportion of mitochondrial reads and doublets using the scds R package. We did not find clusters enriched for high doublet scores. Data from the 12 samples were combined using single cell integration implemented in Seurat, which identifies mutual nearest neighbor (MNN) cells across pairwise subjects to use as "anchors" to perform batch correction. Integration was carried out using the top 30 dimensions from a canonical correlation analysis (CCA) based on SCTransform-normalized expression of the top 3,000 genes, defined by gene dispersion using the Seurat's SelectIntegrationFeatures function. Integrated data were then clustered

and visualized using the top 25 principal components. For visualization, we reduced variation to two dimensions using Uniform Manifold Approximation and Projection(106) (UMAP; n.neighbors = 50, min.dist = 0.3). Unsupervised clustering was performed using a shared nearest neighbor (SNN) graph based on 20-nearest neighbors and then determining the number and composition of clusters using a smart local moving (SLM) algorithm (resolution = 0.6). This algorithm identified 28 clusters. To identify cluster markers, we carried out pairwise differential expression analysis comparing SCTransform-normalized expression in each cluster to all others using a Wilcoxon rank sum test. Markers were identified as genes exhibiting significant upregulation when compared against all other clusters, defined by having a Bonferroni adjusted p-value < 0.05, a log fold change > 0.25, and >10% of cells with detectable expression. This analysis was then performed separately for each subject using Seurat's FindConservedMarkers function to determine if marker genes were consistent across samples. Cluster specific analyses were used to compare gene expression between conditions separately for CD45 negative GFP positive, CD45 positive, and CD45 negative samples. Pairwise differential expression was performed using a Wilcoxon rank sum test. Differentially expressed genes were defined by having a Bonferroni adjusted p-value < 0.05, a log fold change > 0.1, >1, and >5% of cells with detectable expression.

Microarray analysis of human lung MVPC was performed as previously described(9, 10, 39, 107) (GSE 225760, NCBI 23718621), in triplicate or with an n of three or greater independent patient samples. Complimentary DNA generated from amplified RNA was hybridized to duplicate Affymetrix Human gene 1.0 st chips (Santa Clara, CA). Differential expression analysis was carried out using Bioconductor (v3.2, R 3.2.2). Raw probe level data were read in, using oligo package. After normalization with RMA, moderated t-test implemented in the limma package was employed for differential expression testing. A minimal fold change of 1.7X, up or down and p<0.05 were employed as criteria for defining differentially expressed genes. Expression values for these genes are represented in a

heatmap; generated using the pheatmap function in R. Representative genes were selected for target validation (Reagent list **Supplemental Table 7)**.

Western blot. Adherent mouse or human MVPC were lysed with RIPA Buffer (#9806S, Cell Signaling, Boston, MA) containing protease and phosphatase inhibitors (#78444, ThermoFisher Scientific, Waltham, MA) and scraped to collect protein extracts. Protein concentration was determined using a Pierce BCA Protein Assay Kit (#23227, ThermoFisher Scientific, Waltham, MA) following the manufactures' instructions. After standardization, protein extracts were mixed with an equal volume of Laemmli Sample Buffer (#1610747, BioRad, Hercules, CA), resolved on 1.5mm thick NuPAGE 4-12% Bis-Tris gels (ThermoFisher, Waltham, MA), and transferred to PVD membranes. The blots were blocked with phosphate buffered saline (PBS) (Fisher, Hampton, NH) containing 5% dry milk (BioRad, Hercules, CA) and 0.1% Tween 20 (Sigma-Aldrich, St. Louis, MO), and then treated with antibodies to detect target protein overnight at 4°C (Reagent list **Supplemental Table 7)**. The blots were washed and subsequently treated with appropriate secondary antibodies conjugated to horseradish peroxidase. Blots were washed one more time and specific immune complexes were visualized with Clarity Western ECL Substrate (BioRad, Hercules, CA). A Licor Odyssey Imager (Lincoln, NE) was used for analysis with Licor Image Studio Software (v 5.2.5).

1. Summers ME, Richmond BW, Menon S, Sheridan RM, Kropski JA, Majka SA, et al. Resident mesenchymal vascular progenitors modulate adaptive angiogenesis and pulmonary remodeling via regulation of canonical Wnt signaling. *The FASEB Journal.* 2020;n/a(n/a).
2. Kopf KW, Harral JW, Staker EA, Summers ME, Petrache I, Kheyfets V, et al. Optimization of combined measures of airway physiology and cardiovascular hemodynamics in mice. *Pulmonary Circulation.* 2020;10(1):2045894020912937.
3. Summers ME, Richmond BW, Kropski JA, Majka SA, Bastarache JA, Hatzopoulos AK, et al. Balanced Wnt/Dickkopf-1 signaling by mesenchymal vascular progenitor cells in the microvascular niche maintains distal lung structure and function. *Am J Physiol Cell Physiol.* 2021;320(1):C119-c31.
4. McGrath-Morrow SA, Cho C, Soutiere S, Mitzner W, and Tuder R. The Effect of Neonatal Hyperoxia on the Lung of p21Waf1/Cip1/Sdi1-Deficient Mice. *American Journal of Respiratory Cell and Molecular Biology.* 2004;30(5):635-40.
5. Case D, Irwin D, Ivester C, Harral J, Morris K, Imamura M, et al. Mice deficient in galectin-1 exhibit attenuated physiological responses to chronic hypoxia-induced pulmonary hypertension. *Am J Physiol Lung Cell Mol Physiol.* 2007;292(1):L154-64.
6. Gaskill CF, Carrier EJ, Kropski JA, Bloodworth NC, Menon S, Foronjy RF, et al. Disruption of lineage specification in adult pulmonary mesenchymal progenitor cells promotes microvascular dysfunction. *The Journal of Clinical Investigation.* 2017;127(6):2262-76.
7. Marriott S, Baskir RS, Gaskill C, Menon S, Carrier EJ, Williams J, et al. ABCG2(pos) lung mesenchymal stem cells are a novel pericyte subpopulation that contributes to fibrotic remodeling. *American Journal of Physiology - Cell Physiology.* 2014;307(8):C684-C98.
8. Summers M, Karen Helm, Susan M. Majka. *Enrichment and Characterization of Human and Murine Pulmonary Mesenchymal Progenitor Cells (MPC Mesenchymal progenitor cell (MPC)* Springer Protocols; 2020.
9. Stuart T, Butler A, Hoffman P, Hafemeister C, Papalexi E, Mauck WM, III, et al. Comprehensive Integration of Single-Cell Data. *Cell.* 2019;177(7):1888-902.e21.
10. Butler A, Hoffman P, Smibert P, Papalexi E, and Satija R. Integrating single-cell transcriptomic data across different conditions, technologies, and species. *Nature Biotechnology.* 2018;36(5):411-20.
11. McInnes L, and Healy J. UMAP: Uniform Manifold Approximation and Projection for Dimension Reduction. *ArXiv.* 2018;abs/1802.03426.
12. Gaskill C, Marriott S, Pratap S, Menon S, Hedges LK, Fessel JP, et al. Shared gene expression patterns in mesenchymal progenitors derived from lung and epidermis in pulmonary arterial hypertension: identifying key pathways in pulmonary vascular disease. *Pulmonary Circulation.* 2016;6(4):483-97.
13. West JD, Austin ED, Gaskill C, Marriott S, Baskir R, Bilousova G, et al. Identification of a common Wnt-associated genetic signature across multiple cell types in pulmonary arterial hypertension. *American Journal of Physiology - Cell Physiology.* 2014;307(5):C415-C30.

SUPPLEMENTAL TABLE 1. Identified Low Prevalence Somatic *TSC2* Mutations

Gene	***Nucleotide Change***	***Db SNP***	***Chromosome Position***
TSC2			
	A>G	rs3760042	16:2049231
**Chromosome**	A>G	rs2073636	16:2055054
**16**	G>C	rs28709874	16:2059251
5'-3'	C>G	rs2074968	16:2060570
	T>C	rs17135764	16:2061778
	A>C	rs13334473	16:2071000
	C>T	rs45517384 (Regulatory region;3'UTR TSC2/PDK1)	16:2086841
	A<C	rs1800718 (Regulatory region;3'UTR TSC2/PDK1)	16:2088217
	*delCACA	rs374706125 (downstream variant 500B, utr variant 3 prime PDK1 polycystin1; tail to tail)	16:2088882

Supplemental Table 2. HUMAN mTOR Activated MVPC GO Gene Lists (Figure 2D)

GO category	log P	GeneSet
mRNA transport	10	ALYREF,BUD13,DDX39A,GLE1,HNRNPA3,NDC1,NUP107,NUP153,NUP155,NUP160,NUP188,NUP205,NUP35,NUP43,NUP58,NUP85,NUP88,SRSF7,THOC3
G2/M transition of mitotic cell cycle	10	AURKA,BIRC5,BLM,BORA,CCNA2,CCNB1,CCNB2,CCP110,CDC25A,CDC25C,CDC7,CDK1,CDK2,CDKN2A,CDKN2B,CENPF,CENPJ,CEP135,CEP152,CEP192,CEP76,CEP78,CHEK1,CIT,CKAP5,CLSPN,CNTRL,FANCI,FOXM1,HAUS4,HAUS8,HMMR,KIF14,LMNB1,MASTL,MELK,NDE1,NEK2,ODF2,PKMYT1,PLK1,PLK4,TICRR,TPX2
interstrand cross-link repair	10	DCLRE1B,DCLRE1C,EME1,FANCA,FANCB,FANCC,FANCD2,FANCG,FANCI,FANCM,UBE2T,USP1
negative regulation of RNA biosynthetic process	10	ASF1A,ATAD2,ATOH8,AURKB,BIRC5,BRCA1,CASP8AP2,CDC45,CDKN2A,CENPF,CTCF,DEPDC1,DNMT1,E2F8,EZH2,FOXM1,GMNN,H2AFX,H2AFZ,HAT1,HELLS,HIST1H1B,HIST1H1C,HIST1H1D,HIST1H1E,HIST1H2AB,HIST1H2AC,HIST1H2AD,HIST1H2AE,HIST1H2AG,HIST1H2AH,HIST1H2AI,HIST1H2AJ,HIST1H2AK,HIST1H2AL,HIST1H2AM,HIST1H3A,HIST1H3B,HIST1H3C,HIST1H3F,HIST1H3G,HIST1H3H,HIST1H3I,HIST1H3J,HIST1H4A,HIST1H4B,HIST1H4C,HIST1H4D,HIST1H4E,HIST1H4F,HIST1H4I,HIST1H4J,HIST1H4K,HIST1H4L,HIST2H2AA3,HIST2H2AA4,HIST2H2AB,HIST2H2AC,HIST2H3A,HIST2H3C,HIST2H3D,HMGB1,HMGB2,HMGN2,ID1,LRRFIP1,MTERF3,NOTCH2,PARP1,PCNA,PHB2,PHF19,PLK1,PRDM1,RBBP8,RBL1,SUV39H1,SUV39H2,TIMELESS,TRIM28,UHRF1,ZNF85,ZNF93
cell cycle phase transition	10	ANAPC1,ANAPC15,ANLN,AURKA,BIRC5,BLM,BORA,BUB1,BUB1B,BUB3,CASP2,CCNA2,CCNB1,CCNB2,CCNE2,CCP110,CDC20,CDC25A,CDC25C,CDC45,CDC6,CDC7,CDCA5,CDK1,CDK2,CDKN2A,CDKN2B,CDKN2C,CDKN3,CDT1,CENPE,CENPF,CENPJ,CEP135,CEP152,CEP192,CEP76,CEP78,CHEK1,CIT,CKAP5,CKS1B,CKS2,CLSPN,CNTRL,DHFR,DHFRP1,DLGAP5,ESPL1,EZH2,FANCI,FBXO5,FOXM1,GEN1,GSG2,GTSE1,HAUS4,HAUS8,HMMR,IQGAP3,KIF14,KNTC1,LMNB1,MAD2L1,MASTL,MCM10,MCM2,MCM3,MCM4,MCM5,MCM6,MCM7,MCM8,MELK,MTBP,NDE1,NEK2,ODF2,ORC1,ORC5,ORC6,PAXIP1,PCNA,PHB2,PKMYT1,PLK1,PLK4,POLA1,POLA2,POLE,POLE2,PRIM1,RANBP1,RBBP8,RCC1,RFWD3,RRM2,STIL,SUSD2,TCF19,TICRR,TIMELESS,TIPIN,TPX2,TTK,TYMS,UBE2C,UBE2S
regulation of cell division	10	ANAPC15,ANLN,ASPM,AURKA,AURKB,BIRC5,BLM,BORA,BRCA2,BUB1,BUB1B,BUB3,CCNB1,CCP110,CDC20,CDC25C,CDC6,CDCA5,CENPE,CENPF,CEP192,CEP97,CHEK1,CIT,DLGAP5,E2F8,ECT2,ESPL1,FBXO5,GEN1,KIF11,KIF14,KIF18B,KIF20B,KIF23,KNTC1,MAD2L1,MSH2,MTBP,NEK2,NUSAP1,PDE3A,PHB2,PKMYT1,PLK1,PTTG1,RACGAP1,RANBP1,RCC1,SLF1,STIL,SUSD2,TTK,UBE2C
DNA integrity checkpoint	10	AURKA,BLM,BRCA1,BRIP1,CASP2,CCNA2,CCNB1,CDC25C,CDC45,CDC6,CDK1,CDK2,CDT1,CENPJ,CHEK1,CLSPN,DNA2,DTL,FANCI,GTSE1,H2AFX,MSH2,PCNA,PLK1,RBBP8,RFWD3,TICRR,TIPIN,TOP2A
negative regulation of chromosome segregation	10	ANAPC15,BUB1,BUB1B,BUB3,CCNB1,CENPE,CENPF,CEP192,ESPL1,GEN1,MAD2L1,MTBP,PLK1,PTTG1,STIL,TTK
cytoskeleton-dependent cytokinesis	10	ANLN,AURKB,CEP55,CKAP2,KIF20A,KIF20B,KIF23,KIF4A,KIF4B,NUSAP1,PLK1,RACGAP1,STMN1
regulation of chromosome organization	10	ACD,ANAPC15,ASF1A,ATAD2,AURKB,BRCA1,BUB1,BUB1B,BUB3,CCNB1,CDC45,CDC6,CDCA5,CENPE,CENPF,CEP192,CHEK1,CIT,CTCF,DLGAP5,DNMT1,ESPL1,GEN1,HIST1H1B,MAD2L1,MTBP,NEK2,PARP1,PAXIP1,PHF19,PLK1,PTTG1,SLF1,STIL,TCP1,TRIM28,TTK,UBE2C,UHRF1,WDHD1
protein tetramerization	10	HIST1H3A,HIST1H3B,HIST1H3C,HIST1H3F,HIST1H3G,HIST1H3H,HIST1H3I,HIST1H3J,HIST1H4A,HIST1H4B,HIST1H4C,HIST1H4D,HIST1H4E,HIST1H4F,HIST1H4I,HIST1H4J,HIST1H4K,HIST1H4L,HIST2H3D,HPRT1,NUP58,RRM1,RRM2,SOD2,TK1
DNA packaging	10	ASF1A,ASF1B,CCNB1,CDCA5,CDK1,CDKN2A,CENPA,CENPH,CENPI,CENPK,CENPL,CENPM,CENPN,CENPO,CENPP,CENPQ,CENPU,CENPW,CHAF1A,CHAF1B,CTCF,H2AFX,H2BFS,HAT1,HELLS,HIST1H1A,HIST1H1B,HIST1H1C,HIST1H1D,HIST1H1E,HIST1H2BB,HIST1H2BC,HIST1H2BD,HIST1H2BE,HIST1H2BF,HIST1H2BG,HIST1H2BH,HIST1H2BI,HIST1H2BK,HIST1H2BL,HIST1H2BM,HIST1H2BN,HIST1H2BO,HIST1H3A,HIST1H3B,HIST1H3C,HIST1H3F,HIST1H3G,HIST1H3H,HIST1H3I,HIST1H3J,HIST1H4A,HIST1H4B,HIST1H4C,HIST1H4D,HIST1H4E,HIST1H4F,HIST1H4I,HIST1H4J,HIST1H4K,HIST1H4L,HIST2H2BD,HIST2H2BE,HIST2H2BF,HIST2H3A,HIST2H3C,HIST2H3D,HJURP,HMGB1,HMGB2,ITGB3BP,MCM2,MIS18A,NASP,NCAPD2,NCAPD3,NCAPG,NCAPG2,NCAPH,NUSAP1,RUVBL1,SMC2,SMC4,SUV39H1,TOP2A
ATP-dependent chromatin remodeling	10	ACTL6A,ANP32E,CENPA,CENPH,CENPI,CENPK,CENPL,CENPM,CENPN,CENPO,CENPP,CENPQ,CENPU,CENPW,HIST1H4A,HIST1H4B,HIST1H4C,HIST1H4D,HIST1H4E,HIST1H4F,HIST1H4I,HIST1H4J,HIST1H4K,HIST1H4L,HJURP,ITGB3BP,MIS18A,NASP,RUVBL1
nuclear chromosome segregation	10	ANAPC15,AURKB,BIRC5,BRCA2,BUB1,BUB1B,BUB3,CCNB1,CDC20,CDC6,CDCA5,CDCA8,CENPA,CENPE,CENPF,CENPH,CENPI,CENPK,CENPL,CENPM,CENPN,CENPO,CENPP,CENPQ,CENPU,CEP192,CEP55,CIT,CKAP5,CTCF,DDX11,DLGAP5,DMC1,DSCC1,DSN1,ECT2,ERCC6L,ESPL1,FANCD2,FANCM,GEN1,GSG2,INCENP,ITGB3BP,KIF14,KIF18A,KIF18B,KIF22,KIF23,KIF2C,KIF4A,KIF4B,KIFC1,KNSTRN,KNTC1,MAD2L1,MCMBP,MLH1,MTBP,NCAPD2,NCAPD3,NCAPG,NCAPH,NDC1,NDC80,NDE1,NEK2,NUF2,NUP107,NUP160,NUP43,NUP85,NUSAP1,PHB2,PLK1,PRC1,PSRC1,PTTG1,RACGAP1,SGO1,SKA1,SKA2,SLF1,SMC1A,SMC2,SMC3,SMC4,SPAG5,SPC24,SPC25,SPDL1,STIL,TOP2A,TRIP13,TTK,UBE2C,ZWILCH,ZWINT
spindle checkpoint	10	ANAPC15,AURKA,AURKB,BIRC5,BUB1,BUB1B,BUB3,CCNB1,CENPE,CENPF,CEP192,GEN1,GSG2,MAD2L1,MTBP,PLK1,SPDL1,STIL,TTK
regulation of mitotic nuclear division	10	ANAPC15,ANLN,AURKA,BIRC5,BORA,BUB1,BUB1B,BUB3,CCNB1,CDC25C,CDC6,CDCA5,CENPE,CENPF,CEP192,CEP97,CHEK1,CIT,DLGAP5,ESPL1,FBXO5,GEN1,KIF11,KIF20B,KNTC1,MAD2L1,MTBP,NEK2,NUSAP1,PHB2,PKMYT1,PLK1,PTTG1,RANBP1,RCC1,SLF1,STIL,TTK,UBE2C
spindle assembly checkpoint	10	ANAPC15,BUB1,BUB1B,BUB3,CCNB1,CENPE,CENPF,CEP192,GEN1,MAD2L1,PLK1,TTK
DNA ligation involved in DNA repair	10	HMGB1,HMGB2,LIG1,PARP1,PARP2,PARP3
negative regulation of mitotic cell cycle phase transition	10	ANAPC15,AURKA,BLM,BUB1,BUB1B,BUB3,CASP2,CCNA2,CCNB1,CDC25C,CDK1,CDK2,CDKN2B,CENPE,CENPF,CENPJ,CEP192,CHEK1,CLSPN,EZH2,FANCI,GEN1,GSG2,GTSE1,MAD2L1,MTBP,PCNA,PLK1,RFWD3,STIL,TICRR,TTK
mitotic cell cycle checkpoint	10	ANAPC15,AURKA,BLM,BUB1,BUB1B,BUB3,CASP2,CCNA2,CCNB1,CDC25C,CDK1,CDK2,CDKN2B,CENPE,CENPF,CENPJ,CEP192,CLSPN,FANCI,GEN1,GTSE1,KNTC1,MAD2L1,MSH2,MTBP,PCNA,PLK1,RFWD3,SMC1A,STIL,TICRR,TIPIN,TOP2A,TTK,ZWILCH,ZWINT
mitotic spindle assembly checkpoint	10	ANAPC15,BUB1,BUB1B,BUB3,CCNB1,CENPE,CENPF,CEP192,GEN1,MAD2L1,PLK1,TTK
negative regulation of proteasomal ubiquitin-dependent protein catabolic process	10	ANAPC15,BUB1,BUB1B,BUB3,CCNB1,CENPE,CENPF,CEP192,GEN1,MAD2L1,MTBP,PBK,PLK1,SENP1,STIL,TTK
nucleosome organization	10	ANP32E,ASF1A,ASF1B,CENPA,CENPH,CENPI,CENPK,CENPL,CENPM,CENPN,CENPO,CENPP,CENPQ,CENPU,CENPW,CHAF1A,CHAF1B,CTCF,H2AFX,H2BFS,HAT1,HIST1H1A,HIST1H1B,HIST1H1C,HIST1H1D,HIST1H1E,HIST1H2BB,HIST1H2BC,HIST1H2BD,HIST1H2BE,HIST1H2BF,HIST1H2BG,HIST1H2BH,HIST1H2BI,HIST1H2BK,HIST1H2BL,HIST1H2BM,HIST1H2BN,HIST1H2BO,HIST1H3A,HIST1H3B,HIST1H3C,HIST1H3F,HIST1H3G,HIST1H3H,HIST1H3I,HIST1H3J,HIST1H4A,HIST1H4B,HIST1H4C,HIST1H4D,HIST1H4E,HIST1H4F,HIST1H4I,HIST1H4J,HIST1H4K,HIST1H4L,HIST2H2BD,HIST2H2BE,HIST2H2BF,HIST2H3A,HIST2H3C,HIST2H3D,HJURP,HMGB2,ITGB3BP,MCM2,MIS18A,NASP,RUVBL1
regulation of ubiquitin protein ligase activity	10	ANAPC1,ANAPC15,BUB1B,BUB3,CCNB1,CDC20,CDK1,CDK2,FBXO5,MAD2L1,MASTL,PLK1,UBE2C,UBE2S

attachment of spindle microtubules to kinetochore	10	AURKB,BRCA2,BUB3,CCNB1,CENPE,ECT2,KNSTRN,NDC80,NEK2,NUF2,RACGAP1,SGO1,SPAG5
centrosome cycle	10	AURKA,BRCA1,BRCA2,CCNF,CCP110,CDK1,CDK2,CENPJ,CEP135,CEP152,CEP192,CEP76,CHEK1,CHORDC1,GEN1,KIF11,KIFC1,NDE1,NEK2,PLK4,RANBP1,RBM14,SASS6,STIL,TUBGCP3,TUBGCP4,WDR62
signal transduction by p53 class mediator	10	AURKA,AURKB,BARD1,BLM,BRCA1,BRCA2,BRIP1,CASP2,CCNB1,CDC25C,CDK1,CDK2,CDKN2A,CENPJ,CHEK1,DNA2,EXO1,FOXM1,GTSE1,MSH2,PAXIP1,PCNA,RBBP8,RFC2,RFC3,RFC4,RMI1,RPS6KA6,SSRP1,TAF5,TMEM109,TOPBP1,TPX2
tRNA export from nucleus	10	NDC1,NUP107,NUP153,NUP155,NUP160,NUP188,NUP205,NUP35,NUP43,NUP58,NUP85,NUP88
mitotic nuclear envelope disassembly	10	CCNB1,CCNB2,CDK1,NDC1,NUP107,NUP153,NUP155,NUP160,NUP188,NUP205,NUP35,NUP43,NUP58,NUP85,NUP88,PLK1,VRK1
organelle fission	10	ANAPC1,ANAPC15,ANLN,ASPM,AURKA,AURKB,BIRC5,BORA,BRCA2,BUB1,BUB1B,BUB3,CCNA2,CCNB1,CCNB2,CCNF,CDC20,CDC25A,CDC25C,CDC6,CDCA2,CDCA3,CDCA5,CDCA8,CDK1,CDK2,CENPE,CENPF,CENPH,CENPN,CENPW,CEP192,CEP55,CEP97,CHEK1,CIT,CKAP5,CKS2,DLGAP5,DMC1,DSCC1,DSN1,ERCC6L,ESPL1,FAM64A,FANCA,FANCD2,FANCM,FBXO5,GEN1,GSG2,HAUS4,HAUS8,HELLS,INCENP,ITGB3BP,KIF11,KIF14,KIF15,KIF18A,KIF18B,KIF20B,KIF22,KIF23,KIF2C,KIF4A,KIF4B,KIFC1,KNSTRN,KNTC1,MAD2L1,MASTL,MCMBP,MIS18A,MKI67,MLH1,MSH2,MSH6,MTBP,MTFR2,MYBL2,NCAPD2,NCAPD3,NCAPG,NCAPG2,NCAPH,NDC1,NDC80,NDE1,NEK2,NEK3,NOLC1,NUF2,NUP153,NUP43,NUP88,NUSAP1,PBK,PDE3A,PHB2,PKMYT1,PLK1,PRC1,PSRC1,PTTG1,RACGAP1,RAD51,RAD54B,RAD54L,RANBP1,RBBP8,RCC1,REEP4,RUVBL1,SGO1,SKA1,SKA2,SKA3,SLF1,SMC1A,SMC2,SMC3,SMC4,SPAG5,SPC24,SPC25,SPDL1,STIL,TIMELESS,TIPIN,TOP2A,TOPBP1,TPX2,TRIP13,TTK,TUBGCP3,TUBGCP4,UBE2C,UBE2S,VRK1,XRCC2,ZWILCH,ZWINT
cell cycle G2/M phase transition	10	AURKA,BIRC5,BLM,BORA,CCNA2,CCNB1,CCNB2,CCP110,CDC25A,CDC25C,CDC7,CDK1,CDK2,CDKN2A,CDKN2B,CENPF,CENPJ,CEP135,CEP152,CEP192,CEP76,CEP78,CHEK1,CIT,CKAP5,CLSPN,CNTRL,FANCI,FOXM1,GTSE1,HAUS4,HAUS8,HMMR,KIF14,LMNB1,MASTL,MELK,NDE1,NEK2,ODF2,PAXIP1,PKMYT1,PLK1,PLK4,TICRR,TPX2
mitotic DNA damage checkpoint	10	AURKA,BLM,CASP2,CCNA2,CCNB1,CDC25C,CDK1,CDK2,CENPJ,FANCI,GTSE1,MSH2,PCNA,RFWD3,TIPIN
negative regulation of chromosome organization	10	ACD,ANAPC15,ASF1A,ATAD2,BRCA1,BUB1,BUB1B,BUB3,CCNB1,CENPE,CENPF,CEP192,DNMT1,ESPL1,GEN1,MAD2L1,MTBP,PARP1,PLK1,PTTG1,STIL,TTK
regulation of cell cycle G1/S phase transition	10	AURKA,CASP2,CCNB1,CDC25C,CDC45,CDC6,CDK1,CDK2,CDKN2B,CENPJ,EZH2,GSG2,GTSE1,KIF14,MTBP,PCNA,PHB2,RFWD3,SUSD2
negative regulation of DNA recombination	10	BLM,MLH1,MSH2,MSH6,PARPBP,POLQ,RAD18
gene silencing	10	ASF1A,ATAD2,CDC45,CDK2,DNMT1,H2AFX,H2AFZ,HAT1,HELLS,HIST1H2AB,HIST1H2AC,HIST1H2AD,HIST1H2AE,HIST1H2AG,HIST1H2AH,HIST1H2AI,HIST1H2AJ,HIST1H2AK,HIST1H2AL,HIST1H2AM,HIST1H3A,HIST1H3B,HIST1H3C,HIST1H3F,HIST1H3G,HIST1H3H,HIST1H3I,HIST1H3J,HIST1H4A,HIST1H4B,HIST1H4C,HIST1H4D,HIST1H4E,HIST1H4F,HIST1H4I,HIST1H4J,HIST1H4K,HIST1H4L,HIST2H2AA3,HIST2H2AA4,HIST2H2AB,HIST2H2AC,HIST2H3A,HIST2H3C,HIST2H3D,NDC1,NUP107,NUP153,NUP155,NUP160,NUP188,NUP205,NUP35,NUP43,NUP58,NUP85,NUP88,SRRT,SUV39H1,TRIM28,UHRF1
mitotic metaphase plate congression	10	CCNB1,CDCA5,CDCA8,CENPE,CEP55,KIF14,KIF18A,KIF22,KIF2C,KIFC1,PSRC1,SPDL1
mitotic recombination	10	BLM,BRCA2,DMC1,DNA2,FEN1,GEN1,LIG1,MLH1,PCNA,POLA1,POLA2,POLD3,POLE,POLE2,PRIM1,RAD51,RAD54B,RFC2,RFC3,RFC4,TOP2A,XRCC2
chromosome separation	10	ANAPC15,BUB1,BUB1B,BUB3,CCNB1,CDC6,CENPE,CENPF,CEP192,CIT,DLGAP5,ESPL1,FANCM,GEN1,MAD2L1,MLH1,MTBP,NCAPD2,NCAPD3,PLK1,PTTG1,STIL,TOP2A,TTK,UBE2C
sister chromatid cohesion	10	AURKB,BIRC5,BUB1,BUB1B,BUB3,CDC20,CDCA5,CDCA8,CENPA,CENPE,CENPF,CENPH,CENPI,CENPK,CENPL,CENPM,CENPN,CENPO,CENPP,CENPQ,CENPU,CKAP5,CTCF,DDX11,DSCC1,DSN1,ERCC6L,ESPL1,GSG2,INCENP,ITGB3BP,KIF18A,KIF22,KIF2C,KNTC1,MAD2L1,MCMBP,NDC80,NDE1,NUF2,NUP107,NUP160,NUP43,NUP85,PHB2,PLK1,SGO1,SKA1,SKA2,SLF1,SMC1A,SMC3,SPC24,SPC25,SPDL1,ZWILCH,ZWINT
negative regulation of gene expression, epigenetic	10	ASF1A,ATAD2,CDC45,DNMT1,EZH2,H2AFX,H2AFZ,HAT1,HELLS,HIST1H2AB,HIST1H2AC,HIST1H2AD,HIST1H2AE,HIST1H2AG,HIST1H2AH,HIST1H2AI,HIST1H2AJ,HIST1H2AK,HIST1H2AL,HIST1H2AM,HIST1H3A,HIST1H3B,HIST1H3C,HIST1H3F,HIST1H3G,HIST1H3H,HIST1H3I,HIST1H3J,HIST1H4A,HIST1H4B,HIST1H4C,HIST1H4D,HIST1H4E,HIST1H4F,HIST1H4I,HIST1H4J,HIST1H4K,HIST1H4L,HIST2H2AA3,HIST2H2AA4,HIST2H2AB,HIST2H2AC,HIST2H3A,HIST2H3C,HIST2H3D,PHF19,SUV39H1,TRIM28,UHRF1
chromatin remodeling at centromere	10	CENPA,CENPH,CENPI,CENPK,CENPL,CENPM,CENPN,CENPO,CENPP,CENPQ,CENPU,CENPW,HELLS,HIST1H4A,HIST1H4B,HIST1H4C,HIST1H4D,HIST1H4E,HIST1H4F,HIST1H4I,HIST1H4J,HIST1H4K,HIST1H4L,HJURP,ITGB3BP,MIS18A,RUVBL1
mitotic sister chromatid segregation	10	ANAPC15,AURKB,BUB1,BUB1B,BUB3,CCNB1,CDC6,CDCA5,CDCA8,CENPE,CENPF,CEP192,CIT,DLGAP5,DSCC1,ESPL1,GEN1,GSG2,KIF14,KIF18A,KIF18B,KIF22,KIF23,KIF2C,KIF4A,KIF4B,KIFC1,KNSTRN,MAD2L1,MTBP,NCAPD2,NCAPD3,NCAPG,NCAPH,NDC80,NEK2,NUSAP1,PLK1,PRC1,PSRC1,PTTG1,RACGAP1,SLF1,SMC1A,SMC2,SMC3,SMC4,SPAG5,SPDL1,STIL,TTK,UBE2C,ZWINT
regulation of mitotic metaphase/anaphase transition	10	ANAPC15,BUB1,BUB1B,BUB3,CCNB1,CDC6,CENPE,CENPF,CEP192,DLGAP5,ESPL1,GEN1,MAD2L1,MTBP,PLK1,STIL,TTK,UBE2C
regulation of metaphase/anaphase transition of cell cycle	10	ANAPC15,BUB1,BUB1B,BUB3,CCNB1,CDC6,CENPE,CENPF,CEP192,DLGAP5,ESPL1,GEN1,MAD2L1,MTBP,PLK1,STIL,TTK,UBE2C
recombinational repair	10	BLM,BRCA1,BRCA2,CDC45,CDC7,CHEK1,DMC1,FEN1,FIGNL1,GEN1,GINS2,GINS4,H2AFX,MCM8,MMS22L,PARP1,PARPBP,POLQ,RAD51,RAD51AP1,RAD54B,RAD54L,RBBP8,XRCC2
regulation of megakaryocyte differentiation	10	HIST1H4A,HIST1H4B,HIST1H4C,HIST1H4D,HIST1H4E,HIST1H4F,HIST1H4I,HIST1H4J,HIST1H4K,HIST1H4L,HMGB2
mitotic sister chromatid separation	10	ANAPC15,BUB1,BUB1B,BUB3,CCNB1,CDC6,CENPE,CENPF,CEP192,CIT,DLGAP5,ESPL1,GEN1,MAD2L1,MTBP,PLK1,PTTG1,STIL,TTK,UBE2C
regulation of cytoskeleton organization	10	AURKA,BORA,BRCA1,CCNB1,CCNF,CDC42EP3,CENPE,CENPJ,CEP192,CEP76,CEP97,CHEK1,CHORDC1,CIT,CKAP2,CLIP3,ECT2,GEN1,GSN,HSPH1,ID1,KIF11,KIF18A,KIFC1,KNSTRN,NEK2,PARP3,PLK1,PLK4,PSRC1,RACGAP1,RANBP1,RBM14,SDC4,SKA1,SKA2,SKA3,SPAG5,STMN1,TPX2
cell cycle process	10	ANAPC1,ANAPC15,ANLN,ASPM,AUNIP,AURKA,AURKB,BARD1,BIRC5,BLM,BORA,BRCA1,BRCA2,BRIP1,BUB1,BUB1B,BUB3,CASP2,CCNA2,CCNB1,CCNB2,CCNE2,CCNF,CCP110,CDC20,CDC25A,CDC25C,CDC45,CDC6,CDC7,CDCA2,CDCA3,CDCA5,CDCA8,CDK1,CDK2,CDKN2A,CDKN2B,CDKN2C,CDKN3,CDT1,CENPA,CENPE,CENPF,CENPH,CENPI,CENPJ,CENPK,CENPL,CENPM,CENPN,CENPO,CENPP,CENPQ,CENPU,CENPW,CEP135,CEP152,CEP192,CEP55,CEP76,CEP78,CEP97,CHEK1,CHORDC1,CIT,CKAP2,CKAP5,CKS1B,CKS2,CLSPN,CNTRL,CTCF,DCLRE1B,DDIAS,DDX11,DHFR,DHFRP1,DLGAP5,DMC1,DNA2,DSCC1,DSN1,DTL,E2F8,ECT2,ERCC6L,ESPL1,EZH2,FAM64A,FANCA,FANCD2,FANCG,FANCI,FANCM,FBXO5,FOXM1,GAS2L3,GEN1,GINS1,GINS2,GPSM2,GSG2,GTSE1,H2AFX,HAUS4,HAUS8,HELLS,HMMR,INCENP,IQGAP3,ITGB3BP,KIF11,KIF14,KIF15,KIF18A,KIF18B,KIF20A,KIF20B,KIF22,KIF23,KIF2C,KIF4A,KIF4B,KIFC1,KNSTRN,KNTC1,LIG1,LMNB1,MAD2L1,MASTL,MCM10,MCM2,MCM3,MCM4,MCM5,MCM6,MCM7,MCM8,MCMBP,MELK,MIS18A,MKI67,MLH1,MSH2,MSH6,MTBP,MYBL2,NCAPD2,NCAPD3,NCAPG,NCAPG2,NCAPH,NDC1,NDC80,NDE1,NEK2,NEK3,NOLC1,NOTCH2,NUF2,NUP107,NUP153,NUP155,NUP160,NUP188,NUP205,NUP35,NUP43,NUP58,NUP85,NUP88,NUSAP1,ODF2,ORC1,ORC5,ORC6,PARP3,PAXIP1,PBK,PCNA,PDE3A,PHB2,PKMYT1,PLK1,PLK4,POLA1,POLA2,POLE,POLE2,PRC1,PRIM1,PSRC1,PTTG1,RACGAP1,RAD51,RAD54B,RAD54L,RANBP1,RBBP8,RBM14,RCC1,REEP4,RFWD3,RRM2,RUVBL1,SASS6,SGO1,SKA1,

		SKA2,SKA3,SLF1,SMC1A,SMC2,SMC3,SMC4,SPAG5,SPC24,SPC25,SPDL1,STIL,STMN1,SUSD2,TACC3,TCF19,TICRR,TIMELESS,TIPIN,TOP2A,TOPBP1,TPX2,TRIP13,TTK,TUBGCP3,TUBGCP4,TYMS,UBE2C,UBE2S,VRK1,WDR62,XRCC2,ZWILCH,ZWINT
negative regulation of nucleic acid-templated transcription	10	ASF1A,ATAD2,ATOH8,AURKB,BIRC5,BRCA1,CASP8AP2,CDC45,CDKN2A,CENPF,CTCF,DEPDC1,DNMT1,E2F8,EZH2,FOXM1,GMNN,H2AFX,H2AFZ,HAT1,HELLS,HIST1H1B,HIST1H1C,HIST1H1D,HIST1H1E,HIST1H2AB,HIST1H2AC,HIST1H2AD,HIST1H2AE,HIST1H2AG,HIST1H2AH,HIST1H2AI,HIST1H2AJ,HIST1H2AK,HIST1H2AL,HIST1H2AM,HIST1H3A,HIST1H3B,HIST1H3C,HIST1H3F,HIST1H3G,HIST1H3H,HIST1H3I,HIST1H3J,HIST1H4A,HIST1H4B,HIST1H4C,HIST1H4D,HIST1H4E,HIST1H4F,HIST1H4I,HIST1H4J,HIST1H4K,HIST1H4L,HIST2H2AA3,HIST2H2AA4,HIST2H2AB,HIST2H2AC,HIST2H3A,HIST2H3C,HIST2H3D,HMGB1,HMGB2,HMGN2,ID1,LRRFIP1,MTERF3,NOTCH2,PARP1,PCNA,PHB2,PHF19,PLK1,PRDM1,RBBP8,RBL1,SUV39H1,SUV39H2,TIMELESS,TRIM28,UHRF1,ZNF85,ZNF93
metaphase/anaphase transition of mitotic cell cycle	10	ANAPC15,BUB1,BUB1B,BUB3,CCNB1,CDC6,CENPE,CENPF,CEP192,CIT,DLGAP5,ESPL1,GEN1,MAD2L1,MTBP,PLK1,STIL,TTK,UBE2C
multi-organism transport	10	ALYREF,KPNA2,NDC1,NUP107,NUP153,NUP155,NUP160,NUP188,NUP205,NUP35,NUP43,NUP58,NUP85,NUP88,TCP1,THOC3
anatomical structure homeostasis	10	ACD,AURKB,BARD1,BLM,BRCA2,DCLRE1B,DCLRE1C,DNA2,FEN1,HIST1H4A,HIST1H4B,HIST1H4C,HIST1H4D,HIST1H4E,HIST1H4F,HIST1H4I,HIST1H4J,HIST1H4K,HIST1H4L,LIG1,NEK2,PARP1,PARP3,PCNA,POLA1,POLA2,POLD3,POLE,POLE2,PRIM1,RAD51,RFC2,RFC3,RFC4,TCP1
regulation of cell cycle process	10	ANAPC1,ANAPC15,ANLN,AURKA,AURKB,BIRC5,BLM,BORA,BRCA1,BRCA2,BUB1,BUB1B,BUB3,CASP2,CCNA2,CCNB1,CCNF,CCP110,CDC20,CDC25C,CDC45,CDC6,CDC7,CDCA5,CDK1,CDK2,CDKN2A,CDKN2B,CDT1,CENPE,CENPF,CENPJ,CEP192,CEP76,CEP97,CHEK1,CHORDC1,CIT,CLSPN,CTCF,DLGAP5,E2F8,ECT2,ESPL1,EZH2,FANCI,FBXO5,FOXM1,GEN1,GSG2,GTSE1,KIF11,KIF14,KIF20B,KIF23,KIFC1,KNSTRN,KNTC1,LMNB1,MAD2L1,MSH2,MTBP,NEK2,NUSAP1,ORC1,PARP3,PAXIP1,PCNA,PDE3A,PHB2,PKMYT1,PLK1,PLK4,PSRC1,PTTG1,RACGAP1,RANBP1,RBM14,RCC1,RFWD3,SLF1,SMC1A,SMC3,SPAG5,STIL,SUSD2,TICRR,TIPIN,TPX2,TTK,UBE2C
negative regulation of metaphase/anaphase transition of cell cycle	10	ANAPC15,BUB1,BUB1B,BUB3,CCNB1,CENPE,CENPF,CEP192,GEN1,MAD2L1,MTBP,PLK1,STIL,TTK
transport of virus	10	ALYREF,KPNA2,NDC1,NUP107,NUP153,NUP155,NUP160,NUP188,NUP205,NUP35,NUP43,NUP58,NUP85,NUP88,THOC3
mitotic spindle assembly	10	AURKB,BIRC5,CEP97,KIF11,KIF23,KIF4A,KIF4B,KIFC1,MYBL2,NEK2,PLK1,RACGAP1,TPX2,TUBGCP3,TUBGCP4
telomere capping	10	ACD,AURKB,DCLRE1B,DCLRE1C,HIST1H4A,HIST1H4B,HIST1H4C,HIST1H4D,HIST1H4E,HIST1H4F,HIST1H4I,HIST1H4J,HIST1H4K,HIST1H4L,NEK2
protein localization to chromosome, centromeric region	10	AURKB,BUB1B,CDK1,CENPA,GSG2,MTBP,SPDL1
regulation of transcription involved in G1/S transition of mitotic cell cycle	10	CDC45,CDC6,CDK1,CDT1,DHFR,DHFRP1,FBXO5,ORC1,PCNA,POLA1,RRM2,TYMS
multi-organism intracellular transport	10	ALYREF,KPNA2,NDC1,NUP107,NUP153,NUP155,NUP160,NUP188,NUP205,NUP35,NUP43,NUP58,NUP85,NUP88,THOC3
regulation of response to DNA damage stimulus	10	BRCA1,CDKN2A,CHEK1,FIGNL1,FOXM1,H2AFX,HMGB1,PARP1,PARPBP,PAXIP1,PCNA,POLQ,RAD51,RAD51AP1,RFWD3,SLF1,TPT1,TRIM28,USP1
mitotic nuclear division	10	ANAPC1,ANAPC15,ANLN,ASPM,AURKA,AURKB,BIRC5,BORA,BUB1,BUB1B,BUB3,CCNA2,CCNB1,CCNB2,CCNF,CDC20,CDC25A,CDC25C,CDC6,CDCA2,CDCA3,CDCA5,CDCA8,CDK1,CDK2,CENPE,CENPF,CENPH,CENPN,CENPW,CEP192,CEP55,CEP97,CHEK1,CIT,CKAP5,DLGAP5,DSCC1,DSN1,ERCC6L,ESPL1,FAM64A,FBXO5,GEN1,GSG2,HAUS4,HAUS8,HELLS,INCENP,ITGB3BP,KIF11,KIF14,KIF15,KIF18A,KIF18B,KIF20B,KIF22,KIF23,KIF2C,KIF4A,KIF4B,KIFC1,KNSTRN,KNTC1,MAD2L1,MASTL,MCMBP,MIS18A,MTBP,MYBL2,NCAPD2,NCAPD3,NCAPG,NCAPG2,NCAPH,NDC80,NDE1,NEK2,NEK3,NOLC1,NUF2,NUP153,NUP43,NUP88,NUSAP1,PBK,PHB2,PKMYT1,PLK1,PRC1,PSRC1,PTTG1,RACGAP1,RANBP1,RBBP8,RCC1,REEP4,RUVBL1,SGO1,SKA1,SKA2,SKA3,SLF1,SMC1A,SMC2,SMC3,SMC4,SPAG5,SPC24,SPC25,SPDL1,STIL,TIMELESS,TIPIN,TPX2,TTK,TUBGCP3,TUBGCP4,UBE2C,UBE2S,VRK1,ZWILCH,ZWINT
negative regulation of organelle organization	10	ACD,ANAPC15,ASF1A,ATAD2,BRCA1,BUB1,BUB1B,BUB3,CCNB1,CCNF,CCP110,CENPE,CENPF,CEP192,CEP97,CHEK1,CKAP2,CLIP3,DNMT1,ESPL1,FBXO5,GEN1,GSN,KIFC1,MAD2L1,MSH2,MTBP,NEK2,PARP1,PLK1,PTTG1,RBM14,STIL,STMN1,TTK,VAT1
histone exchange	10	ANP32E,CENPA,CENPH,CENPI,CENPK,CENPL,CENPM,CENPN,CENPO,CENPP,CENPQ,CENPU,CENPW,HIST1H4A,HIST1H4B,HIST1H4C,HIST1H4D,HIST1H4E,HIST1H4F,HIST1H4I,HIST1H4J,HIST1H4K,HIST1H4L,HJURP,ITGB3BP,MIS18A,NASP,RUVBL1
mitotic DNA integrity checkpoint	10	AURKA,BLM,CASP2,CCNA2,CCNB1,CDC25C,CDK1,CDK2,CENPJ,CLSPN,FANCI,GTSE1,MSH2,PCNA,RFWD3,TICRR,TIPIN,TOP2A
double-strand break repair	10	BARD1,BLM,BRCA1,BRCA2,BRIP1,CDC45,CDC7,CDCA5,CHEK1,DCLRE1B,DCLRE1C,DMC1,DNA2,ESCO2,FEN1,FIGNL1,FOXM1,GEN1,GINS2,GINS4,H2AFX,HIST1H4A,HIST1H4B,HIST1H4C,HIST1H4D,HIST1H4E,HIST1H4F,HIST1H4I,HIST1H4J,HIST1H4K,HIST1H4L,LIG1,MCM8,MLH1,MMS22L,MSH2,PARP1,PARP3,PARPBP,PAXIP1,POLA1,POLQ,RAD51,RAD51AP1,RAD54B,RAD54L,RBBP8,SLF1,TDP1,TRIP13,XRCC2
negative regulation of hematopoietic progenitor cell differentiation	10	HIST1H4A,HIST1H4B,HIST1H4C,HIST1H4D,HIST1H4E,HIST1H4F,HIST1H4I,HIST1H4J,HIST1H4K,HIST1H4L
G1/S transition of mitotic cell cycle	10	AURKA,CASP2,CCNB1,CCNE2,CDC25A,CDC25C,CDC45,CDC6,CDC7,CDCA5,CDK1,CDK2,CDKN2A,CDKN2B,CDKN2C,CDKN3,CDT1,CENPJ,DHFR,DHFRP1,EZH2,FBXO5,GSG2,GTSE1,IQGAP3,KIF14,MCM10,MCM2,MCM3,MCM4,MCM5,MCM6,MCM7,MCM8,MTBP,ORC1,ORC5,ORC6,PCNA,PKMYT1,POLA1,POLA2,POLE,POLE2,PRIM1,RANBP1,RBBP8,RCC1,RFWD3,RRM2,TCF19,TYMS
gene silencing by RNA	10	HIST1H3A,HIST1H3B,HIST1H3C,HIST1H3F,HIST1H3G,HIST1H3H,HIST1H3I,HIST1H3J,HIST1H4A,HIST1H4B,HIST1H4C,HIST1H4D,HIST1H4E,HIST1H4F,HIST1H4I,HIST1H4J,HIST1H4K,HIST1H4L,HIST2H3A,HIST2H3C,HIST2H3D,NDC1,NUP107,NUP153,NUP155,NUP160,NUP188,NUP205,NUP35,NUP43,NUP58,NUP85,NUP88,SRRT
protein heterooligomerization	10	C1QTNF2,HIST1H3A,HIST1H3B,HIST1H3C,HIST1H3F,HIST1H3G,HIST1H3H,HIST1H3I,HIST1H3J,HIST1H4A,HIST1H4B,HIST1H4C,HIST1H4D,HIST1H4E,HIST1H4F,HIST1H4I,HIST1H4J,HIST1H4K,HIST1H4L,HIST2H3D,NUP58,RRM1,RRM2
DNA synthesis involved in DNA repair	10	BARD1,BLM,BRCA1,BRCA2,BRIP1,DNA2,DTL,EXO1,PCNA,POLA1,POLD3,POLE,POLE2,RAD51,RAD51AP1,RBBP8,RFC2,RFC3,RFC4,RMI1,XRCC2
regulation of DNA metabolic process	10	ACD,ALYREF,APAF1,AURKB,BLM,BRCA1,BRCA2,CACYBP,CDC6,CDC7,CDK1,CDK2,CDKN2A,CDT1,CHEK1,DNA2,DNMT1,DSCC1,E2F8,ESCO2,FIGNL1,FOXM1,GMNN,H2AFX,HMGB1,KPNA2,MIS18A,MLH1,MSH2,MSH6,NEK2,PARP1,PARP3,PARPBP,PAXIP1,PCNA,POLQ,RAD18,RAD51,RAD51AP1,RFC2,RFC3,RFC4,SLF1,SMC1A,SMC3,TCP1,TICRR,TIPIN,TOP2A,TRIM28,UNG,USP1,ZNF93
CENP-A containing chromatin organization	10	CENPA,CENPH,CENPI,CENPK,CENPL,CENPM,CENPN,CENPO,CENPP,CENPQ,CENPU,CENPW,HIST1H4A,HIST1H4B,HIST1H4C,HIST1H4D,HIST1H4E,HIST1H4F,HIST1H4I,HIST1H4J,HIST1H4K,HIST1H4L,HJURP,ITGB3BP,MIS18A,RUVBL1
negative regulation of mitotic sister	10	ANAPC15,BUB1,BUB1B,BUB3,CCNB1,CENPE,CENPF,CEP192,GEN1,MAD2L1,MTBP,PLK1,PTTG1,STIL,TTK

chromatid segregation		
nuclear envelope organization	10	CCNB1,CCNB2,CDK1,NDC1,NUP107,NUP153,NUP155,NUP160,NUP188,NUP205,NUP35,NUP43,NUP58,NUP85,NUP88,PLK1,REEP4,VRK1
regulation of DNA replication	10	ACD,AURKB,BLM,BRCA2,CACYBP,CDC6,CDC7,CDK1,CDK2,CDT1,DNA2,DSCC1,E2F8,ESCO2,GMNN,NEK2,PCNA,RFC2,RFC3,RFC4,SMC1A,SMC3,TCP1,TICRR,TIPIN
positive regulation of cell cycle process	10	AURKA,AURKB,BIRC5,BRCA1,CASP2,CCNB1,CDC25C,CDC45,CDC6,CDC7,CDCA5,CDK1,CDK2,CDKN2A,CENPE,CENPJ,CIT,DLGAP5,E2F8,ECT2,ESPL1,FBXO5,GEN1,GTSE1,KIF14,KIF20B,KIF23,MAD2L1,MTBP,NUSAP1,ORC1,PCNA,PHB2,PLK4,RACGAP1,RANBP1,SLF1,UBE2C
DNA duplex unwinding	10	BLM,BRIP1,CDC45,DDX11,DDX12P,DNA2,GINS1,GINS2,GINS4,MCM2,MCM3,MCM4,MCM5,MCM6,MCM7,PARP1,RAD51,RAD54B,RUVBL1,TOP2A
DNA metabolic process	10	ACD,ACTL6A,ALYREF,APAF1,ASF1A,AURKB,BARD1,BLM,BRCA1,BRCA2,BRIP1,CACYBP,CCNE2,CDC25A,CDC25C,CDC45,CDC6,CDC7,CDCA5,CDK1,CDK2,CDKN2A,CDT1,CENPF,CHAF1A,CHAF1B,CHEK1,CLSPN,CTCF,DCLRE1B,DCLRE1C,DMC1,DNA2,DNMT1,DSCC1,DTL,DUT,E2F8,EME1,ESCO2,EXO1,EZH2,FAM111A,FANCA,FANCB,FANCC,FANCD2,FANCG,FANCI,FANCM,FEN1,FIGNL1,FOXM1,GEN1,GINS1,GINS2,GINS3,GINS4,GMNN,H2AFX,HELLS,HIST1H4A,HIST1H4B,HIST1H4C,HIST1H4D,HIST1H4E,HIST1H4F,HIST1H4I,HIST1H4J,HIST1H4K,HIST1H4L,HMGB1,HMGB2,KIF22,KPNA2,LIG1,LIN9,MCM10,MCM2,MCM3,MCM4,MCM5,MCM6,MCM7,MCM8,MCMBP,MGME1,MIS18A,MKI67,MLH1,MMS22L,MND1,MSH2,MSH6,NASP,NEIL3,NEK2,ORC1,ORC5,ORC6,PARP1,PARP2,PARP3,PARPBP,PAXIP1,PCNA,POLA1,POLA2,POLD3,POLE,POLE2,POLE3,POLQ,PRIM1,PSMC3IP,PTTG1,RAD18,RAD51,RAD51AP1,RAD54B,RAD54L,RBBP8,RBM14,RFC2,RFC3,RFC4,RFWD3,RMI1,RNASEH2A,RRM1,RRM2,RUVBL1,SLF1,SMC1A,SMC3,SSRP1,TCP1,TDP1,TFAM,TICRR,TIMELESS,TIPIN,TK1,TOP2A,TOPBP1,TRIM28,TRIP13,TYMS,UBE2T,UHRF1,UNG,USP1,XRCC2,ZNF93
spindle organization	10	ASPM,AUNIP,AURKA,AURKB,BIRC5,BORA,CCNB1,CENPJ,CEP192,CEP97,CKAP5,ESPL1,FBXO5,GPSM2,HAUS4,HAUS8,KIF11,KIF23,KIF4A,KIF4B,KIFC1,KNSTRN,MLH1,MYBL2,NDC80,NEK2,PARP3,PLK1,PRC1,PSRC1,RACGAP1,RANBP1,RCC1,SMC1A,SMC3,SPAG5,SPC25,STIL,STMN1,TACC3,TPX2,TTK,TUBGCP3,TUBGCP4,WDR62
regulation of gene expression, epigenetic	10	ASF1A,ATAD2,BRCA1,CDC45,CHEK1,CTCF,DNMT1,EZH2,H2AFX,H2AFZ,HAT1,HELLS,HIST1H2AB,HIST1H2AC,HIST1H2AD,HIST1H2AE,HIST1H2AG,HIST1H2AH,HIST1H2AI,HIST1H2AJ,HIST1H2AK,HIST1H2AL,HIST1H2AM,HIST1H3A,HIST1H3B,HIST1H3C,HIST1H3F,HIST1H3G,HIST1H3H,HIST1H3I,HIST1H3J,HIST1H4A,HIST1H4B,HIST1H4C,HIST1H4D,HIST1H4E,HIST1H4F,HIST1H4I,HIST1H4J,HIST1H4K,HIST1H4L,HIST2H2AA3,HIST2H2AA4,HIST2H2AB,HIST2H2AC,HIST2H3A,HIST2H3C,HIST2H3D,PHF19,SMCHD1,SRRT,SUV39H1,TRIM28,UHRF1
regulation of cell cycle G2/M phase transition	10	BLM,CCNA2,CDC7,CDK1,CDKN2A,CENPF,CHEK1,CLSPN,FANCI,GTSE1,KIF14,LMNB1,PAXIP1,PLK1,TICRR
regulation of microtubule-based process	10	AURKA,BORA,BRCA1,CCNB1,CCNF,CENPE,CENPJ,CEP192,CEP76,CEP97,CFAP20,CHEK1,CHORDC1,CKAP2,CLIP3,ECT2,GEN1,HSPH1,KIF11,KIF18A,KIFC1,KNSTRN,NEK2,PARP3,PLK1,PLK4,PSRC1,RACGAP1,RANBP1,RBM14,SKA1,SKA2,SKA3,SPAG5,STMN1,TACC3,TPX2
regulation of hematopoietic progenitor cell differentiation	10	HIST1H4A,HIST1H4B,HIST1H4C,HIST1H4D,HIST1H4E,HIST1H4F,HIST1H4I,HIST1H4J,HIST1H4K,HIST1H4L,HMGB2
DNA unwinding involved in DNA replication	10	MCM2,MCM4,MCM6,MCM7,RAD51,TOP2A
lagging strand elongation	10	DNA2,FEN1,LIG1,PARP1,PARP2,PARP3,POLA1,RNASEH2A
DNA-dependent DNA replication maintenance of fidelity	10	ALYREF,BLM,BRCA2,FANCM,MMS22L,PCNA,RAD51,TIPIN
chromatin silencing	10	ASF1A,ATAD2,CDC45,DNMT1,H2AFX,H2AFZ,HAT1,HELLS,HIST1H2AB,HIST1H2AC,HIST1H2AD,HIST1H2AE,HIST1H2AG,HIST1H2AH,HIST1H2AI,HIST1H2AJ,HIST1H2AK,HIST1H2AL,HIST1H2AM,HIST1H3A,HIST1H3B,HIST1H3C,HIST1H3F,HIST1H3G,HIST1H3H,HIST1H3I,HIST1H3J,HIST1H4A,HIST1H4B,HIST1H4C,HIST1H4D,HIST1H4E,HIST1H4F,HIST1H4I,HIST1H4J,HIST1H4K,HIST1H4L,HIST2H2AA3,HIST2H2AA4,HIST2H2AB,HIST2H2AC,HIST2H3A,HIST2H3C,HIST2H3D,SUV39H1,TRIM28,UHRF1
spindle elongation	10	AURKB,KIF23,KIF4A,KIF4B,PRC1,RACGAP1
establishment of chromosome localization	10	BIRC5,BRCA2,CCNB1,CDCA5,CDCA8,CENPE,CENPF,CEP55,DLGAP5,KIF14,KIF18A,KIF22,KIF2C,KIFC1,MLH1,NDE1,PSRC1,SPDL1
DNA repair	10	ACTL6A,ASF1A,BARD1,BLM,BRCA1,BRCA2,BRIP1,CDC45,CDC7,CDCA5,CDK1,CDK2,CHAF1A,CHAF1B,CHEK1,CLSPN,DCLRE1B,DCLRE1C,DMC1,DNA2,DTL,EME1,ESCO2,EXO1,FANCA,FANCB,FANCC,FANCD2,FANCG,FANCI,FANCM,FEN1,FIGNL1,FOXM1,GEN1,GINS2,GINS4,H2AFX,HIST1H4A,HIST1H4B,HIST1H4C,HIST1H4D,HIST1H4E,HIST1H4F,HIST1H4I,HIST1H4J,HIST1H4K,HIST1H4L,HMGB1,HMGB2,KIF22,LIG1,MCM8,MGME1,MLH1,MMS22L,MSH2,MSH6,NEIL3,PARP1,PARP2,PARP3,PARPBP,PAXIP1,PCNA,POLA1,POLD3,POLE,POLE2,POLQ,PTTG1,RAD18,RAD51,RAD51AP1,RAD54B,RAD54L,RBBP8,RBM14,RFC2,RFC3,RFC4,RFWD3,RMI1,RNASEH2A,RUVBL1,SLF1,SMC1A,SMC3,SSRP1,TDP1,TICRR,TOPBP1,TRIM28,TRIP13,UBE2T,UHRF1,UNG,USP1,XRCC2
centriole replication	10	BRCA1,CCP110,CDK2,CENPJ,CEP135,CEP152,CEP192,CEP76,PLK4,RBM14,SASS6,WDR62
microtubule-based process	10	ASPM,AUNIP,AURKA,AURKB,BIRC5,BORA,BRCA1,BRCA2,CCNB1,CCNF,CCP110,CDK1,CDK2,CENPA,CENPE,CENPJ,CEP135,CEP152,CEP192,CEP76,CEP97,CFAP20,CHEK1,CHORDC1,CKAP2,CKAP5,CLIP3,DLGAP5,ECT2,ESPL1,FBXO5,FIGNL1,GAS2L3,GEN1,GPSM2,GTSE1,HAUS4,HAUS8,HSPB11,HSPH1,KIF11,KIF14,KIF15,KIF18A,KIF18B,KIF20A,KIF20B,KIF22,KIF23,KIF24,KIF2C,KIF4A,KIF4B,KIFC1,KNSTRN,MLH1,MYBL2,NDC1,NDC80,NDE1,NEK2,NEK3,NUSAP1,PARP3,PLK1,PLK4,PRC1,PSRC1,RACGAP1,RANBP1,RBM14,RCC1,SASS6,SGO1,SKA1,SKA2,SKA3,SMC1A,SMC3,SPAG5,SPC25,SPDL1,STIL,STMN1,TACC3,TPX2,TTK,TUBD1,TUBGCP3,TUBGCP4,WDR62,XRCC2
regulation of sister chromatid segregation	10	ANAPC15,BUB1,BUB1B,BUB3,CCNB1,CDC6,CDCA5,CENPE,CENPF,CEP192,CIT,CTCF,DLGAP5,ESPL1,GEN1,MAD2L1,MTBP,PLK1,PTTG1,SLF1,STIL,TTK,UBE2C
negative regulation of mitotic metaphase/anaphase transition	10	ANAPC15,BUB1,BUB1B,BUB3,CCNB1,CENPE,CENPF,CEP192,GEN1,MAD2L1,MTBP,PLK1,STIL,TTK
centrosome organization	10	AURKA,BRCA1,BRCA2,CCNF,CCP110,CDK1,CDK2,CENPJ,CEP135,CEP152,CEP192,CEP76,CHEK1,CHORDC1,CKAP5,GEN1,HAUS4,HAUS8,KIF11,KIFC1,NDE1,NEK2,PLK1,PLK4,RANBP1,RBM14,SASS6,SGO1,STIL,TUBGCP3,TUBGCP4,WDR62,XRCC2
meiosis I	10	AURKA,BRCA2,CKS2,DMC1,ESPL1,FANCD2,FANCM,FBXO5,MLH1,MSH2,MSH6,NDC1,PLK1,PTTG1,RAD51,RAD54B,TOP2A,TOPBP1,TRIP13,XRCC2
spindle assembly	10	ASPM,AURKA,AURKB,BIRC5,CEP192,CEP97,FBXO5,HAUS4,HAUS8,KIF11,KIF23,KIF4A,KIF4B,KIFC1,MLH1,MYBL2,NEK2,PLK1,RACGAP1,RCC1,TPX2,TUBGCP3,TUBGCP4
ribonucleoprotein complex export from nucleus	10	ALYREF,BUD13,DDX39A,GLE1,NDC1,NUP107,NUP153,NUP155,NUP160,NUP188,NUP205,NUP35,NUP43,NUP58,NUP85,NUP88,SRSF7,THOC3
cell division	10	ANAPC1,ANAPC15,ANLN,ASPM,AURKA,AURKB,BIRC5,BLM,BORA,BRCA2,BUB1,BUB1B,BUB3,CCNA2,CCNB1,CCNB2,CCNE2,CCNF,CCP110,CDC20,CDC25A,CDC25C,CDC6,CDC7,CDCA2,CDCA3,CDCA5,CDCA8,CDK1,CDK2,CDKN2A,CENPE,CENPF,CENPJ,CENPW,CEP192,CEP55,CEP97,CHEK1,CIT,CKAP2,CKAP5,CKS1B,CKS2,CNTRL,DLGAP5,DSN1,E2F8,ECT2,ERCC6L,ESPL1,FAM64A,FBXO5,GEN1,HAUS4,HAUS8,HELLS,INCENP,ITGB3BP,KIF11,KIF14,KIF18B,KIF20A,KIF20B,KIF23,KIF2C,KIF4A,KIF4B,KIFC1,KNSTRN,KNTC1,LIG1,MAD2L1,MASTL,MCMBP,MIS18A,MSH2,MTBP,NCAPD2,NCAPD3,NCAPG,NCAPG2,NCAPH,NDC80,NDE1,NEK2,NEK3,NUF2,NUP43,NUSAP1,PDE3A,PHB2,PKMYT1,PLK1,PRC1,PSRC1,PTTG1,RACGAP1,RANBP1,RBBP8,RCC1,REEP4,RUVBL1,SGO1,SKA1,SKA2,SKA3,SLF1,SMC1A,SMC2,SMC3,SMC4,SPAG5,SPC24,SPC25,SPDL1,STIL,STMN1,SUSD2,TIMELESS,TIPIN,TOP2A,TPX2,TTK,UBE2C,UBE2S,VRK1,ZWILCH,ZWINT
negative regulation of	10	ACD,ASF1A,ATAD2,ATOH8,AURKB,BIRC5,BLM,BRCA1,BRCA2,CDC45,CDC6,CDKN2A,CENPF,CHEK1,CTCF,DEPDC1,DNMT1,E2F8,EZH2,FOXM1,GMNN,H2AFX,H2AFZ,HAT1,HELLS,HIST1H1B,HIST1H1C,HIST1H1D,HIST1H1E,HIST1H2AB,HIST1H2AC,HIST1H2AD,HIST1H2AE,HIST1H2AG,

cellular macromolecule biosynthetic process		HIST1H2AH,HIST1H2AI,HIST1H2AJ,HIST1H2AK,HIST1H2AL,HIST1H2AM,HIST1H3A,HIST1H3B,HIST1H3C,HIST1H3F,HIST1H3G,HIST1H3H,HIST1H3I,HIST1H3J,HIST1H4A,HIST1H4B,HIST1H4C,HIST1H4D,HIST1H4E,HIST1H4F,HIST1H4I,HIST1H4J,HIST1H4K,HIST1H4L,HIST2H2AA3,HIST2H2AA4,HIST2H2AB,HIST2H2AC,HIST2H3A,HIST2H3C,HIST2H3D,HMGB1,HMGB2,HMGN2,ID1,LRRFIP1,MTERF3,NOTCH2,PARP1,PCNA,PHB2,PHF19,PLK1,PRDM1,RBBP8,RBL1,SMC1A,SMC3,SRRT,SUV39H1,SUV39H2,TIMELESS,TIPIN,TRIM28,UHRF1,ZNF93
cell cycle G1/S phase transition	10	AURKA,CASP2,CCNB1,CCNE2,CDC25A,CDC25C,CDC45,CDC6,CDC7,CDCA5,CDK1,CDK2,CDKN2A,CDKN2B,CDKN2C,CDKN3,CDT1,CENPJ,DHFR,DHFRP1,EZH2,FBXO5,GSG2,GTSE1,IQGAP3,KIF14,MCM10,MCM2,MCM3,MCM4,MCM5,MCM6,MCM7,MCM8,MTBP,ORC1,ORC5,ORC6,PCNA,PHB2,PKMYT1,POLA1,POLA2,POLE,POLE2,PRIM1,RANBP1,RBBP8,RCC1,RFWD3,RRM2,SUSD2,TCF19,TYMS
RNA transport	10	ALYREF,BUD13,CKAP5,DDX39A,GLE1,HNRNPA3,NDC1,NUP107,NUP153,NUP155,NUP160,NUP188,NUP205,NUP35,NUP43,NUP58,NUP85,NUP88,RANBP1,SRSF7,THOC3
regulation of G2/M transition of mitotic cell cycle	10	BLM,CCNA2,CDC7,CDK1,CDKN2A,CENPF,CHEK1,CLSPN,FANCI,KIF14,LMNB1,TICRR
reciprocal meiotic recombination	10	DMC1,FANCM,MLH1,MSH2,MSH6,RAD51,RAD54B,TOP2A,TOPBP1,TRIP13,XRCC2
negative regulation of transcription, DNA-templated	10	ASF1A,ATAD2,ATOH8,AURKB,BIRC5,BRCA1,CDC45,CDKN2A,CENPF,CTCF,DEPDC1,DNMT1,E2F8,EZH2,FOXM1,GMNN,H2AFX,H2AFZ,HAT1,HELLS,HIST1H1B,HIST1H1C,HIST1H1D,HIST1H1E,HIST1H2AB,HIST1H2AC,HIST1H2AD,HIST1H2AE,HIST1H2AG,HIST1H2AH,HIST1H2AI,HIST1H2AJ,HIST1H2AK,HIST1H2AL,HIST1H2AM,HIST1H3A,HIST1H3B,HIST1H3C,HIST1H3F,HIST1H3G,HIST1H3H,HIST1H3I,HIST1H3J,HIST1H4A,HIST1H4B,HIST1H4C,HIST1H4D,HIST1H4E,HIST1H4F,HIST1H4I,HIST1H4J,HIST1H4K,HIST1H4L,HIST2H2AA3,HIST2H2AA4,HIST2H2AB,HIST2H2AC,HIST2H3A,HIST2H3C,HIST2H3D,HMGB1,HMGB2,HMGN2,ID1,LRRFIP1,MTERF3,NOTCH2,PARP1,PCNA,PHB2,PHF19,PLK1,PRDM1,RBBP8,RBL1,SUV39H1,SUV39H2,TIMELESS,TRIM28,UHRF1,ZNF93
ribonucleoprotein complex localization	10	ALYREF,BUD13,DDX39A,GLE1,NDC1,NOP58,NUP107,NUP153,NUP155,NUP160,NUP188,NUP205,NUP35,NUP43,NUP58,NUP85,NUP88,SRSF7,THOC3
nucleus organization	10	CCNB1,CCNB2,CDK1,CEP55,EMG1,HMGB2,NDC1,NOLC1,NUP107,NUP153,NUP155,NUP160,NUP188,NUP205,NUP35,NUP43,NUP58,NUP85,NUP88,PLK1,REEP4,VRK1
chromosome segregation	10	ANAPC15,AURKB,BIRC5,BRCA1,BRCA2,BUB1,BUB1B,BUB3,CCNB1,CDC20,CDC6,CDCA2,CDCA5,CDCA8,CENPA,CENPE,CENPF,CENPH,CENPI,CENPK,CENPL,CENPM,CENPN,CENPO,CENPP,CENPQ,CENPU,CENPW,CEP192,CEP55,CIT,CKAP5,CTCF,DDX11,DLGAP5,DMC1,DSCC1,DSN1,ECT2,ERCC6L,ESCO2,ESPL1,FANCD2,FANCM,GEN1,GSG2,HJURP,INCENP,ITGB3BP,KIF11,KIF14,KIF18A,KIF18B,KIF22,KIF23,KIF2C,KIF4A,KIF4B,KIFC1,KNSTRN,KNTC1,MAD2L1,MCMBP,MIS18A,MLH1,MTBP,NCAPD2,NCAPD3,NCAPG,NCAPH,NDC1,NDC80,NDE1,NEK2,NEK3,NUF2,NUP107,NUP160,NUP43,NUP85,NUSAP1,PHB2,PLK1,PRC1,PSRC1,PTTG1,RACGAP1,RAD18,RCC1,SGO1,SKA1,SKA2,SKA3,SLF1,SMC1A,SMC2,SMC3,SMC4,SPAG5,SPC24,SPC25,SPDL1,STIL,TOP2A,TRIP13,TTK,UBE2C,ZWILCH,ZWINT
negative regulation of DNA metabolic process	10	ACD,BLM,BRCA2,CDC6,CHEK1,GMNN,MLH1,MSH2,MSH6,PARP1,PARPBP,POLQ,RAD18,SMC1A,SMC3,TIPIN,TRIM28,ZNF93
microtubule cytoskeleton organization	10	ASPM,AUNIP,AURKA,AURKB,BIRC5,BORA,BRCA1,BRCA2,CCNB1,CCNF,CCP110,CDK1,CDK2,CENPA,CENPE,CENPJ,CEP135,CEP152,CEP192,CEP76,CEP97,CHEK1,CHORDC1,CKAP2,CKAP5,CLIP3,ECT2,ESPL1,FBXO5,FIGNL1,GAS2L3,GEN1,GPSM2,HAUS4,HAUS8,HSPH1,KIF11,KIF14,KIF18A,KIF18B,KIF20A,KIF23,KIF24,KIF2C,KIF4A,KIF4B,KIFC1,KNSTRN,MLH1,MYBL2,NDC1,NDC80,NDE1,NEK2,NUSAP1,PARP3,PLK1,PLK4,PRC1,PSRC1,RACGAP1,RANBP1,RBM14,RCC1,SASS6,SGO1,SKA1,SKA2,SKA3,SMC1A,SMC3,SPAG5,SPC25,SPDL1,STIL,STMN1,TACC3,TPX2,TTK,TUBGCP3,TUBGCP4,WDR62,XRCC2
mitotic spindle checkpoint	10	ANAPC15,BUB1,BUB1B,BUB3,CCNB1,CENPE,CENPF,CEP192,GEN1,MAD2L1,MTBP,PLK1,STIL,TTK
meiotic nuclear division	10	ASPM,AURKA,BRCA2,CDC20,CDK2,CKS2,DMC1,ESPL1,FANCA,FANCD2,FANCM,FBXO5,KIF18A,MASTL,MKI67,MLH1,MSH2,MSH6,NDC1,PDE3A,PLK1,PTTG1,RAD51,RAD54B,RAD54L,SGO1,SMC1A,SMC2,SMC3,SMC4,TOP2A,TOPBP1,TRIP13,TUBGCP3,TUBGCP4,XRCC2
DNA replication-dependent nucleosome assembly	10	ASF1A,ASF1B,CHAF1A,CHAF1B,HAT1,HIST1H3A,HIST1H3B,HIST1H3C,HIST1H3F,HIST1H3G,HIST1H3H,HIST1H3I,HIST1H3J,HIST1H4A,HIST1H4B,HIST1H4C,HIST1H4D,HIST1H4E,HIST1H4F,HIST1H4I,HIST1H4J,HIST1H4K,HIST1H4L,HIST2H3D,NASP
regulation of mitotic cell cycle	10	ANAPC1,ANAPC15,ANLN,AURKA,BIRC5,BLM,BORA,BRCA2,BTG3,BUB1,BUB1B,BUB3,CASP2,CCNA2,CCNB1,CDC20,CDC25C,CDC45,CDC6,CDC7,CDCA5,CDK1,CDK2,CDKN2A,CDKN2B,CENPE,CENPF,CENPJ,CEP192,CEP97,CHEK1,CIT,CLSPN,DLGAP5,ESPL1,EZH2,FANCI,FBXO5,GEN1,GSG2,GTSE1,HMGB1,KIF11,KIF14,KIF20B,KNTC1,LMNB1,MAD2L1,MSH2,MTBP,NEK2,NUSAP1,PARP3,PCNA,PHB2,PKMYT1,PLK1,PSRC1,PTTG1,RANBP1,RCC1,RFWD3,SLF1,SMC1A,STIL,TICRR,TIPIN,TOP2A,TPX2,TTK,UBE2C,ZWILCH,ZWINT
strand displacement	10	BARD1,BLM,BRCA1,BRCA2,BRIP1,DNA2,EXO1,RAD51,RAD51AP1,RBBP8,RMI1,XRCC2
membrane disassembly	10	CCNB1,CCNB2,CDK1,NDC1,NUP107,NUP153,NUP155,NUP160,NUP188,NUP205,NUP35,NUP43,NUP58,NUP85,NUP88,PLK1,VRK1
nuclear export	10	ALYREF,BARD1,BUD13,CDKN2A,CSE1L,DDX39A,GLE1,GTSE1,NDC1,NUP107,NUP153,NUP155,NUP160,NUP188,NUP205,NUP35,NUP43,NUP58,NUP85,NUP88,RANBP1,SRSF7,THOC3
single organism reproductive process	10	ARSA,ASF1B,ASPM,AURKA,BRCA2,BUB3,CASP2,CCNB1,CCNF,CDC20,CDC25C,CDK1,CDK2,CENPI,CGA,CIT,CKS2,DDIAS,DIAPH3,DMC1,E2F8,EMP2,ESPL1,EXO1,FANCA,FANCC,FANCD2,FANCG,FANCM,FBXO5,FNDC3A,H2AFX,HIST1H1A,HMGB2,IL4R,KIAA1524,KIF18A,KIFC1,KRT19,MASTL,MCM8,MFGE8,MKI67,MLH1,MND1,MSH2,MSH6,NASP,NCAPD2,NCAPD3,NCL,NDC1,NEK2,NOTCH2,ODF2,PDE3A,PHB2,PLK1,PLK4,PRDM1,PSAP,PSMC3IP,PTTG1,RACGAP1,RAD18,RAD51,RAD54B,RAD54L,RBBP8,RPL39L,RRM1,RUVBL1,SGO1,SMC1A,SMC2,SMC3,SMC4,SPATA5,TCP1,TK1,TOP2A,TOPBP1,TRIM28,TRIP13,TUBD1,TUBGCP3,TUBGCP4,WNT7B,XRCC2
chromosome organization	10	ACD,ACTL6A,ANAPC15,ANP32E,ASF1A,ASF1B,ATAD2,AURKA,AURKB,BIRC5,BLM,BRCA1,BRCA2,BRIP1,BUB1,BUB1B,BUB3,CCNB1,CDC20,CDC45,CDC6,CDCA5,CDCA8,CDK1,CDK2,CDKN2A,CENPA,CENPE,CENPF,CENPH,CENPI,CENPK,CENPL,CENPM,CENPN,CENPO,CENPP,CENPQ,CENPU,CENPW,CEP192,CEP55,CHAF1A,CHAF1B,CHEK1,CIT,CKAP5,CTCF,DCLRE1B,DCLRE1C,DDX11,DDX12P,DLGAP5,DMC1,DNA2,DNMT1,DSCC1,DSN1,ERCC6L,ESPL1,EZH2,FANCD2,FBL,FEN1,GEN1,GINS1,GINS2,GINS4,GSG2,H2AFX,H2AFZ,H2BFS,HAT1,HELLS,HIST1H1A,HIST1H1B,HIST1H1C,HIST1H1D,HIST1H1E,HIST1H2AB,HIST1H2AC,HIST1H2AD,HIST1H2AE,HIST1H2AG,HIST1H2AH,HIST1H2AI,HIST1H2AJ,HIST1H2AK,HIST1H2AL,HIST1H2AM,HIST1H2BB,HIST1H2BC,HIST1H2BD,HIST1H2BE,HIST1H2BF,HIST1H2BG,HIST1H2BH,HIST1H2BI,HIST1H2BK,HIST1H2BL,HIST1H2BM,HIST1H2BN,HIST1H2BO,HIST1H3A,HIST1H3B,HIST1H3C,HIST1H3F,HIST1H3G,HIST1H3H,HIST1H3I,HIST1H3J,HIST1H4A,HIST1H4B,HIST1H4C,HIST1H4D,HIST1H4E,HIST1H4F,HIST1H4I,HIST1H4J,HIST1H4K,HIST1H4L,HIST2H2AA3,HIST2H2AA4,HIST2H2AB,HIST2H2AC,HIST2H2BD,HIST2H2BE,HIST2H2BF,HIST2H3A,HIST2H3C,HIST2H3D,HJURP,HMGB1,HMGB2,HMGN3,INCENP,ITGB3BP,KIF14,KIF18A,KIF18B,KIF22,KIF23,KIF2C,KIF4A,KIF4B,KIFC1,KNSTRN,KNTC1,LIG1,MAD2L1,MCM2,MCM3,MCM4,MCM5,MCM6,MCM7,MCMBP,MIS18A,MLH1,MSH2,MSH6,MTBP,NASP,NCAPD2,NCAPD3,NCAPG,NCAPG2,NCAPH,NDC1,NDC80,NDE1,NEK2,NUF2,NUP107,NUP155,NUP160,NUP43,NUP85,NUSAP1,PARP1,PARP3,PAXIP1,PCNA,PHB2,PHF19,PLK1,POLA1,POLA2,POLD3,POLE,POLE2,POLE3,PRC1,PRIM1,PSRC1,PTTG1,RACGAP1,RAD51,RAD54B,RAD54L,RBL1,RBM14,RFC2,RFC3,RFC4,RUVBL1,SGO1,SKA1,SKA2,SLF1,SMC1A,SMC2,SMC3,SMC4,SMCHD1,SPAG5,SPC24,SPC25,SPDL1,STIL,SUV39H1,SUV39H2,TAF5,TCP1,TOP2A,TRIM28,TRIP13,TTK,UBE2C,UHRF1,VRK1,WDHD1,ZWILCH,ZWINT
mitotic centrosome separation	10	AURKA,CHEK1,KIF11,NDE1,NEK2,RANBP1
G2 DNA damage checkpoint	10	BLM,BRCA1,CCNA2,CDK1,CHEK1,CLSPN,DTL,FANCI,PLK1,RBBP8
negative regulation of mitotic sister chromatid separation	10	ANAPC15,BUB1,BUB1B,BUB3,CCNB1,CENPE,CENPF,CEP192,GEN1,MAD2L1,MTBP,PLK1,PTTG1,STIL,TTK
intracellular transport of virus	10	ALYREF,KPNA2,NDC1,NUP107,NUP153,NUP155,NUP160,NUP188,NUP205,NUP35,NUP43,NUP58,NUP85,NUP88,THOC3
cell cycle checkpoint	10	ANAPC15,AURKA,AURKB,BIRC5,BLM,BRCA1,BRIP1,BUB1,BUB1B,BUB3,CASP2,CCNA2,CCNB1,CCNE2,CDC25C,CDC45,CDC6,CDK1,CDK2,CDKN2B,CDT1,CENPE,CENPF,CENPJ,CEP192,CHEK1,CLSPN,DCLRE1B,DNA2,DTL,FANCG,FANCI,GEN1,GSG2,GTSE1,H2AFX,KNTC1,MAD2L1,MSH2,MTBP,PCNA,PLK1,RBBP8,RFWD3,SMC1A,SPDL1,STIL,TICRR,TIPIN,TOP2A,TTK,ZWILCH,ZWINT
DNA replication-dependent	10	ASF1A,ASF1B,CHAF1A,CHAF1B,HAT1,HIST1H3A,HIST1H3B,HIST1H3C,HIST1H3F,HIST1H3G,HIST1H3H,HIST1H3I,HIST1H3J,HIST1H4A,HIST1H4B,HIST1H4C,HIST1H4D,HIST1H4E,HIST1H4F,HIST1H4I,HIST1H4J,HIST1H4K,HIST1H4L,HIST2H3D,NASP

nucleosome organization		
chromatin assembly or disassembly	10	ASF1A,ASF1B,CDKN2A,CENPA,CENPH,CENPI,CENPK,CENPL,CENPM,CENPN,CENPO,CENPP,CENPQ,CENPU,CENPW,CHAF1A,CHAF1B,CTCF,H2AFX,H2BFS,HAT1,HELLS,HIST1H1A,HIST1H1B,HIST1H1C,HIST1H1D,HIST1H1E,HIST1H2BB,HIST1H2BC,HIST1H2BD,HIST1H2BE,HIST1H2BF,HIST1H2BG,HIST1H2BH,HIST1H2BI,HIST1H2BK,HIST1H2BL,HIST1H2BM,HIST1H2BN,HIST1H2BO,HIST1H3A,HIST1H3B,HIST1H3C,HIST1H3F,HIST1H3G,HIST1H3H,HIST1H3I,HIST1H3J,HIST1H4A,HIST1H4B,HIST1H4C,HIST1H4D,HIST1H4E,HIST1H4F,HIST1H4I,HIST1H4J,HIST1H4K,HIST1H4L,HIST2H2BD,HIST2H2BE,HIST2H2BF,HIST2H3A,HIST2H3C,HIST2H3D,HJURP,HMGB1,HMGB2,ITGB3BP,MCM2,MIS18A,NASP,RUVBL1,SUV39H2
nuclear envelope disassembly	10	CCNB1,CCNB2,CDK1,NDC1,NUP107,NUP153,NUP155,NUP160,NUP188,NUP205,NUP35,NUP43,NUP58,NUP85,NUP88,PLK1,VRK1
positive regulation of cell division	10	AURKA,AURKB,BIRC5,CDC6,CDCA5,CENPE,CIT,DLGAP5,ECT2,ESPL1,FBXO5,KIF14,KIF20B,KIF23,NUSAP1,PHB2,RACGAP1,RANBP1,SLF1,UBE2C
mitotic cell cycle	10	ANAPC1,ANAPC15,ANLN,ASPM,AURKA,AURKB,BIRC5,BLM,BORA,BRCA2,BTG3,BUB1,BUB1B,BUB3,CASP2,CCNA2,CCNB1,CCNB2,CCNE2,CCNF,CCP110,CDC20,CDC25A,CDC25C,CDC45,CDC6,CDC7,CDCA2,CDCA3,CDCA5,CDCA8,CDH13,CDK1,CDK2,CDKN2A,CDKN2B,CDKN2C,CDKN3,CDT1,CENPA,CENPE,CENPF,CENPH,CENPJ,CENPN,CENPW,CEP135,CEP152,CEP192,CEP55,CEP76,CEP78,CEP97,CHEK1,CIT,CKAP2,CKAP5,CKS1B,CKS2,CLSPN,CNTRL,DHFR,DHFRP1,DLGAP5,DNA2,DSCC1,DSN1,E2F8,ERCC6L,ESPL1,EZH2,FAM64A,FANCI,FBXO5,FOXM1,GEN1,GINS1,GINS2,GPSM2,GSG2,GTSE1,HAUS4,HAUS8,HELLS,HMGB1,HMMR,INCENP,IQGAP3,ITGB3BP,KIF11,KIF14,KIF15,KIF18A,KIF18B,KIF20A,KIF20B,KIF22,KIF23,KIF2C,KIF4A,KIF4B,KIFC1,KNSTRN,KNTC1,LIG1,LMNB1,MAD2L1,MASTL,MCM10,MCM2,MCM3,MCM4,MCM5,MCM6,MCM7,MCM8,MCMBP,MELK,MIS18A,MSH2,MTBP,MYBL2,NCAPD2,NCAPD3,NCAPG,NCAPG2,NCAPH,NDC1,NDC80,NDE1,NEK2,NEK3,NOLC1,NUF2,NUP107,NUP153,NUP155,NUP160,NUP188,NUP205,NUP35,NUP43,NUP58,NUP85,NUP88,NUSAP1,ODF2,ORC1,ORC5,ORC6,PARP3,PBK,PCNA,PHB2,PKMYT1,PLK1,PLK4,POLA1,POLA2,POLE,POLE2,PRC1,PRIM1,PSRC1,PTTG1,RACGAP1,RANBP1,RBBP8,RCC1,REEP4,RFWD3,RRM1,RRM2,RUVBL1,SGO1,SKA1,SKA2,SKA3,SLF1,SMC1A,SMC2,SMC3,SMC4,SPAG5,SPC24,SPC25,SPDL1,STIL,STMN1,TCF19,TICRR,TIMELESS,TIPIN,TOP2A,TPX2,TTK,TUBGCP3,TUBGCP4,TYMS,UBE2C,UBE2S,VRK1,WDR62,XRCC2,ZWILCH,ZWINT
protein sumoylation	10	AURKB,BIRC5,BLM,BRCA1,CDCA8,CDKN2A,INCENP,NDC1,NOP58,NUP107,NUP153,NUP155,NUP160,NUP188,NUP205,NUP35,NUP43,NUP58,NUP85,NUP88,PARP1,PCNA,SENP1,SMC1A,SMC3,TOP2A,TRIM28,TRPM4
chromatin remodeling	10	ACTL6A,ANP32E,CDKN2A,CENPA,CENPH,CENPI,CENPK,CENPL,CENPM,CENPN,CENPO,CENPP,CENPQ,CENPU,CENPW,CHEK1,HELLS,HIST1H4A,HIST1H4B,HIST1H4C,HIST1H4D,HIST1H4E,HIST1H4F,HIST1H4I,HIST1H4J,HIST1H4K,HIST1H4L,HJURP,ITGB3BP,MIS18A,NASP,RUVBL1,SUV39H2
regulation of centrosome duplication	10	BRCA1,CCNF,CENPJ,CEP192,CEP76,CHORDC1,GEN1,KIFC1,PLK4,RBM14
DNA replication-independent nucleosome organization	10	ASF1A,ASF1B,CENPA,CENPH,CENPI,CENPK,CENPL,CENPM,CENPN,CENPO,CENPP,CENPQ,CENPU,CENPW,HAT1,HIST1H4A,HIST1H4B,HIST1H4C,HIST1H4D,HIST1H4E,HIST1H4F,HIST1H4I,HIST1H4J,HIST1H4K,HIST1H4L,HJURP,ITGB3BP,MIS18A,NASP,RUVBL1
nucleic acid phosphodiester bond hydrolysis	10	APAF1,CDKN2A,CSTF2,DCLRE1B,DCLRE1C,DNA2,EME1,EXO1,EXOSC2,EXOSC9,FANCM,FEN1,GEN1,HMGB1,HMGB2,MGME1,PARP1,PCNA,POLA1,POLD3,POLE,RBBP8,RFC2,RFC3,RFC4,RNASEH2A,RPP30,TDP1,TOE1
nucleocytoplasmic transport	10	ALYREF,BARD1,BRCA1,BUD13,CDK1,CDKN2A,CSE1L,DDX39A,ECT2,GDF5,GEMIN5,GEMIN6,GLE1,GTSE1,KPNA2,NDC1,NOP58,NUP107,NUP153,NUP155,NUP160,NUP188,NUP205,NUP35,NUP43,NUP58,NUP85,NUP88,PARP1,PHB2,POLA2,RANBP1,SNRPB,SNRPD1,SRSF7,TACC3,THOC3,TRIM28
regulation of DNA recombination	10	ALYREF,BLM,CHEK1,FIGNL1,KPNA2,MLH1,MSH2,MSH6,PARPBP,PAXIP1,POLQ,RAD18,RAD51,RAD51AP1,UNG
DNA replication checkpoint	10	CDC45,CDC6,CDT1,CLSPN,DNA2,TICRR,TIPIN
RNA localization	10	ALYREF,BUD13,CKAP5,DDX39A,EXOSC2,FBL,GLE1,HNRNPA3,NDC1,NOP58,NUP107,NUP153,NUP155,NUP160,NUP188,NUP205,NUP35,NUP43,NUP58,NUP85,NUP88,RANBP1,RUVBL1,SRSF7,TCP1,THOC3
DNA conformation change	10	ASF1A,ASF1B,BLM,BRIP1,CCNB1,CDC45,CDCA5,CDK1,CDKN2A,CENPA,CENPH,CENPI,CENPK,CENPL,CENPM,CENPN,CENPO,CENPP,CENPQ,CENPU,CENPW,CHAF1A,CHAF1B,CTCF,DDX11,DDX12P,DNA2,GINS1,GINS2,GINS4,H2AFX,H2BFS,HAT1,HELLS,HIST1H1A,HIST1H1B,HIST1H1C,HIST1H1D,HIST1H1E,HIST1H2BB,HIST1H2BC,HIST1H2BD,HIST1H2BE,HIST1H2BF,HIST1H2BG,HIST1H2BH,HIST1H2BI,HIST1H2BK,HIST1H2BL,HIST1H2BM,HIST1H2BN,HIST1H2BO,HIST1H3A,HIST1H3B,HIST1H3C,HIST1H3F,HIST1H3G,HIST1H3H,HIST1H3I,HIST1H3J,HIST1H4A,HIST1H4B,HIST1H4C,HIST1H4D,HIST1H4E,HIST1H4F,HIST1H4I,HIST1H4J,HIST1H4K,HIST1H4L,HIST2H2BD,HIST2H2BE,HIST2H2BF,HIST2H3A,HIST2H3C,HIST2H3D,HJURP,HMGB1,HMGB2,ITGB3BP,MCM2,MCM3,MCM4,MCM5,MCM6,MCM7,MIS18A,NASP,NCAPD2,NCAPD3,NCAPG,NCAPG2,NCAPH,NUSAP1,PARP1,RAD51,RAD54B,RUVBL1,SMC2,SMC4,SUV39H1,TOP2A
response to ionizing radiation	10	BLM,BRCA1,BRCA2,DCLRE1C,DDIAS,DMC1,DNMT1,ECT2,FANCD2,FIGNL1,H2AFX,MSH2,PAXIP1,RAD51,RAD51AP1,RAD54B,RAD54L,RFWD3,RRM1,SOD2,TICRR,TMEM109,TOPBP1,XRCC2
DNA geometric change	10	BLM,BRIP1,CDC45,DDX11,DDX12P,DNA2,GINS1,GINS2,GINS4,HMGB1,HMGB2,MCM2,MCM3,MCM4,MCM5,MCM6,MCM7,PARP1,RAD51,RAD54B,RUVBL1,TOP2A
cellular macromolecular complex assembly	10	ANLN,ASF1A,ASF1B,CDC42EP3,CDC45,CENPA,CENPE,CENPF,CENPH,CENPI,CENPJ,CENPK,CENPL,CENPM,CENPN,CENPO,CENPP,CENPQ,CENPU,CENPW,CEP192,CHAF1A,CHAF1B,CLIP3,DMC1,FBXO5,GEMIN5,GEMIN6,GSN,H2AFX,H2BFS,HAT1,HELLS,HIST1H1A,HIST1H1B,HIST1H1C,HIST1H1D,HIST1H1E,HIST1H2BB,HIST1H2BC,HIST1H2BD,HIST1H2BE,HIST1H2BF,HIST1H2BG,HIST1H2BH,HIST1H2BI,HIST1H2BK,HIST1H2BL,HIST1H2BM,HIST1H2BN,HIST1H2BO,HIST1H3A,HIST1H3B,HIST1H3C,HIST1H3F,HIST1H3G,HIST1H3H,HIST1H3I,HIST1H3J,HIST1H4A,HIST1H4B,HIST1H4C,HIST1H4D,HIST1H4E,HIST1H4F,HIST1H4I,HIST1H4J,HIST1H4K,HIST1H4L,HIST2H2BD,HIST2H2BE,HIST2H2BF,HIST2H3A,HIST2H3C,HIST2H3D,HJURP,HLA-DMB,HMGB1,HMGB2,ITGB3BP,LSM2,LSM3,MCM2,MIS18A,MRTO4,NASP,NDC1,NDE1,NUP107,NUP153,NUP205,PARP1,PRPF3,PSRC1,RAD51,RBMX,RBMXL1,RPL23A,RUVBL1,SNRPB,SNRPD1,STMN1,TCP1,TFAM,TICRR,TIMM21,TUBGCP3,TUBGCP4,UBE2C,UBE2S,XRCC2
DNA replication initiation	10	CCNE2,CDC45,CDC6,CDC7,CDK2,CDT1,GINS2,GINS4,MCM10,MCM2,MCM3,MCM4,MCM5,MCM6,MCM7,ORC1,ORC5,POLA1,POLA2,POLE,POLE2,PRIM1,TICRR
DNA strand elongation involved in DNA replication	10	DNA2,FEN1,GINS1,GINS2,GINS3,GINS4,LIG1,PARP1,PARP2,PARP3,PCNA,POLA1,POLA2,POLD3,PRIM1,RFC3,RFC4,RNASEH2A
DNA damage checkpoint	10	AURKA,BLM,BRCA1,BRIP1,CASP2,CCNA2,CCNB1,CDC25C,CDK1,CDK2,CENPJ,CHEK1,CLSPN,DTL,FANCI,GTSE1,H2AFX,MSH2,PCNA,PLK1,RBBP8,RFWD3,TIPIN
regulation of organelle organization	10	ACD,ACTL6A,ANAPC15,ANLN,ASF1A,ATAD2,AURKA,AURKB,BIRC5,BORA,BRCA1,BUB1,BUB1B,BUB3,CCNB1,CCNF,CCP110,CDC20,CDC25C,CDC42EP3,CDC45,CDC6,CDCA5,CDKN2A,CENPE,CENPF,CENPJ,CEP135,CEP192,CEP295,CEP76,CEP97,CHEK1,CHORDC1,CIT,CKAP2,CLIP3,CTCF,DLGAP5,DNMT1,ECT2,ESPL1,FBXO5,GEN1,GSN,HIST1H1B,HSPH1,ID1,KIF11,KIF18A,KIF20B,KIFC1,KNSTRN,KNTC1,MAD2L1,MIS18A,MSH2,MTBP,NEK2,NUSAP1,ODF2,PARP1,PARP3,PAXIP1,PDE3A,PHB2,PHF19,PKMYT1,PLK1,PLK4,PSRC1,PTTG1,RACGAP1,RANBP1,RBM14,RCC1,RUVBL1,SDC4,SKA1,SKA2,SKA3,SLF1,SPAG5,STIL,STMN1,TCP1,TPX2,TRIM28,TTK,UBE2C,UHRF1,VAT1,WDHD1
DNA-dependent DNA replication	10	ALYREF,BLM,BRCA2,CCNE2,CDC45,CDC6,CDC7,CDK2,CDT1,DNA2,DSCC1,E2F8,FANCM,FEN1,GINS1,GINS2,GINS3,GINS4,LIG1,MCM10,MCM2,MCM3,MCM4,MCM5,MCM6,MCM7,MCMBP,MGME1,MMS22L,ORC1,ORC5,ORC6,PARP1,PARP2,PARP3,PCNA,POLA1,POLA2,POLD3,POLE,POLE2,POLQ,PRIM1,RAD51,RFC2,RFC3,RFC4,RNASEH2A,SMC1A,SMC3,TFAM,TICRR,TIPIN,TOP2A
negative regulation of nuclear division	10	ANAPC15,BUB1,BUB1B,BUB3,CCNB1,CENPE,CENPF,CEP192,CHEK1,FBXO5,GEN1,MAD2L1,MSH2,MTBP,PLK1,PTTG1,STIL,TTK
regulation of mitotic sister chromatid segregation	10	ANAPC15,BUB1,BUB1B,BUB3,CCNB1,CDC6,CENPE,CENPF,CEP192,CIT,DLGAP5,ESPL1,GEN1,MAD2L1,MTBP,PLK1,PTTG1,SLF1,STIL,TTK,UBE2C
telomere maintenance via recombination	10	BRCA2,DNA2,FEN1,LIG1,PCNA,POLA1,POLA2,POLD3,POLE,POLE2,PRIM1,RAD51,RFC2,RFC3,RFC4

protein-DNA complex subunit organization	10	ANP32E,ASF1A,ASF1B,CDC45,CENPA,CENPE,CENPF,CENPH,CENPI,CENPK,CENPL,CENPM,CENPN,CENPO,CENPP,CENPQ,CENPU,CENPW,CHAF1A,CHAF1B,CTCF,DMC1,H2AFX,H2BFS,HAT1,HELLS,HIST1H1A,HIST1H1B,HIST1H1C,HIST1H1D,HIST1H1E,HIST1H2BB,HIST1H2BC,HIST1H2BD,HIST1H2BE,HIST1H2BF,HIST1H2BG,HIST1H2BH,HIST1H2BI,HIST1H2BK,HIST1H2BL,HIST1H2BM,HIST1H2BN,HIST1H2BO,HIST1H3A,HIST1H3B,HIST1H3C,HIST1H3F,HIST1H3G,HIST1H3H,HIST1H3I,HIST1H3J,HIST1H4A,HIST1H4B,HIST1H4C,HIST1H4D,HIST1H4E,HIST1H4F,HIST1H4I,HIST1H4J,HIST1H4K,HIST1H4L,HIST2H2BD,HIST2H2BE,HIST2H2BF,HIST2H3A,HIST2H3C,HIST2H3D,HJURP,HMGB1,HMGB2,ITGB3BP,MCM2,MIS18A,NASP,PARP1,RAD51,RUVBL1,XRCC2
regulation of microtubule cytoskeleton organization	10	AURKA,BORA,BRCA1,CCNB1,CCNF,CENPE,CENPJ,CEP192,CEP76,CEP97,CHEK1,CHORDC1,CKAP2,CLIP3,ECT2,GEN1,HSPH1,KIF11,KIF18A,KIFC1,KNSTRN,NEK2,PARP3,PLK1,PLK4,PSRC1,RACGAP1,RANBP1,RBM14,SKA1,SKA2,SKA3,SPAG5,STMN1,TPX2
regulation of cyclin-dependent protein serine/threonine kinase activity	10	BLM,CCNA2,CCNE2,CDC25A,CDC25C,CDC6,CDKN2A,CDKN2B,CDKN2C,CDKN3,CKS1B,CKS2,LMNB1,PKMYT1,PLK1,PSRC1,STIL,UBE2C
microtubule cytoskeleton organization involved in mitosis	10	AURKB,BIRC5,CEP97,KIF11,KIF23,KIF4A,KIF4B,KIFC1,MYBL2,NEK2,PLK1,RACGAP1,TPX2,TUBGCP3,TUBGCP4
RNA export from nucleus	10	ALYREF,BUD13,DDX39A,GLE1,NDC1,NUP107,NUP153,NUP155,NUP160,NUP188,NUP205,NUP35,NUP43,NUP58,NUP85,NUP88,RANBP1,SRSF7,THOC3
centrosome duplication	10	BRCA1,BRCA2,CCNF,CCP110,CDK2,CENPJ,CEP135,CEP152,CEP192,CEP76,CHORDC1,GEN1,KIFC1,NDE1,PLK4,RBM14,SASS6,STIL,TUBGCP3,TUBGCP4,WDR62
spindle midzone assembly	10	AURKB,KIF23,KIF4A,KIF4B,MLH1,RACGAP1
DNA recombination	10	ACTL6A,ALYREF,BARD1,BLM,BRCA1,BRCA2,BRIP1,CDC45,CDC7,CHEK1,DCLRE1C,DMC1,DNA2,EME1,EXO1,FANCM,FEN1,FIGNL1,GEN1,GINS2,GINS4,H2AFX,HMGB1,HMGB2,KPNA2,LIG1,MCM8,MLH1,MMS22L,MND1,MSH2,MSH6,PARP1,PARPBP,PAXIP1,PCNA,POLA1,POLA2,POLD3,POLE,POLE2,POLQ,PRIM1,PSMC3IP,RAD18,RAD51,RAD51AP1,RAD54B,RAD54L,RBBP8,RBM14,RFC2,RFC3,RFC4,RMI1,RUVBL1,TOP2A,TOPBP1,TRIP13,UNG,XRCC2
mitotic cell cycle phase transition	10	ANAPC1,ANAPC15,ANLN,AURKA,BIRC5,BLM,BORA,BUB1,BUB1B,BUB3,CASP2,CCNA2,CCNB1,CCNB2,CCNE2,CCP110,CDC20,CDC25A,CDC25C,CDC45,CDC6,CDC7,CDCA5,CDK1,CDK2,CDKN2A,CDKN2B,CDKN2C,CDKN3,CDT1,CENPE,CENPF,CENPJ,CEP135,CEP152,CEP192,CEP76,CEP78,CHEK1,CIT,CKAP5,CKS1B,CKS2,CLSPN,CNTRL,DHFR,DHFRP1,DLGAP5,ESPL1,EZH2,FANCI,FBXO5,FOXM1,GEN1,GSG2,GTSE1,HAUS4,HAUS8,HMMR,IQGAP3,KIF14,KNTC1,LMNB1,MAD2L1,MASTL,MCM10,MCM2,MCM3,MCM4,MCM5,MCM6,MCM7,MCM8,MELK,MTBP,NDE1,NEK2,ODF2,ORC1,ORC5,ORC6,PCNA,PHB2,PKMYT1,PLK1,PLK4,POLA1,POLA2,POLE,POLE2,PRIM1,RANBP1,RBBP8,RCC1,RFWD3,RRM2,STIL,TCF19,TICRR,TPX2,TTK,TYMS,UBE2C,UBE2S
sister chromatid segregation	10	ANAPC15,AURKB,BIRC5,BUB1,BUB1B,BUB3,CCNB1,CDC20,CDC6,CDCA5,CDCA8,CENPA,CENPE,CENPF,CENPH,CENPI,CENPK,CENPL,CENPM,CENPN,CENPO,CENPP,CENPQ,CENPU,CEP192,CEP55,CIT,CKAP5,CTCF,DDX11,DLGAP5,DSCC1,DSN1,ERCC6L,ESPL1,GEN1,GSG2,INCENP,ITGB3BP,KIF14,KIF18A,KIF18B,KIF22,KIF23,KIF2C,KIF4A,KIF4B,KIFC1,KNSTRN,KNTC1,MAD2L1,MCMBP,MTBP,NCAPD2,NCAPD3,NCAPG,NCAPH,NDC80,NDE1,NEK2,NUF2,NUP107,NUP160,NUP43,NUP85,NUSAP1,PHB2,PLK1,PRC1,PSRC1,PTTG1,RACGAP1,SGO1,SKA1,SKA2,SLF1,SMC1A,SMC2,SMC3,SMC4,SPAG5,SPC24,SPC25,SPDL1,STIL,TOP2A,TTK,UBE2C,ZWILCH,ZWINT
negative regulation of megakaryocyte differentiation	10	HIST1H4A,HIST1H4B,HIST1H4C,HIST1H4D,HIST1H4E,HIST1H4F,HIST1H4I,HIST1H4J,HIST1H4K,HIST1H4L
protein localization to chromosome	10	ACD,AURKB,BRCA2,BUB1B,CDCA5,CDK1,CENPA,ESCO2,EZH2,GSG2,HIST1H1B,MTBP,PARP3,PLK1,SLF1,SPDL1,TCP1
chromosome condensation	10	CCNB1,CDCA5,CDK1,NCAPD2,NCAPD3,NCAPG,NCAPG2,NCAPH,NUSAP1,SMC2,SMC4,TOP2A
centriole assembly	10	BRCA1,CCP110,CDK2,CENPJ,CEP135,CEP152,CEP192,CEP76,PLK4,RBM14,SASS6,WDR62
DNA replication	10	ACD,ALYREF,AURKB,BARD1,BLM,BRCA1,BRCA2,BRIP1,CACYBP,CCNE2,CDC25A,CDC25C,CDC45,CDC6,CDC7,CDK1,CDK2,CDT1,CHAF1A,CHAF1B,CHEK1,CLSPN,DNA2,DSCC1,DTL,DUT,E2F8,ESCO2,EXO1,FAM111A,FANCM,FEN1,GINS1,GINS2,GINS3,GINS4,GMNN,LIG1,MCM10,MCM2,MCM3,MCM4,MCM5,MCM6,MCM7,MCM8,MCMBP,MGME1,MMS22L,NASP,NEK2,ORC1,ORC5,ORC6,PARP1,PARP2,PARP3,PCNA,POLA1,POLA2,POLD3,POLE,POLE2,POLE3,POLQ,PRIM1,RAD51,RBBP8,RBM14,RFC2,RFC3,RFC4,RMI1,RNASEH2A,RRM1,RRM2,SMC1A,SMC3,SSRP1,TCP1,TFAM,TICRR,TIMELESS,TIPIN,TOP2A,TOPBP1
negative regulation of cell cycle phase transition	10	ANAPC15,AURKA,BLM,BUB1,BUB1B,BUB3,CASP2,CCNA2,CCNB1,CDC25C,CDK1,CDK2,CDKN2B,CENPE,CENPF,CENPJ,CEP192,CHEK1,CLSPN,EZH2,FANCI,GEN1,GSG2,GTSE1,MAD2L1,MTBP,PCNA,PLK1,RFWD3,STIL,SUSD2,TICRR,TTK
metaphase/anaphase transition of cell cycle	10	ANAPC15,BUB1,BUB1B,BUB3,CCNB1,CDC6,CENPE,CENPF,CEP192,CIT,DLGAP5,ESPL1,GEN1,MAD2L1,MTBP,PLK1,STIL,TTK,UBE2C
double-strand break repair via homologous recombination	10	BLM,BRCA1,BRCA2,CDC45,CDC7,CHEK1,DMC1,FEN1,FIGNL1,GEN1,GINS2,GINS4,H2AFX,MCM8,MMS22L,PARP1,PARPBP,POLQ,RAD51,RAD51AP1,RAD54B,RAD54L,RBBP8,XRCC2
multi-organism localization	10	ALYREF,KPNA2,NDC1,NUP107,NUP153,NUP155,NUP160,NUP188,NUP205,NUP35,NUP43,NUP58,NUP85,NUP88,TCP1,THOC3
cellular response to DNA damage stimulus	10	ACD,ACTL6A,ALYREF,ASF1A,ATAD5,AURKA,BARD1,BLM,BRCA1,BRCA2,BRIP1,CASP2,CCNA2,CCNB1,CDC25C,CDC45,CDC7,CDCA5,CDK1,CDK2,CDKN2A,CDKN3,CENPJ,CHAF1A,CHAF1B,CHEK1,CLSPN,DCLRE1B,DCLRE1C,DDIAS,DDX39A,DMC1,DNA2,DTL,EME1,ESCO2,EXO1,FANCA,FANCB,FANCC,FANCD2,FANCG,FANCI,FANCM,FEN1,FIGNL1,FOXM1,GEN1,GINS2,GINS4,GTSE1,H2AFX,HIST1H4A,HIST1H4B,HIST1H4C,HIST1H4D,HIST1H4E,HIST1H4F,HIST1H4I,HIST1H4J,HIST1H4K,HIST1H4L,HMGB1,HMGB2,KIF22,LIG1,MASTL,MCM10,MCM7,MCM8,MGME1,MLH1,MMS22L,MSH2,MSH6,NEIL3,PARP1,PARP2,PARP3,PARPBP,PAXIP1,PCNA,PLK1,POLA1,POLD3,POLE,POLE2,POLQ,PTTG1,RAD18,RAD51,RAD51AP1,RAD54B,RAD54L,RBBP8,RBM14,RFC2,RFC3,RFC4,RFWD3,RMI1,RNASEH2A,RPS6KA6,RUVBL1,SLF1,SMC1A,SMC3,SOD2,SSRP1,SUV39H1,TDP1,TICRR,TIMELESS,TIPIN,TMEM109,TOP2A,TOPBP1,TPT1,TRIM28,TRIP13,UBE2T,UHRF1,UNG,USP1,WDR76,XRCC2
negative regulation of RNA metabolic process	10	ASF1A,ATAD2,ATOH8,AURKB,BARD1,BIRC5,BRCA1,CASP8AP2,CDC45,CDKN2A,CENPF,CTCF,DEPDC1,DNMT1,E2F8,EZH2,FOXM1,GMNN,H2AFX,H2AFZ,HAT1,HELLS,HIST1H1B,HIST1H1C,HIST1H1D,HIST1H1E,HIST1H2AB,HIST1H2AC,HIST1H2AD,HIST1H2AE,HIST1H2AG,HIST1H2AH,HIST1H2AI,HIST1H2AJ,HIST1H2AK,HIST1H2AL,HIST1H2AM,HIST1H3A,HIST1H3B,HIST1H3C,HIST1H3F,HIST1H3G,HIST1H3H,HIST1H3I,HIST1H3J,HIST1H4A,HIST1H4B,HIST1H4C,HIST1H4D,HIST1H4E,HIST1H4F,HIST1H4I,HIST1H4J,HIST1H4K,HIST1H4L,HIST2H2AA3,HIST2H2AA4,HIST2H2AB,HIST2H2AC,HIST2H3A,HIST2H3C,HIST2H3D,HMGB1,HMGB2,HMGN2,ID1,LRRFIP1,MTERF3,NOTCH2,PARP1,PCNA,PHB2,PHF19,PLK1,PRDM1,RBBP8,RBL1,RBMX,SRSF7,SUV39H1,SUV39H2,TIMELESS,TRA2B,TRIM28,UHRF1,ZNF85,ZNF93
positive regulation of DNA metabolic process	10	ACD,AURKB,BRCA1,CACYBP,CDC7,CDK1,CDK2,DNA2,DSCC1,E2F8,FOXM1,H2AFX,HMGB1,MSH6,NEK2,PARP3,PAXIP1,PCNA,RAD51,RFC2,RFC3,RFC4,SLF1,TCP1,TOP2A,TRIM28,UNG
regulation of proteasomal ubiquitin-dependent protein catabolic process	10	ANAPC15,AURKA,BUB1,BUB1B,BUB3,CCNB1,CENPE,CENPF,CEP192,DLGAP5,ESPL1,GEN1,MAD2L1,MTBP,PBK,PLK1,SENP1,STIL,TTK
protein modification by	10	ANAPC1,ANAPC15,AURKA,AURKB,BARD1,BIRC5,BLM,BRCA1,BUB1B,BUB3,CCNB1,CCNF,CDC20,CDCA3,CDCA8,CDK1,CDK2,CDKN2A,DTL,FANCI,FBXO5,G2E3,INCENP,LRR1,MAD2L1,MASTL,NDC1,NOP58,NUP107,NUP153,NUP155,NUP160,NUP188,NUP205,NUP35,NUP43,NUP58,NUP8

small protein conjugation		5,NUP88,PARP1,PAXIP1,PCNA,PLK1,PTTG1,RAD18,RFWD3,SENP1,SMC1A,SMC3,TOP2A,TRAIP,TRIM28,TRIM59,TRPM4,UBE2C,UBE2S,UBE2T,UHRF1,ZNF738
negative regulation of mitotic cell cycle	10	ANAPC15,AURKA,BLM,BTG3,BUB1,BUB1B,BUB3,CASP2,CCNA2,CCNB1,CDC25C,CDK1,CDK2,CDKN2B,CENPE,CENPF,CENPJ,CEP192,CHEK1,CLSPN,EZH2,FANCI,GEN1,GSG2,GTSE1,KNTC1,MAD2L1,MSH2,MTBP,PCNA,PLK1,PTTG1,RFWD3,SMC1A,STIL,TICRR,TIPIN,TOP2A,TTK,ZWILCH,ZWINT
telomere maintenance	10	ACD,AURKB,BLM,BRCA2,DCLRE1B,DCLRE1C,DNA2,FEN1,HIST1H4A,HIST1H4B,HIST1H4C,HIST1H4D,HIST1H4E,HIST1H4F,HIST1H4I,HIST1H4J,HIST1H4K,HIST1H4L,LIG1,NEK2,PARP1,PARP3,PCNA,POLA1,POLA2,POLD3,POLE,POLE2,PRIM1,RAD51,RFC2,RFC3,RFC4,TCP1
regulation of centrosome cycle	10	AURKA,BRCA1,CCNF,CENPJ,CEP192,CEP76,CHEK1,CHORDC1,GEN1,KIF11,KIFC1,NEK2,PLK4,RANBP1,RBM14
innate immune response in mucosa	10	H2BFS,HIST1H2BC,HIST1H2BE,HIST1H2BF,HIST1H2BG,HIST1H2BI,HIST1H2BK,HIST2H2BE
positive regulation of mitotic cell cycle	10	AURKA,BIRC5,BRCA2,CCNB1,CDC45,CDC6,CDC7,CDCA5,CDK1,CENPE,DLGAP5,ESPL1,GEN1,HMGB1,KIF20B,MAD2L1,MTBP,NUSAP1,PHB2,RANBP1,SLF1,UBE2C
double-strand break repair via nonhomologous end joining	10	BARD1,BRCA1,DCLRE1B,DCLRE1C,H2AFX,HIST1H4A,HIST1H4B,HIST1H4C,HIST1H4D,HIST1H4E,HIST1H4F,HIST1H4I,HIST1H4J,HIST1H4K,HIST1H4L,LIG1,MLH1,PAXIP1,POLA1,POLQ
mitotic chromosome condensation	10	CDCA5,NCAPD2,NCAPD3,NCAPG,NCAPH,NUSAP1,SMC2,SMC4
negative regulation of proteasomal protein catabolic process	10	ANAPC15,BUB1,BUB1B,BUB3,CCNB1,CENPE,CENPF,CEP192,GEN1,MAD2L1,MTBP,PBK,PLK1,SENP1,STIL,TTK
kinetochore organization	10	CENPA,CENPE,CENPF,CENPH,CENPW,SMC2,SMC4
metaphase plate congression	10	BRCA2,CCNB1,CDCA5,CDCA8,CENPE,CENPF,CEP55,KIF14,KIF18A,KIF22,KIF2C,KIFC1,MLH1,PSRC1,SPDL1
mitotic spindle elongation	10	AURKB,KIF23,KIF4A,KIF4B,PRC1,RACGAP1
regulation of DNA-dependent DNA replication	10	BLM,BRCA2,CDC7,CDK2,CDT1,DSCC1,E2F8,RFC2,RFC3,RFC4,SMC1A,SMC3,TICRR,TIPIN
anaphase-promoting complex-dependent catabolic process	10	ANAPC1,ANAPC15,AURKA,AURKB,BUB1B,BUB3,CCNB1,CDC20,CDK1,MAD2L1,PLK1,PTTG1,UBE2C,UBE2S
mitotic spindle organization	10	AURKA,AURKB,BIRC5,BORA,CCNB1,CEP97,GPSM2,KIF11,KIF23,KIF4A,KIF4B,KIFC1,MYBL2,NDC80,NEK2,PARP3,PLK1,PRC1,PSRC1,RACGAP1,RCC1,SMC1A,SMC3,SPC25,STIL,STMN1,TPX2,TTK,TUBGCP3,TUBGCP4,WDR62
positive regulation of cell cycle	10	AURKA,AURKB,BIRC5,BRCA1,BRCA2,CASP2,CCNB1,CDC25C,CDC45,CDC6,CDC7,CDCA5,CDK1,CDK2,CDKN2A,CENPE,CENPJ,CIT,DLGAP5,E2F8,ECT2,ESPL1,FBXO5,GEN1,GTSE1,HMGB1,KIF14,KIF20B,KIF23,LMNB1,MAD2L1,MTBP,NUSAP1,ORC1,PCNA,PHB2,PLK4,PSRC1,RACGAP1,RANBP1,SLF1,STIL,UBE2C
CENP-A containing nucleosome assembly	10	CENPA,CENPH,CENPI,CENPK,CENPL,CENPM,CENPN,CENPO,CENPP,CENPQ,CENPU,CENPW,HIST1H4A,HIST1H4B,HIST1H4C,HIST1H4D,HIST1H4E,HIST1H4F,HIST1H4I,HIST1H4J,HIST1H4K,HIST1H4L,HJURP,ITGB3BP,MIS18A,RUVBL1
mitotic cell cycle process	10	ANAPC1,ANAPC15,ANLN,ASPM,AURKA,AURKB,BIRC5,BLM,BORA,BUB1,BUB1B,BUB3,CASP2,CCNA2,CCNB1,CCNB2,CCNE2,CCNF,CCP110,CDC20,CDC25A,CDC25C,CDC45,CDC6,CDC7,CDCA2,CDCA3,CDCA5,CDCA8,CDK1,CDK2,CDKN2A,CDKN2B,CDKN2C,CDKN3,CDT1,CENPA,CENPE,CENPF,CENPH,CENPJ,CENPN,CENPW,CEP135,CEP152,CEP192,CEP55,CEP76,CEP78,CEP97,CHEK1,CIT,CKAP2,CKAP5,CKS1B,CKS2,CLSPN,CNTRL,DHFR,DHFRP1,DLGAP5,DNA2,DSCC1,DSN1,ERCC6L,ESPL1,EZH2,FAM64A,FANCI,FBXO5,FOXM1,GEN1,GINS1,GINS2,GPSM2,GSG2,GTSE1,HAUS4,HAUS8,HELLS,HMMR,INCENP,IQGAP3,ITGB3BP,KIF11,KIF14,KIF15,KIF18A,KIF18B,KIF20A,KIF20B,KIF22,KIF23,KIF2C,KIF4A,KIF4B,KIFC1,KNSTRN,KNTC1,LIG1,LMNB1,MAD2L1,MASTL,MCM10,MCM2,MCM3,MCM4,MCM5,MCM6,MCM7,MCM8,MCMBP,MELK,MIS18A,MSH2,MTBP,MYBL2,NCAPD2,NCAPD3,NCAPG,NCAPG2,NCAPH,NDC1,NDC80,NDE1,NEK2,NEK3,NOLC1,NUF2,NUP107,NUP153,NUP155,NUP160,NUP188,NUP205,NUP35,NUP43,NUP58,NUP85,NUP88,NUSAP1,ODF2,ORC1,ORC5,ORC6,PARP3,PBK,PCNA,PHB2,PKMYT1,PLK1,PLK4,POLA1,POLA2,POLE,POLE2,PRC1,PRIM1,PSRC1,PTTG1,RACGAP1,RANBP1,RBBP8,RCC1,REEP4,RFWD3,RRM2,RUVBL1,SGO1,SKA1,SKA2,SKA3,SLF1,SMC1A,SMC2,SMC3,SMC4,SPAG5,SPC24,SPC25,SPDL1,STIL,STMN1,TCF19,TICRR,TIMELESS,TIPIN,TOP2A,TPX2,TTK,TUBGCP3,TUBGCP4,TYMS,UBE2C,UBE2S,VRK1,WDR62,ZWILCH,ZWINT
positive regulation of mitotic nuclear division	10	AURKA,BIRC5,CDCA5,CENPE,DLGAP5,ESPL1,NUSAP1,PHB2,RANBP1,SLF1,UBE2C
peptidyl-lysine modification	10	ACTL6A,AURKB,BIRC5,BLM,BRCA1,BRCA2,CDCA8,CDKN2A,CHEK1,CTCF,DNMT1,EZH2,HAT1,HIST1H1B,HIST1H1C,HIST1H1D,HIST1H1E,INCENP,NDC1,NOP58,NUP107,NUP153,NUP155,NUP160,NUP188,NUP205,NUP35,NUP43,NUP58,NUP85,NUP88,PARP1,PAXIP1,PCNA,PHF19,POLE3,RUVBL1,SENP1,SMC1A,SMC3,SUV39H1,SUV39H2,TAF5,TOP2A,TRIM28,TRPM4
non-recombinational repair	10	BARD1,BRCA1,DCLRE1B,DCLRE1C,H2AFX,HIST1H4A,HIST1H4B,HIST1H4C,HIST1H4D,HIST1H4E,HIST1H4F,HIST1H4I,HIST1H4J,HIST1H4K,HIST1H4L,LIG1,MLH1,PAXIP1,POLA1,POLQ,RBBP8
DNA ligation	10	HMGB1,HMGB2,LIG1,PARP1,PARP2,PARP3,RAD51,TOP2A
telomere organization	10	ACD,AURKB,BLM,BRCA2,DCLRE1B,DCLRE1C,DNA2,FEN1,HIST1H3A,HIST1H3B,HIST1H3C,HIST1H3F,HIST1H3G,HIST1H3H,HIST1H3I,HIST1H3J,HIST1H4A,HIST1H4B,HIST1H4C,HIST1H4D,HIST1H4E,HIST1H4F,HIST1H4I,HIST1H4J,HIST1H4K,HIST1H4L,LIG1,NEK2,PARP1,PARP3,PCNA,POLA1,POLA2,POLD3,POLE,POLE2,PRIM1,RAD51,RFC2,RFC3,RFC4,TCP1
beta-catenin-TCF complex assembly	10	HIST1H4A,HIST1H4B,HIST1H4C,HIST1H4D,HIST1H4E,HIST1H4F,HIST1H4I,HIST1H4J,HIST1H4K,HIST1H4L,RUVBL1
protein-DNA complex assembly	10	ASF1A,ASF1B,CDC45,CENPA,CENPE,CENPF,CENPH,CENPI,CENPK,CENPL,CENPM,CENPN,CENPO,CENPP,CENPQ,CENPU,CENPW,CHAF1A,CHAF1B,DMC1,H2AFX,H2BFS,HAT1,HELLS,HIST1H1A,HIST1H1B,HIST1H1C,HIST1H1D,HIST1H1E,HIST1H2BB,HIST1H2BC,HIST1H2BD,HIST1H2BE,HIST1H2BF,HIST1H2BG,HIST1H2BH,HIST1H2BI,HIST1H2BK,HIST1H2BL,HIST1H2BM,HIST1H2BN,HIST1H2BO,HIST1H3A,HIST1H3B,HIST1H3C,HIST1H3F,HIST1H3G,HIST1H3H,HIST1H3I,HIST1H3J,HIST1H4A,HIST1H4B,HIST1H4C,HIST1H4D,HIST1H4E,HIST1H4F,HIST1H4I,HIST1H4J,HIST1H4K,HIST1H4L,HIST2H2BD,HIST2H2BE,HIST2H2BF,HIST2H3A,HIST2H3C,HIST2H3D,HJURP,HMGB1,HMGB2,ITGB3BP,MCM2,MIS18A,NASP,PARP1,RAD51,RUVBL1,XRCC2
nuclear division	10	ANAPC1,ANAPC15,ANLN,ASPM,AURKA,AURKB,BIRC5,BORA,BRCA2,BUB1,BUB1B,BUB3,CCNA2,CCNB1,CCNB2,CCNF,CDC20,CDC25A,CDC25C,CDC6,CDCA2,CDCA3,CDCA5,CDCA8,CDK1,CDK2,CENPE,CENPF,CENPH,CENPN,CENPW,CEP192,CEP55,CEP97,CHEK1,CIT,CKAP5,CKS2,DLGAP5,DMC1,DSCC1,DSN1,ERCC6L,ESPL1,FAM64A,FANCA,FANCD2,FANCM,FBXO5,GEN1,GSG2,HAUS4,HAUS8,HELLS,INCENP,ITGB3BP,KIF11,KIF14,KIF15,KIF18A,KIF18B,KIF20B,KIF22,KIF23,KIF2C,KIF4A,KIF4B,KIFC1,KNSTRN,KNTC1,MAD2L1,MASTL,MCMBP,MIS18A,MKI67,MLH1,MSH2,MSH6,MTBP,MYBL2,NCAPD2,NCAPD3,NCAPG,NCAPG2,NCAPH,NDC1,NDC80,NDE1,NEK2,NEK3,NOLC1,NUF2,NUP107,NUP153,NUP43,NUP88,NUSAP1,PBK,PDE3A,PHB2,PKMYT1,PLK1,PRC1,PSRC1,PTTG1,RACGAP1,RAD51,RAD54B,RAD54L,RANBP1,RBBP8,RCC1,REEP4,RUVBL1,SGO1,SKA1,SKA2,SKA3,SLF1,SMC1A,SMC2,SMC3,SMC4,SPAG5,SPC24,SPC25,SPDL1,STIL,TIMELESS,TIPIN,TOP2A,TOPBP1,TPX2,TRIP13,TTK,TUBGCP3,TUBGCP4,UBE2C,UBE2S,VRK1,XRCC2,ZWILCH,ZWINT

establishment of RNA localization	10	ALYREF,BUD13,CKAP5,DDX39A,GLE1,HNRNPA3,NDC1,NUP107,NUP153,NUP155,NUP160,NUP188,NUP205,NUP35,NUP43,NUP58,NUP85,NUP88,RANBP1,SRSF7,THOC3
regulation of mitotic sister chromatid separation	10	ANAPC15,BUB1,BUB1B,BUB3,CCNB1,CDC6,CENPE,CENPF,CEP192,CIT,DLGAP5,ESPL1,GEN1,MAD2L1,MTBP,PLK1,PTTG1,STIL,TTK,UBE2C
regulation of cell cycle	10	ANAPC1,ANAPC15,ANLN,ASPM,AURKA,AURKB,BARD1,BIRC5,BLM,BORA,BRCA1,BRCA2,BTG3,BUB1,BUB1B,BUB3,CASP2,CCNA2,CCNB1,CCNB2,CCNE2,CCNF,CCP110,CDC20,CDC25A,CDC25C,CDC45,CDC6,CDC7,CDCA5,CDK1,CDK2,CDKN2A,CDKN2B,CDKN2C,CDKN3,CDT1,CENPE,CENPF,CENPJ,CEP192,CEP76,CEP97,CHEK1,CHORDC1,CIT,CKS1B,CKS2,CLSPN,CTCF,DDIAS,DLGAP5,DTL,E2F8,ECT2,ESPL1,EZH2,FANCI,FBXO5,FIGNL1,FOXM1,GAS2L3,GEN1,GMNN,GSG2,GTSE1,HMGB1,KIF11,KIF14,KIF20B,KIF23,KIFC1,KNSTRN,KNTC1,LIN9,LMNB1,MAD2L1,MASTL,MSH2,MTBP,MYBL2,NEK2,NOTCH2,NUSAP1,ORC1,PARP3,PAXIP1,PCNA,PDE3A,PHB2,PKMYT1,PLK1,PLK4,PRR11,PSRC1,PTTG1,RACGAP1,RANBP1,RBL1,RBM14,RCC1,RFWD3,SLF1,SMC1A,SMC3,SPAG5,STIL,SUSD2,TACC3,TICRR,TIPIN,TOP2A,TPX2,TTK,UBE2C,ZWILCH,ZWINT
negative regulation of cell cycle process	10	ANAPC15,AURKA,AURKB,BLM,BRCA1,BUB1,BUB1B,BUB3,CASP2,CCNA2,CCNB1,CCNF,CDC25C,CDK1,CDK2,CDKN2B,CENPE,CENPF,CENPJ,CEP192,CHEK1,CLSPN,E2F8,ESPL1,EZH2,FANCI,FBXO5,GEN1,GSG2,GTSE1,KIFC1,MAD2L1,MSH2,MTBP,PCNA,PLK1,PTTG1,RBM14,RFWD3,SMC1A,SMC3,STIL,SUSD2,TICRR,TTK
meiotic cell cycle	10	ASPM,AURKA,BRCA2,BUB3,CDC20,CDK2,CKS2,DMC1,ESPL1,EXO1,FANCA,FANCD2,FANCM,FBXO5,H2AFX,KIF18A,MASTL,MKI67,MLH1,MND1,MSH2,MSH6,NCAPD2,NCAPD3,NDC1,NEK2,PDE3A,PLK1,PSMC3IP,PTTG1,RAD51,RAD54B,RAD54L,RBBP8,SGO1,SMC1A,SMC2,SMC3,SMC4,TOP2A,TOPBP1,TRIP13,TUBGCP3,TUBGCP4,XRCC2
regulation of nuclear division	10	ANAPC15,ANLN,AURKA,BIRC5,BORA,BUB1,BUB1B,BUB3,CCNB1,CDC20,CDC25C,CDC6,CDCA5,CENPE,CENPF,CEP192,CEP97,CHEK1,CIT,DLGAP5,ESPL1,FBXO5,GEN1,KIF11,KIF20B,KNTC1,MAD2L1,MSH2,MTBP,NEK2,NUSAP1,PDE3A,PHB2,PKMYT1,PLK1,PTTG1,RANBP1,RCC1,SLF1,STIL,TTK,UBE2C
centrosome separation	10	AURKA,CHEK1,KIF11,NDE1,NEK2,RANBP1
mRNA-containing ribonucleoprotein complex export from nucleus	10	ALYREF,BUD13,DDX39A,GLE1,NDC1,NUP107,NUP153,NUP155,NUP160,NUP188,NUP205,NUP35,NUP43,NUP58,NUP85,NUP88,SRSF7,THOC3
negative regulation of cell division	10	ANAPC15,ASPM,AURKB,BLM,BUB1,BUB1B,BUB3,CCNB1,CENPE,CENPF,CEP192,CHEK1,E2F8,FBXO5,GEN1,MAD2L1,MSH2,MTBP,PLK1,PTTG1,STIL,SUSD2,TTK
cytokinesis	10	ANLN,AURKA,AURKB,BIRC5,BRCA2,CCP110,CDC6,CEP55,CIT,CKAP2,E2F8,ECT2,ESPL1,INCENP,KIF14,KIF20A,KIF20B,KIF23,KIF4A,KIF4B,NUSAP1,PLK1,PRC1,RACGAP1,STMN1
regulation of chromosome segregation	10	ANAPC15,AURKB,BUB1,BUB1B,BUB3,CCNB1,CDC6,CDCA5,CENPE,CENPF,CEP192,CIT,CTCF,DLGAP5,ECT2,ESPL1,GEN1,KIF2C,KNSTRN,MAD2L1,MTBP,NEK2,PLK1,PTTG1,RACGAP1,RAD18,SLF1,SPAG5,STIL,TTK,UBE2C
chromosome localization	10	BIRC5,BRCA2,CCNB1,CDCA5,CDCA8,CENPE,CENPF,CEP55,DLGAP5,KIF14,KIF18A,KIF22,KIF2C,KIFC1,MLH1,NDE1,PSRC1,SPDL1
regulation of signal transduction by p53 class mediator	10	AURKA,AURKB,BARD1,BLM,BRCA1,BRIP1,CDK2,CDKN2A,CHEK1,DNA2,EXO1,RBBP8,RFC2,RFC3,RFC4,RMI1,SSRP1,TAF5,TOPBP1,TPX2
regulation of gene silencing	10	ASF1A,ATAD2,CDC45,CDK2,DNMT1,HIST1H3A,HIST1H3B,HIST1H3C,HIST1H3F,HIST1H3G,HIST1H3H,HIST1H3I,HIST1H3J,HIST2H3D,TRIM28,UHRF1
protein localization to kinetochore	10	AURKB,BUB1B,CDK1,GSG2,MTBP,SPDL1
regulation of mitotic cell cycle phase transition	10	ANAPC1,ANAPC15,ANLN,AURKA,BIRC5,BLM,BUB1,BUB1B,BUB3,CASP2,CCNA2,CCNB1,CDC20,CDC25C,CDC45,CDC6,CDC7,CDCA5,CDK1,CDK2,CDKN2A,CDKN2B,CENPE,CENPF,CENPJ,CEP192,CHEK1,CLSPN,DLGAP5,ESPL1,EZH2,FANCI,FBXO5,GEN1,GSG2,GTSE1,KIF14,KNTC1,LMNB1,MAD2L1,MTBP,PCNA,PHB2,PLK1,RFWD3,STIL,TICRR,TTK,UBE2C
microtubule polymerization or depolymerization	10	CENPJ,CEP192,CKAP2,CLIP3,FBXO5,KIF14,KIF18A,KIF18B,KIF24,KIF2C,NDE1,PSRC1,SKA1,SKA2,SKA3,STMN1,TUBGCP3,TUBGCP4
DNA strand elongation	10	DCLRE1B,DNA2,FEN1,GINS1,GINS2,GINS3,GINS4,LIG1,PARP1,PARP2,PARP3,PCNA,POLA1,POLA2,POLD3,PRIM1,RFC3,RFC4,RNASEH2A
negative regulation of cellular protein catabolic process	10	ANAPC15,BUB1,BUB1B,BUB3,CCNB1,CDKN2A,CENPE,CENPF,CEP192,GEN1,MAD2L1,MTBP,PBK,PLK1,SENP1,STIL,TTK
mRNA export from nucleus	10	ALYREF,BUD13,DDX39A,GLE1,NDC1,NUP107,NUP153,NUP155,NUP160,NUP188,NUP205,NUP35,NUP43,NUP58,NUP85,NUP88,SRSF7,THOC3
cytoskeleton organization	10	ANLN,ASPM,AUNIP,AURKA,AURKB,BIRC5,BORA,BRCA1,BRCA2,CCNB1,CCNF,CCP110,CDC42EP3,CDK1,CDK2,CENPA,CENPE,CENPJ,CEP135,CEP152,CEP192,CEP76,CEP97,CHEK1,CHORDC1,CIT,CKAP2,CKAP5,CLIP3,CNTNAP1,DIAPH3,ECT2,EMP2,ESPL1,FBXO5,FIGNL1,GAS2L3,GEN1,GPSM2,GSN,HAUS4,HAUS8,HSPH1,ID1,KIF11,KIF14,KIF18A,KIF18B,KIF20A,KIF23,KIF24,KIF2C,KIF4A,KIF4B,KIFC1,KNSTRN,KRT19,MLH1,MYBL2,NDC1,NDC80,NDE1,NEK2,NUSAP1,PARP3,PLK1,PLK4,PRC1,PSRC1,RACGAP1,RANBP1,RBM14,RCC1,SASS6,SDC4,SGO1,SKA1,SKA2,SKA3,SMC1A,SMC3,SPAG5,SPC25,SPDL1,STIL,STMN1,TACC3,TMSB15A,TPX2,TTK,TUBGCP3,TUBGCP4,WDR62,XRCC2
regulation of DNA repair	10	BRCA1,CHEK1,FIGNL1,FOXM1,H2AFX,HMGB1,PARP1,PARPBP,PCNA,POLQ,RAD51,RAD51AP1,SLF1,TRIM28,USP1
negative regulation of mitotic nuclear division	10	ANAPC15,BUB1,BUB1B,BUB3,CCNB1,CENPE,CENPF,CEP192,CHEK1,GEN1,MAD2L1,MTBP,PLK1,PTTG1,STIL,TTK
chromatin organization	10	ACTL6A,ANP32E,ASF1A,ASF1B,ATAD2,AURKA,AURKB,BRCA1,BRCA2,CCNB1,CDC45,CDK1,CDK2,CDKN2A,CENPA,CENPH,CENPI,CENPK,CENPL,CENPM,CENPN,CENPO,CENPP,CENPQ,CENPU,CENPW,CHAF1A,CHAF1B,CHEK1,CTCF,DNMT1,EZH2,FBL,GSG2,H2AFX,H2AFZ,H2BFS,HAT1,HELLS,HIST1H1A,HIST1H1B,HIST1H1C,HIST1H1D,HIST1H1E,HIST1H2AB,HIST1H2AC,HIST1H2AD,HIST1H2AE,HIST1H2AG,HIST1H2AH,HIST1H2AI,HIST1H2AJ,HIST1H2AK,HIST1H2AL,HIST1H2AM,HIST1H2BB,HIST1H2BC,HIST1H2BD,HIST1H2BE,HIST1H2BF,HIST1H2BG,HIST1H2BH,HIST1H2BI,HIST1H2BK,HIST1H2BL,HIST1H2BM,HIST1H2BN,HIST1H2BO,HIST1H3A,HIST1H3B,HIST1H3C,HIST1H3F,HIST1H3G,HIST1H3H,HIST1H3I,HIST1H3J,HIST1H4A,HIST1H4B,HIST1H4C,HIST1H4D,HIST1H4E,HIST1H4F,HIST1H4I,HIST1H4J,HIST1H4K,HIST1H4L,HIST2H2AA3,HIST2H2AA4,HIST2H2AB,HIST2H2AC,HIST2H2BD,HIST2H2BE,HIST2H2BF,HIST2H3A,HIST2H3C,HIST2H3D,HJURP,HMGB1,HMGB2,HMGN3,ITGB3BP,MCM2,MIS18A,NASP,PAXIP1,PHF19,POLE3,RBL1,RBM14,RUVBL1,SUV39H1,SUV39H2,TAF5,TRIM28,UHRF1,VRK1,WDHD1
tRNA transport	10	NDC1,NUP107,NUP153,NUP155,NUP160,NUP188,NUP205,NUP35,NUP43,NUP58,NUP85,NUP88
regulation of attachment of spindle microtubules to kinetochore	10	CCNB1,CENPE,ECT2,KNSTRN,NEK2,RACGAP1,SPAG5
regulation of cell cycle phase transition	10	ANAPC1,ANAPC15,ANLN,AURKA,BIRC5,BLM,BUB1,BUB1B,BUB3,CASP2,CCNA2,CCNB1,CDC20,CDC25C,CDC45,CDC6,CDC7,CDCA5,CDK1,CDK2,CDKN2A,CDKN2B,CENPE,CENPF,CENPJ,CEP192,CHEK1,CLSPN,DLGAP5,ESPL1,EZH2,FANCI,FBXO5,GEN1,GSG2,GTSE1,KIF14,KNTC1,LMNB1,MAD2L1,MTBP,PAXIP1,PCNA,PHB2,PLK1,RFWD3,STIL,SUSD2,TICRR,TTK,UBE2C
protein heterotetramerization	10	HIST1H3A,HIST1H3B,HIST1H3C,HIST1H3F,HIST1H3G,HIST1H3H,HIST1H3I,HIST1H3J,HIST1H4A,HIST1H4B,HIST1H4C,HIST1H4D,HIST1H4E,HIST1H4F,HIST1H4I,HIST1H4J,HIST1H4K,HIST1H4L,HIST2H3D,NUP58,RRM1,RRM2

negative regulation of proteolysis involved in cellular protein catabolic process	10	ANAPC15,BUB1,BUB1B,BUB3,CCNB1,CDKN2A,CENPE,CENPF,CEP192,GEN1,MAD2L1,MTBP,PBK,PLK1,SENP1,STIL,TTK
cell cycle DNA replication	10	CDC45,CDC7,CDT1,DNA2,E2F8,GINS1,GINS2,LIG1,PCNA,SMC1A,SMC3,TIPIN
tRNA-containing ribonucleoprotein complex export from nucleus	10	NDC1,NUP107,NUP153,NUP155,NUP160,NUP188,NUP205,NUP35,NUP43,NUP58,NUP85,NUP88
meiotic chromosome segregation	10	BRCA2,DMC1,ESPL1,FANCD2,FANCM,MLH1,NDC1,PLK1,PTTG1,SGO1,SMC2,SMC4,TOP2A,TRIP13
regulation of cyclin-dependent protein kinase activity	10	BLM,CCNA2,CCNE2,CDC25A,CDC25C,CDC6,CDKN2A,CDKN2B,CDKN2C,CDKN3,CKS1B,CKS2,LMNB1,PKMYT1,PLK1,PSRC1,STIL,UBE2C
regulation of cellular response to heat	10	CHORDC1,HSPH1,NDC1,NUP107,NUP153,NUP155,NUP160,NUP188,NUP205,NUP35,NUP43,NUP58,NUP85,NUP88
microtubule organizing center organization	10	AURKA,BRCA1,BRCA2,CCNF,CCP110,CDK1,CDK2,CENPJ,CEP135,CEP152,CEP192,CEP76,CHEK1,CHORDC1,CKAP5,GEN1,HAUS4,HAUS8,KIF11,KIFC1,NDC1,NDE1,NEK2,PLK1,PLK4,RANBP1,RBM14,SASS6,SGO1,STIL,TUBGCP3,TUBGCP4,WDR62,XRCC2
nucleic acid transport	10	ALYREF,BUD13,CKAP5,DDX39A,GLE1,HNRNPA3,NDC1,NUP107,NUP153,NUP155,NUP160,NUP188,NUP205,NUP35,NUP43,NUP58,NUP85,NUP88,RANBP1,SRSF7,THOC3
negative regulation of cell cycle	10	ANAPC15,AURKA,AURKB,BARD1,BLM,BRCA1,BTG3,BUB1,BUB1B,BUB3,CASP2,CCNA2,CCNB1,CCNF,CDC25C,CDK1,CDK2,CDKN2A,CDKN2B,CDKN2C,CDKN3,CENPE,CENPF,CENPJ,CEP192,CHEK1,CLSPN,DDIAS,E2F8,ESPL1,EZH2,FANCI,FBXO5,FOXM1,GAS2L3,GEN1,GMNN,GSG2,GTSE1,KIF20B,KIFC1,KNTC1,MAD2L1,MSH2,MTBP,NOTCH2,PCNA,PLK1,PTTG1,RBM14,RFWD3,SMC1A,SMC3,STIL,SUSD2,TICRR,TIPIN,TOP2A,TTK,UBE2C,ZWILCH,ZWINT
centromere complex assembly	10	CENPA,CENPE,CENPF,CENPH,CENPI,CENPK,CENPL,CENPM,CENPN,CENPO,CENPP,CENPQ,CENPU,CENPW,HELLS,HIST1H4A,HIST1H4B,HIST1H4C,HIST1H4D,HIST1H4E,HIST1H4F,HIST1H4I,HIST1H4J,HIST1H4K,HIST1H4L,HJURP,ITGB3BP,MIS18A,RUVBL1
positive regulation of gene expression, epigenetic	10	ASF1A,ATAD2,CHEK1,HIST1H3A,HIST1H3B,HIST1H3C,HIST1H3F,HIST1H3G,HIST1H3H,HIST1H3I,HIST1H3J,HIST1H4A,HIST1H4B,HIST1H4C,HIST1H4D,HIST1H4E,HIST1H4F,HIST1H4I,HIST1H4J,HIST1H4K,HIST1H4L,HIST2H3A,HIST2H3C,HIST2H3D
DNA replication-independent nucleosome assembly	10	ASF1A,ASF1B,CENPA,CENPH,CENPI,CENPK,CENPL,CENPM,CENPN,CENPO,CENPP,CENPQ,CENPU,CENPW,HAT1,HIST1H4A,HIST1H4B,HIST1H4C,HIST1H4D,HIST1H4E,HIST1H4F,HIST1H4I,HIST1H4J,HIST1H4K,HIST1H4L,HJURP,ITGB3BP,MIS18A,NASP,RUVBL1
negative regulation of sister chromatid segregation	10	ANAPC15,BUB1,BUB1B,BUB3,CCNB1,CENPE,CENPF,CEP192,ESPL1,GEN1,MAD2L1,MTBP,PLK1,PTTG1,STIL,TTK
DNA biosynthetic process	10	ACD,AURKB,BARD1,BLM,BRCA1,BRCA2,BRIP1,CENPF,CHEK1,DNA2,DSCC1,DTL,EXO1,LIG1,LIN9,NEK2,PCNA,POLA1,POLA2,POLD3,POLE,POLE2,POLE3,POLQ,RAD51,RAD51AP1,RBBP8,RFC2,RFC3,RFC4,RMI1,TCP1,TK1,TYMS,XRCC2
chromatin silencing at rDNA	10	HIST1H3A,HIST1H3B,HIST1H3C,HIST1H3F,HIST1H3G,HIST1H3H,HIST1H3I,HIST1H3J,HIST1H4A,HIST1H4B,HIST1H4C,HIST1H4D,HIST1H4E,HIST1H4F,HIST1H4I,HIST1H4J,HIST1H4K,HIST1H4L,HIST2H3A,HIST2H3C,HIST2H3D,SUV39H1
reciprocal DNA recombination	10	DMC1,FANCM,MLH1,MSH2,MSH6,RAD51,RAD54B,TOP2A,TOPBP1,TRIP13,XRCC2
mitotic cytokinesis	10	ANLN,CEP55,CKAP2,KIF20A,KIF20B,KIF23,KIF4A,KIF4B,NUSAP1,PLK1,RACGAP1,STMN1
base-excision repair	10	DNA2,FEN1,HMGB1,LIG1,NEIL3,PARP1,PARP2,PCNA,POLE,POLQ,UNG
nuclear DNA replication	10	CDC45,CDC7,CDT1,DNA2,GINS1,GINS2,LIG1,PCNA,TIPIN
nucleosome assembly	10	ASF1A,ASF1B,CENPA,CENPH,CENPI,CENPK,CENPL,CENPM,CENPN,CENPO,CENPP,CENPQ,CENPU,CENPW,CHAF1A,CHAF1B,H2AFX,H2BFS,HAT1,HIST1H1A,HIST1H1B,HIST1H1C,HIST1H1D,HIST1H1E,HIST1H2BB,HIST1H2BC,HIST1H2BD,HIST1H2BE,HIST1H2BF,HIST1H2BG,HIST1H2BH,HIST1H2BI,HIST1H2BK,HIST1H2BL,HIST1H2BM,HIST1H2BN,HIST1H2BO,HIST1H3A,HIST1H3B,HIST1H3C,HIST1H3F,HIST1H3G,HIST1H3H,HIST1H3I,HIST1H3J,HIST1H4A,HIST1H4B,HIST1H4C,HIST1H4D,HIST1H4E,HIST1H4F,HIST1H4I,HIST1H4J,HIST1H4K,HIST1H4L,HIST2H2BD,HIST2H2BE,HIST2H2BF,HIST2H3A,HIST2H3C,HIST2H3D,HJURP,HMGB2,ITGB3BP,MCM2,MIS18A,NASP,RUVBL1
organelle assembly	10	ANLN,ASPM,AURKA,AURKB,BIRC5,BRCA1,CCP110,CDK2,CENPA,CENPE,CENPF,CENPH,CENPJ,CENPW,CEP135,CEP152,CEP192,CEP295,CEP76,CEP97,FBXO5,GSN,HAUS4,HAUS8,HSPB11,KIAA0586,KIF11,KIF23,KIF24,KIF4A,KIF4B,KIFC1,KRT19,LSM3,MLH1,MRTO4,MYBL2,NEK2,ODF2,PLK1,PLK4,RACGAP1,RBM14,RCC1,RPL23A,SASS6,SCLT1,SDC4,TMEM107,TPX2,TRIM59,TUBGCP3,TUBGCP4,WDR62
meiotic cell cycle process	10	ASPM,AURKA,BRCA2,CDC20,CDK2,CKS2,DMC1,ESPL1,FANCA,FANCD2,FANCM,FBXO5,KIF18A,MASTL,MKI67,MLH1,MSH2,MSH6,NCAPD2,NCAPD3,NDC1,PDE3A,PLK1,PTTG1,RAD51,RAD54B,RAD54L,SGO1,SMC1A,SMC2,SMC3,SMC4,TOP2A,TOPBP1,TRIP13,TUBGCP3,TUBGCP4,XRCC2
chromatin assembly	10	ASF1A,ASF1B,CDKN2A,CENPA,CENPH,CENPI,CENPK,CENPL,CENPM,CENPN,CENPO,CENPP,CENPQ,CENPU,CENPW,CHAF1A,CHAF1B,CTCF,H2AFX,H2BFS,HAT1,HELLS,HIST1H1A,HIST1H1B,HIST1H1C,HIST1H1D,HIST1H1E,HIST1H2BB,HIST1H2BC,HIST1H2BD,HIST1H2BE,HIST1H2BF,HIST1H2BG,HIST1H2BH,HIST1H2BI,HIST1H2BK,HIST1H2BL,HIST1H2BM,HIST1H2BN,HIST1H2BO,HIST1H3A,HIST1H3B,HIST1H3C,HIST1H3F,HIST1H3G,HIST1H3H,HIST1H3I,HIST1H3J,HIST1H4A,HIST1H4B,HIST1H4C,HIST1H4D,HIST1H4E,HIST1H4F,HIST1H4I,HIST1H4J,HIST1H4K,HIST1H4L,HIST2H2BD,HIST2H2BE,HIST2H2BF,HIST2H3A,HIST2H3C,HIST2H3D,HJURP,HMGB1,HMGB2,ITGB3BP,MCM2,MIS18A,NASP,RUVBL1
megakaryocyte differentiation	10	CDKN2B,HIST1H4A,HIST1H4B,HIST1H4C,HIST1H4D,HIST1H4E,HIST1H4F,HIST1H4I,HIST1H4J,HIST1H4K,HIST1H4L,HMGB2
nuclear lamina	10	LBR,LMNB1,LMNB2,NRM,NUP35,PCNA,SUV39H1
condensed chromosome, centromeric region	10	AURKA,AURKB,BIRC5,BUB1,BUB1B,BUB3,CCNB1,CENPA,CENPE,CENPF,CENPH,CENPK,CENPM,CENPN,CENPO,CENPU,CENPW,DCTN5,DSN1,ERCC6L,HJURP,INCENP,ITGB3BP,KIF2C,KNSTRN,KNTC1,MAD2L1,NCAPD2,NCAPD3,NDC80,NDE1,NEK2,NUF2,NUP107,NUP43,NUP85,PLK1,SGO1,SKA1,SKA2,SKA3,SMC1A,SPAG5,SPC24,SPC25,SPDL1,ZWILCH,ZWINT
chromosome, centromeric region	10	AURKA,AURKB,BIRC5,BUB1,BUB1B,BUB3,CCNB1,CDCA5,CDCA8,CENPA,CENPE,CENPF,CENPH,CENPI,CENPK,CENPL,CENPM,CENPN,CENPO,CENPP,CENPQ,CENPU,CENPW,CTCF,DCTN5,DNMT1,DSCC1,DSN1,ERCC6L,ESCO2,HELLS,HJURP,INCENP,ITGB3BP,KIF18A,KIF22,KIF2C,KNSTRN,KNTC1,MAD2L1,MIS18A,MKI67,MTBP,NCAPD2,NCAPD3,NDC80,NDE1,NEK2,NUF2,NUP107,NUP160,NUP43,NUP85,PLK1,SGO1,SKA1,SKA2,SKA3,SMC1A,SMC3,SPAG5,SPC24,SPC25,SPDL1,SUV39H1,SUV39H2,WDHD1,ZWILCH,ZWINT
condensed chromosome outer kinetochore	10	BUB1,BUB1B,CCNB1,CENPE,CENPF,NDC80,PLK1,SKA1,SKA2,SKA3,SPDL1
condensed nuclear chromosome, centromeric region	10	AURKA,AURKB,BUB1,BUB1B,CCNB1,CENPA,CENPE,NDC80,PLK1,SGO1

spindle	10	ASPM,AUNIP,AURKA,AURKB,BIRC5,BORA,BUB1B,CCNB1,CDC20,CDC6,CDCA8,CDK1,CENPE,CENPF,CEP128,CKAP2,CKAP2L,CKAP5,CNTRL,DDX11,DLGAP5,ECT2,ESPL1,FBXO5,GPSM2,GSG2,HAUS4,HAUS8,INCENP,KIF11,KIF14,KIF15,KIF18A,KIF18B,KIF20A,KIF20B,KIF22,KIF23,KIF4A,KIFC1,KNSTRN,KNTC1,MAD2L1,NDC1,NDE1,NEK2,NUP85,NUSAP1,ODF2,PLK1,POC1A,PRC1,PSRC1,RACGAP1,SGO1,SKA1,SKA2,SKA3,SMC3,SPAG5,SPDL1,TOPBP1,TPX2,TTK,TUBGCP3,TUBGCP4,VRK1,WDR62
microtubule cytoskeleton	10	ASPM,AUNIP,AURKA,AURKB,BIRC5,BORA,BRCA1,BRCA2,BUB1B,CCDC15,CCDC77,CCNB1,CCNB2,CCNF,CCP110,CDC20,CDC45,CDC6,CDC7,CDCA8,CDK1,CDK2,CENPE,CENPF,CENPJ,CENPU,CEP128,CEP135,CEP152,CEP192,CEP295,CEP55,CEP76,CEP78,CEP97,CFAP20,CHEK1,CKAP2,CKAP2L,CKAP5,CNTRL,DCLRE1B,DCTN5,DDX11,DLGAP5,DTL,ECT2,ERCC6L,ESPL1,FBXO5,GAS2L3,GEN1,GPSM2,GSG2,GTSE1,HAUS4,HAUS8,HSPB11,HSPH1,HYLS1,ID1,INCENP,KIAA0586,KIF11,KIF14,KIF15,KIF18A,KIF18B,KIF20A,KIF20B,KIF22,KIF23,KIF24,KIF2C,KIF4A,KIF4B,KIFC1,KNSTRN,KNTC1,MAD2L1,MASTL,MCM2,MCM3,MPHOSPH9,NCAPG,NDC1,NDE1,NEK2,NEK3,NUP107,NUP85,NUSAP1,ODF2,PARP3,PCNA,PLK1,PLK4,POC1A,POC5,PRC1,PSRC1,RACGAP1,RAD18,RAD51,RANBP1,REEP4,RTTN,RUVBL1,SASS6,SCLT1,SGO1,SKA1,SKA2,SKA3,SLF1,SMC3,SPAG5,SPDL1,STIL,STMN1,TACC3,TCP1,TIMELESS,TOP2A,TOPBP1,TPX2,TRIM59,TTK,TUBD1,TUBGCP3,TUBGCP4,VRK1,WDR34,WDR62,XRCC2
replication fork	10	BLM,CDC45,CHEK1,DNMT1,GINS2,GINS4,H2AFX,MCM10,MCM3,MMS22L,PCNA,POLA1,POLA2,POLD3,POLE,POLE2,POLE3,PRIM1,RAD18,RFC2,RFC3,RFC4,UHRF1,XRCC2
nuclear heterochromatin	10	EME1,ESCO2,H2AFZ,HIST1H1B,HIST1H1E,NCAPD3,SUV39H1,SUV39H2,TCP1,TRIM28,UHRF1
heterochromatin	10	CDKN2A,DNMT1,EME1,ESCO2,H2AFZ,HELLS,HIST1H1B,HIST1H1E,INCENP,NCAPD3,SUV39H1,SUV39H2,TCP1,TRIM28,UHRF1
spindle midzone	10	AURKA,AURKB,BUB1B,CDC6,CDCA8,CENPE,KIF14,KIF18A,KIF20B,PLK1,RACGAP1
MCM complex	10	MCM2,MCM3,MCM4,MCM5,MCM6,MCM7,MCM8,MCMBP,MMS22L
polymeric cytoskeletal fiber	10	ASPM,AURKA,AURKB,BIRC5,CDK1,CENPE,CENPJ,CFAP20,CKAP2,CKAP5,GAS2L3,GTSE1,HAUS4,HAUS8,HSPH1,INCENP,KIF11,KIF14,KIF15,KIF18A,KIF18B,KIF20A,KIF20B,KIF22,KIF23,KIF24,KIF2C,KIF4A,KIF4B,KIFC1,KNSTRN,KNTC1,KRT15,KRT19,LMNB1,LMNB2,NDE1,NEK2,NUSAP1,ODF2,PLK1,PRC1,PSRC1,RACGAP1,REEP4,SKA1,SKA2,SKA3,SPAG5,STMN1,TCP1,TPX2,TUBD1,TUBGCP3,TUBGCP4
nuclear envelope	10	AGPAT5,BRIP1,CACYBP,CENPF,CSE1L,DCTN5,DTL,GLE1,KPNA2,LBR,LMNB1,LMNB2,MAD2L1,MRTO4,NDC1,NEMP1,NRM,NUP107,NUP153,NUP155,NUP160,NUP188,NUP205,NUP35,NUP43,NUP58,NUP85,NUP88,PARP1,POLA1,RANBP1,RCC1,RRM1,RRM2,SENP1,SEPHS1,TMEM109,TMEM97,TMPO,TRA2B
kinesin complex	10	CENPE,KIF11,KIF14,KIF15,KIF18A,KIF18B,KIF20A,KIF20B,KIF22,KIF23,KIF24,KIF2C,KIF4A,KIF4B,KIFC1,NDE1
condensin complex	10	NCAPD2,NCAPD3,NCAPG,NCAPH,SMC2,SMC4
mitotic spindle	10	ASPM,AURKA,AURKB,CDK1,CENPE,CNTRL,ECT2,ESPL1,GPSM2,KIF11,KIF18A,KIF20B,KIF22,KIF23,KIFC1,KNSTRN,MAD2L1,RACGAP1,SPAG5,TUBGCP4
centrosome	10	AUNIP,AURKA,AURKB,BIRC5,BRCA1,BRCA2,CCDC15,CCDC77,CCNB1,CCNB2,CCNF,CCP110,CDC20,CDC45,CDK1,CDK2,CENPF,CENPJ,CEP128,CEP135,CEP152,CEP192,CEP295,CEP55,CEP76,CEP78,CEP97,CFAP20,CHEK1,CKAP2,CKAP2L,CKAP5,CNTRL,DCLRE1B,DCTN5,DLGAP5,DTL,ERCC6L,ESPL1,GEN1,GSG2,HAUS4,HAUS8,HSPB11,HYLS1,ID1,KIAA0586,KIF15,KIF20B,KIF23,KIF24,KIFC1,MASTL,MCM3,MPHOSPH9,NCAPG,NDE1,NEK2,NEK3,NUP107,ODF2,PARP3,PCNA,PLK1,PLK4,POC1A,POC5,RAD18,RANBP1,RTTN,SASS6,SCLT1,SGO1,SLF1,SPAG5,STIL,TACC3,TCP1,TOP2A,TOPBP1,TRIM59,TUBD1,TUBGCP3,TUBGCP4,WDR34,WDR62,XRCC2
chromatin	10	ACTL6A,ANP32E,ASF1A,ASF1B,CDCA5,CDKN2A,CENPA,CENPF,CHAF1A,CHAF1B,CHEK1,DDX11,DNMT1,DSCC1,EME1,ESCO2,EZH2,FAM111A,FANCC,H2AFX,H2AFZ,H2BFS,HAT1,HELLS,HIST1H1A,HIST1H1B,HIST1H1C,HIST1H1D,HIST1H1E,HIST1H2AB,HIST1H2AC,HIST1H2AD,HIST1H2AE,HIST1H2AG,HIST1H2AH,HIST1H2AI,HIST1H2AJ,HIST1H2AK,HIST1H2AL,HIST1H2AM,HIST1H2BB,HIST1H2BC,HIST1H2BD,HIST1H2BE,HIST1H2BF,HIST1H2BG,HIST1H2BH,HIST1H2BI,HIST1H2BK,HIST1H2BL,HIST1H2BM,HIST1H2BN,HIST1H2BO,HIST1H3A,HIST1H3B,HIST1H3C,HIST1H3F,HIST1H3G,HIST1H3H,HIST1H3I,HIST1H3J,HIST1H4A,HIST1H4B,HIST1H4C,HIST1H4D,HIST1H4E,HIST1H4F,HIST1H4I,HIST1H4J,HIST1H4K,HIST1H4L,HIST2H2AA3,HIST2H2AA4,HIST2H2AB,HIST2H2AC,HIST2H2BD,HIST2H2BE,HIST2H2BF,HIST2H3A,HIST2H3C,HIST2H3D,HMGB2,HMGN2,HMGN3,INCENP,KIF22,MCM2,MCM7,MSH6,MTBP,NASP,NCAPD3,PARPBP,PLK1,POLA1,RAD18,RAD51,RBMX,RCC1,RUVBL1,SLF1,SMC3,SUV39H1,SUV39H2,TCP1,TIMELESS,TIPIN,TMPO,TRIM28,UHRF1
chromosomal part	10	ACD,ACTL6A,ALYREF,ANP32E,ASF1A,ASF1B,AURKA,AURKB,BIRC5,BLM,BRCA2,BUB1,BUB1B,BUB3,CCNB1,CDC45,CDCA5,CDCA8,CDK1,CDK2,CDKN2A,CENPA,CENPE,CENPF,CENPH,CENPK,CENPL,CENPM,CENPN,CENPO,CENPP,CENPQ,CENPU,CENPW,CHAF1A,CHAF1B,CHEK1,CTCF,DCLRE1B,DCLRE1C,DCTN5,DDX11,DMC1,DNA2,DNMT1,DSCC1,DSN1,EME1,ERCC6L,ESCO2,EZH2,FAM111A,FANCC,FEN1,GINS1,GINS2,GINS4,H2AFX,H2AFZ,H2BFS,HAT1,HELLS,HIST1H1A,HIST1H1B,HIST1H1C,HIST1H1D,HIST1H1E,HIST1H2AB,HIST1H2AC,HIST1H2AD,HIST1H2AE,HIST1H2AG,HIST1H2AH,HIST1H2AI,HIST1H2AJ,HIST1H2AK,HIST1H2AL,HIST1H2AM,HIST1H2BB,HIST1H2BC,HIST1H2BD,HIST1H2BE,HIST1H2BF,HIST1H2BG,HIST1H2BH,HIST1H2BI,HIST1H2BK,HIST1H2BL,HIST1H2BM,HIST1H2BN,HIST1H2BO,HIST1H3A,HIST1H3B,HIST1H3C,HIST1H3F,HIST1H3G,HIST1H3H,HIST1H3I,HIST1H3J,HIST1H4A,HIST1H4B,HIST1H4C,HIST1H4D,HIST1H4E,HIST1H4F,HIST1H4I,HIST1H4J,HIST1H4K,HIST1H4L,HIST2H2AA3,HIST2H2AA4,HIST2H2AB,HIST2H2AC,HIST2H2BD,HIST2H2BE,HIST2H2BF,HIST2H3A,HIST2H3C,HIST2H3D,HJURP,HMGB2,HMGN2,HMGN3,INCENP,ITGB3BP,KIF18A,KIF22,KIF2C,KNSTRN,KNTC1,MAD2L1,MCM10,MCM2,MCM3,MCM4,MCM5,MCM6,MCM7,MIS18A,MKI67,MLH1,MMS22L,MSH2,MSH6,MTBP,MYBL2,NASP,NCAPD2,NCAPD3,NCAPG,NCAPH,NDC80,NDE1,NEK2,NUF2,NUP107,NUP160,NUP43,NUP85,ORC1,ORC5,ORC6,PARP1,PARP3,PARPBP,PCNA,PLK1,POLA1,POLA2,POLD3,POLE,POLE2,POLE3,PRIM1,RAD18,RAD51,RBMX,RCC1,RFC2,RFC3,RFC4,RFWD3,RUVBL1,SGO1,SKA1,SKA2,SKA3,SLF1,SMC1A,SMC2,SMC3,SMC4,SMCHD1,SPAG5,SPC24,SPC25,SPDL1,SUV39H1,SUV39H2,TCP1,THOC3,TIMELESS,TIPIN,TMPO,TRIM28,UHRF1,WDHD1,XRCC2,ZWILCH,ZWINT
nuclear replication fork	10	CDC45,GINS2,GINS4,MCM10,MCM3,MMS22L,PCNA,POLA1,POLA2,POLD3,POLE,POLE2,POLE3
chromosome, telomeric region	10	ACD,ALYREF,BLM,BRCA2,CDK1,CDK2,CHEK1,DCLRE1B,DCLRE1C,DMC1,DNA2,FEN1,H2AFX,HAT1,HIST1H2BB,HIST1H3A,HIST1H3B,HIST1H3C,HIST1H3F,HIST1H3G,HIST1H3H,HIST1H3I,HIST1H3J,HIST1H4A,HIST1H4B,HIST1H4C,HIST1H4D,HIST1H4E,HIST1H4F,HIST1H4I,HIST1H4J,HIST1H4K,HIST1H4L,HIST2H3D,MCM2,MCM3,MCM4,MCM5,MCM6,MCM7,MSH2,ORC1,ORC5,PARP1,PCNA,RAD51,SMCHD1,THOC3
supramolecular fiber	10	ASPM,AURKA,AURKB,BIRC5,CDK1,CENPE,CENPJ,CFAP20,CKAP2,CKAP5,GAS2L3,GTSE1,HAUS4,HAUS8,HSPH1,INCENP,KIF11,KIF14,KIF15,KIF18A,KIF18B,KIF20A,KIF20B,KIF22,KIF23,KIF24,KIF2C,KIF4A,KIF4B,KIFC1,KNSTRN,KNTC1,KRT15,KRT19,LMNB1,LMNB2,NDE1,NEK2,NUSAP1,ODF2,PLK1,PRC1,PSRC1,RACGAP1,REEP4,SKA1,SKA2,SKA3,SPAG5,STMN1,TCP1,TPX2,TUBD1,TUBGCP3,TUBGCP4
nuclear chromosome	10	ACD,ACTL6A,ALYREF,ANP32E,ASF1A,ASF1B,AURKA,AURKB,BIRC5,BLM,BRCA1,BRCA2,BUB1,BUB1B,CCNB1,CDC45,CDCA5,CDK1,CENPA,CENPE,CHAF1A,CHAF1B,CHEK1,DCLRE1B,DCLRE1C,DDX11,DMC1,DNA2,EME1,ESCO2,EXOSC9,EZH2,FEN1,FIGNL1,GINS1,GINS2,GINS4,H2AFX,H2AFZ,H2BFS,HAT1,HIST1H1A,HIST1H1B,HIST1H1C,HIST1H1D,HIST1H1E,HIST1H2AB,HIST1H2AC,HIST1H2AD,HIST1H2AE,HIST1H2AG,HIST1H2AH,HIST1H2AI,HIST1H2AJ,HIST1H2AK,HIST1H2AL,HIST1H2AM,HIST1H2BB,HIST1H2BC,HIST1H2BD,HIST1H2BE,HIST1H2BF,HIST1H2BG,HIST1H2BH,HIST1H2BI,HIST1H2BK,HIST1H2BL,HIST1H2BM,HIST1H2BN,HIST1H2BO,HIST1H3A,HIST1H3B,HIST1H3C,HIST1H3F,HIST1H3G,HIST1H3H,HIST1H3I,HIST1H3J,HIST1H4A,HIST1H4B,HIST1H4C,HIST1H4D,HIST1H4E,HIST1H4F,HIST1H4I,HIST1H4J,HIST1H4K,HIST1H4L,HIST2H2AA3,HIST2H2AA4,HIST2H2AB,HIST2H2AC,HIST2H2BD,HIST2H2BE,HIST2H2BF,HIST2H3D,HMGB2,INCENP,MCM10,MCM2,MCM3,MCM4,MCM5,MCM6,MCM7,MLH1,MMS22L,MSH2,MSH6,NASP,NCAPD2,NCAPD3,NDC80,NEK2,ORC1,ORC5,ORC6,PARP1,PCNA,PLK1,POLA1,POLA2,POLD3,POLE,POLE2,POLE3,RAD51,RBMX,RCC1,RUVBL1,SGO1,SMC1A,SMC2,SMC3,SMCHD1,SUV39H1,SUV39H2,TCP1,THOC3,TIMELESS,TIPIN,TOP2A,TOPBP1,TRIM28,UHRF1
nuclear nucleosome	10	H2BFS,HIST1H2BB,HIST1H2BC,HIST1H2BD,HIST1H2BE,HIST1H2BF,HIST1H2BG,HIST1H2BH,HIST1H2BI,HIST1H2BK,HIST1H2BL,HIST1H2BM,HIST1H2BN,HIST1H2BO,HIST1H3A,HIST1H3B,HIST1H3C,HIST1H3F,HIST1H3G,HIST1H3H,HIST1H3I,HIST1H3J,HIST2H2BD,HIST2H2BE,HIST2H2BF,HIST2H3D
protein-DNA complex	10	ACD,CDC45,CENPA,GINS1,GINS2,GINS4,H2AFX,H2AFZ,H2BFS,HIST1H1A,HIST1H1B,HIST1H1C,HIST1H1D,HIST1H1E,HIST1H2AB,HIST1H2AC,HIST1H2AD,HIST1H2AE,HIST1H2AG,HIST1H2AH,HIST1H2AI,HIST1H2AJ,HIST1H2AK,HIST1H2AL,HIST1H2AM,HIST1H2BB,HIST1H2BC,HIST1H2BD,HIST1H2BE,HIST1H2BF,HIST1H2BG,HIST1H2BH,HIST1H2BI,HIST1H2BK,HIST1H2BL,HIST1H2BM,HIST1H2BN,HIST1H2BO,HIST1H3A,HIST1H3B,HIST1H3C,HIST1H3F,HIST1H3G,HIST1H3H,HIST1H3I,HIST1H3J,HIST1H4A,HIST1H4B,HIST1H4C,HIST1H4D,HIST1H4E,HIST1H4F,HIST1H4I,HIST1H4J,HIST1H4K,HIST1H4L,HIST2H2AA3,HIST2H2AA4,HIST2H2AB,HIST2H2AC,HIST2H2BD,HIST2H2BE,HIST2H2BF,HIST2H3A,HIST2H3C,HIST2H3D,MCM3,PCNA,POLA1,POLA2,POLD3,POLE,POLE2,POLE3,PRIM1,SLF1
nuclear chromosome part	10	ACD,ACTL6A,ALYREF,ANP32E,ASF1A,ASF1B,AURKA,AURKB,BLM,BRCA2,BUB1,BUB1B,CCNB1,CDC45,CDCA5,CDK1,CENPA,CENPE,CHAF1A,CHAF1B,DCLRE1B,DCLRE1C,DDX11,DNA2,EME1,ESCO2,EZH2,FEN1,GINS1,GINS2,GINS4,H2AFX,H2AFZ,H2BFS,HAT1,HIST1H1A,HIST1H1B,HIST1H1C,HIST1H1D,HIST1H1E,HIST1H2AB,HIST1H2AC,HIST1H2AD,HIST1H2AE,HIST1H2AG,HIST1H2AH,HIST1H2AI,HIST1H2AJ,HIST1H2AK,HIST1H2AL,HIST1H2AM,HIST1H2BB,HIST1H2BC,HIST1H2BD,HIST1H2BE,HIST1H2BF,HIST1H2BG,HIST1H2BH,HIST1H2BI,HIST1H2BK,HIST1H2BL,HIST1H2BM,HIST1H2BN,HIST1H2BO,HIST1H3A,HIST1H3B,HIST1H3C,HIST1H3F,HIST1H3G,HIST1H3H,HIST1H3I,HIST1H3J,HIST1H4A,HIST1H4B,HIST1H4C,HIST1H4D,HIST1H4E,HIST1H4F,HIST1H4I,HIST1H4J,HIST1H4K,HIST1H4L,HIST2H2AA3,HIST2H2AA4,HIST2H2AB,HIST2H2AC,HIST2H2BD,HIST2H2BE,HIST2H2BF,HIST2H3D,HMGB2,INCENP,MCM10,MCM2,MCM3,MCM4,MCM5,MCM6,MCM7,MLH1,MMS22L,MSH2,MSH6,NASP

		,NCAPD2,NCAPD3,NDC80,ORC1,ORC5,ORC6,PARP1,PCNA,PLK1,POLA1,POLA2,POLD3,POLE,POLE2,POLE3,RAD51,RBMX,RCC1,RUVBL1,SGO1,SMC3,SMCHD1,SUV39H1,SUV39H2,TCP1,THOC3,TIMELESS,TIPIN,TRIM28,UHRF1
microtubule organizing center	10	AUNIP,AURKA,AURKB,BIRC5,BRCA1,BRCA2,BUB1B,CCDC15,CCDC77,CCNB1,CCNB2,CCNF,CCP110,CDC20,CDC45,CDK1,CDK2,CENPF,CENPJ,CENPU,CEP128,CEP135,CEP152,CEP192,CEP295,CEP55,CEP76,CEP78,CEP97,CFAP20,CHEK1,CKAP2,CKAP2L,CKAP5,CNTRL,DCLRE1B,DCTN5,DLGAP5,DTL,ERCC6L,ESPL1,GEN1,GSG2,HAUS4,HAUS8,HSPB11,HYLS1,ID1,KIAA0586,KIF15,KIF18A,KIF20B,KIF23,KIF24,KIFC1,MASTL,MCM3,MPHOSPH9,NCAPG,NDC1,NDE1,NEK2,NEK3,NUP107,ODF2,PARP3,PCNA,PLK1,PLK4,POC1A,POC5,RAD18,RAD51,RANBP1,RTTN,RUVBL1,SASS6,SCLT1,SGO1,SLF1,SPAG5,SPDL1,STIL,TACC3,TCP1,TOP2A,TOPBP1,TRIM59,TUBD1,TUBGCP3,TUBGCP4,WDR34,WDR62,XRCC2
microtubule organizing center part	10	AURKA,BIRC5,BRCA1,CCNF,CCP110,CENPJ,CEP128,CEP135,CEP152,CEP192,CEP295,CEP55,CEP76,CFAP20,CKAP5,KIF24,MPHOSPH9,ODF2,PARP3,PLK4,POC1A,POC5,SASS6,SCLT1,SPAG5,STIL,TCP1,TOP2A,TUBD1,TUBGCP3,TUBGCP4,WDR34,WDR62
nuclear chromatin	10	ACTL6A,ANP32E,ASF1A,ASF1B,CDCA5,CHAF1A,CHAF1B,DDX11,EME1,ESCO2,EZH2,H2AFX,H2AFZ,H2BFS,HAT1,HIST1H1A,HIST1H1B,HIST1H1C,HIST1H1D,HIST1H1E,HIST1H2AB,HIST1H2AC,HIST1H2AD,HIST1H2AE,HIST1H2AG,HIST1H2AH,HIST1H2AI,HIST1H2AJ,HIST1H2AK,HIST1H2AL,HIST1H2AM,HIST1H2BB,HIST1H2BC,HIST1H2BD,HIST1H2BE,HIST1H2BF,HIST1H2BG,HIST1H2BH,HIST1H2BI,HIST1H2BK,HIST1H2BL,HIST1H2BM,HIST1H2BN,HIST1H2BO,HIST1H3A,HIST1H3B,HIST1H3C,HIST1H3F,HIST1H3G,HIST1H3H,HIST1H3I,HIST1H3J,HIST2H2AA3,HIST2H2AA4,HIST2H2AB,HIST2H2AC,HIST2H2BD,HIST2H2BE,HIST2H2BF,HIST2H3D,HMGB2,MSH6,NASP,NCAPD3,RBMX,RCC1,RUVBL1,SUV39H1,SUV39H2,TCP1,TIMELESS,TIPIN,TRIM28,UHRF1
nuclear pore	10	GLE1,KPNA2,LBR,MAD2L1,NDC1,NUP107,NUP153,NUP155,NUP160,NUP188,NUP205,NUP35,NUP43,NUP58,NUP85,NUP88
midbody	10	ASPM,AURKA,AURKB,BIRC5,CDCA8,CDK1,CENPE,CENPF,CEP55,DDX11,ECT2,INCENP,KIF14,KIF20A,KIF20B,KIF23,KIF4A,NEK2,PLK1,PRC1,PSRC1,RACGAP1,SPAG5
spindle microtubule	10	AURKA,AURKB,BIRC5,CDK1,KIF11,KIF18A,KIF18B,KIF4A,KNTC1,NUSAP1,PLK1,PRC1,PSRC1,SKA1,SKA2,SKA3,SPAG5,TPX2,TUBGCP3
microtubule	10	ASPM,AURKA,AURKB,BIRC5,CDK1,CENPE,CENPJ,CFAP20,CKAP2,CKAP5,GAS2L3,GTSE1,HAUS4,HAUS8,HSPH1,INCENP,KIF11,KIF14,KIF15,KIF18A,KIF18B,KIF20A,KIF20B,KIF22,KIF23,KIF24,KIF2C,KIF4A,KIF4B,KIFC1,KNSTRN,KNTC1,NDE1,NEK2,NUSAP1,ODF2,PLK1,PRC1,PSRC1,RACGAP1,REEP4,SKA1,SKA2,SKA3,SPAG5,STMN1,TCP1,TPX2,TUBD1,TUBGCP3,TUBGCP4
nucleolus	10	BLM,CDCA8,CDKN2A,CENPP,CENPW,CHAF1A,CTCF,DDX11,DTL,EME1,EMG1,ETV4,EXOSC2,EXOSC9,FAM64A,FANCD2,FANCG,FBL,FEN1,G2E3,HIST1H1E,HJURP,HMGB2,KIF20B,MCM10,MKI67,MRTO4,NCL,NEK2,NOLC1,NOP56,NOP58,NUP153,NUSAP1,ORC1,PARP1,PARP2,PLK1,PLK4,POLA1,RAD51,RBBP8,RBM14,RPL23A,RPL7A,RPP30,RPS6KA6,SMC2,SPC24,SSRP1,TAF5,TIMELESS,TOE1,TOP2A,TRAIP,TRIM28,TYMS,VRK1
nuclear chromosome, telomeric region	10	ACD,ALYREF,BRCA2,CDK1,DCLRE1B,DCLRE1C,DNA2,FEN1,HAT1,HIST1H2BB,HIST1H3A,HIST1H3B,HIST1H3C,HIST1H3F,HIST1H3G,HIST1H3H,HIST1H3I,HIST1H3J,HIST1H4A,HIST1H4B,HIST1H4C,HIST1H4D,HIST1H4E,HIST1H4F,HIST1H4I,HIST1H4J,HIST1H4K,HIST1H4L,HIST2H3D,MCM2,MCM3,MCM4,MCM5,MCM6,MCM7,MSH2,ORC1,ORC5,PARP1,PCNA,RAD51,SMCHD1,THOC3
centriole	10	AURKA,BIRC5,CCNF,CCP110,CENPJ,CEP128,CEP135,CEP152,CEP192,CEP295,CEP55,CEP76,CFAP20,KIF24,MPHOSPH9,ODF2,PARP3,PLK4,POC1A,POC5,SASS6,SCLT1,STIL,TOP2A,TUBD1,TUBGCP3,WDR34,WDR62
microtubule associated complex	10	AURKA,AURKB,BIRC5,CDCA8,CENPE,DCTN5,HAUS4,HAUS8,KIF11,KIF14,KIF15,KIF18A,KIF18B,KIF20A,KIF20B,KIF22,KIF23,KIF24,KIF2C,KIF4A,KIF4B,KIFC1,NDE1,WDR34
chromosome	10	ACD,ACTL6A,ALYREF,ANP32E,ASF1A,ASF1B,AURKA,AURKB,BIRC5,BLM,BRCA1,BRCA2,BUB1,BUB1B,BUB3,CCNB1,CDC45,CDCA2,CDCA5,CDCA8,CDK1,CDK2,CDKN2A,CENPA,CENPE,CENPF,CENPH,CENPI,CENPK,CENPL,CENPM,CENPN,CENPO,CENPP,CENPQ,CENPU,CENPW,CHAF1A,CHAF1B,CHEK1,CLSPN,CTCF,DCLRE1B,DCLRE1C,DCTN5,DDX11,DMC1,DNA2,DNMT1,DSCC1,DSN1,DTL,EME1,ERCC6L,ESCO2,EXOSC9,EZH2,FAM111A,FANCC,FANCD2,FBL,FEN1,FIGNL1,GINS1,GINS2,GINS4,GSG2,H2AFX,H2AFZ,H2BFS,HAT1,HELLS,HIST1H1A,HIST1H1B,HIST1H1C,HIST1H1D,HIST1H1E,HIST1H2AB,HIST1H2AC,HIST1H2AD,HIST1H2AE,HIST1H2AG,HIST1H2AH,HIST1H2AI,HIST1H2AJ,HIST1H2AK,HIST1H2AL,HIST1H2AM,HIST1H2BB,HIST1H2BC,HIST1H2BD,HIST1H2BE,HIST1H2BF,HIST1H2BG,HIST1H2BH,HIST1H2BI,HIST1H2BK,HIST1H2BL,HIST1H2BM,HIST1H2BN,HIST1H2BO,HIST1H3A,HIST1H3B,HIST1H3C,HIST1H3F,HIST1H3G,HIST1H3H,HIST1H3I,HIST1H3J,HIST1H4A,HIST1H4B,HIST1H4C,HIST1H4D,HIST1H4E,HIST1H4F,HIST1H4I,HIST1H4J,HIST1H4K,HIST1H4L,HIST2H2AA3,HIST2H2AA4,HIST2H2AB,HIST2H2AC,HIST2H2BD,HIST2H2BE,HIST2H2BF,HIST2H3A,HIST2H3C,HIST2H3D,HJURP,HMBS,HMGB1,HMGB2,HMGN2,HMGN3,INCENP,ITGB3BP,KIF18A,KIF22,KIF2C,KIF4A,KNSTRN,KNTC1,MAD2L1,MCM10,MCM2,MCM3,MCM4,MCM5,MCM6,MCM7,MIS18A,MKI67,MLH1,MMS22L,MSH2,MSH6,MTBP,MYBL2,NASP,NCAPD2,NCAPD3,NCAPG,NCAPH,NDC80,NDE1,NEK2,NUF2,NUP107,NUP160,NUP43,NUP85,NUSAP1,ORC1,ORC5,ORC6,PARP1,PARP3,PARPBP,PCNA,PLK1,PLK4,POLA1,POLA2,POLD3,POLE,POLE2,POLE3,POLQ,PRIM1,RAD18,RAD51,RBBP8,RBMX,RCC1,RFC2,RFC3,RFC4,RFWD3,RUVBL1,SGO1,SKA1,SKA2,SKA3,SLF1,SMC1A,SMC2,SMC3,SMC4,SMCHD1,SPAG5,SPC24,SPC25,SPDL1,SSRP1,SUV39H1,SUV39H2,TCP1,THOC3,TIMELESS,TIPIN,TMPO,TOP2A,TOPBP1,TRIM28,UHRF1,WDHD1,XRCC2,ZWILCH,ZWINT
spindle pole	10	ASPM,AUNIP,AURKA,AURKB,CCNB1,CDC20,CDC6,CENPF,CEP128,CKAP2,CKAP2L,CKAP5,CNTRL,DDX11,DLGAP5,GPSM2,HAUS8,KIF11,KIF20B,KIFC1,KNSTRN,KNTC1,MAD2L1,NDC1,NDE1,NEK2,ODF2,PLK1,POC1A,PRC1,PSRC1,SGO1,SMC3,SPAG5,SPDL1,TOPBP1,TPX2,TUBGCP3,TUBGCP4,WDR62
nucleosome	10	CENPA,H2AFX,H2AFZ,H2BFS,HIST1H1A,HIST1H1B,HIST1H1C,HIST1H1D,HIST1H1E,HIST1H2AB,HIST1H2AC,HIST1H2AD,HIST1H2AE,HIST1H2AG,HIST1H2AH,HIST1H2AI,HIST1H2AJ,HIST1H2AK,HIST1H2AL,HIST1H2AM,HIST1H2BB,HIST1H2BC,HIST1H2BD,HIST1H2BE,HIST1H2BF,HIST1H2BG,HIST1H2BH,HIST1H2BI,HIST1H2BK,HIST1H2BL,HIST1H2BM,HIST1H2BN,HIST1H2BO,HIST1H3A,HIST1H3B,HIST1H3C,HIST1H3F,HIST1H3G,HIST1H3H,HIST1H3I,HIST1H3J,HIST1H4A,HIST1H4B,HIST1H4C,HIST1H4D,HIST1H4E,HIST1H4F,HIST1H4I,HIST1H4J,HIST1H4K,HIST1H4L,HIST2H2AA3,HIST2H2AA4,HIST2H2AB,HIST2H2AC,HIST2H2BD,HIST2H2BE,HIST2H2BF,HIST2H3A,HIST2H3C,HIST2H3D,SLF1
replisome	10	MCM3,PCNA,POLA1,POLA2,POLD3,POLE,POLE2,POLE3,PRIM1
chromosomal region	10	ACD,ALYREF,AURKA,AURKB,BIRC5,BLM,BRCA2,BUB1,BUB1B,BUB3,CCNB1,CDCA5,CDCA8,CDK1,CDK2,CENPA,CENPE,CENPF,CENPH,CENPI,CENPK,CENPL,CENPM,CENPN,CENPO,CENPP,CENPQ,CENPU,CENPW,CHEK1,CTCF,DCLRE1B,DCLRE1C,DCTN5,DMC1,DNA2,DNMT1,DSCC1,DSN1,ERCC6L,ESCO2,FEN1,H2AFX,HAT1,HELLS,HIST1H2BB,HIST1H3A,HIST1H3B,HIST1H3C,HIST1H3F,HIST1H3G,HIST1H3H,HIST1H3I,HIST1H3J,HIST1H4A,HIST1H4B,HIST1H4C,HIST1H4D,HIST1H4E,HIST1H4F,HIST1H4I,HIST1H4J,HIST1H4K,HIST1H4L,HIST2H3D,HJURP,INCENP,ITGB3BP,KIF18A,KIF22,KIF2C,KNSTRN,KNTC1,MAD2L1,MCM2,MCM3,MCM4,MCM5,MCM6,MCM7,MIS18A,MKI67,MSH2,MTBP,NCAPD2,NCAPD3,NDC80,NDE1,NEK2,NUF2,NUP107,NUP160,NUP43,NUP85,ORC1,ORC5,PARP1,PCNA,PLK1,RAD51,SGO1,SKA1,SKA2,SKA3,SMC1A,SMC3,SMCHD1,SPAG5,SPC24,SPC25,SPDL1,SUV39H1,SUV39H2,THOC3,WDHD1,ZWILCH,ZWINT
kinetochore	10	AURKB,BIRC5,BUB1,BUB1B,BUB3,CCNB1,CENPA,CENPE,CENPF,CENPH,CENPI,CENPK,CENPM,CENPN,CENPO,CENPU,CENPW,DCTN5,DSN1,ERCC6L,HJURP,INCENP,ITGB3BP,KIF18A,KIF22,KIF2C,KNSTRN,KNTC1,MAD2L1,MTBP,NDC80,NDE1,NEK2,NUF2,NUP107,NUP160,NUP43,NUP85,PLK1,SGO1,SKA1,SKA2,SKA3,SMC1A,SPAG5,SPC24,SPC25,SPDL1,ZWILCH,ZWINT
nuclear periphery	10	BLM,CENPF,CENPW,HAT1,KIF4A,KIF4B,LBR,LMNB1,LMNB2,NRM,NUP107,NUP153,NUP205,NUP35,PAXIP1,PCNA,PHB2,POLA1,RUVBL1,SMC3,SUV39H1,UHRF1
DNA packaging complex	10	CENPA,H2AFX,H2AFZ,H2BFS,HIST1H1A,HIST1H1B,HIST1H1C,HIST1H1D,HIST1H1E,HIST1H2AB,HIST1H2AC,HIST1H2AD,HIST1H2AE,HIST1H2AG,HIST1H2AH,HIST1H2AI,HIST1H2AJ,HIST1H2AK,HIST1H2AL,HIST1H2AM,HIST1H2BB,HIST1H2BC,HIST1H2BD,HIST1H2BE,HIST1H2BF,HIST1H2BG,HIST1H2BH,HIST1H2BI,HIST1H2BK,HIST1H2BL,HIST1H2BM,HIST1H2BN,HIST1H2BO,HIST1H3A,HIST1H3B,HIST1H3C,HIST1H3F,HIST1H3G,HIST1H3H,HIST1H3I,HIST1H3J,HIST1H4A,HIST1H4B,HIST1H4C,HIST1H4D,HIST1H4E,HIST1H4F,HIST1H4I,HIST1H4J,HIST1H4K,HIST1H4L,HIST2H2AA3,HIST2H2AA4,HIST2H2AB,HIST2H2AC,HIST2H2BD,HIST2H2BE,HIST2H2BF,HIST2H3A,HIST2H3C,HIST2H3D,NCAPD2,NCAPD3,NCAPG,NCAPH,SLF1,SMC2,SMC4
condensed chromosome	10	AURKA,AURKB,BIRC5,BLM,BRCA1,BUB1,BUB1B,BUB3,CCNB1,CDCA5,CDK2,CENPA,CENPE,CENPF,CENPH,CENPK,CENPM,CENPN,CENPO,CENPU,CENPW,CHEK1,CTCF,DCTN5,DMC1,DSN1,ERCC6L,FANCD2,H2AFX,HJURP,HMBS,HMGB1,HMGB2,INCENP,ITGB3BP,KIF2C,KNSTRN,KNTC1,MAD2L1,MKI67,MLH1,NCAPD2,NCAPD3,NCAPG,NCAPH,NDC80,NDE1,NEK2,NUF2,NUP107,NUP43,NUP85,PLK1,RAD51,RCC1,SGO1,SKA1,SKA2,SKA3,SMC1A,SMC2,SMC3,SMC4,SPAG5,SPC24,SPC25,SPDL1,SUV39H1,TOP2A,TOPBP1,ZWILCH,ZWINT
condensed chromosome kinetochore	10	BIRC5,BUB1,BUB1B,BUB3,CCNB1,CENPA,CENPE,CENPF,CENPH,CENPK,CENPM,CENPN,CENPO,CENPU,CENPW,DCTN5,DSN1,ERCC6L,HJURP,INCENP,ITGB3BP,KIF2C,KNSTRN,KNTC1,MAD2L1,NDC80,NDE1,NEK2,NUF2,NUP107,NUP43,NUP85,PLK1,SGO1,SKA1,SKA2,SKA3,SMC1A,SPAG5,SPC24,SPC25,SPDL1,ZWILCH,ZWINT
condensed nuclear chromosome kinetochore	10	BUB1,BUB1B,CCNB1,CENPA,CENPE,NDC80,PLK1
condensed nuclear chromosome	10	AURKA,AURKB,BLM,BRCA1,BUB1,BUB1B,CCNB1,CENPA,CENPE,CHEK1,DMC1,H2AFX,INCENP,MLH1,NCAPD2,NCAPD3,NDC80,NEK2,PLK1,RAD51,RCC1,SGO1,SMC1A,SMC3,SUV39H1,TOPBP1

hsa-miR-760:PITA_TOP	10	HIST1H1B,HIST1H1D,HIST1H1E,HIST1H2AB,HIST1H2AC,HIST1H2AD,HIST1H2AE,HIST1H2AH,HIST1H2AI,HIST1H2AJ,HIST1H2AK,HIST1H2AL,HIST1H2AM,HIST1H2BE,HIST1H2BG,HIST1H2BM,HIST1H2BN,HIST1H3A,HIST1H3B,HIST1H3F,HIST1H3H,HIST1H3I,HIST1H3J,HIST2H2BE,HIST2H2BF,LMNB1,NCL
hsa-miR-1276:PITA_TOP	10	ANP32E,HIST1H1A,HIST1H1B,HIST1H1D,HIST1H1E,HIST1H2AB,HIST1H2AC,HIST1H2AD,HIST1H2AE,HIST1H2AG,HIST1H2AH,HIST1H2AI,HIST1H2AJ,HIST1H2AL,HIST1H2AM,HIST1H2BC,HIST1H2BE,HIST1H2BG,HIST1H2BM,HIST1H2BN,HIST1H3A,HIST1H3B,HIST1H3F,HIST1H3G,HIST1H3H,HIST1H3I,HIST1H3J,HIST2H2AB,HIST2H2BE,HIST2H2BF,LMNB1,NEMP1
hsa-miR-24:miRTarbase	10	ARHGAP19,AURKB,BRCA1,CCNA2,CDK1,CDKN2A,CHEK1,DHFR,DHFRP1,FEN1,H2AFX,PCNA
structural constituent of nuclear pore	10	NDC1,NUP107,NUP153,NUP155,NUP188,NUP205,NUP85
pyrophosphatase activity	10	ATAD2,BLM,BRIP1,CDC45,CENPE,DDX11,DDX12P,DDX39A,DMC1,DNA2,DSCC1,DUT,ERCC6L,FANCM,FIGNL1,GBP4,GINS1,GINS2,GINS4,HELLS,KIF11,KIF14,KIF15,KIF18A,KIF18B,KIF20A,KIF20B,KIF22,KIF23,KIF24,KIF2C,KIF4A,KIF4B,KIFC1,MCM2,MCM3,MCM4,MCM5,MCM6,MCM7,MCM8,MLH1,MSH2,MSH6,MYO19,POLQ,PRIM1,PRUNE2,RAD18,RAD51,RAD54B,RAD54L,RFC2,RFC3,RFC4,RUVBL1,SMC3,TAPBP,TOP2A,TTF2,TUBD1,XRCC2
hydrolase activity, acting on acid anhydrides	10	ATAD2,BLM,BRIP1,CDC45,CENPE,DDX11,DDX12P,DDX39A,DMC1,DNA2,DSCC1,DUT,ERCC6L,FANCM,FIGNL1,GBP4,GINS1,GINS2,GINS4,HELLS,KIF11,KIF14,KIF15,KIF18A,KIF18B,KIF20A,KIF20B,KIF22,KIF23,KIF24,KIF2C,KIF4A,KIF4B,KIFC1,MCM2,MCM3,MCM4,MCM5,MCM6,MCM7,MCM8,MLH1,MSH2,MSH6,MYO19,POLQ,PRIM1,PRUNE2,RAD18,RAD51,RAD54B,RAD54L,RFC2,RFC3,RFC4,RUVBL1,SMC3,TAPBP,TOP2A,TTF2,TUBD1,XRCC2
helicase activity	10	BLM,BRIP1,CDC45,DDX11,DDX12P,DDX39A,DNA2,ERCC6L,FANCM,GINS1,GINS2,GINS4,HELLS,MCM2,MCM3,MCM4,MCM5,MCM6,MCM7,MCM8,PRIM1,RAD54B,RAD54L,RUVBL1,TTF2
DNA helicase activity	10	BLM,BRIP1,CDC45,DDX11,DDX12P,DNA2,GINS1,GINS2,GINS4,MCM2,MCM3,MCM4,MCM5,MCM6,MCM7,RAD54B,RUVBL1
four-way junction DNA binding	10	DMC1,HMGB1,HMGB2,MSH2,MSH6,RAD51,XRCC2
ATP binding	10	ALPK2,APAF1,ASS1,ATAD2,ATAD5,AURKA,AURKB,BLM,BRIP1,BUB1,BUB1B,CDC6,CDC7,CDK1,CDK2,CENPE,CHEK1,CHORDC1,CIT,DARS2,DCK,DDR2,DDX11,DDX12P,DDX39A,DMC1,DNA2,DTYMK,ERCC6L,FANCM,FIGNL1,GSG2,HELLS,HSPA14,HSPE1,HSPH1,KIF11,KIF14,KIF15,KIF18A,KIF18B,KIF20A,KIF20B,KIF22,KIF23,KIF24,KIF2C,KIF4A,KIF4B,KIFC1,LIG1,MASTL,MB21D1,MCM2,MCM3,MCM4,MCM5,MCM6,MCM7,MCM8,MELK,MKI67,MLH1,MSH2,MSH6,MTHFD1,MYLK,MYO19,NEK2,NEK3,NOLC1,ORC1,ORC5,PAICS,PBK,PFAS,PKMYT1,PLK1,PLK4,POLQ,RAD51,RAD54B,RAD54L,RFC2,RFC4,RPS6KA6,RRM1,RUVBL1,SEPHS1,SMC1A,SMC2,SMC3,SMC4,SMCHD1,SPATA5,TCP1,TK1,TOP2A,TPX2,TRIP13,TRPM4,TTF2,TTK,UBE2C,UBE2S,UBE2T,VRK1,XRCC2
single-stranded DNA-dependent ATPase activity	10	DNA2,DSCC1,POLQ,RAD18,RAD51,RFC2,RFC3,RFC4
histone binding	10	ANP32E,ASF1A,ASF1B,ATAD2,CHAF1B,H2AFX,HAT1,HIST1H3A,HIST1H3B,HIST1H3C,HIST1H3F,HIST1H3G,HIST1H3H,HIST1H3I,HIST1H3J,HIST1H4A,HIST1H4B,HIST1H4C,HIST1H4D,HIST1H4E,HIST1H4F,HIST1H4I,HIST1H4J,HIST1H4K,HIST1H4L,HIST2H3A,HIST2H3C,HIST2H3D,HJURP,MCM2,MSH6,NASP,NCAPD2,NCAPD3,NCAPG2,NCL,PHF19,RCC1,UHRF1,VRK1
chromatin binding	10	ACTL6A,ASF1A,ATAD2,BRCA1,CDC45,CDC6,CDCA5,CDK1,CENPA,CENPF,CHAF1A,CHAF1B,CKS2,CTCF,DNMT1,EXO1,EZH2,FANCM,H2AFZ,HELLS,HIST1H1A,HIST1H1B,HIST1H1C,HIST1H1D,HIST1H1E,HIST1H3C,HMGN2,HMGN3,MCM5,MCMBP,MLH1,MSH6,NCAPD2,NCAPD3,NCOA5,NUP153,ORC1,PARP1,PCNA,POLA1,POLE,POLQ,RAD51,RBL1,RBMX,RCC1,SMC1A,SMC3,SSRP1,SUV39H1,SUV39H2,TFAM,TICRR,TOP2A,TRIM28,UBE2T,UHRF1,VRK1,WDHD1
histone kinase activity	10	AURKA,AURKB,CCNB1,CDK1,CDK2,CHEK1,GSG2,VRK1
damaged DNA binding	10	BRCA1,DCLRE1B,DCLRE1C,FANCG,FEN1,H2AFX,HMGB1,HMGB2,MSH2,MSH6,NEIL3,PCNA,POLQ,RAD18,RBBP8,UNG
DNA-dependent ATPase activity	10	BLM,BRIP1,DDX11,DDX12P,DMC1,DNA2,DSCC1,MCM4,MCM6,MCM7,POLQ,RAD18,RAD51,RAD54B,RFC2,RFC3,RFC4,RUVBL1,TOP2A,TTF2,XRCC2
tubulin binding	10	BIRC5,BRCA1,BRCA2,CENPE,CENPJ,CKAP5,CLIP3,GAS2L3,HSPH1,KIF11,KIF14,KIF15,KIF18A,KIF18B,KIF20A,KIF20B,KIF22,KIF23,KIF24,KIF2C,KIF4A,KIF4B,KIFC1,NDE1,NUSAP1,PLK1,PRC1,PSRC1,RACGAP1,REEP4,SKA1,SKA2,SPAG5,STMN1,TUBGCP3,TUBGCP4
ATPase activity	10	ATAD2,BLM,BRIP1,CENPE,DDX11,DDX12P,DDX39A,DMC1,DNA2,DSCC1,FIGNL1,KIF11,KIF14,KIF15,KIF18A,KIF18B,KIF20A,KIF20B,KIF22,KIF23,KIF24,KIF2C,KIF4A,KIF4B,KIFC1,MCM4,MCM6,MCM7,MLH1,MSH2,MSH6,MYO19,POLQ,RAD18,RAD51,RAD54B,RFC2,RFC3,RFC4,RUVBL1,TAPBP,TOP2A,TTF2,XRCC2
hydrolase activity, acting on acid anhydrides, in phosphorus-containing anhydrides	10	ATAD2,BLM,BRIP1,CDC45,CENPE,DDX11,DDX12P,DDX39A,DMC1,DNA2,DSCC1,DUT,ERCC6L,FANCM,FIGNL1,GBP4,GINS1,GINS2,GINS4,HELLS,KIF11,KIF14,KIF15,KIF18A,KIF18B,KIF20A,KIF20B,KIF22,KIF23,KIF24,KIF2C,KIF4A,KIF4B,KIFC1,MCM2,MCM3,MCM4,MCM5,MCM6,MCM7,MCM8,MLH1,MSH2,MSH6,MYO19,POLQ,PRIM1,PRUNE2,RAD18,RAD51,RAD54B,RAD54L,RFC2,RFC3,RFC4,RUVBL1,SMC3,TAPBP,TOP2A,TTF2,TUBD1,XRCC2
protein heterodimerization activity	10	BARD1,BIRC5,CENPA,CENPW,GABPB1,H2AFX,H2AFZ,H2BFS,HIST1H2AB,HIST1H2AC,HIST1H2AD,HIST1H2AE,HIST1H2AG,HIST1H2AH,HIST1H2AI,HIST1H2AJ,HIST1H2AK,HIST1H2AL,HIST1H2AM,HIST1H2BB,HIST1H2BC,HIST1H2BD,HIST1H2BE,HIST1H2BF,HIST1H2BG,HIST1H2BH,HIST1H2BI,HIST1H2BK,HIST1H2BL,HIST1H2BM,HIST1H2BN,HIST1H2BO,HIST1H3A,HIST1H3B,HIST1H3C,HIST1H3F,HIST1H3G,HIST1H3H,HIST1H3I,HIST1H3J,HIST1H4A,HIST1H4B,HIST1H4C,HIST1H4D,HIST1H4E,HIST1H4F,HIST1H4I,HIST1H4J,HIST1H4K,HIST1H4L,HIST2H2AA3,HIST2H2AA4,HIST2H2AB,HIST2H2AC,HIST2H2BD,HIST2H2BE,HIST2H2BF,HIST2H3A,HIST2H3C,HIST2H3D,ITGA7,LSM5,POLA1,POLA2,POLE3,SEPHS1,SMC1A,SMC2,SMC3,SMC4,TIMELESS,TOP2A
ATP-dependent microtubule motor activity	10	KIF11,KIF14,KIF18A,KIF18B,KIF20B,KIF4A,KIF4B,KIFC1
DNA secondary structure binding	10	BLM,CLSPN,DMC1,HMGB1,HMGB2,MSH2,MSH6,NEIL3,RAD18,RAD51,XRCC2
nucleoside-triphosphatase activity	10	ATAD2,BLM,BRIP1,CDC45,CENPE,DDX11,DDX12P,DDX39A,DMC1,DNA2,DSCC1,ERCC6L,FANCM,FIGNL1,GBP4,GINS1,GINS2,GINS4,HELLS,KIF11,KIF14,KIF15,KIF18A,KIF18B,KIF20A,KIF20B,KIF22,KIF23,KIF24,KIF2C,KIF4A,KIF4B,KIFC1,MCM2,MCM3,MCM4,MCM5,MCM6,MCM7,MCM8,MLH1,MSH2,MSH6,MYO19,POLQ,PRIM1,RAD18,RAD51,RAD54B,RAD54L,RFC2,RFC3,RFC4,RUVBL1,SMC3,TAPBP,TOP2A,TTF2,TUBD1,XRCC2
ATP-dependent microtubule motor activity, plus-end-directed	10	KIF11,KIF14,KIF18A,KIF18B,KIF20B,KIF4A,KIF4B
microtubule motor activity	10	CENPE,KIF11,KIF14,KIF15,KIF18A,KIF18B,KIF20A,KIF20B,KIF22,KIF23,KIF24,KIF2C,KIF4A,KIF4B,KIFC1,SMC3
microtubule binding	10	BIRC5,CENPE,CKAP5,CLIP3,GAS2L3,KIF11,KIF14,KIF15,KIF18A,KIF18B,KIF20A,KIF20B,KIF22,KIF23,KIF24,KIF2C,KIF4A,KIF4B,KIFC1,NDE1,NUSAP1,PLK1,PRC1,PSRC1,RACGAP1,REEP4,SKA1,SKA2,SPAG5,TUBGCP3,TUBGCP4
protein dimerization activity	10	ATOH8,BARD1,BIRC5,CACYBP,CDH13,CENPA,CENPF,CENPW,DARS2,DCK,E2F8,ECT2,GABPB1,H2AFX,H2AFZ,H2BFS,HIST1H2AB,HIST1H2AC,HIST1H2AD,HIST1H2AE,HIST1H2AG,HIST1H2AH,HIST1H2AI,HIST1H2AJ,HIST1H2AK,HIST1H2AL,HIST1H2AM,HIST1H2BB,HIST1H2BC,HIST1H2BD,HIST1H2BE,HIST1H2BF,HIST1H2BG,HIST1H2BH,HIST1H2BI,HIST1H2BK,HIST1H2BL,HIST1H2BM,HIST1H2BN,HIST1H2BO,HIST1H3A,HIST1H3B,HIST1H3C,HIST1H3F,HIST1H3G,HIST1H3H,HIST1H3I,HIST1H3J,HIST1H4A,HIST1H4B,HIST1H4C,HIST1H4D,HIST1H4E,HIST1H4F,HIST1H4I,HIST1H4J,HIST1H4K,HIST1H4L,HIST2H2AA3,HIST2H2AA4,HIST2H2AB,HIST2H2AC,HIST2H2BD,HIST2H2BE,HIST2H2BF,HIST2H3A,HIST2H3C,HIST2H3D,HPRT1,ID1,ITGA7,KIF20B,LRRFIP1,LSM5,MAD2L1,MSH2,MSH6,NUP35,OLFML2B,POLA1,POLA2,POLE3,PRTFDC1,PSMC3IP,SEPHS1,SMC1A,SMC2,SMC3,SMC4,TAF5,TIMELESS,TOP2A,TYMS,ZMYM1
single-stranded DNA binding	10	ALYREF,BLM,BRCA2,CDC45,DDX11,DMC1,HMGB1,HMGB2,MCM10,MCM4,MCM6,MCM7,MLH1,MSH2,NCL,NEIL3,NUP35,RAD51,RAD51AP1,TDP1,XRCC2
Ndc80 complex	5.9505143	NDC80,NUF2,SPC24,SPC25

DNA replication preinitiation complex	5.9505143	CDC45,GINS1,GINS2,GINS4
condensed nuclear chromosome outer kinetochore	5.9505143	BUB1,CCNB1,NDC80,PLK1
double-strand break repair via break-induced replication	5.94046294	CDC45,CDC7,GINS2,GINS4
negative regulation of protein catabolic process	5.91255347	ANAPC15,BUB1,BUB1B,BUB3,CCNB1,CDKN2A,CENPE,CENPF,CEP192,GEN1,MAD2L1,MTBP,PBK,PLK1,SENP1,STIL,TTK
positive regulation of nuclear division	5.90508693	AURKA,BIRC5,CDCA5,CENPE,DLGAP5,ESPL1,FBXO5,NUSAP1,PHB2,RANBP1,SLF1,UBE2C
response to radiation	5.89878349	AURKB,BLM,BRCA1,BRCA2,CDC25A,CHEK1,DCLRE1C,DDIAS,DMC1,DNMT1,DTL,ECT2,FANCD2,FANCG,FEN1,FIGNL1,H2AFX,MSH2,MSH6,PAXIP1,PBK,PCNA,RAD18,RAD51,RAD51AP1,RAD54B,RAD54L,RFWD3,RRM1,SMC1A,SOD2,TICRR,TIPIN,TMEM109,TOPBP1,USP1,XRCC2
motor activity	5.89576589	CENPE,KIF11,KIF14,KIF15,KIF18A,KIF18B,KIF20A,KIF20B,KIF22,KIF23,KIF24,KIF2C,KIF4A,KIF4B,KIFC1,MYO19,SMC3
mismatch repair	5.89547591	EXO1,HMGB1,LIG1,MLH1,MSH2,MSH6,PCNA,POLD3,RNASEH2A
hsa-miR-302c:mirSVR_conserved_highEffect-0.5	5.85625876	ACTL6A,ANAPC15,ARHGAP11B,ARL6IP6,ASF1A,ASF1B,ATAD2,AUNIP,BTG3,BUB1,C18orf54,C19orf48,CCNE2,CDCA2,CDCA7,CDK1,CENPJ,CENPO,CEP135,CEP55,CEP76,CEP97,CKS1B,DCLRE1C,DDIAS,DNAJC9,DRAM1,ECT2,ERLIN1,FANCD2,FANCM,FNDC3A,GABPB1,GPN3,GPR137C,HAUS8,HELLS,HIST1H2BB,HIST1H2BD,HMGB1,HMGN3,ITGB3BP,KIAA0586,KNSTRN,KPNA2,MCM10,MCMBP,MLH1,MMS22L,MPHOSPH9,MYO19,NUP205,ORC5,OSGEPL1,PARPBP,PBK,PLK4,POC1A,POLQ,PPIH,PRIM1,PRR11,PRTFDC1,RACGAP1,RAD18,RAD51AP1,RBL1,RCC1,RPP30,RRM1,SENP1,SHC4,SHCBP1,SKA2,SLF1,SRRT,SUV39H1,TTI1,TUBGCP4,ZNF367
nuclear transport	5.85261743	ALYREF,BARD1,BRCA1,BUD13,CDK1,CDKN2A,CSE1L,DDX39A,ECT2,GDF5,GEMIN5,GEMIN6,GLE1,GTSE1,KPNA2,NDC1,NOP58,NUP107,NUP153,NUP155,NUP160,NUP188,NUP205,NUP35,NUP43,NUP58,NUP85,NUP88,PARP1,PHB2,POLA2,RANBP1,SNRPB,SNRPD1,SRSF7,TACC3,THOC3,TRIM28
regulation of cytokinesis	5.83225019	AURKA,AURKB,BRCA2,CCP110,CDC6,CIT,E2F8,ECT2,KIF14,KIF20B,KIF23,RACGAP1
DNA polymerase binding	5.80028978	ACD,FANCD2,FANCI,HMGB1,PCNA,RAD51
3'-5' DNA helicase activity	5.80028978	BLM,CDC45,GINS1,GINS2,GINS4,RUVBL1
DNA damage response, detection of DNA damage	5.78316295	DTL,PARP1,PCNA,POLD3,RAD18,RFC2,RFC3,RFC4,USP1
mitotic spindle midzone assembly	5.7330439	AURKB,KIF23,KIF4A,KIF4B,RACGAP1
protein modification by small protein conjugation or removal	5.72576933	ANAPC1,ANAPC15,AURKA,AURKB,BARD1,BIRC5,BLM,BRCA1,BUB1B,BUB3,CCNB1,CCNF,CDC20,CDCA3,CDCA8,CDK1,CDK2,CDKN2A,DTL,FANCI,FBXO5,G2E3,INCENP,LRR1,MAD2L1,MASTL,NDC1,NOP58,NUP107,NUP153,NUP155,NUP160,NUP188,NUP205,NUP35,NUP43,NUP58,NUP85,NUP88,OTUD7A,PARP1,PAXIP1,PCNA,PLK1,PTTG1,RAD18,RFWD3,SENP1,SMC1A,SMC3,TOP2A,TRAIP,TRIM28,TRIM59,TRPM4,UBE2C,UBE2S,UBE2T,UHRF1,USP1,USP13,ZNF738
positive regulation of ubiquitin protein ligase activity	5.70366988	ANAPC1,ANAPC15,BUB1B,BUB3,CCNB1,CDC20,CDK1,FBXO5,MAD2L1,MASTL,PLK1,UBE2C,UBE2S
positive regulation of chromosome segregation	5.70023583	CCNB1,CDC6,CENPE,DLGAP5,ESPL1,RAD18,SLF1
spliceosomal tri-snRNP complex	5.69810448	LSM2,LSM3,LSM5,PPIH,PRPF3,PRPF4,SNRPB,SNRPD1

DNA damage response, signal transduction by p53 class mediator	5.66278753	AURKA,BRCA1,BRCA2,CASP2,CCNB1,CDC25C,CDK1,CDK2,CDKN2A,CENPJ,FOXM1,GTSE1,PAXIP1,PCNA,RPS6KA6
signal transduction in response to DNA damage	5.66216392	AURKA,BRCA1,BRCA2,CASP2,CCNB1,CDC25C,CDK1,CDK2,CDKN2A,CENPJ,FOXM1,GTSE1,PAXIP1,PCNA,RPS6KA6,SMC1A
regulation of proteolysis involved in cellular protein catabolic process	5.63801032	ANAPC1,ANAPC15,AURKA,BUB1,BUB1B,BUB3,CCNB1,CDC20,CDK1,CDKN2A,CENPE,CENPF,CEP192,DLGAP5,ESPL1,FBXO5,GEN1,MAD2L1,MTBP,PBK,PLK1,SENP1,STIL,TTK,UBE2C
regulation of centriole replication	5.55651324	BRCA1,CENPJ,CEP192,CEP76,PLK4,RBM14
replication fork processing	5.53647311	ALYREF,BLM,BRCA2,FANCM,MMS22L,PCNA,RAD51
cellular component disassembly	5.50097659	ACTL6A,APAF1,CCNB1,CCNB2,CDK1,CDKN2A,CHAF1B,CKAP2,CLSPN,DDR2,DKKL1,FANCC,GDF5,GLE1,GSN,HMGB1,HMGB2,KIF14,KIF18A,KIF18B,KIF24,KIF2C,KRT15,MYLK,NDC1,NUP107,NUP153,NUP155,NUP160,NUP188,NUP205,NUP35,NUP43,NUP58,NUP85,NUP88,PLK1,PRSS2,RFWD3,RUVBL1,SNRPB,SNRPD1,STMN1,TOP2A,VRK1
regulation of ubiquitin-protein ligase activity involved in mitotic cell cycle	5.48803941	ANAPC1,ANAPC15,BUB1B,BUB3,CCNB1,CDC20,CDK1,CDK2,FBXO5,MAD2L1,PLK1,UBE2C
cellular response to heat	5.41250615	CHORDC1,HSPH1,MKI67,NDC1,NUP107,NUP153,NUP155,NUP160,NUP188,NUP205,NUP35,NUP43,NUP58,NUP85,NUP88
xenophagy	5.41250615	CHAF1B,DKKL1,HIST1H3A,HIST1H3B,HIST1H3C,HIST1H3F,HIST1H3G,HIST1H3H,HIST1H3I,HIST1H3J,HIST2H3D,MYLK,RFWD3,SNRPB,SNRPD1
DNA replication origin binding	5.40349852	CDC45,MCM10,MCM2,MCM5,ORC5
protein complex binding	5.38221721	ACTL6A,ASPN,BIRC5,CCNB1,CDC20,CDK2,CENPE,CENPH,CKAP5,CLIP3,CLSPN,DDR2,EMP2,GAS2L3,H2AFZ,HLA-DMB,HMGN2,HMGN3,ID1,ITGA7,KIF11,KIF14,KIF15,KIF18A,KIF18B,KIF20A,KIF20B,KIF22,KIF23,KIF24,KIF2C,KIF4A,KIF4B,KIFC1,KRT19,MFGE8,MLH1,MSH2,MSH6,NASP,NDE1,NUSAP1,PCNA,PLK1,PRC1,PROS1,PSRC1,RACGAP1,RAD18,RCC1,REEP4,SKA1,SKA2,SLF1,SMC1A,SMC3,SPAG5,SPDL1,TUBGCP3,TUBGCP4,UHRF1,USP13,VRK1
spindle localization	5.37055739	ASPM,CENPA,ESPL1,GPSM2,NDC80,NDE1,NUSAP1,SPAG5,SPDL1
Fanconi anaemia nuclear complex	5.361036	FANCA,FANCB,FANCC,FANCG,FANCI,FANCM
positive regulation of mitotic cell cycle phase transition	5.35302831	BIRC5,CDC45,CDC6,CDC7,CDCA5,CENPE,DLGAP5,ESPL1,MTBP,PHB2,UBE2C
positive regulation of defense response to virus by host	5.34048294	CHAF1B,DKKL1,HIST1H3A,HIST1H3B,HIST1H3C,HIST1H3F,HIST1H3G,HIST1H3H,HIST1H3I,HIST1H3J,HIST2H3D,MB21D1,MYLK,RFWD3,SNRPB,SNRPD1
negative regulation of cell cycle G1/S phase transition	5.3152279	AURKA,CASP2,CCNB1,CDC25C,CDK1,CDK2,CDKN2B,CENPJ,EZH2,GSG2,GTSE1,PCNA,RFWD3,SUSD2
chromosome passenger complex	5.27742111	AURKA,AURKB,BIRC5,CDCA8
DNA replication, Okazaki fragment processing	5.26752157	DNA2,FEN1,LIG1,RNASEH2A
regulation of mitotic centrosome separation	5.26752157	CHEK1,KIF11,NEK2,RANBP1

meiotic chromosome condensation	5.26752157	NCAPD2,NCAPD3,SMC2,SMC4
U4/U6 x U5 tri-snRNP complex	5.25338684	LSM2,LSM3,LSM5,PPIH,PRPF3,PRPF4,SNRPB
nuclease activity	5.21783911	DCLRE1B,DCLRE1C,DMC1,DNA2,EME1,EXO1,EXOSC2,EXOSC9,FANCM,FEN1,GEN1,MGME1,NEIL3,POLA1,POLE,RAD51,RBBP8,RNASEH2A,RPP30,TDP1,TOE1,XRCC2
regulation of G1/S transition of mitotic cell cycle	5.21756044	AURKA,CASP2,CCNB1,CDC25C,CDC45,CDC6,CDK1,CDK2,CDKN2B,CENPJ,EZH2,GSG2,GTSE1,KIF14,MTBP,PCNA,RFWD3
regulation of exit from mitosis	5.15475059	ANLN,BIRC5,CDCA5,KNTC1,PHB2,UBE2C
regulation of spindle checkpoint	5.15475059	ANAPC15,AURKA,CCNB1,GEN1,GSG2,MAD2L1
somatic hypermutation of immunoglobulin genes	5.15475059	EXO1,MLH1,MSH2,MSH6,POLQ,UNG
nucleobase-containing compound transport	5.11564018	ALYREF,BUD13,CKAP5,DDX39A,GLE1,HNRNPA3,NDC1,NUP107,NUP153,NUP155,NUP160,NUP188,NUP205,NUP35,NUP43,NUP58,NUP85,NUP88,RANBP1,SRSF7,THOC3
nucleotide-excision repair, DNA gap filling	5.09972344	LIG1,PCNA,POLD3,POLE,RFC2,RFC3,RFC4
postreplication repair	5.09151455	BRCA1,DTL,MSH2,PCNA,POLD3,POLE2,RAD18,RFC2,RFC3,RFC4
regulation of ubiquitin-protein transferase activity	5.08625004	ANAPC1,ANAPC15,BUB1B,BUB3,CCNB1,CDC20,CDK1,CDK2,CDKN2A,FBXO5,MAD2L1,MASTL,PLK1,UBE2C,UBE2S
hsa-miR-192:miRTarbase	5.05984143	CDC7,DTL,KIF20B,LMNB2,MAD2L1,MCM10,RACGAP1
regulation of cellular protein catabolic process	5.0553802	ANAPC1,ANAPC15,AURKA,BUB1,BUB1B,BUB3,CCNB1,CDC20,CDK1,CDKN2A,CENPE,CENPF,CEP192,DLGAP5,ESPL1,FBXO5,GEN1,MAD2L1,MTBP,PBK,PLK1,SENP1,STIL,TTK,UBE2C
peptidyl-amino acid modification	5.01953864	ACTL6A,AURKA,AURKB,BIRC5,BLM,BRCA1,BRCA2,CCNB1,CDC7,CDCA8,CDK1,CDK2,CDKN2A,CFAP20,CHEK1,CLIP3,CLSPN,CNTRL,CTCF,DDR2,DNMT1,EZH2,FAM72A,GSG2,HAT1,HIST1H1B,HIST1H1C,HIST1H1D,HIST1H1E,HMBS,HSPH1,INCENP,MASTL,MELK,NDC1,NOP58,NUP107,NUP153,NUP155,NUP160,NUP188,NUP205,NUP35,NUP43,NUP58,NUP85,NUP88,PARP1,PAXIP1,PBK,PCNA,PHF19,PLK1,POLE3,PPIH,PROS1,QPCT,RUVBL1,SENP1,SMC1A,SMC3,SUV39H1,SUV39H2,TAF5,TOP2A,TRIM28,TRPM4,TTK,VRK1
somatic diversification of immune receptors via somatic mutation	4.97778679	EXO1,MLH1,MSH2,MSH6,POLQ,UNG
regulation of ligase activity	4.95428158	ANAPC1,ANAPC15,BUB1B,BUB3,CCNB1,CDC20,CDK1,CDK2,CDKN2A,FBXO5,MAD2L1,MASTL,PLK1,UBE2C,UBE2S
positive regulation of cell cycle phase transition	4.95025639	BIRC5,CDC45,CDC6,CDC7,CDCA5,CENPE,DLGAP5,ESPL1,MTBP,PHB2,UBE2C
negative regulation of ubiquitin-protein transferase activity	4.94061983	ANAPC1,ANAPC15,BUB1B,BUB3,CCNB1,CDC20,CDK1,CDK2,CDKN2A,FBXO5,MAD2L1,UBE2C
hsa-miR-302a:mirSVR_conserved_highEffect-0.5	4.93978381	ACTL6A,ANAPC15,ARHGAP11B,ARL6IP6,ASF1A,ASF1B,ATAD2,AUNIP,BTG3,BUB1,C18orf54,C19orf48,CCDC15,CCNE2,CDCA2,CDCA7,CDK1,CENPJ,CENPO,CEP135,CEP55,CEP97,CKS1B,DCLRE1C,DDIAS,DNAJC9,DRAM1,ECT2,ERLIN1,FANCD2,FANCM,FNDC3A,GABPB1,GPN3,GPR137C,HAUS8,HELLS,HIST1H2BB,HMGB1,HMGN3,ITGB3BP,KIAA0586,KNSTRN,KPNA2,MCM10,MCMBP,MLH1,MMS22L,MPHOSPH9,MYO19,NOTCH2NL,NUP205,OSGEPL1,PARPBP,PBK,PLK4,POC1A,POLQ,PPIH,PRIM1,PRR11,PRTFDC1,RACGAP1,RAD18,RAD51AP1,RBL1,RPP30,RRM1,SENP1,SHC4,SHCBP1,SRRT,SUV39H1,TTI1,TUBGCP4,ZNF367

endodeoxyribonuclease activity	4.93950075	DCLRE1B,DCLRE1C,DMC1,DNA2,EME1,EXO1,FEN1,GEN1,MGME1,NEIL3,RAD51,RBBP8,XRCC2
cell division site part	4.8944923	ANLN,CEP55,ECT2,MASTL,MYLK,NDE1,PLK4,RACGAP1,TUBGCP3,TUBGCP4
cell division site	4.8944923	ANLN,CEP55,ECT2,MASTL,MYLK,NDE1,PLK4,RACGAP1,TUBGCP3,TUBGCP4
DNA replication factor C complex	4.81169642	PCNA,RFC2,RFC3,RFC4
replication fork protection complex	4.81169642	CDC45,GINS2,GINS4,MCM10
DNA ligase (ATP) activity	4.81059719	LIG1,PARP1,PARP2,PARP3
chromatin DNA binding	4.74417277	ACTL6A,CTCF,EZH2,H2AFZ,HIST1H1A,HIST1H1B,HIST1H1C,HIST1H1D,HIST1H1E,HMGN2,HMGN3,RCC1
germ cell nucleus	4.74333845	AURKA,BLM,H2AFX,MLH1,NCAPD3,TOPBP1,TRIP13
nuclear replisome	4.63061922	MCM3,POLA1,POLA2,POLD3,POLE,POLE2,POLE3
DNA-directed DNA polymerase activity	4.62893049	POLA1,POLA2,POLD3,POLE,POLE2,POLE3,POLQ
nuclear membrane	4.57502674	BRIP1,DCTN5,DTL,LBR,LMNB1,LMNB2,MRTO4,NDC1,NEMP1,NRM,NUP107,NUP153,NUP155,NUP205,NUP35,NUP58,NUP85,RCC1,SENP1,SEPHS1,TMEM109,TMEM97,TMPO,TRA2B
small nuclear ribonucleoprotein complex	4.56136398	LSM2,LSM3,LSM5,NOLC1,PPIH,PRPF3,PRPF4,SNRNP40,SNRPB,SNRPD1
GINS complex	4.4581758	GINS1,GINS2,GINS4
centralspindlin complex	4.4581758	ECT2,KIF23,RACGAP1
nuclear pore inner ring	4.4581758	NUP155,NUP188,NUP205
Ctf18 RFC-like complex	4.45500795	DSCC1,RFC2,RFC3,RFC4
DNA ligase activity	4.45391524	LIG1,PARP1,PARP2,PARP3
spindle pole centrosome	4.4425092	AURKA,AURKB,DLGAP5,KIF1C,NDE1
nuclear matrix	4.42399438	BLM,CENPF,CENPW,HAT1,KIF4A,KIF4B,LMNB1,PAXIP1,PHB2,POLA1,RUVBL1,SMC3,UHRF1

Supplemental Table 3. HUMAN mTOR Activated MVPC Reactome Category Lists (Figure 2G)

Diseases of programmed cell death
Senescence-Associated Secretory Phenotype (SASP)
DNA Damage/Telomere Stress Induced Senescence
Telomere Maintenance
Cellular Senescence
Oxidative Stress Induced Senescence
Apoptosis induced DNA fragmentation
Formation of Senescence-Associated Heterochromatin Foci (SAHF)
Apoptotic execution phase
Defective Intrinsic Pathway for Apoptosis
Telomere Extension by Telomerase
Cellular response to heat stress
Glycolysis
Glucose metabolism
Ub-specific processing proteases
Protein ubiquitination
Intra-Golgi and retrograde Golgi-to-ER traffic
RHO GTPase Effectors
RHO GTPases activate PKNs
RHO GTPases Activate Formins
RHOD GTPase cycle
RHOC GTPase cycle
RHOF GTPase cycle
RHOB GTPase cycle
Formation of the beta-catenin:TCF transactivating complex
TCF dependent signaling in response to WNT
Signaling by WNT
Pre-NOTCH Transcription and Translation
Pre-NOTCH Expression and Processing
Signaling by NOTCH
Activation of anterior HOX genes in hindbrain development during early embryogenesis
Activation of HOX genes during differentiation
Epigenetic regulation of gene expression
Estrogen-dependent gene expression
Transcriptional regulation by RUNX1
Signaling by Nuclear Receptors
AURKA Activation by TPX2
Transcription of E2F targets under negative control by DREAM complex
SIRT1 negatively regulates rRNA expression
DNA Repair
Homology Directed Repair
DNA Double-Strand Break Repair
DNA Double Strand Break Response
Diseases of DNA repair
Defective HDR through Homologous Recombination (HRR) due to BRCA1 loss-of-function
Defective HDR through Homologous Recombination Repair (HRR) due to PALB2 loss of BRCA1 binding function
Defective HDR through Homologous Recombination Repair (HRR) due to PALB2 loss of BRCA2/RAD51/RAD51C binding function
PCNA-Dependent Long Patch Base Excision Repair
Recognition of DNA damage by PCNA-containing replication complex
Mismatch Repair
Cell Cycle Checkpoints
DNA Replication
G2/M Checkpoints
G1/S Transition

Supplemental Table 4. Tsc2KD mTOR Activated MVPC Gene Lists for STRING Major Pathways (Fig5B)

MCL Cluster	mgi symbol	Pathway	direction	sig	logFC	STRING_id
		**mTOR signaling & Autophagy**				
3	***RPS6KA2***	mTOR signaling	up	0.81	0.51	10090.ENSMUSP00000024575
3	***TTI1***	mTOR signaling	down	0.3	-0.22	10090.ENSMUSP00000029179
3	***RRAGC***	mTOR signaling/ autophagy	up	0.36	0.23	10090.ENSMUSP00000030399
3	***SESN2***	mTOR signaling	up	0.74	0.5	10090.ENSMUSP00000030724
3	***UVRAG***	autophagy	up	0.69	0.32	10090.ENSMUSP00000045297
3	***FNIP1***	mTOR signaling	down	0.36	-0.25	10090.ENSMUSP00000049026
3	***LAMP2***	autophagy	down	0.6	-0.33	10090.ENSMUSP00000052283
3	***DAPK1***	autophagy	up	5.95	1.8	10090.ENSMUSP00000076666
3	***TSC2***	mTOR signaling/autophagy/ AMPK signaling	down	0.48	-0.27	10090.ENSMUSP00000094986
3	***ACACA***	AMPK signaling	down	0.47	-0.32	10090.ENSMUSP00000099490
3	***AKT1S1***	mTOR signaling/autophagy/ AMPK signaling	up	0.63	0.3	10090.ENSMUSP00000103512
3	***PIK3C3***	autophagy	down	0.51	-0.26	10090.ENSMUSP00000111479
3	***SGK1***	mTOR signaling	down	7.32	-1.82	10090.ENSMUSP00000114074
		**P53/PTEN PROTEOSOME Subunit**				
1	***PSMA4***	p53/PTEN/Proteosome	down	0.69	-0.27	10090.ENSMUSP00000034848
1	***PSMB2***	p53/PTEN/Proteosome	up	0.31	0.2	10090.ENSMUSP00000030642
1	***PSMB6***	p53/PTEN/Proteosome	down	0.48	-0.27	10090.ENSMUSP00000018430
1	***PSMB7***	p53/PTEN/Proteosome	down	0.38	-0.2	10090.ENSMUSP00000028083
1	***PSMC2***	p53/PTEN/Proteosome	down	0.36	-0.23	10090.ENSMUSP00000030769
1	***PSMD10***	p53/PTEN/Proteosome	down	0.74	-0.3	10090.ENSMUSP00000033805
1	***PSMD12***	p53/PTEN/Proteosome	down	0.35	-0.23	10090.ENSMUSP00000021063
1	***PSMD8***	p53/PTEN/Proteosome	up	4.39	0.9	10090.ENSMUSP00000051657
		**WNT Signaling**				
7	***ROR2***	Wnt signaling	down	5.49	-1.4	10090.ENSMUSP00000021918
7	***FZD6***	Wnt signaling	down	1.53	-0.81	10090.ENSMUSP00000022906
7	***DKK2***	Wnt signaling	down	6.12	-1.83	10090.ENSMUSP00000029665
7	***SFRP1***	Wnt signaling	up	7.3	3.14	10090.ENSMUSP00000033952
7	***DIXDC1***	Wnt signaling	down	3.93	-1.06	10090.ENSMUSP00000034566
7	***FZD5***	Wnt signaling	up	2.93	1.02	10090.ENSMUSP00000067783
7	***CTHRC1***	Wnt signaling	down	3.93	-1.88	10090.ENSMUSP00000070018
7	***FZD2***	Wnt signaling	down	0.46	-0.32	10090.ENSMUSP00000091463
7	***NLK***	Wnt signaling	up	0.63	0.34	10090.ENSMUSP00000119345
1	***AP2M1***	Wnt signaling	up	0.44	0.22	10090.ENSMUSP00000007216
1	***CLTB***	Wnt signaling	up	0.27	0.21	10090.ENSMUSP00000089198
1	***AP2A2***	Wnt signaling	up	0.75	0.35	10090.ENSMUSP00000003038
1	***AP2S1***	Wnt signaling	up	0.46	0.26	10090.ENSMUSP00000083281
1	***RBX1***	Hedehog off/ Beta-catenin destruction	down	0.37	-0.26	10090.ENSMUSP00000023036
9	***SCRIB***	Wnt signaling	down	0.67	-0.4	10090.ENSMUSP00000002603
9	***DVL3***	Wnt signaling	up	0.38	0.27	10090.ENSMUSP00000003318
9	***DACT3***	Wnt signaling	up	0.66	0.45	10090.ENSMUSP00000104133
9	***PRICKLE2***	Wnt signaling	down	1	-0.44	10090.ENSMUSP00000109073
9	***VANGL1***	Wnt signaling	up	1.41	0.45	10090.ENSMUSP00000125043
		**AMPK/PPAR Signaling**				
10	***PPARG***	AMPK/PPAR	up	3.45	1.12	10090.ENSMUSP00000000450
10	***HPS3***	AMPK/PPAR	up	0.41	0.28	10090.ENSMUSP00000012580
10	***GOT2***	AMPK/PPAR	down	0.5	-0.3	10090.ENSMUSP00000034097
10	***SCD1***	AMPK/PPAR	down	1.73	-0.63	10090.ENSMUSP00000036936
10	***FASN***	AMPK/PPAR	down	0.78	-0.51	10090.ENSMUSP00000052872

Supplementary Table 5. Seurat FindMarkers TSC2KD vs WT DE (p.adj <0.05, lfc >1) I				
gene	lfc	pct1	pct2	pval.adj
4931406P1	1.214599	0.788	0.236	8.1926E-198
Ace	2.151262	0.982	0.475	0
Acer2	1.089443	0.72	0.232	4.9771E-157
Actb	1.09357	1	0.972	8.8583E-173
Actg1	1.835208	0.96	0.468	1.4403E-286
Acvrl1	1.555615	0.972	0.511	1.3388E-287
Adgre5	1.637613	0.937	0.417	1.0374E-270
Adgrf5	2.178809	0.999	0.712	0
Adgrl4	1.911485	0.953	0.282	0
Aldh2	1.636158	0.895	0.245	6.5251E-279
Aldoa	1.081901	0.663	0.132	3.3026E-169
Aplp2	1.090219	0.799	0.325	9.9777E-163
App	1.052479	0.859	0.441	5.7875E-155
Aqp1	1.999609	0.984	0.525	0
Arap2	1.126771	0.686	0.242	1.129E-137
Arhgap29	1.177248	0.766	0.241	1.0657E-179
Arhgap31	1.186505	0.728	0.167	7.44E-193
Arhgef12	1.256996	0.806	0.32	8.5627E-180
Arid5b	1.108939	0.672	0.206	7.9473E-141
Atf3	1.15326	0.429	0.108	9.33275E-78
Atp1a1	1.183516	0.718	0.181	1.4007E-178
AY036118	3.022201	0.999	0.773	0
B2m	1.847239	1	0.872	0
BC028528	1.48237	0.902	0.407	4.1257E-239
Bmp6	1.045438	0.698	0.225	2.7523E-146
Bmpr2	2.352936	1	0.729	0
Bst2	1.038434	0.649	0.176	9.6731E-143
Btg2	1.52524	0.76	0.145	1.2247E-219
Calcrl	1.979376	1	0.938	0
Calm1	1.448266	0.87	0.303	2.9883E-231
Cav1	1.705512	0.989	0.685	1.0398E-292
Cav2	1.279708	0.847	0.329	1.4252E-201
Cavin2	1.438948	0.949	0.644	9.048E-201
Cbfa2t3	1.045039	0.661	0.197	3.1229E-138
Cd200	1.213728	0.767	0.332	2.9329E-144
Cd36	1.722228	1	0.876	9.4624E-300
Cd47	1.781729	0.957	0.444	8.3087E-294
Cd74	2.685991	0.928	0.329	0
Cd9	1.755807	0.955	0.421	1.7321E-289
Cd93	1.982465	0.993	0.61	0
Cdh5	1.881753	0.998	0.794	0
Cdk19	1.184672	0.593	0.203	7.031E-102
Cdkn1a	1.808514	0.763	0.117	8.4149E-241
Cebpd	1.912479	0.819	0.199	4.2979E-236
Cfl1	1.061119	0.762	0.252	1.137E-168
Chchd2	1.282368	0.824	0.263	9.6185E-212
Cldn5	2.484248	0.998	0.679	0
Clec14a	1.890162	0.963	0.427	8.5675E-299
Clec1a	1.768209	0.93	0.391	1.3977E-268
Clec2d	1.601857	0.963	0.578	8.535E-260
Clic1	1.187983	0.759	0.194	7.0407E-195
Clic4	1.011453	0.742	0.319	4.328E-130
Clic5	1.745414	0.955	0.506	5.6583E-283
Clk1	1.268059	0.742	0.158	6.642E-202

Cox8a	1.042392	0.753	0.252	2.2976E-163
Crip1	1.124228	0.742	0.387	2.5561E-105
Crip2	1.225847	0.817	0.27	4.9725E-199
Csnk1a1	1.14734	0.753	0.235	4.3457E-176
Cst3	1.238084	0.826	0.272	1.9123E-199
Ctla2a	1.935657	0.941	0.435	2.8078E-275
Ctnna1	1.331736	0.83	0.278	5.6053E-213
Cxcl12	1.992958	0.943	0.556	1.7998E-217
Cyyr1	1.848712	0.977	0.473	0
Ddx3x	1.069772	0.696	0.184	1.1394E-161
Ddx5	1.666974	0.981	0.546	9.4306E-294
Dock9	1.097511	0.684	0.154	1.4033E-171
Dusp1	1.442788	0.797	0.294	2.4608E-180
Dusp6	1.060826	0.594	0.164	7.3154E-120
Dynll1	1.214782	0.767	0.221	1.821E-190
Ece1	1.413642	0.84	0.257	1.209E-226
Eef1a1	1.965239	0.994	0.638	0
Efnb1	1.03806	0.698	0.213	1.553E-148
Efnb2	1.462483	0.802	0.294	7.5152E-189
Egfl7	1.916912	0.996	0.665	0
EGFP-REPO	2.630633	0.846	0.457	1.0823E-185
Ehd4	1.823636	0.968	0.53	3.9295E-290
Eif1	2.071454	0.98	0.424	0
Eif4g2	1.087091	0.721	0.211	1.8295E-164
Eng	2.06213	0.969	0.372	0
Epas1	2.318299	0.999	0.849	0
Esam	1.352429	0.896	0.394	1.2633E-228
Ets2	1.087359	0.687	0.185	1.4333E-157
Fau	1.858756	0.984	0.537	0
Fkbp1a	1.180982	0.853	0.414	9.2828E-175
Flt1	1.855076	0.927	0.332	8.8178E-283
Fmo1	1.647347	0.924	0.274	3.2834E-294
Fmo2	1.105476	0.598	0.119	7.0705E-146
Foxf1	1.362441	0.878	0.397	2.3395E-207
Foxp1	1.322522	0.938	0.623	1.1109E-196
Fth1	1.553105	0.892	0.328	5.4291E-248
Ftl1	1.407675	0.848	0.263	1.8691E-226
Fzd4	1.026387	0.659	0.188	2.0476E-140
Gata2	1.243655	0.74	0.208	8.6047E-180
Gimap6	1.008004	0.619	0.137	8.1466E-143
Gm42418	2.818366	1	0.997	0
Gnai2	1.448193	0.931	0.468	1.2489E-245
Gpihbp1	1.98317	0.948	0.419	1.7817E-289
Gstm1	1.068924	0.747	0.282	6.2504E-151
H2-Aa	1.527145	0.773	0.214	3.2254E-204
H2-Ab1	1.869769	0.845	0.254	2.3535E-246
H2-D1	2.612403	1	0.688	0
H2-Eb1	1.606745	0.813	0.231	6.2712E-225
H2-K1	2.283837	1	0.704	0
H2-Q4	1.54869	0.882	0.258	2.1321E-267
H2-Q6	1.560076	0.852	0.234	9.4034E-250
H2-Q7	2.128849	0.974	0.334	0
H2-T23	1.514939	0.849	0.236	6.2167E-239
H3f3a	1.555466	0.927	0.323	8.1852E-280
H3f3b	1.81906	0.976	0.468	5.0555E-301
Hilpda	1.521259	0.736	0.236	1.9911E-172

Hmcn1	1.071365	0.717	0.309	1.0377E-123
Hmgb1	1.334184	0.92	0.477	4.5952E-220
Hnrnpa2b1	1.11472	0.694	0.199	1.8731E-160
Hnrnpk	1.008944	0.713	0.267	3.5087E-139
Hopx	1.108608	0.622	0.181	2.0707E-127
Hpgd	2.501852	0.994	0.682	0
Hsp90ab1	1.347672	0.86	0.392	3.7286E-192
Hsp90b1	1.170037	0.737	0.257	4.0317E-159
Hspa8	1.370104	0.842	0.264	1.3445E-224
Icam2	1.317034	0.86	0.339	2.9593E-214
Id1	1.212251	0.667	0.146	3.0918E-167
Id3	1.66445	0.891	0.314	5.2197E-254
Ier2	1.256028	0.678	0.176	7.3073E-156
Ier3	1.879125	0.835	0.401	2.3631E-164
Ifitm2	1.454374	0.932	0.448	3.6604E-243
Ifitm3	1.853597	0.997	0.755	0
Il6st	1.077986	0.635	0.113	2.8022E-165
Itga1	1.846815	0.959	0.527	1.3722E-282
Itgb1	1.321552	0.876	0.367	6.5539E-214
Itm2b	2.495808	1	0.773	0
Jun	2.096796	0.994	0.631	0
Junb	1.788638	0.793	0.329	1.7921E-152
Jund	1.79043	0.838	0.209	1.4329E-246
Jup	1.18043	0.841	0.406	2.8503E-174
Kdr	1.003567	0.68	0.205	1.5694E-140
Klf2	2.209947	0.96	0.376	0
Klf4	1.804275	0.927	0.449	2.5995E-234
Klf7	1.247953	0.777	0.245	4.5582E-186
Klf9	1.417905	0.754	0.133	5.3065E-224
Klhl5	1.178807	0.76	0.254	1.0195E-174
Lamp1	1.256426	0.819	0.293	1.6332E-195
Laptm4a	1.19423	0.852	0.392	4.4765E-187
Lars2	2.355988	0.989	0.4	0
Lpl	2.216451	0.835	0.095	2.8251E-302
Ly6a	2.606467	0.999	0.636	0
Ly6c1	2.468103	0.99	0.468	0
Ly6e	1.979861	0.987	0.611	0
Macf1	1.230822	0.789	0.275	3.3447E-181
Malat1	2.51375	0.999	0.995	0
Marcks	1.054388	0.607	0.225	6.4622E-101
Mcl1	1.228804	0.711	0.115	1.1927E-204
Msn	1.244258	0.861	0.39	4.2411E-199
Myl12a	1.050349	0.793	0.346	5.6163E-150
Myl12b	1.165366	0.839	0.34	3.1431E-187
Myl6	1.201123	0.864	0.415	1.0874E-173
Myzap	1.33885	0.795	0.27	3.7179E-197
Nfib	1.544177	0.916	0.392	2.8789E-253
Nfkbia	1.233064	0.725	0.157	4.6683E-190
Nrp1	1.438711	0.88	0.304	3.252E-237
Ntrk2	1.09509	0.59	0.171	4.1864E-115
Oaz1	1.162685	0.737	0.171	5.0264E-195
Pcdh17	1.070428	0.673	0.259	3.0846E-120
Pde4b	1.460356	0.724	0.16	7.9074E-197
Pecam1	1.919064	0.989	0.497	0
Pitpnc1	1.212671	0.788	0.368	1.8193E-151
Pltp	1.596928	0.979	0.61	2.1979E-259

Podxl	1.383139	0.875	0.387	3.4377E-212
Ppia	1.714979	0.977	0.474	0
Ppp1r2	1.095895	0.703	0.21	5.3365E-156
Ptma	1.637456	0.995	0.771	3.0965E-285
Ptprb	2.421397	0.998	0.698	0
Qk	1.204832	0.862	0.424	5.4497E-185
Rabac1	1.03652	0.686	0.154	1.3673E-168
Rac1	1.069126	0.788	0.309	3.1022E-170
Ramp2	2.39013	0.999	0.664	0
Rap1a	1.14949	0.673	0.115	3.2134E-186
Rbm39	1.335513	0.814	0.228	1.388E-222
Rdx	1.846481	0.944	0.479	1.3908E-267
Rhoa	1.16109	0.853	0.389	2.7824E-185
Rsrp1	1.135685	0.695	0.132	4.6167E-186
S100a13	1.101197	0.729	0.198	7.9025E-174
S100a16	1.077012	0.726	0.242	3.6463E-156
S1pr1	2.141735	0.985	0.462	0
Selenop	1.475866	0.865	0.323	4.1322E-226
Sema3c	1.484679	0.93	0.797	4.235E-169
Sema3g	1.301805	0.753	0.219	2.4543E-184
Serinc3	2.22226	0.99	0.421	0
Sgk1	1.092486	0.666	0.302	5.3567E-100
Sh3glb1	1.199226	0.752	0.219	2.2855E-183
Slc3a2	1.361146	0.78	0.223	3.0482E-200
Slc43a3	1.459529	0.84	0.213	1.1972E-247
Slc6a6	1.250271	0.684	0.374	3.17558E-93
Slc9a3r2	1.608982	0.898	0.3	1.0408E-264
Slco2a1	2.038395	0.976	0.478	0
Slfn5	1.297719	0.731	0.174	4.9099E-191
Smad6	1.238192	0.722	0.157	5.3124E-190
Smad7	1.505216	0.834	0.208	6.4987E-244
Socs3	1.403665	0.625	0.233	3.6E-108
Son	1.241461	0.796	0.212	3.4371E-212
Sparcl1	1.431836	0.709	0.265	1.3924E-146
Sptbn1	2.038419	0.994	0.654	0
Srgn	1.135957	0.708	0.188	4.1666E-165
Srrm2	1.099765	0.717	0.194	5.9989E-170
Srsf5	1.259187	0.767	0.177	1.5191E-210
Stmn1	1.006	0.649	0.177	1.2337E-139
Syne1	1.097929	0.622	0.151	1.7643E-142
Tagln2	1.032912	0.712	0.265	1.2737E-132
Tax1bp1	1.198139	0.686	0.149	3.1708E-176
Tbx3	1.053356	0.667	0.208	3.4301E-138
Tcf4	1.502064	0.929	0.461	6.6478E-237
Tcn2	1.239288	0.737	0.167	1.1123E-195
Tek	1.483143	0.871	0.277	7.2317E-249
Thbd	1.383977	0.906	0.514	2.0706E-187
Timp3	1.145521	0.569	0.12	3.0238E-129
Tjp1	1.345788	0.81	0.277	1.8616E-205
Tm4sf1	1.541936	0.902	0.436	4.2714E-216
Tmbim6	1.016207	0.747	0.269	7.2047E-154
Tmem100	2.050862	0.996	0.796	0
Tmem176a	1.13223	0.693	0.162	1.24E-171
Tmem176b	1.103926	0.708	0.191	1.4585E-165
Tmem2	1.03577	0.675	0.302	8.3915E-99
Tmem59	1.036069	0.712	0.191	8.4537E-167

Tmsb4x	1.770016	0.997	0.851	0
Tpt1	2.312316	0.996	0.563	0
Tspan13	1.378619	0.886	0.378	5.7801E-221
Tspan18	1.218189	0.732	0.186	4.2593E-185
Tspan7	1.692918	0.991	0.69	1.1353E-297
Txnip	1.209831	0.792	0.387	5.2932E-148
Uba52	1.60245	0.945	0.461	2.4887E-266
Ubb	2.135685	0.991	0.464	0
Ucp2	1.396412	0.771	0.184	1.4279E-215
Vegfa	1.556494	0.907	0.402	4.7333E-236
Vim	1.095229	0.786	0.434	1.6255E-121
Vwf	1.307761	0.533	0.08	1.5872E-134
Ybx1	1.914986	0.966	0.409	0
Zbtb20	1.193086	0.724	0.281	3.0441E-140
Zfp36	1.428747	0.691	0.221	4.3461E-142
Zfp36l1	1.051602	0.665	0.188	6.9871E-144

Seurat FindMarkers TSC2KD vs WT DE (p.adj <0.05, lfc >1) MVPC Cluster 1				
gene	lfc	pct1	pct2	pval.adj
4931406P1	1.158504	0.768	0.214	1.4262E-232
Ace	2.294776	0.995	0.649	0
Acer2	1.155118	0.752	0.252	1.2194E-204
Actb	1.098962	0.998	0.965	5.3349E-224
Actg1	1.702239	0.938	0.416	0
Acvrl1	1.593846	0.977	0.548	0
Adgre5	1.682747	0.964	0.477	0
Adgrf5	2.286165	0.999	0.684	0
Adgrl3	1.111238	0.686	0.165	2.1697E-201
Adgrl4	2.091457	0.975	0.359	0
Aldh2	2.009239	0.972	0.38	0
Aplp2	1.122206	0.785	0.324	4.7711E-198
App	1.100279	0.866	0.455	2.3848E-205
Aqp1	2.116993	0.981	0.5	0
Arhgap29	1.210073	0.761	0.229	3.3235E-223
Arhgap31	1.233236	0.759	0.187	9.7199E-248
Arhgef12	1.330662	0.85	0.347	1.6534E-248
Atp1a1	1.334136	0.809	0.243	7.3663E-256
Atrx	1.09122	0.639	0.173	1.3046E-168
AY036118	2.846511	1	0.794	0
B2m	1.747298	0.999	0.858	0
BC028528	1.448973	0.904	0.387	2.729E-294
Bmp6	1.02103	0.714	0.25	1.097E-170
Bmpr2	2.381858	1	0.778	0
Bst2	1.215652	0.749	0.216	5.4452E-225
Btg2	1.548491	0.757	0.159	1.4642E-259
Calcrl	1.879029	1	0.951	0
Calm1	1.335842	0.854	0.31	5.6752E-258
Cav1	1.766054	0.992	0.64	0
Cav2	1.199373	0.837	0.361	5.2803E-232
Cavin2	1.248732	0.922	0.572	2.2288E-217
Cbfa2t3	1.025361	0.668	0.211	2.6617E-165
Cd200	1.265184	0.84	0.415	1.0559E-202
Cd36	1.779771	0.999	0.899	0
Cd47	1.741946	0.956	0.416	0
Cd74	1.663739	0.611	0.105	8.2827E-199
Cd9	1.463749	0.87	0.287	1.0782E-290
Cd93	1.798016	0.978	0.638	0
Cdc42bpa	1.11417	0.625	0.116	1.8E-195
Cdh5	1.924699	0.999	0.791	0
Cdkn1a	1.569576	0.727	0.112	7.5401E-267
Cebpd	1.921635	0.804	0.175	2.8564E-286
Chchd2	1.222062	0.821	0.263	1.2099E-254
Cldn5	2.475502	0.996	0.677	0
Clec14a	1.921108	0.977	0.588	0
Clec1a	1.751946	0.914	0.364	0
Clec2d	1.650144	0.969	0.553	0
Clic1	1.106348	0.73	0.157	5.8148E-233
Clic4	1.036497	0.772	0.347	5.2231E-170
Clic5	1.453737	0.868	0.362	6.0106E-269
Clk1	1.287838	0.729	0.146	1.7333E-245
Col4a1	1.023187	0.696	0.258	1.2609E-156
Crim1	1.184918	0.738	0.231	1.4833E-201
Crip2	1.150467	0.796	0.294	3.5603E-215

Csnk1a1	1.173205	0.771	0.232	2.3723E-232
Cst3	1.444696	0.892	0.351	3.3442E-279
Ctla2a	1.98046	0.961	0.48	0
Ctnna1	1.202827	0.783	0.275	2.8312E-218
Ctsl	1.206466	0.775	0.241	4.6239E-225
Cxcl12	1.813226	0.873	0.487	1.4088E-198
Cyp4b1	1.341936	0.86	0.415	1.0834E-232
Cyyr1	1.706524	0.963	0.459	0
Ddx3x	1.155698	0.717	0.168	2.0938E-222
Ddx5	1.709872	0.972	0.512	0
Dock9	1.393418	0.804	0.193	3.2365E-284
Dusp1	1.505886	0.836	0.346	3.0041E-229
Dynll1	1.025837	0.731	0.222	4.0913E-199
Ece1	1.898975	0.972	0.399	0
Edn1	1.146568	0.717	0.601	7.04676E-49
Eef1a1	2.004822	0.996	0.626	0
Efnb1	1.058519	0.63	0.112	6.2099E-197
Efnb2	1.854901	0.963	0.532	0
Egfl7	1.999043	0.995	0.666	0
EGFP-REPO	2.724278	0.897	0.525	7.8502E-285
Ehd4	1.857203	0.978	0.569	0
Eif1	1.972486	0.977	0.384	0
Eif4g2	1.027742	0.712	0.222	1.3908E-189
Eng	2.292293	0.995	0.584	0
Epas1	2.285934	0.999	0.816	0
Esam	1.386862	0.944	0.444	0
Esm1	1.604258	0.485	0.132	2.7156E-107
Fau	1.782667	0.975	0.495	0
Fkbp1a	1.212293	0.877	0.442	5.4903E-226
Flt1	2.186697	0.992	0.48	0
Fmo1	1.85338	0.967	0.394	0
Fmo2	1.613501	0.819	0.211	8.0858E-288
Fnbp1l	1.075587	0.653	0.13	5.8363E-200
Foxf1	1.304177	0.854	0.381	4.2475E-234
Foxp1	1.207349	0.9	0.546	4.0018E-201
Fth1	1.517138	0.897	0.329	5.5403E-303
Ftl1	1.330992	0.825	0.222	3.3449E-273
Fzd4	1.0244	0.653	0.202	2.2649E-162
Gata2	1.168141	0.685	0.164	2.2993E-204
Gm42418	2.577395	1	0.999	0
Gnai2	1.461614	0.935	0.417	0
Gpihbp1	2.215637	0.98	0.518	0
Gstm1	1.101375	0.787	0.329	4.4255E-193
H2-Ab1	1.226724	0.593	0.11	2.1157E-180
H2-D1	2.488995	1	0.685	0
H2-K1	2.12986	0.998	0.676	0
H2-Q4	1.326414	0.813	0.225	4.8264E-267
H2-Q6	1.271291	0.767	0.193	2.9873E-244
H2-Q7	1.7524	0.906	0.279	0
H2-T23	1.296211	0.764	0.202	1.2991E-239
H3f3a	1.416206	0.916	0.321	0
H3f3b	1.703194	0.943	0.428	0
Hilpda	1.758848	0.891	0.358	0
Hmgb1	1.389008	0.922	0.439	8.777E-286
Hnrnpa2b1	1.057767	0.72	0.225	3.1252E-194
Hopx	1.008326	0.638	0.238	1.2227E-133

Hpgd	2.25384	0.99	0.723	0
Hsp90ab1	1.306332	0.868	0.419	3.4347E-229
Hsp90b1	1.174205	0.747	0.263	9.6564E-198
Hspa8	1.428591	0.855	0.256	1.9792E-294
Icam2	1.274434	0.845	0.335	2.6438E-236
Id1	1.202046	0.698	0.186	2.9884E-200
Id3	1.756765	0.922	0.385	0
Ier2	1.064081	0.593	0.144	4.5726E-156
Ier3	1.621643	0.714	0.259	2.1685E-169
Ifitm2	1.426027	0.934	0.489	4.3155E-285
Ifitm3	1.673042	0.996	0.8	0
Il6st	1.185153	0.688	0.14	5.9186E-223
Itga1	1.657419	0.933	0.428	0
Itga6	1.107677	0.68	0.193	1.419E-183
Itgb1	1.324002	0.864	0.347	1.5969E-263
Itm2b	2.362661	1	0.756	0
Jun	1.983127	0.985	0.624	0
Junb	1.714994	0.726	0.259	5.8125E-176
Jund	1.582978	0.766	0.195	3.1453E-247
Jup	1.022904	0.737	0.296	5.5673E-164
Kdr	1.252395	0.785	0.274	1.2548E-217
Kitl	1.409213	0.746	0.229	8.1402E-217
Klf2	2.020643	0.937	0.391	0
Klf4	1.61515	0.827	0.328	4.524E-230
Klf7	1.199684	0.771	0.233	1.31E-223
Klf9	1.499002	0.786	0.161	1.7156E-284
Klhl5	1.076116	0.716	0.208	4.5768E-200
Lamp1	1.336086	0.858	0.309	3.3502E-271
Laptm4a	1.136564	0.861	0.405	7.6069E-222
Lars2	2.301217	0.975	0.378	0
Lpl	2.748915	0.95	0.275	0
Ly6a	2.520921	1	0.757	0
Ly6c1	2.495361	0.995	0.487	0
Ly6e	2.01911	0.997	0.744	0
Lyve1	1.571565	0.544	0.193	8.5866E-109
Macf1	1.307527	0.787	0.253	1.3642E-233
Malat1	2.71683	1	0.996	0
Marcks	1.300856	0.7	0.17	3.5148E-216
Mcam	1.116845	0.707	0.241	4.0623E-182
Mcl1	1.143663	0.679	0.133	2.4538E-218
Mctp1	1.155989	0.633	0.095	5.4875E-213
Mgll	1.015143	0.543	0.088	3.8179E-164
Morf4l1	1.085004	0.761	0.278	5.9469E-197
Msn	1.326171	0.866	0.386	1.7055E-251
Myl12a	1.049296	0.821	0.369	6.0113E-196
Myl12b	1.150153	0.819	0.324	6.6721E-214
Myl6	1.12196	0.842	0.384	1.0066E-208
Myzap	1.219929	0.764	0.263	5.5548E-213
Nfib	1.55531	0.93	0.435	0
Nfkbia	1.257856	0.743	0.184	1.7026E-229
Notch1	1.205389	0.74	0.241	1.0964E-205
Nrp1	1.476824	0.902	0.369	1.2102E-292
Oaz1	1.012073	0.69	0.157	1.5778E-208
Pcdh17	1.184874	0.678	0.222	8.7897E-172
Pde4b	1.200827	0.645	0.15	2.6365E-189
Pecam1	1.924542	0.992	0.541	0

Plat	1.071635	0.498	0.071	9.7126E-153
Pltp	1.557976	0.959	0.653	8.5268E-292
Plvap	1.910872	0.922	0.572	5.9358E-297
Podxl	1.117041	0.779	0.312	6.5756E-194
Ppia	1.663838	0.967	0.445	0
Prrc2c	1.088716	0.693	0.223	1.618E-178
Ptma	1.700334	0.993	0.731	0
Ptprb	2.592445	1	0.833	0
Qk	1.203639	0.854	0.366	5.3622E-235
Rabac1	1.109264	0.711	0.143	3.5556E-230
Rac1	1.015892	0.77	0.286	3.609E-190
Ramp2	2.320053	0.999	0.803	0
Rap1a	1.037008	0.643	0.127	7.2207E-198
Rapgef5	1.126519	0.676	0.171	1.8124E-194
Rbm39	1.415355	0.845	0.216	2.8497E-301
Rdx	1.408719	0.804	0.295	1.0454E-233
Rhoa	1.095556	0.845	0.369	7.2896E-218
Rsrp1	1.111041	0.668	0.114	3.0124E-220
S100a13	1.082648	0.709	0.2	4.5754E-198
S1pr1	2.215248	0.995	0.514	0
Sat1	1.024983	0.586	0.146	1.2715E-149
Sec62	1.126626	0.751	0.261	4.6365E-199
Selenop	1.820938	0.979	0.574	0
Sema3g	1.658577	0.891	0.286	0
Serf2	1.008718	0.74	0.266	4.0436E-182
Serinc3	2.441916	0.997	0.516	0
Sh3glb1	1.144709	0.748	0.227	5.8604E-215
Slc16a9	1.370199	0.809	0.281	1.0375E-238
Slc38a2	1.03925	0.596	0.145	1.7328E-158
Slc3a2	1.366653	0.78	0.212	1.1911E-254
Slc43a3	1.43243	0.86	0.237	8.5507E-303
Slc9a3r2	1.417706	0.86	0.272	2.6297E-286
Slco2a1	2.084607	0.973	0.442	0
Slfn5	1.185992	0.686	0.196	1.0846E-189
Smad6	1.49467	0.883	0.367	3.4234E-280
Smad7	1.512005	0.873	0.271	4.4024E-303
Socs3	1.158864	0.517	0.159	5.2084E-106
Son	1.223603	0.789	0.225	9.4757E-250
Sparcl1	1.480576	0.798	0.32	5.17E-221
Sptan1	1.021827	0.682	0.183	9.6171E-191
Sptbn1	1.955517	0.994	0.626	0
Srgn	1.075362	0.702	0.207	4.8383E-188
Srrm2	1.135458	0.735	0.198	3.4727E-217
Srsf5	1.149762	0.741	0.185	3.797E-227
Syne1	1.008164	0.598	0.136	2.7111E-163
Tagln2	1.024184	0.697	0.268	1.7255E-157
Tax1bp1	1.747688	0.838	0.209	2.8121E-303
Tcf4	1.690814	0.953	0.466	0
Tcn2	1.207187	0.718	0.149	1.5917E-235
Tek	1.660416	0.93	0.342	0
Thbd	1.280055	0.868	0.446	6.0135E-210
Timp3	1.2293	0.616	0.132	1.9279E-175
Tjp1	1.132771	0.752	0.258	6.6889E-200
Tm4sf1	1.6999	0.948	0.611	1.0897E-266
Tmbim6	1.094536	0.761	0.263	1.6577E-207
Tmem100	1.981889	0.996	0.817	0

Tmem176a	1.268965	0.782	0.212	8.4531E-247
Tmem176b	1.162302	0.749	0.247	2.6106E-203
Tmem59	1.048882	0.738	0.214	4.0555E-209
Tmsb4x	1.815612	0.998	0.891	0
Tpt1	2.256446	0.993	0.502	0
Tspan13	1.414244	0.949	0.524	0
Tspan18	1.198176	0.709	0.177	9.6895E-211
Tspan7	1.753015	0.996	0.771	0
Txnip	1.410673	0.859	0.398	9.042E-241
Uba52	1.623317	0.948	0.407	0
Ubb	2.059782	0.978	0.465	0
Ucp2	1.189392	0.665	0.106	2.649E-228
Vegfa	1.686748	0.957	0.541	0
Vim	1.013365	0.72	0.328	3.4608E-140
Vwf	1.822665	0.734	0.191	5.0433E-230
Ybx1	1.754897	0.945	0.406	0
Zbtb20	1.129604	0.721	0.254	1.0981E-179
Zfp36	1.109412	0.541	0.184	1.6592E-105
Zfp36l1	1.153635	0.706	0.22	3.7629E-192

Seurat FindMarkers TSC2KD vs WT DE (p.adj <0.05, lfc >1) M				
gene	lfc	pct1	pct2	pval.adj
4931406P1	1.224887	0.802	0.226	4.6E-213
Ace	2.174981	0.984	0.445	0
Acer2	1.094563	0.73	0.205	3.3E-174
Actb	1.371227	1	0.982	2.4E-265
Actg1	2.287107	0.981	0.548	0
Acvrl1	1.476877	0.956	0.481	7.4E-280
Adamts1	1.224105	0.6	0.307	9.43E-74
Adgre5	1.466351	0.915	0.408	5E-254
Adgrf5	2.149316	0.996	0.623	0
Adgrl4	1.829965	0.946	0.305	0
Akap12	1.055554	0.647	0.323	3.19E-79
Aldh2	1.722537	0.906	0.287	7.1E-292
App	1.059882	0.866	0.423	7E-177
Aqp1	1.969812	0.973	0.46	0
Arap2	1.03212	0.691	0.213	3.8E-147
Arhgap29	1.148572	0.78	0.252	1.3E-191
Arhgap31	1.150842	0.732	0.143	9E-209
Arhgef12	1.198292	0.811	0.308	7.8E-190
Arid5b	1.199922	0.725	0.194	7.9E-180
Atf3	2.222123	0.912	0.508	1.9E-242
Atf4	1.060962	0.74	0.287	3.3E-147
Atp1a1	1.177068	0.726	0.198	5.4E-180
AY036118	2.812122	0.999	0.756	0
B2m	1.827963	1	0.865	0
BC028528	1.291212	0.848	0.335	2.8E-206
Bmpr2	2.300893	0.998	0.666	0
Bst2	1.104928	0.709	0.158	1.4E-185
Btf3	1.001908	0.721	0.165	2.8E-185
Btg1	1.200259	0.742	0.346	1E-123
Btg2	2.448768	0.97	0.467	0
Calcrl	1.831179	0.998	0.88	0
Calm1	1.490106	0.888	0.332	3.5E-253
Casz1	1.011945	0.672	0.186	2.3E-147
Cav1	1.618042	0.987	0.654	5.7E-301
Cav2	1.192651	0.794	0.291	4.3E-191
Cavin2	1.421305	0.92	0.575	9.2E-195
Cbfa2t3	1.089429	0.698	0.174	6.3E-170
Ccnl1	1.321449	0.791	0.268	1.9E-191
Cd200	1.162047	0.782	0.349	8.6E-151
Cd24a	1.031147	0.522	0.138	1.4E-100
Cd36	1.696678	0.995	0.819	0
Cd47	1.672947	0.934	0.4	1.5E-291
Cd74	2.322857	0.791	0.213	1.7E-231
Cd9	1.551167	0.9	0.347	1.6E-257
Cd93	1.925425	0.979	0.592	0
Cdh5	1.832753	0.998	0.744	0
Cdkn1a	2.306422	0.959	0.388	0
Cebpd	2.349998	0.944	0.468	5.3E-301
Cfl1	1.25258	0.828	0.295	3.3E-210
Chchd2	1.331098	0.877	0.272	7.1E-254
Cldn5	2.520356	0.997	0.646	0
Clec14a	1.868105	0.932	0.36	3.5E-302
Clec1a	1.599225	0.875	0.331	5.1E-243
Clec2d	1.581815	0.923	0.467	5E-250

Clic1	1.234659	0.797	0.178	1.2E-228
Clic4	1.295585	0.858	0.422	2.3E-185
Clic5	1.542373	0.914	0.425	8.4E-256
Clk1	1.519878	0.857	0.213	6.1E-267
Cox8a	1.055033	0.776	0.275	3.5E-177
Crim1	1.003536	0.667	0.19	2.1E-143
Crip2	1.082859	0.762	0.242	7E-176
Csnk1a1	1.168679	0.795	0.221	6.5E-212
Csrnp1	1.026589	0.66	0.217	1.4E-129
Cst3	1.281531	0.827	0.251	1.2E-217
Ctla2a	1.929249	0.936	0.49	1.3E-264
Ctnna1	1.252828	0.822	0.24	5.8E-223
Cxcl12	1.658173	0.857	0.465	4.6E-164
Cyp4b1	1.041164	0.698	0.273	3.6E-130
Cyyr1	1.773653	0.952	0.429	0
Ddx3x	1.437318	0.844	0.24	1.7E-247
Ddx5	1.822245	0.987	0.594	0
Dlc1	1.007451	0.749	0.252	1.4E-161
Dock9	1.227897	0.761	0.163	9.1E-220
Dusp1	1.506675	0.85	0.376	2E-187
Dynll1	1.22135	0.798	0.222	4.2E-213
Ece1	1.550502	0.89	0.271	4.1E-275
Eef1a1	1.96647	0.998	0.654	0
Efnb1	1.053528	0.667	0.154	6.8E-162
Efnb2	1.624915	0.819	0.346	8.6E-192
Egfl7	1.983905	0.993	0.592	0
EGFP-REPO	2.685209	0.875	0.581	5.9E-177
Egr1	1.901387	0.81	0.661	1.1E-104
Ehd4	1.866176	0.975	0.525	0
Eif1	2.367243	0.997	0.561	0
Eif4g2	1.058604	0.766	0.235	1.6E-179
Eng	2.078142	0.977	0.412	0
Epas1	2.307398	0.999	0.799	0
Esam	1.30118	0.9	0.424	2E-232
Ets2	1.050066	0.666	0.154	1.4E-164
Fau	1.76573	0.983	0.56	0
Fermt2	1.06383	0.731	0.188	5.2E-182
Fkbp1a	1.222281	0.876	0.437	4.1E-196
Flt1	2.032624	0.966	0.369	0
Fmo1	1.693917	0.928	0.315	1.9E-299
Fmo2	1.308405	0.688	0.146	3.4E-182
Fos	1.69309	0.81	0.587	6.3E-104
Foxf1	1.430879	0.907	0.384	2.1E-252
Foxp1	1.253635	0.93	0.604	3.3E-194
Fth1	1.62087	0.918	0.337	4.2E-282
Ftl1	1.569697	0.915	0.325	1.1E-279
Fus	1.087807	0.754	0.26	6.8E-165
Fzd4	1.009569	0.673	0.191	1.9E-148
Gabarap	1.02815	0.716	0.213	1.2E-164
Gadd45g	1.383117	0.602	0.269	1.41E-89
Gata2	1.154133	0.712	0.152	4.3E-191
Gimap6	1.171411	0.72	0.213	9.1E-169
Gm42418	2.570195	1	0.994	5E-298
Gnai2	1.483522	0.945	0.467	8.8E-272
Gpihbp1	2.030032	0.954	0.41	0
Gstm1	1.085864	0.76	0.271	2.6E-165

H2-Aa	1.17927	0.644	0.145	1.8E-158
H2-Ab1	1.459371	0.707	0.162	1.5E-188
H2-D1	2.518132	1	0.68	0
H2-Eb1	1.326965	0.652	0.127	6E-174
H2-K1	2.218251	0.998	0.671	0
H2-Q4	1.393683	0.856	0.307	1.4E-228
H2-Q6	1.363679	0.821	0.255	3E-226
H2-Q7	1.93203	0.936	0.292	0
H2-T23	1.356009	0.79	0.175	3.6E-232
H3f3a	1.509782	0.927	0.331	2.4E-287
H3f3b	2.119734	0.99	0.587	0
Hes1	1.165035	0.581	0.256	6.9E-80
Hilpda	1.395486	0.665	0.182	3.5E-156
Hmgb1	1.304976	0.913	0.451	1.9E-230
Hnrnpa2b1	1.145543	0.754	0.226	2.6E-184
Hopx	1.071805	0.605	0.173	8.8E-127
Hpgd	2.522797	0.978	0.61	0
Hsp90ab1	1.356047	0.895	0.427	5.2E-215
Hspa5	1.006137	0.666	0.188	9.4E-147
Hspa8	1.470647	0.897	0.335	1.7E-250
Hspb1	1.105437	0.531	0.206	3.94E-75
Icam2	1.210087	0.848	0.291	1.3E-217
Id1	1.039132	0.539	0.11	1.1E-121
Id3	1.466163	0.781	0.227	4.2E-200
Ier2	2.004677	0.942	0.416	2.6E-301
Ier3	2.138634	0.972	0.74	1.5E-279
Ifitm2	1.412648	0.922	0.448	7.8E-243
Ifitm3	1.781424	0.998	0.84	0
Iigp1	1.283783	0.533	0.193	1.98E-81
Il6st	1.129323	0.677	0.131	4.9E-183
Irf1	1.146414	0.725	0.407	3.83E-91
Itga1	1.679994	0.942	0.456	4.2E-277
Itgb1	1.240079	0.848	0.346	3.9E-208
Itm2b	2.353254	1	0.703	0
Jun	2.273599	0.997	0.719	0
Junb	2.28328	0.995	0.802	0
Jund	2.196681	0.965	0.473	0
Jup	1.1273	0.796	0.343	7E-169
Kdr	1.139862	0.727	0.205	2.3E-174
Kitl	1.044408	0.598	0.191	3.8E-112
Klf2	2.306729	0.957	0.336	0
Klf4	1.941356	0.926	0.476	6.5E-258
Klf6	1.111689	0.621	0.237	2.6E-105
Klf7	1.429336	0.84	0.282	5.4E-226
Klf9	1.684067	0.856	0.184	1.5E-287
Klhl5	1.162411	0.756	0.237	1.1E-186
Lamp1	1.20747	0.829	0.283	3.6E-215
Laptm4a	1.137747	0.863	0.37	8E-201
Lars2	2.224362	0.983	0.351	0
Lpl	2.34869	0.864	0.137	0
Ly6a	2.515628	0.999	0.646	0
Ly6c1	2.502308	0.99	0.46	0
Ly6e	1.884582	0.991	0.648	0
Lyve1	1.192711	0.405	0.104	5.94E-71
Macf1	1.155337	0.734	0.226	9.9E-175
Malat1	2.540039	1	0.999	0

Marcks	1.196194	0.637	0.186	1.4E-138
Mast4	1.089062	0.616	0.197	1.3E-119
Mcl1	1.642262	0.876	0.234	4.4E-279
Mef2a	1.022756	0.697	0.188	1.9E-161
Morf4l1	1.038604	0.793	0.284	2.6E-176
Msn	1.307766	0.89	0.402	1.7E-220
Myl12a	1.172587	0.868	0.406	9.7E-197
Myl12b	1.135241	0.835	0.314	6.2E-201
Myl6	1.268189	0.898	0.441	3.1E-211
Myzap	1.337522	0.801	0.217	2.9E-221
Nfib	1.614403	0.936	0.395	6.2E-287
Nfkbia	1.306193	0.748	0.299	2.4E-157
Notch1	1.034513	0.687	0.154	1.7E-171
Nrp1	1.406422	0.856	0.274	9.7E-240
Oaz1	1.23149	0.797	0.167	5.4E-237
Pcdh17	1.149558	0.716	0.279	1.8E-140
Pde4b	1.367531	0.756	0.179	3.7E-206
Pecam1	1.861318	0.985	0.493	0
Pim3	1.012805	0.569	0.182	4.9E-103
Pitpnc1	1.18683	0.784	0.332	1.2E-161
Pltp	1.458466	0.93	0.563	6.7E-229
Plvap	1.408156	0.689	0.299	1.5E-124
Pnrc1	1.120053	0.64	0.154	1.7E-152
Podxl	1.199241	0.805	0.301	1.4E-189
Ppia	1.669067	0.965	0.482	0
Ppp1r15a	1.408044	0.74	0.279	7.2E-160
Ppp1r2	1.244934	0.731	0.213	7.2E-179
Ptma	1.71305	0.997	0.782	0
Ptprb	2.397324	0.998	0.685	0
Qk	1.278555	0.88	0.376	4.6E-219
Rabac1	1.003997	0.711	0.137	8.8E-194
Rac1	1.043757	0.8	0.313	1.2E-174
Ramp2	2.306207	0.993	0.64	0
Rap1a	1.220701	0.734	0.13	9.8E-218
Rapgef5	1.012146	0.658	0.162	8.9E-155
Rasip1	1.00969	0.697	0.204	1.3E-154
Rassf1	1.076775	0.66	0.114	3.6E-182
Rbm39	1.4942	0.882	0.243	3.6E-278
Rdx	1.572904	0.877	0.346	8.2E-237
Rhoa	1.145057	0.878	0.399	4.5E-207
Rhob	1.012357	0.707	0.334	1.45E-98
S100a13	1.056042	0.724	0.204	2.3E-172
S100a16	1.023554	0.719	0.214	4E-165
S1pr1	2.221269	0.98	0.468	0
Sat1	1.028168	0.602	0.2	3.6E-108
Sbno2	1.227923	0.682	0.136	5.8E-185
Sec62	1.029579	0.73	0.24	4.1E-158
Selenok	1.00203	0.744	0.196	1.7E-184
Selenop	1.652044	0.919	0.389	2.7E-263
Sema3c	1.273731	0.767	0.575	6.74E-83
Sema3g	1.305454	0.74	0.194	2.8E-193
Serf2	1.006005	0.765	0.282	1.3E-161
Serinc3	2.287468	0.991	0.423	0
Sertad1	1.077272	0.669	0.143	5.3E-168
Sh3glb1	1.180847	0.767	0.198	2.8E-204
Slc16a9	1.009526	0.596	0.131	1.4E-137

Slc38a2	1.33576	0.783	0.255	1.5E-190
Slc3a2	1.373608	0.794	0.211	7.2E-221
Slc43a3	1.425551	0.856	0.205	8.8E-268
Slc9a3r2	1.511466	0.87	0.237	9.7E-271
Slco2a1	1.968334	0.969	0.404	0
Slfn5	1.759994	0.827	0.299	1.1E-218
Smad6	1.363889	0.78	0.229	2.5E-204
Smad7	1.518636	0.798	0.198	7.3E-235
Snrk	1.008154	0.67	0.15	1.6E-164
Socs3	1.747236	0.915	0.527	5.9E-232
Son	1.322688	0.831	0.208	4E-246
Sparcl1	1.336784	0.736	0.283	2E-154
Sptbn1	1.945877	0.99	0.599	0
Srgn	1.114996	0.722	0.201	2.3E-172
Srrm2	1.156599	0.751	0.19	2.8E-197
Srsf2	1.082869	0.697	0.202	3.2E-159
Srsf5	1.497587	0.887	0.244	8E-278
Tagln2	1.055842	0.75	0.283	2.7E-152
Tax1bp1	1.477401	0.76	0.166	6.5E-219
Tcf4	1.673806	0.958	0.479	2.4E-284
Tcn2	1.141991	0.711	0.151	1.9E-189
Tek	1.396878	0.878	0.287	2.1E-255
Thbd	1.414909	0.873	0.462	1.5E-191
Timp3	1.669402	0.693	0.201	1.2E-159
Tjp1	1.101341	0.776	0.262	8E-178
Tm4sf1	1.580787	0.906	0.475	1.2E-217
Tmbim6	1.001987	0.733	0.226	3.6E-168
Tmem100	2.006356	0.98	0.681	0
Tmem176a	1.083627	0.701	0.174	9.2E-174
Tmem176b	1.024074	0.69	0.19	2.1E-155
Tmsb4x	1.795542	0.998	0.866	0
Tpm3	1.37548	0.836	0.244	4.2E-241
Tpt1	2.32043	0.998	0.609	0
Tra2b	1.200837	0.72	0.213	5.8E-165
Tspan13	1.332148	0.896	0.396	5.1E-234
Tspan18	1.172366	0.698	0.173	1.1E-174
Tspan7	1.613641	0.992	0.716	0
Txnip	1.092754	0.714	0.269	3.7E-139
Uba52	1.514187	0.928	0.439	6.8E-260
Ubb	2.144104	0.995	0.553	0
Ubc	1.338104	0.828	0.307	6.1E-210
Ube2d3	1.18369	0.772	0.23	7.9E-193
Ucp2	1.255028	0.716	0.146	3.4E-199
Vegfa	1.623126	0.904	0.416	4.8E-246
Vim	1.021519	0.763	0.368	5.4E-125
Vwf	1.581835	0.572	0.109	1.1E-140
Xdh	1.005175	0.621	0.075	4.1E-181
Ybx1	1.847515	0.945	0.403	0
Zbtb20	1.178521	0.738	0.243	3.6E-163
Zfp36	2.001057	0.969	0.632	8.8E-290
Zfp36l1	1.284109	0.783	0.287	1.6E-178

Seurat FindMarkers TSC2KD vs WT DE (p.adj <0.05, lfc >1) M				
gene	lfc	pct1	pct2	pval.adj
4931406P1	1.105953	0.764	0.187	9.9E-145
Abcb1a	1.006993	0.714	0.218	1.6E-111
Abi3bp	1.405823	0.814	0.41	2.2E-121
Ace	2.396892	0.998	0.656	3.7E-284
Acer2	1.367306	0.852	0.265	1.2E-176
Actb	1.183261	0.997	0.956	3.8E-153
Actg1	2.050544	0.969	0.554	2E-217
Acvrl1	1.49366	0.956	0.481	1.2E-201
Adgre5	1.316238	0.869	0.448	6E-139
Adgrf5	2.127406	0.984	0.553	1.4E-251
Adgrg6	1.311993	0.732	0.299	1.6E-108
Adgrl4	1.742358	0.922	0.279	6.2E-222
Ahnak	1.182332	0.805	0.396	5E-109
Akap12	1.05402	0.674	0.245	5.53E-88
Aldh2	1.933582	0.973	0.396	3.9E-252
Aldoa	1.113192	0.722	0.135	2E-143
Aplp2	1.167129	0.827	0.312	9.6E-141
App	1.248233	0.911	0.486	3.6E-162
Aqp1	1.910593	0.927	0.548	2.4E-185
Arhgap29	1.175854	0.78	0.208	7.5E-149
Arhgef12	1.029572	0.754	0.259	3.4E-119
Atp13a3	2.300518	0.797	0.328	3.2E-139
Atp1b3	1.444894	0.782	0.199	1.7E-157
Atp2b1	1.031583	0.676	0.2	4.5E-103
Atrx	1.191132	0.734	0.196	1.3E-132
AY036118	2.929151	1	0.851	6.9E-285
B2m	1.590857	0.99	0.751	9.4E-211
Bmpr2	2.314002	0.995	0.702	4.2E-272
Bsg	1.276246	0.743	0.192	3.8E-130
Bst2	1.152146	0.743	0.166	2.4E-143
Btg2	1.785	0.785	0.173	2.1E-167
Calcrl	1.934801	0.994	0.893	5.4E-199
Calm1	1.538662	0.917	0.357	2.2E-201
Cav1	1.673327	0.977	0.59	1.4E-221
Cav2	1.231007	0.83	0.288	4.4E-154
Cavin1	1.140308	0.797	0.358	1E-111
Cavin2	1.492407	0.961	0.593	3.5E-182
Cd200	1.249837	0.839	0.38	6.9E-136
Cd24a	1.192771	0.694	0.167	4.1E-123
Cd2ap	1.014826	0.649	0.157	3.1E-108
Cd36	1.858062	0.915	0.768	3.6E-153
Cd47	1.342298	0.888	0.354	1.1E-169
Cd9	1.558098	0.888	0.333	3.7E-182
Cd93	1.587723	0.924	0.453	1.3E-185
Cdc42	1.014061	0.733	0.208	4.9E-126
Cdh5	1.753384	0.989	0.686	3.3E-235
Cdkn1a	1.482446	0.693	0.118	2.6E-141
Cebpd	1.469118	0.653	0.139	3.6E-117
Chchd2	1.27404	0.868	0.265	3E-180
Cldn5	2.387332	0.995	0.569	3.6E-274
Clec14a	1.866053	0.977	0.503	9.1E-235
Clec1a	1.134992	0.697	0.197	8.6E-114
Clec2d	1.309083	0.872	0.456	6.7E-137
Clic1	1.052147	0.735	0.163	6.8E-140

Clic4	1.120706	0.821	0.355	1E-121
Clic5	1.22798	0.748	0.289	3.7E-111
Clk1	1.048857	0.69	0.165	2.1E-119
Cox4i1	1.057435	0.77	0.214	1.1E-139
Cox8a	1.012021	0.768	0.254	1.2E-126
Crim1	1.375174	0.834	0.299	1.8E-156
Crip1	1.308819	0.84	0.496	2.1E-107
Crip2	1.255625	0.877	0.366	5.5E-163
Csnk1a1	1.111509	0.777	0.205	7E-146
Cst3	1.753595	0.93	0.473	4.2E-176
Ctla2a	1.781017	0.831	0.308	2.6E-158
Ctnna1	1.112725	0.796	0.243	8.2E-144
Cxcl12	1.861337	0.959	0.9	6.3E-120
Cyyr1	1.455465	0.886	0.37	2E-173
Ddx3x	1.174822	0.761	0.217	4.3E-137
Ddx5	1.641932	0.98	0.526	4.3E-229
Dock9	1.356749	0.822	0.184	9.4E-179
Dst	1.631626	0.812	0.238	4.9E-164
Dusp1	1.571029	0.818	0.313	9.8E-144
Dynll1	1.120755	0.777	0.213	1.8E-141
Ece1	1.667008	0.96	0.391	1.9E-228
Edn1	1.361916	0.79	0.621	2.66E-54
Eef1a1	2.223517	0.997	0.656	1.7E-263
Eef2	1.125786	0.753	0.198	4.8E-139
Efnb2	1.815714	0.973	0.604	1.4E-225
Egfl7	2.004094	0.997	0.608	3.5E-260
EGFP-REPO	2.422312	0.833	0.592	2.1E-115
Egr1	1.604697	0.529	0.157	9.55E-69
Ehd4	1.590748	0.927	0.447	3E-188
Eif1	1.91505	0.976	0.391	3.6E-255
Eif4g2	1.004552	0.744	0.229	2.1E-122
Eng	2.125857	0.99	0.501	1.6E-260
Epas1	2.165228	0.999	0.78	9.7E-277
Esam	1.269417	0.887	0.359	1.4E-165
Fau	2.014929	0.988	0.569	5.2E-244
Fkbp1a	1.198356	0.882	0.437	1.5E-144
Flt1	2.101643	0.985	0.471	9.9E-258
Fmo1	1.592201	0.895	0.345	1.3E-185
Fmo2	1.981544	0.952	0.385	5.2E-234
Fos	1.325352	0.534	0.238	5.44E-45
Foxp1	1.115085	0.903	0.577	3.6E-122
Fth1	1.551267	0.927	0.374	5.5E-194
Ftl1	1.404362	0.849	0.246	6.8E-177
Fxyd5	1.217767	0.776	0.218	2.7E-143
Gm42418	2.740535	1	0.998	2.6E-259
Gnai2	1.426674	0.943	0.416	1.4E-195
Gpihbp1	1.767243	0.753	0.273	4.4E-129
Gstm1	1.08591	0.793	0.291	1.6E-124
H2-D1	2.119458	0.984	0.601	7.4E-259
H2-K1	1.744242	0.956	0.516	1.2E-192
H2-Q7	1.13087	0.638	0.168	1.6E-102
H3f3a	1.369635	0.907	0.303	2.4E-198
H3f3b	1.707101	0.951	0.396	1.1E-213
Heg1	1.039554	0.799	0.377	1.4E-102
Hilpda	1.680105	0.844	0.33	1.3E-161
Hmgb1	1.316877	0.924	0.446	6.7E-176

Hnrnpa2b1	1.06353	0.751	0.216	1.7E-129
Hpgd	2.032739	0.919	0.578	1.1E-155
Hsp90ab1	1.447261	0.914	0.437	2.4E-179
Hsp90b1	1.333569	0.86	0.314	1.3E-164
Hspa5	1.027748	0.66	0.152	5.5E-113
Hspa8	1.422189	0.883	0.241	1.2E-198
Icam2	1.342452	0.888	0.337	6.4E-173
Id1	1.412915	0.771	0.226	6.9E-144
Id3	1.773513	0.926	0.392	1.3E-202
Ier2	1.203008	0.677	0.192	4.3E-107
Ier3	1.297959	0.587	0.226	4.1E-65
Ifitm2	1.635128	0.976	0.568	4.3E-225
Ifitm3	1.742605	0.998	0.813	2E-235
Igfbp4	1.018486	0.674	0.241	5.5E-88
Il6st	1.036341	0.666	0.119	1.4E-128
Itga1	1.453048	0.874	0.35	8.5E-164
Itga6	1.181728	0.733	0.192	2.7E-132
Itgb1	1.29758	0.898	0.34	2.9E-175
Itm2b	2.025762	0.997	0.692	1.3E-269
Jun	1.986227	0.949	0.548	1.1E-183
Junb	1.666999	0.724	0.331	3.01E-88
Jund	1.465039	0.721	0.193	1.2E-127
Klf2	2.121537	0.945	0.395	6.2E-226
Klf4	1.528038	0.803	0.359	3.3E-123
Klf7	1.203088	0.776	0.225	3.8E-143
Klf9	1.292592	0.749	0.176	1.1E-147
Klhl5	1.022586	0.67	0.191	2.7E-106
Lamp1	1.17461	0.838	0.28	6.2E-155
Laptm4a	1.076544	0.886	0.427	5.7E-138
Lars2	2.472247	0.996	0.44	1.7E-283
Lpl	2.03128	0.7	0.14	1.6E-145
Lrrc58	1.039159	0.713	0.223	3.4E-112
Ltbp4	1.174023	0.801	0.441	2.17E-90
Ly6a	2.387737	0.998	0.721	2.5E-272
Ly6c1	2.468194	0.995	0.618	2.5E-269
Ly6e	1.863645	0.99	0.688	6.3E-238
Lyve1	2.828815	0.776	0.364	2.3E-122
Macf1	1.208583	0.806	0.274	6.5E-143
Malat1	2.67257	0.999	0.998	1.2E-274
Mcam	1.351615	0.85	0.264	8E-171
Mcl1	1.231541	0.729	0.109	5.8E-159
Mctp1	1.100161	0.629	0.109	3.2E-120
Mgll	1.652402	0.873	0.255	1E-197
Morf4l1	1.046251	0.817	0.292	7E-134
Msn	1.237993	0.878	0.389	5.7E-153
Myl12a	1.061479	0.837	0.356	8.8E-133
Myl12b	1.084995	0.818	0.342	1.6E-132
Myl6	1.197603	0.903	0.44	4.8E-156
Naca	1.072208	0.73	0.168	2.8E-136
Nedd4	1.050532	0.767	0.255	4.3E-125
Nfib	1.532303	0.912	0.366	3.2E-194
Nfkbia	1.244975	0.74	0.196	1.4E-134
Notch1	1.389506	0.853	0.271	1.9E-170
Nrp1	1.476507	0.896	0.35	1.5E-179
Ntn4	1.034103	0.629	0.192	1.15E-86
Oaz1	1.05375	0.731	0.162	1E-139

Pde3a	1.204005	0.793	0.289	1.4E-129
Pde4b	1.274574	0.728	0.162	6.9E-141
Pecam1	1.977903	0.986	0.535	4.1E-255
Plat	1.719795	0.772	0.201	3.2E-153
Pltp	1.305835	0.852	0.546	1.1E-107
Podxl	1.115104	0.828	0.385	1.4E-122
Ppia	1.745153	0.979	0.464	2.3E-240
Prrc2c	1.016291	0.731	0.222	7.8E-119
Psen2	1.04616	0.466	0.058	4.77E-85
Ptma	1.638943	0.996	0.77	3.8E-226
Ptprb	2.495704	1	0.843	5.9E-290
Qk	1.095636	0.816	0.341	2.2E-126
Rabac1	1.100462	0.751	0.163	3.6E-150
Rac1	1.087674	0.811	0.322	6.9E-132
Rack1	1.080543	0.738	0.183	1.6E-133
Ramp2	1.985559	0.981	0.687	8.5E-236
Rapgef5	1.096347	0.73	0.204	4.3E-127
Rbm39	1.229794	0.82	0.229	6.6E-164
Rdx	1.272081	0.807	0.272	3.7E-145
Rhoa	1.151301	0.885	0.399	3E-153
Rhob	1.249554	0.741	0.325	3.24E-95
Rnf144a	1.178209	0.853	0.425	1.2E-129
Rrbp1	1.096263	0.714	0.191	1.9E-120
S100a13	1.005442	0.723	0.243	5.3E-111
S1pr1	2.012979	0.973	0.4	5.5E-249
Sat1	1.349154	0.697	0.184	3.3E-120
Sec62	1.007691	0.746	0.222	7.9E-122
Selenop	1.797655	0.914	0.423	4.3E-179
Sema3g	1.657242	0.937	0.414	5.2E-206
Serbp1	1.013451	0.761	0.242	3.8E-124
Serf2	1.127148	0.814	0.287	3.6E-137
Serinc3	2.095786	0.986	0.44	2.3E-258
Sh3glb1	1.094467	0.78	0.232	8.5E-140
Slc38a2	1.106054	0.627	0.148	1.6E-104
Slc3a2	1.200649	0.703	0.166	2.5E-129
Slc43a3	1.188839	0.783	0.208	2.1E-147
Slc6a6	1.31223	0.606	0.342	5.6E-51
Slc9a3r2	1.518324	0.895	0.304	1.1E-193
Slco2a1	1.404077	0.746	0.281	1E-116
Slfn5	1.250694	0.728	0.188	1.2E-131
Smad6	1.596521	0.944	0.462	1.8E-203
Smad7	1.620147	0.909	0.275	5.1E-211
Socs3	1.004779	0.468	0.177	2.39E-44
Son	1.270313	0.816	0.192	3E-171
Sox18	1.069991	0.782	0.3	4E-116
Sparcl1	1.212865	0.664	0.235	7.67E-92
Sptbn1	1.928899	0.993	0.615	2.3E-240
Srgn	1.241635	0.845	0.275	5.2E-162
Srrm2	1.146921	0.786	0.203	2.2E-151
Srsf5	1.099574	0.73	0.167	3.1E-140
Sulf1	1.133958	0.537	0.147	7.38E-75
Syne1	1.290226	0.728	0.139	4.2E-150
Tagln2	1.171848	0.805	0.299	9.7E-133
Tax1bp1	1.531433	0.8	0.176	2.6E-171
Tcf4	1.483768	0.912	0.44	1.2E-180
Tek	1.425421	0.911	0.324	6.3E-190

Thbd	1.023635	0.684	0.311	4.64E-74
Timp3	1.963225	0.891	0.32	1.6E-193
Tinagl1	1.249959	0.653	0.172	3.4E-109
Tjp1	1.049758	0.774	0.251	3.5E-128
Tm4sf1	1.956499	0.991	0.776	2.8E-247
Tmbim6	1.076629	0.793	0.257	1.1E-136
Tmem100	2.004336	0.967	0.695	4.9E-213
Tmem2	1.436739	0.92	0.495	4.9E-167
Tmod3	1.023094	0.678	0.131	1.7E-129
Tmsb4x	1.875988	1	0.87	8E-256
Tns1	1.041355	0.689	0.138	1.7E-131
Tpt1	2.52814	0.993	0.58	2.3E-277
Tsc22d1	1.349877	0.671	0.157	1.9E-119
Tspan13	1.290159	0.869	0.342	2.6E-157
Tspan7	1.377001	0.921	0.571	1.5E-148
Tspo	1.081571	0.738	0.192	9.7E-131
Txnip	1.29117	0.843	0.413	1.5E-124
Uba52	1.83449	0.966	0.458	6.6E-232
Ubb	2.002313	0.986	0.413	4E-264
Utrn	1.508888	0.811	0.251	8.9E-159
Vegfa	1.545637	0.937	0.569	8.5E-175
Vim	1.065451	0.757	0.386	4.32E-88
Vwf	2.685771	0.979	0.557	2.3E-252
Wwp1	1.026289	0.468	0.073	1.14E-77
Ybx1	1.715024	0.935	0.358	1.4E-218
Zbtb20	1.115286	0.745	0.319	1.9E-103
Zfp36	1.1806	0.565	0.243	3.17E-54
Zfp36l1	1.240882	0.8	0.265	4.7E-141

Seurat FindMarkers TSC2KD vs WT DE (p.adj <0.05, lfc >1) M				
gene	lfc	pct1	pct2	pval.adj
4931406P1	1.139361	0.836	0.303	1.97E-93
Ace	2.134918	0.979	0.445	1E-152
Actb	1.206015	1	0.994	2.6E-119
Actg1	1.906367	0.981	0.643	3E-136
Acvrl1	1.495686	0.987	0.667	5.8E-139
Add3	1.071886	0.789	0.253	2.82E-85
Adgre5	1.330676	0.909	0.373	6.5E-114
Adgrf5	2.131566	0.996	0.728	4.2E-163
Adgrl2	1.002518	0.819	0.415	1.27E-69
Adgrl4	1.045188	0.684	0.197	6.58E-69
Afap1l1	1.015245	0.779	0.215	3.69E-87
Aldh2	1.211198	0.804	0.22	3E-95
Anxa3	1.299459	0.905	0.365	3.7E-112
App	1.469092	0.996	0.791	8.8E-134
Aqp1	1.785744	0.966	0.398	4.5E-147
Arap2	1.311475	0.876	0.297	6.4E-109
Arhgap29	1.167864	0.848	0.288	3.57E-97
Arhgef12	1.3438	0.909	0.415	8.8E-107
Arid5b	1.004859	0.731	0.219	2.74E-74
Arpc2	1.089443	0.882	0.368	1.55E-93
Atf3	1.589155	0.617	0.248	3.4E-46
Atp8a1	1.125679	0.863	0.354	3.71E-90
AW112010	1.293152	0.846	0.436	1.33E-81
AY036118	3.025551	0.994	0.821	2E-166
B2m	1.966675	0.998	0.885	8.1E-165
BC028528	1.082947	0.766	0.247	3.89E-81
Bcam	1.206848	0.886	0.363	8.4E-100
Bmpr2	2.17259	0.992	0.701	3.6E-160
Btf3	1.025673	0.787	0.173	5.5E-100
Btg2	1.921026	0.8	0.211	8.3E-106
Calcrl	1.866059	0.998	0.925	7.2E-156
Calm1	1.746224	0.992	0.503	1.1E-148
Car4	2.038894	0.998	0.758	6.2E-160
Cav1	1.53938	0.977	0.542	1E-134
Cav2	1.311434	0.92	0.379	6.3E-116
Cavin2	1.840605	0.989	0.775	6.6E-149
Cbfa2t3	1.114712	0.8	0.27	4.61E-85
Cd36	1.635212	0.994	0.876	1.7E-143
Cd47	2.15558	0.994	0.709	2.9E-162
Cd9	1.530621	0.952	0.462	8.4E-128
Cdc42	1.123792	0.874	0.341	1.5E-100
Cdh5	1.848777	1	0.851	7.6E-162
Cdkn1a	2.265956	0.966	0.398	8.3E-154
Cebpd	1.592563	0.739	0.129	3.2E-102
Cfl1	1.223091	0.903	0.374	5.3E-107
Chchd2	1.330334	0.928	0.313	2.4E-126
Cldn5	2.493574	1	0.846	7.6E-171
Clec14a	1.297337	0.819	0.244	6.4E-100
Clec1a	1.530874	0.943	0.398	2.6E-132
Clec2d	1.424836	0.899	0.398	2.9E-104
Clic1	1.266106	0.844	0.241	1.2E-107
Clic4	1.604824	0.985	0.621	6.5E-138
Clic5	1.990226	1	0.662	3E-167
Clk1	1.065852	0.718	0.201	2.21E-77

Clu	1.312106	0.859	0.407	1.2E-87
Cox8a	1.027515	0.859	0.34	4.43E-88
Crip1	1.637404	0.893	0.626	3.08E-81
Crip2	1.418997	0.966	0.47	6.4E-125
Csnk1a1	1.221544	0.872	0.319	1.6E-105
Cst3	1.579012	0.947	0.387	1.4E-132
Ctla2a	1.956815	0.973	0.553	5.7E-135
Ctnna1	1.329551	0.924	0.299	1.4E-122
Ctsl	1.16483	0.825	0.285	1.36E-93
Cyp4b1	1.661854	0.979	0.649	1.7E-130
Cyth3	1.265504	0.899	0.431	4.8E-101
Cyyr1	1.276108	0.829	0.255	4.1E-100
Ddx5	1.619069	0.992	0.586	1.6E-148
Dpysl2	1.051978	0.912	0.527	1.1E-83
Dstn	1.041658	0.84	0.285	6.86E-89
Dusp1	1.418062	0.842	0.374	3.13E-84
Dynll1	1.287082	0.886	0.387	1.4E-100
Ece1	1.063003	0.777	0.259	2.25E-78
Ecscr	1.252512	0.893	0.385	4.7E-102
Ednrb	1.959382	0.931	0.58	1.9E-121
Eef1a1	1.881361	1	0.698	1.1E-162
Efnb2	1.127482	0.676	0.231	1.26E-58
Egfl7	1.70818	0.994	0.748	2.2E-150
EGFP-REPO	1.498017	0.566	0.209	7.07E-38
Egr1	1.860456	0.674	0.491	2.73E-34
Ehd4	1.99398	1	0.664	1.7E-165
Eif1	2.117006	0.998	0.605	7E-166
Eif4g2	1.004325	0.792	0.281	1.43E-80
Emp1	1.059579	0.737	0.209	4.71E-79
Emp2	2.099525	0.998	0.915	7.8E-162
Eng	1.636888	0.983	0.445	9.2E-145
Epas1	2.421314	1	0.786	2.2E-171
Esam	1.365958	0.958	0.465	1.6E-120
Fau	1.661124	0.989	0.553	2.6E-142
Fermt2	1.076504	0.838	0.322	9.77E-89
Fibin	1.715983	0.768	0.247	3.46E-87
Fkbp1a	1.398528	0.992	0.682	1.8E-124
Flt1	1.422694	0.869	0.349	2.9E-102
Fmo1	1.973996	0.996	0.538	2.8E-162
Fos	1.792137	0.646	0.347	8.65E-36
Foxf1	1.227841	0.939	0.443	1E-106
Fth1	1.895532	0.985	0.437	4.9E-153
Ftl1	1.62662	0.958	0.415	5.8E-137
Gadd45g	1.21296	0.556	0.189	3.44E-41
Gata2	1.119382	0.813	0.274	6.41E-91
Gm42418	2.659306	1	0.997	3.7E-152
Gnai2	1.708157	0.987	0.601	2.5E-151
Gnb1	1.020376	0.874	0.388	1.54E-85
Gnb2	1.130303	0.829	0.247	2.9E-98
Gng5	1.0181	0.857	0.357	1.43E-82
Gngt2	1.12208	0.802	0.242	3.52E-88
Gpihbp1	1.013989	0.476	0.129	7.87E-39
Gpr146	1.063666	0.749	0.186	2.29E-87
Gstm1	1.016852	0.819	0.335	5.16E-79
H2-D1	2.59882	0.998	0.676	5.9E-172
H2-K1	2.130438	0.985	0.524	1.2E-150

H2-Q4	1.08879	0.783	0.236	6.45E-86
H2-Q7	1.384723	0.829	0.16	2.1E-120
H2-T23	1.212868	0.804	0.231	6.86E-95
H3f3a	1.622999	0.979	0.399	1.3E-151
H3f3b	1.656119	0.949	0.528	1.3E-124
Hes1	1.046019	0.638	0.241	5.76E-49
Hilpda	1.124574	0.661	0.179	1.39E-66
Hint1	1.090331	0.813	0.237	1.64E-95
Hmgb1	1.309081	0.935	0.472	6.2E-110
Hopx	2.209567	0.994	0.693	1.5E-164
Hpgd	2.056618	0.979	0.535	3.4E-149
Hsp90ab1	1.530879	0.975	0.539	9.6E-128
Hsp90b1	1.152668	0.846	0.281	3.25E-94
Hspa8	1.539972	0.952	0.36	4.8E-137
Hspb1	1.779666	0.952	0.59	8.9E-109
Icam2	1.657788	0.979	0.623	2.5E-132
Id1	1.262744	0.722	0.203	1.18E-77
Id3	1.681681	0.916	0.365	1.6E-119
Ier2	1.935826	0.853	0.377	9.8E-99
Ier3	2.067516	0.867	0.467	1.03E-88
Ifitm2	1.200925	0.905	0.417	2.36E-96
Ifitm3	1.313024	0.829	0.41	1.96E-68
Igfbp7	2.353076	0.998	0.879	6.2E-165
Itgb1	1.089203	0.876	0.392	3.12E-88
Itm2b	2.604562	1	0.789	2E-175
Jun	2.375025	0.998	0.756	2E-162
Junb	2.138518	0.886	0.569	3.59E-86
Jund	2.259439	0.956	0.371	2.7E-148
Jup	1.204607	0.895	0.513	8.52E-89
Kctd12	1.014859	0.768	0.305	3.67E-67
Kdr	1.893395	0.989	0.764	9.6E-152
Kitl	1.808729	1	0.717	9.7E-147
Klf13	1.043536	0.771	0.28	1.75E-77
Klf2	2.130921	0.952	0.428	3.3E-141
Klf4	1.929255	0.956	0.574	2.1E-126
Klf7	1.57475	0.962	0.484	3.9E-127
Klf9	1.126486	0.716	0.154	3.23E-87
Krt80	1.26039	0.876	0.314	7.3E-105
Lamp1	1.373867	0.954	0.436	4.1E-128
Laptm4a	1.216475	0.952	0.45	6.1E-116
Lars2	2.286254	0.987	0.443	7.2E-167
Ly6a	2.791895	1	0.895	6.1E-175
Ly6c1	2.851695	1	0.807	8.5E-174
Ly6e	2.171804	1	0.775	1E-168
Macf1	1.062601	0.785	0.27	8.76E-81
Malat1	2.531983	0.998	0.987	3.6E-154
Map1lc3b	1.033144	0.752	0.204	4.41E-85
Marcks	1.707538	0.916	0.406	4.9E-120
Mcl1	1.279401	0.785	0.208	6.28E-98
Mef2a	1.093629	0.775	0.253	6.04E-83
Mgll	1.357268	0.808	0.322	5.5E-85
Morf4l1	1.034076	0.846	0.347	1.32E-83
Msn	1.271146	0.949	0.544	5.9E-109
Mxd4	1.311745	0.832	0.201	2.1E-113
Myl12a	1.116607	0.941	0.566	3.29E-94
Myl12b	1.134976	0.888	0.421	1.84E-90

Myl6	1.475503	0.981	0.615	3.6E-128
Myzap	1.477341	0.931	0.341	2.3E-128
Nfib	1.371835	0.947	0.423	6.2E-119
Nfkbia	1.073846	0.752	0.206	1.7E-83
Nkd1	1.256014	0.893	0.288	4.1E-117
Nrp1	1.565226	0.983	0.646	4.1E-130
Oaz1	1.11426	0.796	0.171	4.8E-105
Pcdh1	1.296145	0.891	0.351	3.3E-107
Pdgfb	1.006482	0.844	0.544	1.58E-55
Pecam1	1.896729	0.998	0.616	5.3E-163
Pfn1	1.10093	0.886	0.387	1.45E-95
Phlda3	1.030352	0.659	0.168	2.23E-68
Pitpnc1	1.199825	0.861	0.412	1.4E-88
Plec	1.012785	0.731	0.215	6.88E-76
Pltp	1.100654	0.76	0.263	1.91E-71
Pmp22	1.738416	0.96	0.542	2.6E-138
Podxl	1.21931	0.867	0.373	2.48E-95
Ppia	1.701278	0.989	0.582	5.2E-153
Ppp2r5a	1.233314	0.821	0.184	7.6E-111
Prx	1.286902	0.861	0.289	2E-104
Ptma	1.826422	1	0.866	5.1E-157
Ptp4a3	1.518725	0.931	0.368	5.1E-125
Ptprb	1.290939	0.556	0.223	1.26E-36
Qk	1.146026	0.937	0.517	1.77E-98
Rabac1	1.093304	0.808	0.195	2.5E-101
Rac1	1.231463	0.926	0.404	3E-110
Ramp2	2.192808	1	0.851	4.8E-168
Rap1a	1.227932	0.836	0.215	2E-108
Rap1b	1.040366	0.821	0.305	9.11E-87
Rapgef5	1.128458	0.775	0.19	1.37E-93
Rbm39	1.19587	0.863	0.236	1.1E-110
Rbms1	1.171386	0.834	0.333	2.61E-89
Rdx	1.268563	0.863	0.343	9.6E-94
Rgs12	1.291421	0.84	0.305	1.64E-98
Rhoa	1.29121	0.975	0.536	5.7E-125
Rock2	1.059602	0.766	0.308	9.21E-72
Rtl8a	1.031204	0.853	0.429	9.35E-75
S100a13	1.189696	0.821	0.241	5.5E-99
S1pr1	2.050154	0.994	0.577	2.6E-165
Scn7a	1.214891	0.928	0.675	1.38E-79
Sec62	1.150345	0.855	0.294	3.18E-95
Selenok	1.137457	0.848	0.278	1.1E-99
Selenop	1.930061	0.998	0.731	9.5E-157
Sema3f	1.19536	0.851	0.314	2.47E-98
Sema6a	1.135248	0.836	0.288	1.44E-91
Sema7a	1.048807	0.739	0.204	5.66E-79
Serf2	1.031795	0.815	0.288	1.03E-85
Serinc3	2.441377	0.998	0.627	2.2E-173
Sh3glb1	1.042498	0.787	0.236	1.37E-86
Slc43a3	1.293014	0.838	0.195	5.6E-117
Slc9a3r2	1.910234	0.996	0.465	1.6E-163
Slco2a1	1.771053	0.964	0.401	5.4E-142
Slfn5	1.467616	0.865	0.325	1.9E-101
Smad6	1.080621	0.798	0.302	2.16E-80
Smad7	1.098946	0.762	0.236	1.43E-81
Smarca2	1.111312	0.859	0.432	4.05E-77

Snrk	1.24775	0.876	0.321	1.1E-101
Socs3	1.058396	0.632	0.184	5.03E-58
Son	1.087784	0.804	0.225	9.48E-94
Sptan1	1.026873	0.752	0.214	2.02E-83
Sptbn1	2.069256	0.998	0.7	8.7E-156
Srgn	1.059159	0.76	0.252	7.42E-76
Srsf5	1.102438	0.792	0.206	1.2E-96
Stmn2	1.35071	0.924	0.511	2.3E-105
Tagln2	1.135266	0.905	0.483	2.96E-87
Tbx2	1.063379	0.789	0.244	2.45E-86
Tbx3	1.693279	0.964	0.465	5.2E-140
Tcf4	1.496274	0.956	0.509	3.6E-125
Tcn2	1.386197	0.838	0.203	1.2E-114
Thbd	1.485176	0.958	0.572	1.7E-112
Timp2	1.236188	0.914	0.451	3.9E-101
Timp3	1.803504	0.918	0.536	1.69E-96
Tjp1	1.174207	0.872	0.322	1.4E-99
Tmbim6	1.14876	0.844	0.281	8.9E-100
Tmem100	1.874322	0.996	0.789	2.5E-147
Tmem176a	1.393024	0.905	0.371	2.7E-110
Tmem176b	1.230669	0.876	0.366	4.22E-95
Tmem204	1.169373	0.861	0.373	7.4E-95
Tmsb4x	1.888981	0.989	0.818	3.9E-141
Tpm3	1.14035	0.838	0.234	1.9E-101
Tpt1	2.222627	0.998	0.53	7.2E-172
Tspan13	1.578848	0.987	0.723	6.1E-138
Tspan18	1.587194	0.931	0.36	1.8E-130
Tspan7	1.296507	0.937	0.615	4.84E-91
Tspo	1.379305	0.895	0.274	5.5E-122
Tuba1a	1.445988	0.884	0.311	8.7E-117
Txn1	1.239965	0.88	0.302	1.7E-108
Txnip	1.237614	0.895	0.377	4.49E-95
Uba52	1.42854	0.945	0.406	4.8E-122
Ubb	2.301546	1	0.642	2.4E-173
Ubc	1.155024	0.802	0.283	6.82E-82
Ucp2	1.156424	0.756	0.101	9.3E-111
Vim	1.396605	0.947	0.602	7.2E-108
Wasf2	1.101939	0.825	0.212	3.1E-104
Xdh	1.018481	0.703	0.086	8.3E-100
Ybx1	2.171284	0.996	0.569	3.8E-167
Zbtb20	1.006062	0.718	0.247	2.66E-65
Zfp36	1.542778	0.777	0.398	2.16E-64

Seurat FindMarkers TSC2KD vs WT DE (p.adj <0.05, lfc >1) M				
gene	lfc	pct1	pct2	pval.adj
4931406P1	1.115409	0.787	0.189	3.1E-80
Ace	1.635175	0.922	0.282	1.3E-110
Acer2	1.179309	0.773	0.178	2.1E-79
Ackr3	1.245207	0.834	0.46	9.3E-57
Actb	1.386645	0.998	0.954	1.5E-101
Actg1	2.045538	0.985	0.558	4.8E-120
Acvrl1	1.481161	0.931	0.342	1.5E-108
Adamts1	1.521383	0.71	0.323	1.57E-43
Adgre5	1.126216	0.804	0.317	3.32E-64
Adgrf5	1.696031	0.952	0.403	9.7E-117
Adgrl2	1.094604	0.825	0.312	3.68E-71
Adgrl4	1.67785	0.939	0.371	1.7E-114
Ahnak	1.151588	0.872	0.554	2.67E-56
Akap12	1.29143	0.632	0.235	3.67E-40
Aldh2	1.433018	0.89	0.273	1.03E-98
Amigo2	1.202776	0.743	0.216	6.07E-67
Aplp2	1.06469	0.876	0.415	1.81E-71
App	1.367924	0.954	0.497	1.3E-103
Aqp1	2.169751	0.992	0.654	1.5E-137
Arhgap29	1.023399	0.73	0.223	2.04E-60
Arhgap31	1.273867	0.836	0.2	3.59E-93
Arid5b	1.173271	0.766	0.248	1.16E-65
Arl4d	1.119811	0.73	0.21	4.58E-61
Atf3	1.734506	0.693	0.433	1.2E-29
Atp2b1	1.088468	0.75	0.267	4.23E-61
AY036118	2.965828	0.998	0.795	1E-138
B2m	1.822824	0.996	0.779	2E-129
Bgn	1.167946	0.77	0.31	7.39E-59
Bhlhe40	1.054198	0.596	0.216	4.85E-35
Bmpr2	2.272755	0.996	0.569	1.6E-147
Btg1	1.122921	0.81	0.405	9.48E-50
Btg2	2.069859	0.884	0.326	1.2E-99
Calcrl	1.814692	0.998	0.854	9.5E-133
Calm1	1.352525	0.909	0.33	1.07E-98
Cav1	1.390374	0.966	0.592	2.11E-98
Cav2	1.005553	0.802	0.28	8.32E-68
Cavin1	1.081603	0.851	0.478	2.25E-58
Cavin2	1.502652	0.985	0.727	1.1E-107
Ccnl1	1.159927	0.688	0.196	2.07E-56
Cd200	1.527589	0.958	0.69	4.61E-93
Cd36	1.838315	0.998	0.827	6E-138
Cd47	1.529726	0.96	0.481	5.3E-112
Cd74	1.736039	0.682	0.109	2.45E-72
Cd9	1.737108	0.992	0.626	2.4E-121
Cd93	1.77613	0.962	0.49	1E-113
Cdh5	1.532065	0.971	0.554	5.4E-108
Cdkn1a	2.037591	0.836	0.221	1.95E-94
Cebpd	2.082493	0.886	0.431	6.99E-91
Ch25h	1.329575	0.537	0.169	8.83E-34
Chchd2	1.286157	0.909	0.33	1.04E-96
Cldn5	2.225069	0.846	0.405	2.08E-82
Clec14a	1.752858	0.956	0.374	2.5E-120
Clec2d	1.437395	0.876	0.385	1.74E-79
Clic1	1.055215	0.785	0.257	5.1E-68

Clic4	1.379407	0.876	0.426	3.02E-73
Clic5	1.076194	0.785	0.264	8.26E-66
Clk1	1.141723	0.75	0.214	7.29E-68
Crim1	1.498594	0.924	0.501	1.6E-83
Crip1	1.216378	0.863	0.572	1.38E-51
Crip2	1.035844	0.861	0.346	2.77E-72
Csnk1a1	1.175748	0.825	0.212	9.83E-87
Csrp2	1.36558	0.859	0.18	3.2E-101
Cst3	1.243405	0.943	0.531	5.46E-88
Ctgf	1.275336	0.497	0.191	1.18E-23
Ctla2a	2.132075	0.979	0.629	2.8E-119
Ctnna1	1.146572	0.846	0.276	5.34E-83
Cxcl12	1.097562	0.503	0.207	1.04E-22
Cytl1	1.158903	0.59	0.319	2.59E-22
Cyyr1	1.822864	0.964	0.385	1.3E-126
Ddx3x	1.278898	0.844	0.276	5.8E-83
Ddx5	1.665282	0.989	0.572	1.2E-126
Dlc1	1.02505	0.739	0.219	4.44E-61
Dock9	1.041194	0.733	0.132	1.38E-76
Dst	1.189451	0.796	0.212	2.27E-79
Dusp1	1.868541	0.909	0.421	6.26E-94
Dynll1	1.004281	0.758	0.228	9.68E-67
Ece1	1.845936	0.983	0.369	2.6E-132
Eef1a1	2.207294	0.998	0.811	1E-140
Eef2	1.20761	0.829	0.287	2.01E-77
Egfl7	1.806624	0.987	0.59	1.2E-124
EGFP-REPO	2.319833	0.857	0.683	1.98E-60
Egr1	2.038597	0.829	0.604	5.59E-42
Ehd4	1.937597	0.987	0.585	4.2E-129
Eif1	2.014309	0.99	0.56	4.3E-134
Emcn	1.061847	0.777	0.264	6.37E-64
Emp1	1.146294	0.75	0.328	9.94E-44
Eng	1.99213	0.964	0.33	2.2E-132
Entpd1	1.003503	0.634	0.137	8.81E-56
Epas1	2.245673	0.998	0.788	4E-145
Esam	1.2097	0.905	0.364	6.09E-89
Fau	1.827338	0.987	0.661	1.2E-128
Fgl2	1.023694	0.728	0.296	4.37E-48
Fkbp1a	1.138745	0.926	0.499	1.48E-79
Flt1	1.794404	0.983	0.513	5.6E-130
Fmo1	1.354779	0.867	0.212	3E-100
Fmo2	1.565949	0.876	0.276	2.39E-98
Fos	1.767683	0.829	0.636	4.1E-36
Foxf1	1.331439	0.895	0.362	8.69E-92
Foxp1	1.28746	0.987	0.79	9.38E-90
Fth1	1.857076	0.962	0.481	1.8E-122
Ftl1	1.501953	0.93	0.303	4.8E-115
Fus	1.060121	0.752	0.216	1.26E-67
Fzd4	1.002478	0.714	0.21	1.8E-59
Gadd45g	1.193875	0.514	0.221	3.29E-24
Gm13889	1.164137	0.672	0.155	3.08E-59
Gm42418	2.816869	1	1	7.3E-113
Gnai2	1.584024	0.966	0.424	8.5E-121
Gnaq	1.022998	0.823	0.303	2.64E-69
Gnas	1.041292	0.907	0.551	6.38E-69
Gpihbp1	1.335911	0.636	0.164	2.56E-52

Gstm1	1.118817	0.796	0.232	4.13E-76
H2-D1	2.29642	0.996	0.579	4.4E-146
H2-K1	2.047952	0.987	0.588	1.9E-128
H2-Q4	1.051693	0.728	0.223	2.31E-60
H2-Q7	1.359992	0.789	0.173	3.39E-85
H2-T23	1.246135	0.834	0.26	2.36E-80
H3f3a	1.383897	0.935	0.328	5.2E-109
H3f3b	1.972487	0.987	0.56	2.9E-122
Heg1	1.229772	0.895	0.446	2.28E-75
Hes1	1.363392	0.705	0.292	6.6E-45
Hmgb1	1.057562	0.903	0.494	1.06E-71
Hpgd	2.669945	0.994	0.613	1.2E-140
Hsp90ab1	1.304127	0.933	0.538	2.27E-86
Hsp90b1	1.002535	0.79	0.308	3.77E-60
Hspa8	1.518459	0.931	0.326	1.2E-107
Hspb1	1.24893	0.592	0.392	7.86E-13
Icam1	1.693272	0.777	0.285	1.78E-61
Id1	1.083537	0.619	0.13	4.63E-54
Id3	1.51132	0.825	0.353	3.42E-71
Ier2	1.659002	0.863	0.401	1.29E-77
Ier3	2.162185	0.903	0.674	3.96E-69
Ier5	1.166455	0.646	0.169	4.99E-53
Ifitm2	1.570659	0.975	0.631	3.7E-113
Ifitm3	1.933729	1	0.866	7.8E-135
Il6st	2.217908	0.949	0.305	6.6E-131
Irf1	1.280643	0.644	0.335	2.37E-28
Itga1	1.404145	0.844	0.319	3.3E-79
Itgb1	1.273357	0.922	0.387	2.3E-96
Itm2b	2.150905	1	0.677	4.3E-149
Jun	2.038185	0.964	0.743	1.52E-95
Junb	2.252117	0.947	0.695	1.51E-91
Jund	1.77152	0.89	0.394	2.48E-88
Kdr	1.125794	0.789	0.214	1.22E-74
Klf2	2.500257	0.975	0.519	4.7E-133
Klf3	1.014827	0.716	0.169	1.34E-64
Klf4	1.945236	0.968	0.576	1.1E-111
Klf6	1.097971	0.69	0.271	3.73E-44
Klf7	1.200884	0.785	0.278	1.68E-68
Klf9	1.517072	0.842	0.155	1.9E-102
Klhl5	1.261443	0.867	0.323	2.65E-89
Lamp1	1.142083	0.863	0.323	2.92E-82
Laptm4a	1.196779	0.933	0.451	8.44E-92
Lars2	2.479566	0.975	0.515	4.2E-139
Lifr	1.322771	0.857	0.28	3.7E-85
Lima1	1.030473	0.707	0.109	1.18E-74
Lrg1	2.577633	0.91	0.196	1.7E-121
Ly6a	2.365819	0.99	0.67	3E-133
Ly6c1	2.081676	0.926	0.394	5.8E-107
Ly6e	1.99566	0.994	0.654	7.3E-135
Lyve1	2.086458	0.964	0.62	5E-114
Macf1	1.271084	0.882	0.342	1.77E-85
Malat1	2.732844	1	0.998	4.5E-140
Man2a1	1.10094	0.737	0.205	4.55E-65
Marcks	1.094496	0.766	0.376	2.12E-48
Mat2a	1.103672	0.705	0.132	1.18E-71
Mbnl1	1.122926	0.821	0.271	4.31E-76

Mbnl2	1.045182	0.779	0.196	3.31E-73
Mcl1	1.476017	0.846	0.237	1.87E-94
Msn	1.184057	0.893	0.39	2.69E-81
Myl12a	1.01586	0.842	0.367	1.3E-66
Myl12b	1.05489	0.819	0.326	2.66E-67
Myl6	1.17751	0.945	0.547	7.39E-81
Myof	1.024243	0.693	0.191	1.53E-57
Myzap	1.05349	0.684	0.137	6.2E-66
Naca	1.071131	0.802	0.221	7.1E-77
Nfib	1.542239	0.952	0.412	6E-112
Nfkbia	1.931174	0.901	0.44	5.79E-87
Nfkbiz	1.260684	0.629	0.228	8.39E-41
Nr2f2	1.060478	0.743	0.317	2.01E-51
Nrp1	1.283873	0.867	0.271	7.18E-91
Oaz1	1.151199	0.825	0.207	8.13E-90
Pde4b	1.054036	0.655	0.18	9.24E-52
Pecam1	1.729771	0.987	0.565	1.8E-127
Pfdn5	1.013708	0.754	0.194	3.8E-70
Pim3	1.437244	0.741	0.194	3.85E-69
Pltp	1.214696	0.924	0.558	2.46E-74
Plvap	1.27216	0.773	0.36	1.38E-54
Pnrc1	1.384885	0.749	0.171	1.88E-75
Podxl	1.188909	0.867	0.383	1.57E-76
Ppia	1.658058	0.985	0.599	1.8E-125
Ppp1r15a	1.301155	0.724	0.264	1.74E-54
Ppp1r2	1.064424	0.646	0.105	2.51E-64
Prex2	1.109635	0.752	0.18	2.37E-73
Prss23	1.907016	0.96	0.759	1.2E-104
Ptma	1.557872	0.996	0.795	1.3E-108
Ptprb	2.443822	0.998	0.802	1.7E-148
Rac1	1.061124	0.867	0.383	3.09E-75
Rack1	1.118403	0.819	0.317	1.22E-70
Ramp2	1.928614	0.981	0.56	2.4E-121
Rapgef5	1.059263	0.695	0.13	6.13E-68
Raph1	1.06106	0.712	0.169	1.14E-65
Rbm39	1.22132	0.853	0.248	3.86E-90
Rcan1	1.123748	0.606	0.289	6.11E-26
Rdx	1.365033	0.882	0.369	2.44E-82
Rhoa	1.19062	0.933	0.437	1.75E-92
Rhob	1.424612	0.855	0.415	2.84E-71
Rhoj	1.056992	0.8	0.273	1.76E-67
S1pr1	1.860051	0.96	0.428	1.5E-118
Samd5	1.504382	0.905	0.424	1.45E-89
Sat1	1.029538	0.665	0.248	4.43E-38
Sbno2	1.179751	0.657	0.125	1.37E-62
Selenop	1.39737	0.899	0.294	8.5E-99
Sema3c	1.197676	0.653	0.296	2.62E-34
Serf2	1.045767	0.861	0.405	7.17E-68
Serinc3	1.804931	0.975	0.433	6.7E-124
Sgk1	1.334053	0.716	0.182	6.88E-67
Sh3glb1	1.085776	0.785	0.205	7.56E-78
Slc3a2	1.505182	0.848	0.175	7.9E-102
Slc43a3	1.602604	0.935	0.303	3.5E-113
Slc6a2	2.952476	0.937	0.449	2.6E-121
Slc9a3r2	1.078238	0.783	0.232	6.68E-71
Slco2a1	1.435443	0.88	0.371	2.55E-85

Slfn5	1.279231	0.714	0.255	1.08E-51
Smad6	1.103091	0.68	0.155	1.72E-60
Smad7	1.468035	0.825	0.214	1.83E-89
Socs3	1.871727	0.851	0.524	4.53E-72
Son	1.10015	0.81	0.228	1.81E-77
Sptbn1	1.90754	0.973	0.599	8.4E-121
Srgn	1.588098	0.945	0.431	7E-106
Srrm2	1.064904	0.79	0.205	1.06E-78
Srsf5	1.178925	0.811	0.271	1.17E-75
Stt3b	1.10625	0.75	0.123	7.85E-83
Tagln2	1.010839	0.77	0.326	4.85E-55
Tcf4	1.370119	0.918	0.421	9.6E-95
Tek	1.402513	0.937	0.319	3.3E-109
Thbd	1.57523	0.964	0.61	6.3E-100
Timp3	2.196225	0.96	0.458	6.9E-109
Tm4sf1	1.979877	0.968	0.544	3.1E-118
Tmem100	1.510932	0.794	0.424	3.65E-53
Tmem176a	1.163111	0.77	0.182	3.28E-77
Tmem2	1.29928	0.924	0.551	4.14E-78
Tmem252	1.540645	0.642	0.205	2.18E-44
Tmsb4x	1.964531	0.998	0.95	2.7E-134
Tpm3	1.34463	0.832	0.244	6.72E-86
Tpt1	2.553609	1	0.754	2.6E-149
Tspan13	1.090166	0.844	0.317	8.63E-76
Tspan7	1.610471	0.977	0.654	1.5E-117
Uba52	1.660454	0.979	0.572	3.2E-120
Ubb	1.922422	0.992	0.551	4.7E-139
Ubc	1.238946	0.794	0.289	1.72E-68
Ube2d3	1.073143	0.75	0.226	5.65E-65
Ucp2	1.155834	0.67	0.107	1.81E-69
Vcam1	1.55574	0.663	0.323	1.36E-32
Vegfc	1.224823	0.8	0.262	3.01E-73
Vim	1.164771	0.865	0.499	3.75E-58
Vwf	2.547286	1	0.895	1.8E-149
Ybx1	1.970232	0.987	0.487	1.1E-135
Zbtb20	1.162659	0.779	0.296	3.8E-63
Zfp36	2.121179	0.912	0.713	2.19E-67
Zfp36l1	1.599078	0.905	0.446	7.61E-84

Seurat FindMarkers TSC2KD vs WT DE (p.adj <0.05, lfc >1) N				
gene	lfc	pct1	pct2	pval.adj
4931406P1	1.122504	0.748	0.257	3.68E-48
Ace	2.161849	0.986	0.554	3.4E-104
Acer2	1.154227	0.76	0.222	1.04E-50
Actb	1.253383	0.998	0.968	3.83E-66
Actg1	1.765114	0.945	0.443	1.08E-80
Acvrl1	1.506975	0.959	0.519	3.37E-84
Adgre5	1.589026	0.945	0.443	6.16E-82
Adgrf5	2.277026	0.998	0.627	3.8E-113
Adgrl4	1.904737	0.928	0.294	4.58E-93
Aldh2	1.847156	0.945	0.292	6.49E-94
App	1.0508	0.841	0.431	6.16E-45
Aqp1	1.962211	0.976	0.475	2.82E-98
Arap2	1.013919	0.661	0.198	3.3E-39
Arhgap29	1.260601	0.764	0.213	8.39E-59
Arhgap31	1.202644	0.743	0.146	4.05E-61
Arhgef12	1.280329	0.803	0.344	5.7E-52
Arid5b	1.08602	0.637	0.131	7.63E-45
Atf3	1.329745	0.459	0.16	1.51E-18
Atp1a1	1.261445	0.796	0.195	8.87E-64
AY036118	3.011036	1	0.741	2.8E-116
B2m	1.797132	1	0.889	1.5E-110
BC028528	1.395688	0.88	0.353	1.62E-67
Bmpr2	2.402314	0.998	0.697	8.5E-116
Bst2	1.351675	0.856	0.312	7.21E-63
Btg2	1.679126	0.755	0.178	7.1E-60
Calcrl	1.99848	1	0.918	5.8E-103
Calm1	1.33392	0.873	0.332	9.18E-67
Cav1	1.694144	0.981	0.644	3.53E-90
Cav2	1.198422	0.839	0.347	4.47E-58
Cavin2	1.272236	0.935	0.624	7.7E-55
Cbfa2t3	1.059948	0.69	0.181	7.37E-45
Cd200	1.246007	0.817	0.391	4.43E-48
Cd36	1.843112	0.998	0.834	1.9E-104
Cd47	1.727329	0.978	0.44	1.64E-93
Cd74	2.294347	0.755	0.195	8.96E-61
Cd9	1.586459	0.923	0.289	5.68E-83
Cd93	1.810702	0.969	0.601	8.22E-87
Cdh5	1.903611	1	0.755	4.3E-107
Cdkn1a	1.84775	0.817	0.149	4.92E-77
Cebpd	1.912875	0.834	0.259	6.47E-66
Cfl1	1.053049	0.714	0.222	2.67E-46
Chchd2	1.24765	0.827	0.289	1.08E-62
Cldn5	2.537212	0.99	0.609	2E-111
Clec14a	1.840109	0.966	0.449	4.03E-90
Clec1a	1.716495	0.892	0.332	6.92E-77
Clec2d	1.668928	0.966	0.638	1.19E-85
Clic1	1.087483	0.738	0.187	9.25E-54
Clic4	1.083404	0.822	0.353	1.57E-48
Clic5	1.584689	0.904	0.399	2.27E-77
Clk1	1.259754	0.728	0.172	9.69E-56
Crim1	1.18817	0.69	0.19	1.07E-45
Crip1	1.058111	0.632	0.329	2.84E-22
Crip2	1.116078	0.764	0.259	2.72E-50
Csnk1a1	1.07583	0.769	0.224	1.63E-52

Cst3	1.229584	0.827	0.297	5.46E-56
Ctla2a	1.779833	0.921	0.455	1.36E-74
Ctnna1	1.160381	0.788	0.286	2.98E-54
Ctsl	1.061169	0.647	0.163	5.82E-42
Cxcl12	1.96148	0.889	0.51	7.79E-55
Cyp4b1	1.107683	0.721	0.321	6.62E-38
Cyyr1	1.858272	0.969	0.431	7.37E-98
Ddx3x	1.10905	0.719	0.207	6.3E-48
Ddx5	1.733968	0.978	0.542	5.65E-98
Dock9	1.286752	0.805	0.19	4.29E-68
Dusp1	1.511493	0.825	0.335	2.26E-57
Dusp6	1.040708	0.649	0.155	9.15E-43
Dynll1	1.181057	0.788	0.19	4.81E-61
Ece1	1.655453	0.892	0.303	1.1E-79
Edn1	1.086487	0.635	0.461	5.01E-10
Eef1a1	1.991481	0.993	0.638	1.4E-102
Efnb2	1.770336	0.904	0.402	3.85E-73
Egfl7	1.990698	0.998	0.653	2.5E-107
EGFP-REPO	2.804739	0.865	0.499	1.48E-62
Ehd4	1.847205	0.983	0.531	3.1E-99
Eif1	2.101559	0.976	0.341	4.4E-104
Eng	2.127717	0.976	0.42	5.5E-102
Epas1	2.457093	1	0.776	1.3E-115
Esam	1.331486	0.913	0.426	8.07E-74
Fau	1.935006	0.969	0.478	9.47E-96
Fkbp1a	1.171958	0.849	0.414	2.42E-51
Flt1	2.012637	0.981	0.461	5.3E-100
Fmo1	1.713901	0.935	0.373	4.05E-86
Fmo2	1.369177	0.724	0.195	2.25E-52
Fnbp1l	1.053617	0.642	0.152	2.19E-43
Foxf1	1.352155	0.87	0.391	8.39E-65
Foxp1	1.278105	0.928	0.554	7.58E-59
Fth1	1.477274	0.899	0.344	2.43E-74
Ftl1	1.348257	0.87	0.233	1.87E-72
Fzd4	1.048412	0.688	0.172	1.34E-45
Gata2	1.10171	0.683	0.201	6.18E-43
Gbp7	1.195243	0.793	0.399	5.38E-41
Gm42418	2.511109	1	1	3.98E-98
Gnai2	1.480788	0.945	0.446	7.58E-80
Gpihbp1	2.053785	0.952	0.434	9.84E-91
Gstm1	1.080234	0.757	0.292	1.37E-44
H2-Aa	1.183885	0.635	0.181	8.9E-39
H2-Ab1	1.610628	0.716	0.187	3.93E-54
H2-D1	2.548328	1	0.679	2E-118
H2-Eb1	1.304144	0.63	0.131	4.6E-45
H2-K1	2.18498	1	0.735	3E-112
H2-Q4	1.517217	0.904	0.297	3.43E-79
H2-Q6	1.466995	0.815	0.242	4.76E-65
H2-Q7	1.828337	0.947	0.356	5.19E-90
H2-T23	1.652575	0.894	0.265	2.88E-85
H3f3a	1.454131	0.928	0.324	6.02E-83
H3f3b	1.843388	0.971	0.475	4.89E-86
Hilpda	1.554625	0.776	0.248	1.74E-57
Hmgb1	1.28712	0.916	0.472	7.28E-66
Hnrnpa2b1	1.074841	0.762	0.259	7.02E-50
Hopx	1.124816	0.63	0.219	3.18E-34

Hpgd	2.406313	0.981	0.697	1.05E-98
Hsp90ab1	1.3423	0.868	0.399	7.74E-60
Hsp90b1	1.056153	0.733	0.268	1.03E-41
Hspa8	1.332829	0.88	0.28	6.46E-74
Icam2	1.372778	0.882	0.353	3.4E-70
Id1	1.166843	0.666	0.169	1.87E-45
Id3	1.654787	0.877	0.332	2.6E-71
Ier2	1.252659	0.666	0.204	1.6E-40
Ier3	1.682501	0.793	0.397	2.75E-42
Ifi203	1.079094	0.649	0.201	1.31E-37
Ifi47	1.062851	0.659	0.143	1.09E-45
Ifitm2	1.489856	0.95	0.431	1.15E-76
Ifitm3	1.917776	1	0.918	1.8E-104
Iigp1	1.547213	0.755	0.274	2.77E-48
Il6st	1.011467	0.623	0.128	5.95E-43
Itga1	1.673337	0.942	0.507	3.21E-78
Itgb1	1.282592	0.851	0.303	1.15E-61
Itm2b	2.301787	0.993	0.755	3.6E-111
Jun	2.018039	0.988	0.627	2.68E-92
Junb	1.759416	0.784	0.382	2.62E-40
Jund	1.727056	0.793	0.265	3.29E-59
Jup	1.084525	0.76	0.324	7.07E-44
Kdr	1.129011	0.731	0.251	1.19E-44
Kitl	1.321361	0.642	0.201	4.99E-38
Klf2	2.143781	0.952	0.461	4.45E-94
Klf4	1.81597	0.889	0.373	6.15E-71
Klf7	1.330274	0.788	0.242	5.82E-58
Klf9	1.492019	0.793	0.143	2.74E-71
Klhl5	1.104786	0.731	0.216	1.97E-49
Lamp1	1.227344	0.875	0.312	8.56E-67
Laptm4a	1.231761	0.882	0.391	6.3E-63
Lars2	2.33286	0.995	0.394	1E-114
Lpl	2.46589	0.911	0.178	1.88E-97
Ly6a	2.520705	1	0.86	1.3E-116
Ly6c1	2.542282	0.995	0.577	2.8E-110
Ly6e	2.092676	1	0.77	4.8E-110
Lyve1	1.34827	0.411	0.122	1.25E-17
Macf1	1.170845	0.762	0.274	1.36E-48
Malat1	2.602429	1	0.991	1.8E-113
Marcks	1.154455	0.666	0.175	2.49E-43
Mcl1	1.309077	0.74	0.117	5.77E-65
Mctp1	1.137642	0.582	0.067	1.01E-46
Mgll	1.016645	0.502	0.07	1.81E-35
Morf4l1	1.066253	0.776	0.277	3.75E-50
Msn	1.341508	0.899	0.373	3.03E-69
Mxd4	1.021069	0.659	0.122	1.82E-49
Myl12a	1.054242	0.815	0.353	2.53E-47
Myl12b	1.29331	0.882	0.318	1.01E-71
Myl6	1.151229	0.849	0.408	5.55E-51
Myzap	1.142954	0.728	0.216	1.76E-49
Naca	1.015844	0.656	0.125	6.91E-49
Ndufa4	1.007027	0.688	0.152	5.62E-50
Nfib	1.520024	0.918	0.443	4.83E-74
Nfkbia	1.260534	0.769	0.239	4.67E-55
Notch1	1.069548	0.721	0.213	3.11E-47
Nrp1	1.484219	0.909	0.356	1.52E-78

Oaz1	1.279976	0.772	0.102	2.61E-72
Pcdh17	1.103346	0.663	0.239	4.14E-35
Pde4b	1.294423	0.697	0.146	6.01E-54
Pecam1	1.872582	0.995	0.536	3.1E-106
Pitpnc1	1.103025	0.745	0.344	1.99E-37
Pltp	1.443904	0.954	0.612	5.24E-71
Plvap	1.446133	0.721	0.391	3.43E-33
Podxl	1.048072	0.781	0.359	3.5E-42
Ppia	1.668971	0.962	0.458	1.92E-91
Prrc2c	1.00483	0.695	0.23	6.83E-41
Psmb8	1.147205	0.733	0.137	1.36E-60
Ptma	1.781882	0.995	0.761	4.38E-94
Ptprb	2.385561	1	0.793	1.7E-110
Qk	1.241748	0.825	0.332	4.34E-54
Rabac1	1.025542	0.709	0.155	4.86E-52
Rac1	1.010865	0.781	0.332	2.24E-43
Ramp2	2.298076	0.993	0.7	7.6E-109
Rap1a	1.04794	0.671	0.14	7.95E-49
Rapgef5	1.051144	0.695	0.192	1.61E-45
Rbm39	1.35225	0.841	0.207	5.95E-73
Rdx	1.651385	0.861	0.294	5.59E-70
Rhoa	1.204778	0.885	0.362	1.16E-66
Rsrp1	1.068202	0.661	0.111	8.91E-52
S100a13	1.053895	0.707	0.175	1.27E-48
S1pr1	2.264171	0.983	0.466	1.6E-106
Sec62	1.140006	0.74	0.195	2.51E-52
Selenop	1.783542	0.942	0.399	5.13E-84
Sema3c	1.286091	0.668	0.429	7.22E-19
Sema3g	1.399898	0.803	0.236	2.48E-61
Serbp1	1.02047	0.692	0.242	1.97E-40
Serf2	1.087668	0.752	0.248	9.14E-50
Serinc3	2.365481	0.99	0.469	1.3E-110
Sh3glb1	1.305448	0.8	0.172	3.04E-66
Slc16a9	1.047574	0.63	0.21	6.84E-34
Slc3a2	1.355616	0.784	0.163	1.32E-67
Slc43a3	1.368764	0.865	0.242	5.7E-75
Slc9a3r2	1.48735	0.865	0.268	1.27E-76
Slco2a1	1.876443	0.94	0.44	1.65E-86
Slfn5	1.615294	0.851	0.303	1.83E-67
Smad6	1.404946	0.774	0.274	4.09E-53
Smad7	1.498703	0.856	0.257	4.12E-73
Socs3	1.233648	0.599	0.254	2.27E-26
Son	1.199416	0.8	0.236	1.78E-61
Sparcl1	1.415965	0.752	0.292	5.55E-48
Sptan1	1.007204	0.702	0.195	3.05E-47
Sptbn1	1.980097	0.995	0.636	2.8E-101
Srrm2	1.201552	0.788	0.175	4.13E-65
Srsf5	1.180458	0.776	0.224	2.89E-56
Tagln2	1.073183	0.721	0.265	5.34E-41
Tax1bp1	1.544294	0.772	0.181	5E-63
Tcf4	1.683624	0.947	0.475	1.27E-85
Tcn2	1.18041	0.688	0.163	6.55E-49
Tek	1.521099	0.882	0.315	1.14E-75
Thbd	1.230832	0.837	0.475	3.78E-42
Timp3	1.386302	0.661	0.146	8.44E-48
Tjp1	1.140904	0.793	0.294	1.71E-52

Tm4sf1	1.674672	0.921	0.548	6.57E-64
Tmbim6	1.092379	0.822	0.274	4.41E-57
Tmem100	1.945226	0.978	0.819	5.41E-94
Tmem176a	1.170487	0.721	0.178	1.81E-52
Tmem176b	1.008723	0.697	0.236	1.97E-41
Tmem59	1.021942	0.74	0.178	1.07E-52
Tmsb4x	1.801535	0.995	0.886	1.59E-96
Tnfsf10	1.022457	0.733	0.332	4.34E-35
Tpm3	1.042159	0.683	0.172	1.83E-45
Tpt1	2.331679	0.995	0.554	1.2E-112
Tspan13	1.406064	0.913	0.397	2.22E-74
Tspan18	1.035125	0.647	0.175	2.42E-40
Tspan7	1.664097	0.988	0.691	3.63E-90
Txnip	1.228832	0.829	0.414	4.73E-47
Uba52	1.76405	0.952	0.379	5.23E-92
Ubb	2.086317	0.993	0.504	5.8E-109
Ucp2	1.207315	0.685	0.134	2.46E-53
Vegfa	1.57139	0.913	0.449	1.63E-70
Vwf	1.638447	0.62	0.146	6.35E-41
Xdh	1.15515	0.685	0.102	7.64E-58
Ybx1	1.890616	0.942	0.353	4.3E-91
Zbtb20	1.168141	0.712	0.265	4.84E-41
Zfp36	1.325898	0.661	0.257	2.72E-32
Zfp36l1	1.086355	0.712	0.271	5.85E-42

Supplemental Table 6. Terms extracted from MSIGDB mouse database to probe enrichment for three pathway/functions in MVPC single cells as shown in Supplementary Figure 8

MTOR RELATED TERMS

BIOCARTA_MTOR_PATHWAY

HALLMARK_MTORC1_SIGNALING

HALLMARK_PI3K_AKT_MTOR_SIGNALING

REACTOME_MTOR_SIGNALLING

REACTOME_MTORC1_MEDIATED_SIGNALLING
504 unique gene symbols – 440 genes in dataset

AUTOPHAGY RELATED TERMS

GOBP_LYSOSOMAL_MICROAUTOPHAGY

GOBP_MACROAUTOPHAGY

GOBP_SELECTIVE_AUTOPHAGY

REACTOME_MACROAUTOPHAGY

REACTOME_SELECTIVE_AUTOPHAGY
328 unique gene symbols – 303 genes in dataset

APOPTOSIS/SENESENCE RELATED TERMS

GOBP_CELLULAR_SENESCENCE

REACTOME_CELLULAR_SENESCENCE

REACTOME_DNA_DAMAGE_TELOMERE_STRESS_INDUCED_SENESCENCE

REACTOME_OXIDATIVE_STRESS_INDUCED_SENESCENCE

REACTOME_APOPTOSIS

REACTOME_APOPTOSIS_INDUCED_DNA_FRAGMENTATION

REACTOME_INTRINSIC_PATHWAY_FOR_APOPTOSIS
511 unique gene symbols – 383 genes in dataset

Supplemental Table 7. Primer & Antibody Specifications

**WESTERN BLOT**			
**B-ACTIN**	Ab8227	1:1000	Abcam, Cambridge, United Kingdom
**HAMARTIN/TSC1 (1B2)**	4963S	1:500	Cell Signaling, Danvers, MA
**TUBERIN/TSC2(D93F12)**	4308S	1:500	Cell Signaling, Danvers, MA
**PHOSPHO-S6 (SER240/244)**	5364S	1:1000	Cell Signaling, Danvers, MA
**S6 (54D2)**	2317S	1:500	Cell Signaling, Danvers, MA
**PHOSPHO-S6 (SER235/236)**	2211S	1:1000	Cell Signaling, Danvers, MA
**P21 (WAF1/CIP1-12D1)**	2947	1:1000	Cell Signaling, Danvers, MA
**P16 (INK4A E6N8P)**	18769	1:1000	Cell Signaling, Danvers, MA
**MDM2 (D1V2Z)**	86934	1:1000	Cell Signaling, Danvers, MA
**MTORC2(PHOSPHO-AKT SER473**	31957	1:2000	Cell Signaling, Danvers, MA
**P53 (7F5) RABBIT MAB**	2527	1:1000	Cell Signaling, Danvers, MA
**SER15 (PHOSPHO-P53 ANTIBODY)**	9284	1:1000	Cell Signaling, Danvers, MA
**B-ACTIN (13E5)**	4970	1:1000	Cell Signaling, Danvers, MA
**DONKEY ANTI-RABBIT HRP**	711-035-152	1:1000	Jackson ImmunoResearch, West Grove, PA
**ANTI-MOUSE IGG-HRP**	7076	1:2000	Cell Signaling, Danvers, MA
**ANTI-RABBIT IGG-HRP**	7074	1:2000	Cell Signaling, Danvers, MA
**IMMUNOSTAIN**			Cell Signaling, Danvers, MA
**TUBERIN/TSC2 (D93F12)**	4308	1:500	Cell Signaling, Danvers, MA
**PHOSPHO-S6 (SER235/236)**	2211	1:400	Cell Signaling, Danvers, MA
**SMOOTH MUSCLE ACTIN (1A4)**	M0851	1:500	DAKO, Carpinteria, CA
**FACTOR VIII**	A0082	1:500	DAKO, Carpinteria, CA
**MURINE FLOW CYTOMETRY**			
**CD45-APC**	17-0451-83		eBioscience, San Diego, CA
**TER119**	48-5921-82		eBioscience, San Diego, CA
**CD105-APC**	120413		Biolegend, San Diego, CA
**CD73-APC**	127209		Biolegend, San Diego, CA
**CD146-APC**	134711		Biolegend, San Diego, CA
**CD140B-APC**	136007		Biolegend, San Diego, CA
**CD44-APC**	103011		Biolegend, San Diego, CA
**CD F4/80-APC**	17-4801-80		eBioscience, San Diego, CA
**CD45-APC –EF780**	47-0451-82		eBioscience, San Diego, CA
**CD14-APC**	17-0141		eBioscience, San Diego, CA
**SCA-1-PE**	12-5981-82		eBioscience, San Diego, CA
**CD106**	105717		Biolegend, San Diego, CA
**CD3-APC**	17-0032		eBioscience, San Diego, CA
**HUMAN FLOW CYTOMETRY**			
**CD45-APC**	17-9459-42		eBioscience, San Diego, CA
**CD144-PE**	12-1449		eBioscience, San Diego, CA
**CD105-PE**	12-1057		eBioscience, San Diego, CA
**CD106-PE**	12-1069		eBioscience, San Diego, CA
**CD140B-PE**	558821		BD Pharmingen, San Jose, CA
**CD31-FITC**	11-0319		eBioscience, San Diego, CA
**CD34-FITC**	11-0349		eBioscience, San Diego, CA
**CD14-FITC**	11-0149		eBioscience, San Diego, CA
**CD44-FITC**	11-0441		eBioscience, San Diego, CA
**CD73-APC**	17-0739		eBioscience, San Diego, CA
**SECONDARY ABS**			
**ANTI-RAT IGG**	A-110007	1:500	Invitrogen, Waltham, MA
**ANTI-RABBIT IGG**	A-11012	1:500	Invitrogen, Waltham, MA

**ANTI-MOUSE IGG**	A-11005	1:500	Invitrogen, Waltham, MA
**ANTI-RAT IGG**	A-11006	1:500	Invitrogen, Waltham, MA
**ANTI-MOUSE IGG**	A-11029	1:500	Invitrogen, Waltham, MA
**ANTI-RABBIT IGG**	A-11008	1:500	Invitrogen, Waltham, MA
**ANTI-GOAT IGG**	A-11078	1:500	Invitrogen, Waltham, MA
**HUMAN PCR PRIMERS**			
GAPDH	4326317E		Applied Biosystems, Foster City, CA
BRCA1	HS01556193_m1		Applied Biosystems, Foster City, CA
BRCA2	HS00609073_m1		Applied Biosystems, Foster City, CA
BRIP1	HS00908144_m1		Applied Biosystems, Foster City, CA
SOD2	HS00167309_m1		Applied Biosystems, Foster City, CA
HMMR	HS00234864_m1		Applied Biosystems, Foster City, CA
DRAM1	HS01022842_m1		Applied Biosystems, Foster City, CA
